# Environmental Factors Shaping the Diversity and Spatial-Temporal Distribution of Indoor and Outdoor Culturable Airborne Fungal Communities in Tianjin University Campus, Tianjin, China

**DOI:** 10.3389/fmicb.2022.928921

**Published:** 2022-06-14

**Authors:** Chaonan Yuan, Xiao Wang, Lorenzo Pecoraro

**Affiliations:** School of Pharmaceutical Science and Technology, Tianjin University, Tianjin, China

**Keywords:** airborne fungi, indoor, outdoor, fungal diversity and community structure, concentration, morphology, ITS sequencing, environmental factors

## Abstract

Airborne fungi have significant influence on air quality and may pose adverse effects on human health due to their allergenic and pathogenic effect. We carried out a 1-year survey on the airborne fungal diversity and concentration of indoor and outdoor environments, within the Tianjin University campus. Airborne fungi were sampled using an HAS-100B air sampler. Isolated fungal strains were identified based on morphological and molecular analysis. A total of 641 fungal strains belonging to 173 species and 74 genera were identified in this study. The dominant fungal genera were *Cladosporium* (29.49%), *Alternaria* (25.9%), and *Epicoccum* (6.24%), while the most frequently occurring species were *A. alternata* (15.44%), *C. cladosporioides* (11.86%), and *E. nigrum* (5.77%). The mean fungal concentration at different sites ranged from 0 to 420 CFU/m^3^, which is lower than the permissive standard level. There was a seasonal variation in the airborne fungal community, while temperature and relative humidity were positively correlated with the fungal concentration and diversity at almost all the sites. Higher fungal diversity was recorded in peak period of human traffic at the two canteens, whereas the two dormitories showed higher fungal diversity in off-peak period. Our study provides the first report on the diversity and concentration of airborne fungal species within different Tianjin University campus environments and clarifies the role played by environmental factors on the analyzed fungal community. Our results may represent valuable information for air quality monitoring and microbial pollution control in densely populated human living environments.

## Introduction

Fungi are one of the most abundant group of organisms on earth and are considered ubiquitous in our living environment (Pei-Chih et al., 2000). These organisms can grow on a large variety of substrates and play different roles in the environment as symbionts, saprotrophs, or parasites (Cvetnić and Pepeljnjak, 1997). Most of the airborne fungi originate from natural sources including soil, lakes, plant, animal, and human hosts, while many industrial operations such as sewage treatment, animal rendering, fermentation processes, and agricultural activities increase the emission of viable microorganisms into the air (Bovallius et al., 1978; Di Giorgio et al., 1996). Fungi commonly occur in the air as particulate spores and cells, while elevated fungal concentrations are correlated with air pollution, and were proposed as a cause of adverse health effects on humans, animals, and plants (Harrison et al., 1992; Bush and Portnoy, 2001; Ren et al., 2001; Kalyoncu, 2010). Certain fungi and their metabolic products, such as mycotoxins, can increase the occurrence of numerous respiratory anomalies and diseases, including wheezing, coughing, shortness of breath, chronic obstructive pulmonary disease (COPD), asthma, allergies, and decreased lung function (Cox, 1989; Flannigan et al., 1991; Halonen et al., 1997; Li and Hsu, 1997; Hargreaves et al., 2003; Fang et al., 2005). Since the first case of asthma attack was reported to be caused by fungal spores in 1726 (Floyer, 1745), more than 80 genera of fungi have been reported to be mainly responsible for respiratory tract allergies (Horner et al., 1995). Over 100 species of fungi have been found to be involved in serious human and animal infections, whereas many other species cause plant diseases (Cvetnić and Pepeljnjak, 1997).

Airborne fungi have received much attention from medical and environmental researchers because of their potential health hazards. Numerous studies on fungal biodiversity in both indoor and outdoor environments (Pavan and Manjunath, 2014), such as hospitals, railway stations, agricultural areas (Björnsson et al., 1995; Newson et al., 2000; Meklin et al., 2002; Adhikari et al., 2004; Kawasaki et al., 2010; Harkawy et al., 2011), classrooms, libraries, and dormitories of educational institutions have been performed (Karwowska, 2003; Karbowska-Berent et al., 2011; Freitas et al., 2012; Kalwasińska et al., 2012; Faridi et al., 2015; Li et al., 2015). Air fungal concentrations in domestic environments have been investigated in numerous studies. For instance, elevated concentrations of *Aspergillus niger* have been found in dust samples from houses where asthmatic children lived, while *A. ochraceus*, *A. unguis*, and *Penicillium* species presence in house dust has been associated with childhood asthma (Reponen et al., 2012; Vesper et al., 2013). The fungal genera *Cladosporium*, *Alternaria*, *Aspergillus*, and *Fusarium* were dominant in the air, from studies carried out at a clinic in Iran (Mirhoseini et al., 2021), a rural agricultural area in India (Adhikari et al., 2004), and indoor-outdoor of buildings in the United States (Shelton et al., 2002). In the latter study, *Alternaria*, *Aspergillus*, and *Cladosporium* were reported to be responsible for allergic rhinitis or asthma (Shelton et al., 2002). Previous studies have shown that the concentrations of the airborne fungi are influenced by local environmental variables, fungal substrates, and human activities (Banerjee et al., 1987; Hameed and Khodr, 2001; Lu et al., 2022). Researchers have demonstrated that indoor and outdoor diversity and concentration of fungal spores vary throughout the day depending on weather parameters (Rodriguez-Rajo et al., 2004). The distribution of airborne fungi has been found to be influenced by environmental factors including type of vegetation, air pollution, human activities, meteorological factors (temperature, wind speed, relative humidity, etc.), and seasonal variations (Lin and Li, 2000; Pepeljnjak and Šegvić, 2003; Rossi et al., 2005). Because pathogenic and allergenic fungal species dispersed by the airflow may affect human health (Maki et al., 2011), it is important to assess and monitor the distribution of fungi in the air, and understand the influence of environmental factors in shaping the airborne fungal communities. A better knowledge of airborne fungal diversity in different environments can enrich our baseline understanding of the large-scale distribution of fungi in the atmosphere, and support management activities related to public health and global health security.

All year round, millions of students and staff of educational institutions in China spend several hours every day in campus places including canteens, libraries, and dormitories. They are exposed to microbes present in the air, which ultimately may impact their health and wellbeing. Thus far, several studies on airborne fungal diversity from different indoor and outdoor environments in university campuses in China have been published. Li et al. (2015) assessed concentration and distribution of airborne culturable fungi in four types of indoor environments, including canteen, classroom, clinic, and student dormitory at Chang’ an University, China. According to the results of the latter study, the highest mean concentration of fungi was found in the canteen, followed by the clinic and dormitories, and the lowest in classrooms. The results also showed that concentration and distribution of airborne fungi varied in the analyzed indoor environments due to the presence of different sources and human occupancy (Li et al., 2015). A study on airborne fungal diversity conducted in Shenzhen University revealed that the abundance of most recorded fungal spores decreased along with the increasing altitude from the ground level (Li et al., 2010).

Our study aimed to assess the air fungal diversity of six most frequently occupied places in the campus of Tianjin University, Tianjin, China. We also analyzed the influence of several environmental factors on the studied fungal concentration and community structure. Results from the present research extend our knowledge of airborne fungal biodiversity in university campuses and may provide support in air quality improvement efforts in densely populated human living environments.

## Materials and Methods

### Sampling Sites

Sampling of airborne fungi was carried out in Tianjin University campus, located in Tianjin city, China. Tianjin University is the oldest institution of higher education in the modern history of China, founded in 1895 as Peiyang University. The University has two campuses, one located in Jinnan district, and another in Nankai district, with about 38,500 students in total. The campus of our interest for the study is the oldest one, located in Nankai district, more specifically at Weijin Road, with a gross floor area of 1,380,000 m^2^. This University campus is surrounded by residential dwellings, public buildings, and several asphalt roads.

**FIGURE 1:**
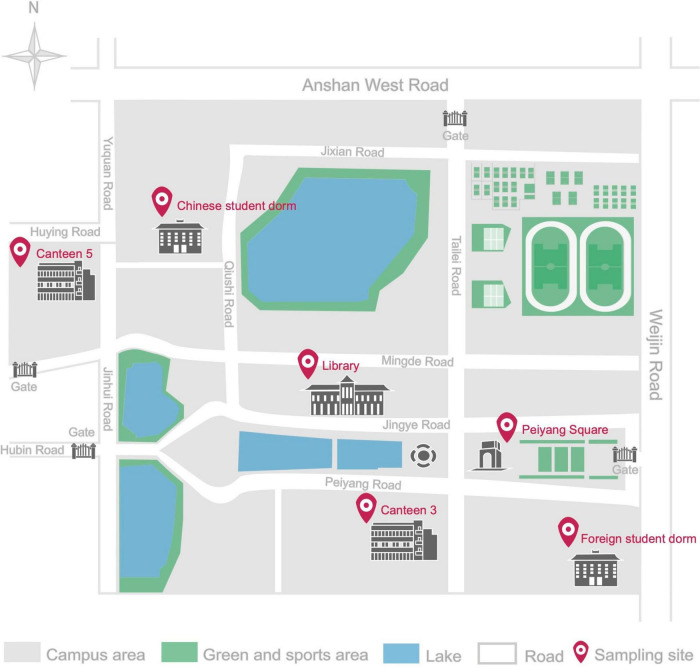
Schematic diagram of Tianjin University Campus in Nankai district, with detailed location of the six sampling sites.

Six different environments, including five indoor and one outdoor, were selected to locate the sampling sites in the studied University campus. The indoor environments included 2 canteens, 2 dormitories, and the library, while the central Peiyang square was chosen as the outdoor environment. Details and locations of the sampling sites are reported in Table 1 and Figure 1. Two distinct sampling times were selected to fully understand the human traffic influence on the airborne fungal community, (1) the off-peak period sampling time, when there were no or there were very limited human activities, and the peak period sampling time, when there were maximum levels of human presence and activities.

**TABLE 1:** Characteristics of the six sampling sites.

Location	Site	Sampling period	Code	Sampling time	GPS position
Canteen 3	Hall (first floor)	Peak	3CP	11:30 a.m.–12:30 p.m.	117^°^18′16″N, 39^°^11′27′E
		Off-peak	3CO	14:00 p.m.–15:00 p.m.	
Canteen 5	Hall (second floor)	Peak	5CP	11:30 a.m.–12:30 p.m.	117^°^17′46″N, 39^°^11′72″E
		Off-peak	5CO	14:00 p.m.–15:00 p.m.	
Chinese student dorm	Corridor (first floor)	Peak	CDP	20:00 p.m.–22:00 p.m.	117^°^17′69″N, 39^°^11′74″E
		Off-peak	CDO	15:30 p.m.–16:30 p.m.	
Foreign student dorm	Corridor (first floor)	Peak	FDP	20:00 p.m.–22:00 p.m.	117^°^18′43″N, 39^°^11′26″E
		Off-peak	FDO	15:30 p.m.–16:30 p.m.	
Library	Corridor (second floor)		LIB	17:00 p.m.–18:00 p.m.	117^°^17′93″N, 39^°^11′49″E
Peiyang square	Square center		PS	17:00 p.m.–18:00 p.m.	117^°^18′10″N, 39^°^11′39″E

### Sampling Method and Culture Media

In all the selected sites, sampling was conducted every month for 1 year, from June 2020 to May 2021, in a single day at the two above-mentioned different times. Air samples were collected using a HAS-100B sampler (Hengao T&D, China) operated at a flow rate of 100 L/min and rotating dish speed of 0–4 rpm for 10 min at each location. Before each sampling, the sampler was swabbed using 70% ethanol to prevent cross-contamination. The samples were collected at 1.2–1.5 m height, which represents the human breathing zone. For each air sampling, one petri dish (9.0 cm diameter) containing Malt Extract Agar (MEA) amended with an antibiotic (ampicillin, at 100 mg/L) to prevent bacterial growth, was used. In order to evaluate the influence of environmental factors on the studied airborne fungal communities, humidity (%) and temperature (°C) were measured in each location during each sampling, using a TES 1364 Humidity-Temperature Meter (Hengao T&D, China). After field work, the plates were incubated at 25°C for 5–7 days, in the darkness, and examined every 24 h to detect fungal growth. Observed fungal colonies in each plate were counted, picked up, and inoculated in new plates (6.0 cm diameter) for isolation.

### Fungal Identification

All the isolated strains were identified based on molecular and morphological analyses. For the molecular identification of isolated strains, fungal genomic DNA was extracted using the hexadecyl trimethyl ammonium bromide (cTAB) method (Möller et al., 1992), and PCR amplification of the internal transcribed spacer (ITS) region was performed using the universal primers pITS4-F (5′-TCCGTAGGTGAACCTG CCG-3′) and pITS1-R (5′-TCCTCCGCTTATTGATATGC-3′) according to White et al. (1990). The reaction mixture (25 μL) consisted of 1.0 μL of genomic DNA, 1.0 μL of each primer, 9.5 μL of deionized water, and 12.5 μL of 2 × T5 Super PCR Mix (Tsingke Biotechnology Co., Ltd., Beijing). The amplification program was as follows: initial denaturation at 98°C for 3 min, 35 cycles of 98°C for 10 s, annealing at 55°C for 10 s, extension at 72°C for 15 s, followed by a final extension at 72°C for 2 min. The PCR products were detected using electrophoresis on a 1% agarose gel and sent to Genewiz, Tianjin, China for sequencing. Sequence analysis was conducted with BLAST searches against the National Center for Biotechnology Information (NCBI) sequence database (GenBank)^1^ to determine the closest sequence matches that enabled taxonomic identification. DNA sequences were deposited in GenBank (Accession Nos. OM236671-OM237311).

Fungal morphology was characterized based on macroscopic and microscopic observations. Microscopy was done using a Nikon ECLIPSE Ci-L microscope (Tokyo, Japan) to examine fungal morphological characters, such as hyphae, pseudohyphae, conidiophores, conidia, poroconidia, arthroconidia, etc. (Wang and Pecoraro, 2021). All isolated fungal strains were deposited in the LP Culture Collection (personal culture collection held in the laboratory of Prof. Lorenzo Pecoraro), at the School of Pharmaceutical Science and Technology, Tianjin University, Tianjin, China.

### Enumeration of Fungi

Number of fungal colonies observed at each sampling location for each month were recorded. The data were transformed to colony forming units per cubic meters (CFU/m^3^) according to the formula below:

$$N=\frac{\text{Cn}\times 1000}{t\times V}$$

Where *N* = concentration of fungal colonies in CFU/m^3^, C_n_ = Number of fungal colonies, 1,000 = conversion factor of liter to cubic meter, *t* = sampling operation time, and V = velocity of the air flow (Fang et al., 2005).

### Statistical Analysis

Krona Charts illustrating the composition of fungal communities were made using Krona Tools (Ondov et al., 2011).^2^ The fungal diversity of different sampling locations was evaluated using Shannon index through multi-Variate v. 3.22 (MVSP 3.22). Pearson correlation analysis was conducted through SPSS version 22.0. One-way analysis of variance (ANOVA) was used to compare the mean fungal concentration at different sampling sites. Principal Co-ordinates Analysis (PCoA) and Analysis of Similarity (ANOSIM) with 999 permutations were performed based on the Bray–Curtis distance in R package. Permutational Multivariate Analysis of Variance (PERMANOVA) based on Bray–Curtis distance was performed to analyze the effects of various factors on the whole fungal diversity.

## Results

### Fungal Strains Isolated at Different Locations and Months

**FIGURE 2:**
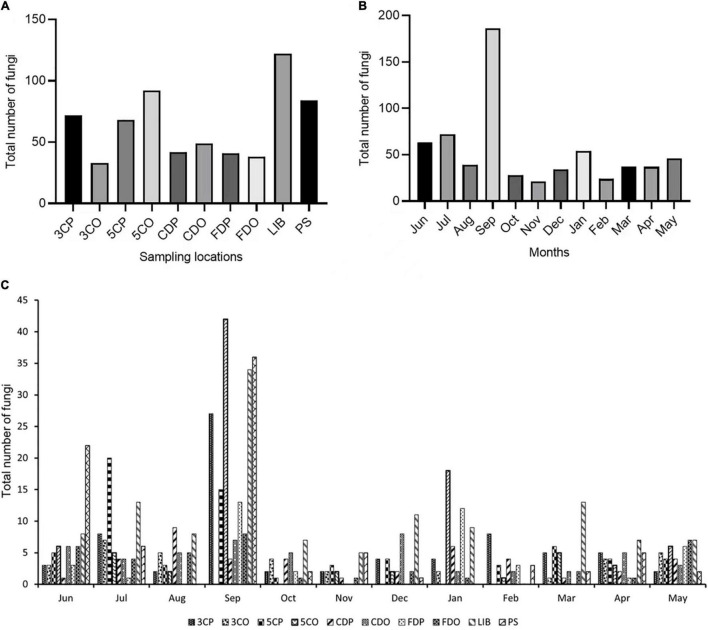
Total number of fungi isolated from different locations **(A)** and months **(B,C)**. 3CP, Canteen 3 Peak; 3CO, Canteen 3 Off-peak; 5CP, Canteen 5 Peak; 5CO, Canteen 5 Off-peak; CDP, Chinese student Dorm Peak; CDO, Chinese student Dorm Off-peak; FDP, Foreign student Dorm Peak; FDO, Foreign student Dorm Off-peak; LIB, Library; PS, Peiyang Square.

In this study, we isolated 641 fungal strains from 6 sampling sites for a period of 1 year (from June 2020 to May 2021). We isolated the highest number of strains (122 strains) from the library (LIB), followed by canteen 5 off-peak period (5CO) and Peiyang square (PS), which provided 92 and 84 strains, respectively (Figure 2). The canteen 3 peak period (3CP), canteen 5 peak period (5CP), and Chinese student dormitory off-peak period (CDO) yielded 72, 68, and 49 strains, respectively. Chinese student dormitory peak period (CDP), foreign student dormitory peak period (FDP) and off-peak period (FDO), and canteen 3 off-peak period (3CO), harbored a much lower number of fungi, with a total of 42, 41, 38, and 33 strains, respectively (Figure 2A).

The month of September showed a significantly higher number of strains (186) compared to all the other months during the year of study (Figure 2B). The majority of September strains was collected from 5CO (42), LIB (36), PS (34), and 3CP (27), while 5CP, FDP, FDO, CDO, and CDP showed a lower fungal richness, with 15, 13, 8, 7, and 4 isolated strains, respectively. Canteen 3 off-peak period (3CO) did not show any fungal presence in September (Figure 2C). The second airborne fungal richest month was July (72 strains), followed by June (63 strains), while the lowest numbers of strains were isolated in November (21), February (24), and October (28) (Figures 2B,C).

### Fungal Concentration in the Sampling Sites

**FIGURE 3:**
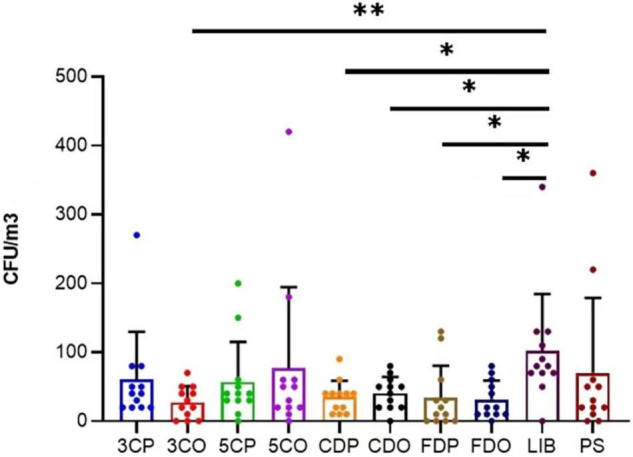
Variation of annual average fungal concentration (CFU/m^3^) at different sampling sites. ^**^P < 0.01, *P < 0.05. 3CP, Canteen 3 Peak; 3CO, Canteen 3 Off-peak; 5CP, Canteen 5 Peak; 5CO, Canteen 5 Off-peak; CDP, Chinese student Dorm Peak; CDO, Chinese student Dorm Off-peak; FDP, Foreign student Dorm Peak; FDO, Foreign student Dorm Off-peak.

**FIGURE 4:**
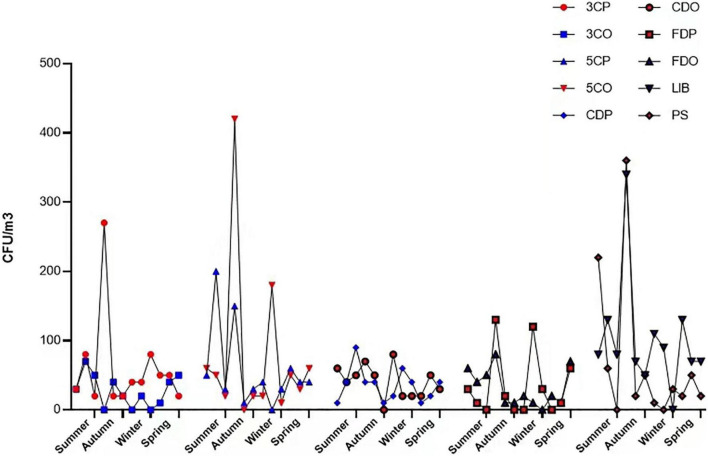
Fungal colony concentration variations in each location at different seasons. 3CP, Canteen 3 Peak; 3CO, Canteen 3 Off-peak; 5CP, Canteen 5 Peak; 5CO, Canteen 5 Off-peak; CDP, Chinese student Dorm Peak; CDO, Chinese student Dorm Off-peak; FDP, Foreign student Dorm Peak; FDO, Foreign student Dorm Off-peak; LIB, Library; PS, Peiyang Square.

The annual average concentration of culturable airborne fungi in each sampling site was analyzed in colony-forming units per cubic meter of air (CFU/m^3^) (Table 2). Considering all the sampling sites, the concentration ranged from 0 to 420 CFU/m^3^. LIB had the highest annual mean fungal concentration of 101.67 CFU/m^3^, while the 3CO had the lowest (27.50 CFU/m^3^). The differences of average fungal concentration among all the sampling sites revealed that the presence of fungi in the air of LIB was significantly higher than that of 3CO (^**^P < 0.01), CDP (*P < 0.05), CDO (*P < 0.05), FDP (*P < 0.05), FDO (*P < 0.05) (Figure 3). Looking at the effect of human presence and activity, the fungal concentration at the peak and off-peak periods of the two investigated canteens (3C and 5C) and two dormitories (CD and FD) did not show significant difference. Besides, no significant difference between the fungal concentration of outdoor (PS) and indoor environments was observed (Figure 3). A varying fungal concentration was observed across different seasons at each sampling site as shown in Figure 4. The concentrations of fungal colonies in each site generally alternate at different months, and higher fungal concentrations were overall recorded between summer and autumn (Figure 4 and Supplementary Table 1).

**TABLE 2:** Fungal colony concentration in each sampling site.

Sampling site	Colony concentration (CFU/m^3^)			
	Mean	*SD*	Min	Max
3CP	60.00	69.54	20	270
3CO	27.50	23.01	0	70
5CP	56.67	58.52	0	200
5CO	76.67	117.81	0	420
CDP	35.00	23.22	10	90
CDO	40.83	23.53	0	80
FDP	34.17	46.02	0	130
FDO	31.67	27.25	0	80
LIB	101.67	82.88	0	340
PS	70.00	108.71	0	360

*SD, standard deviation; Mean, the annual mean concentrations.*

### Influence of Environmental Parameters on Fungal Concentration

The Pearson correlation analysis reveals that the fungal concentration was positively correlated with temperature at the 3CO (y = 2.198x-16.729, *r* = 0.729, *P < 0.05), 5CP, 5CO, CDP, FDO, LIB, and PS, while the presence of airborne fungi at 3CP, CDO, and FDP was negatively correlated with temperature (Table 3). A positive correlation between fungal concentration and relative humidity was found at nearly all sampling sites, with the exception of FDP (Table 3). However, only at 3CO the positive correlation between temperature and fungal concentration was statistically significant (Table 3 and Supplementary Tables 2, 3).

**TABLE 3:** Pearson correlation analysis between fungal concentration and environmental factors from different sampling sites.

Factor	3CP	3CO	5CP	5CO	CDP	CDO	FDP	FDO	LIB	PS
Temperature	−0.019	0.729*	0.486	0.142	0.337	−0.059	−0.099	0.263	0.272	0.269
Relative humidity	0.192	0.497	0.276	0.279	0.129	0.121	−0.155	0.444	0.254	0.182

*Correlation significant at 0.05 level (2-tailed).

*3CP, Canteen 3 Peak; 3CO, Canteen 3 Off-peak; 5CP, Canteen 5 Peak; 5CO, Canteen 5 Off-peak; CDP, Chinese student Dorm Peak; CDO, Chinese student Dorm Off-peak; FDP, Foreign student Dorm Peak; FDO, Foreign student Dorm Off-peak; LIB, Library; PS, Peiyang Square.*

### Fungal Diversity and Distribution

**FIGURE 5:**
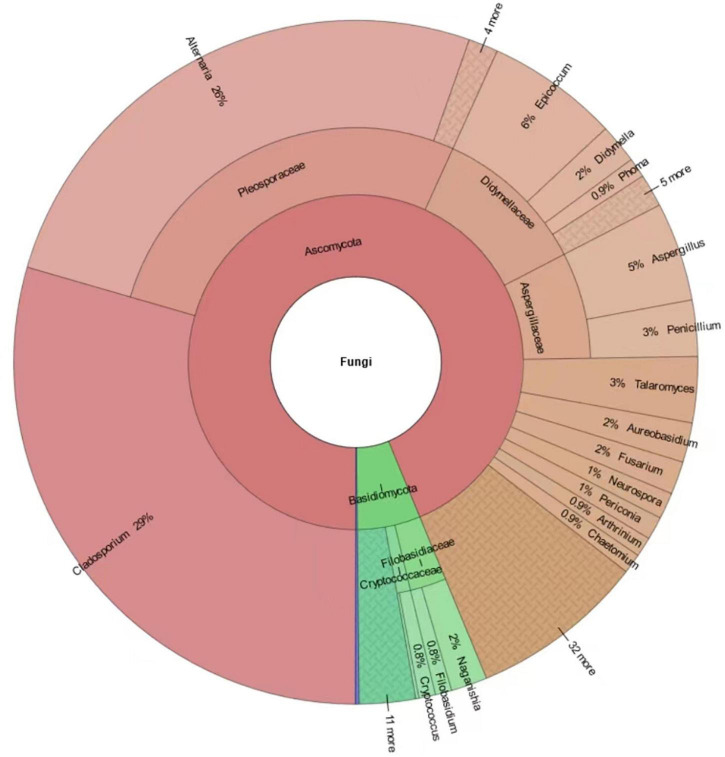
Krona chart indicating the taxonomic diversity and relative abundance of fungi isolated in this study. Round values are reported.

**FIGURE 6:**
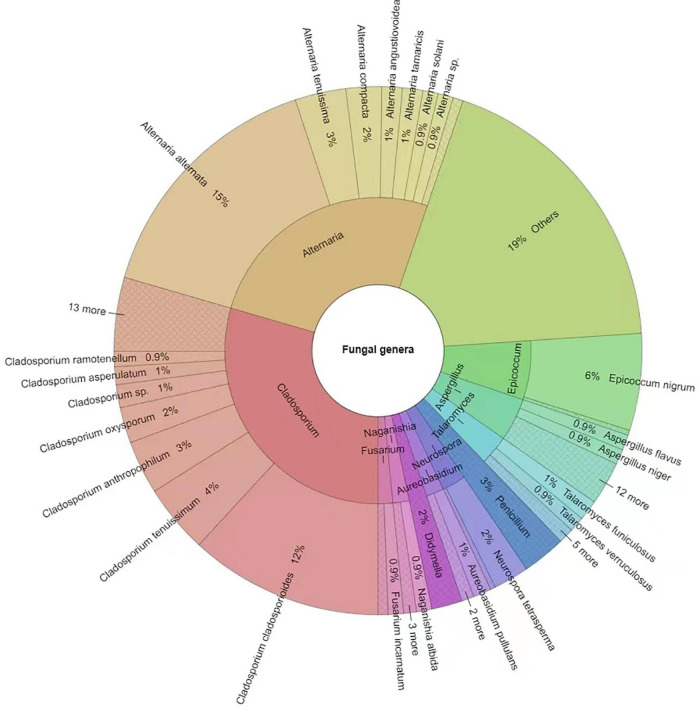
Krona chart indicating the dominant fungal genera and species isolated in this study. Others = strains < 1%. Round values are reported.

A total of 641 different fungal strains belonging to 173 species, 74 genera and 56 families were isolated from the 6 analyzed sites in Tianjin University campus during the whole study. Among the isolated fungi, the most represented phylum was Ascomycota (78.4%), followed by Basidiomycota (20.3%), and Mucoromycota (1.3%) (Figure 5 and Supplementary Table 4). The dominant fungal genus was *Cladosporium*, which accounted for 29.49**%** of the total taxonomic diversity, followed by *Alternaria* 25.9%, *Epicoccum* 6.24%, *Aspergillus* 4.68%, *Talaromyces* 3.12%, and *Penicillium* 2.65% (Figure 5 and Supplementary Table 5). Among the whole airborne fungal community, the genus *Cladosporium* showed the highest species richness, with 20 identified species representing 11.56% of total recorded *taxa*. The genus *Aspergillus* was the second most species-rich group, represented by 14 species (8.09% of total taxa) followed by *Alternaria* and *Penicillium*, both with 10 species (5.78%), while 7 (4.05%) and 5 species (2.89%) were found belonging to the genera *Talaromyces* and *Phoma*, respectively. The most frequently occurring fungal species were *Alternaria alternata* (15.44%), *Cladosporium cladosporioides* (11.86%), and *Epicoccum nigrum* (5.77%), followed by *C. tenuissimum* (4.21%), *C. anthropophilum* (3.28%), *A. tenuissima* (3.28%), *A. compacta* (2.18%), and *C. oxysporum* (2.18%) (Figure 6 and Supplementary Table 6).

**FIGURE 7:**
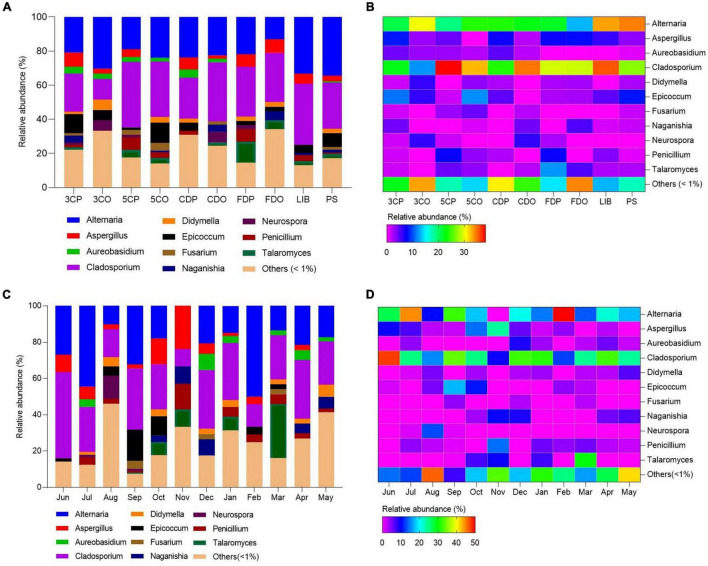
Fungal community structures (genera) with relative abundances in the sampling locations and months. **(A,C)** Represent the relative abundances of fungal genera in different locations and months. **(B,D)** Represent the heatmaps of fungal genera.

We analyzed the distribution, abundance, and community structures of different fungal genera across the sampling sites and seasons (Figure 7 and Supplementary Table 7). *Cladosporium* and *Alternaria* were the most widely distributed genera as they occurred at every site (Figures 7A,B). In particular, *Cladosporium* was more abundant at 5CP, CDO, and LIB as compared to *Alternaria*. The latter fungal genus was less spread across the locations, but more abundant at 3CO than *Cladosporium*. *Alternaria* occurred least at FDO while *Cladosporium* occurred least at 3CO. The genera *Aspergillus* and *Epicoccum* occurred in most sampling sites, both showing the highest abundance at PS, while they were much less present or absent at 5CO and CDO. *Talaromyces* fungi occurred at 3CP, 5CP, 5CO, CDO, FDP, FDO, LIB, and PS, with the highest abundance in FDP, while *Neurospora* species were abundant in 3CO and CDO. Concerning the airborne fungal diversity in different months, *Cladosporium* was also the most abundant and widely distributed genus across the sampling months, with significantly higher occurrence in June, September, December, January, and April, while the lowest presence of this genus was recorded in November (Figures 7C,D). The genus *Alternaria* showed the highest abundance in February and July, and the lowest in August. *Aspergillus* fungi were more abundant in the months of June, July, September, October, and November, while they were less represented in August, January, February, and April. *Penicillium* showed higher frequency in the air samples collected in July, November, January, and March as compared to August, February, and April. *Epicoccum* only occurred in six out of 12 analyzed months, showing the highest abundance in September, while *Talaromyces* was recorded in five different months, and most fungi in this genus were isolated in March.

### Effect of Human Activities on Airborne Fungal Diversity

**FIGURE 8:**
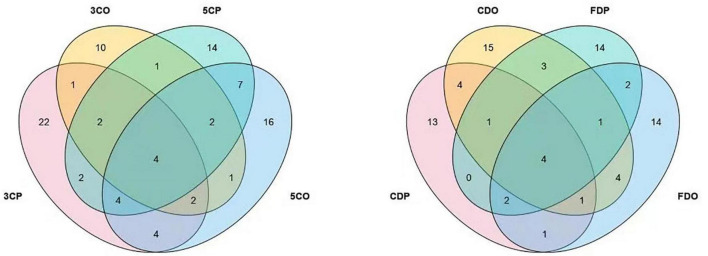
Venn diagram showing the comparative fungal species isolated from the peak and off-peak periods at different canteens (left) and dormitories (right).

**FIGURE 9:**
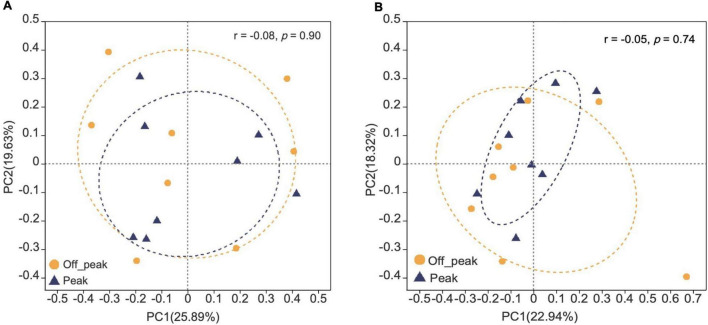
Principal co-ordinate analysis (PCoA) plots of fungal communities in peak and off-peak periods at canteens **(A)** and dormitories **(B)** based on Bray–Curtis distance. The r and *p*-values of analysis of similarity (ANOSIM) are shown in each figure.

The presence of fungal species at the canteens and dormitories was analyzed based on the peak and off-peak periods (Figure 8). For canteen 3, a considerable number of fungal species (22) was exclusively found at peak period (3CP) while 10 species were exclusive of the off-peak period (3CO). For canteen 5, a total of 14 and 16 fungal species were exclusively recorded at peak and off-peak periods, respectively. Four fungal species were found in common for both canteen 3 and 5 at peak (3CP, 5CP) and off-peak period (3CO, 5CO). For the Chinese student dormitory, there were 13 and 15 fungal species exclusively found at peak and off-peak periods, respectively, while for the foreign student dormitory, the number of exclusive species at peak and off-peak periods was the same (14). Four fungal taxa were found in common between the two dormitories at peak (CDP, FDP) and off-peak periods (CDO, FDO). The fungal diversity during the peak and off-peak periods at canteens and dormitories over the 1-year study was analyzed based on Shannon index (Table 4). In general, 3CP showed the highest Shannon index value (3.358) while the 3CO showed the lowest (2.959). The Shannon index values of the two canteens in peak period (3CP and 5CP) were higher than in the off-peak period (3CO and 5CO). On the contrary, the two dormitories showed higher Shannon index values in off-peak (CDO and FDO) than in peak period (CDP and FDP). At genus level, principal coordinate analysis revealed homogeneity of fungal diversity between peak and off-peak periods, in both canteen and dormitory environments (Figure 9).

**TABLE 4:** Shannon index in different sampling sites over the 1 year.

Sampling sites	Shannon index	Evenness
3CP	3.358	0.904
3CO	2.959	0.944
5CP	3.25	0.9
5CO	3.101	0.841
CDP	3.032	0.931
CDO	3.275	0.937
FDP	3.093	0.938
FDO	3.245	0.964

### Effect of Seasonal Variations and Environmental Parameters on the Fungal Diversity

**FIGURE 10:**
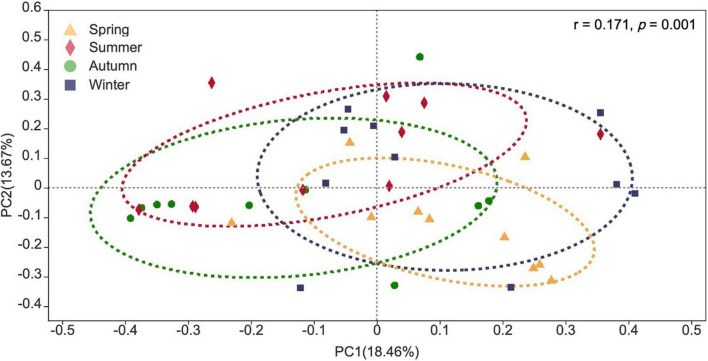
Principal co-ordinate analysis (PCoA) plot of fungal communities detected in four seasons.

Fungal communities detected from different seasons in the campus were clearly distinguished based on Bray–Curtis distance (ANOSIM *p* = 0.001) (Figure 10) at genus level. However, temperature and relative humidity were not the main drivers for the whole campus fungal community structure as revealed by PERMANOVA (Supplementary Table 8).

**FIGURE 11:**
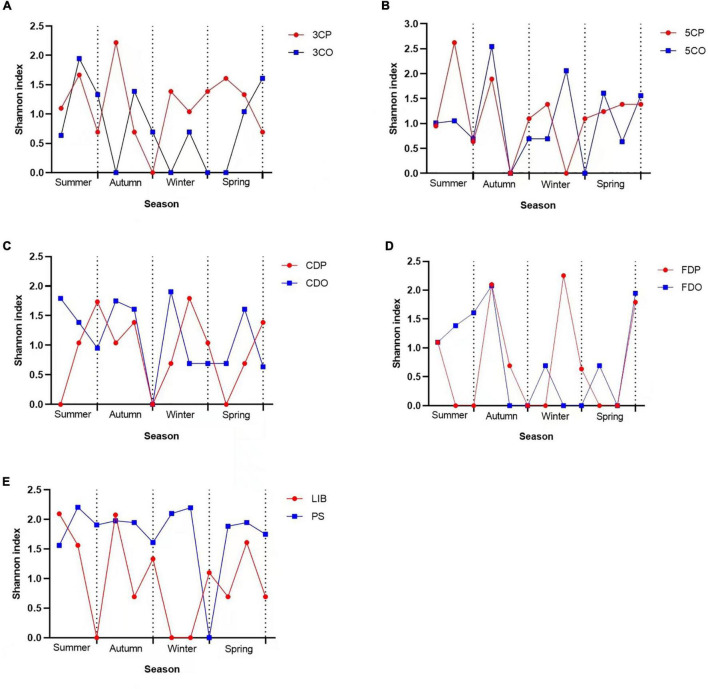
Shannon index variations across seasons at different sampling sites. **(A)** Canteen 3, **(B)** Canteen 5, **(C)** Chinese student Dorm, **(D)** Foreign student Dorm, **(E)** Library and Peiyang Square.

The changes in fungal diversity based on the Shannon index due to seasonal variations are shown in Figure 11. The highest diversity index values in canteen 3 were recorded at the peak period during autumn, winter, and spring while the off-peak period showed the highest value in summer (Figure 11A). Canteen 5 showed its highest fungal diversity index during summer at peak period, while in autumn, winter, and spring the highest index was measured during the off-peak period (Figure 11B). The student dormitories (CDO/CDP and FDO/FDP) showed a remarkably alternating fungal diversity in the four studied seasons (Figures 11C,D), while the PS had higher fungal diversity index than the LIB throughout nearly the 4 seasons of the year (Figure 11E).

We also analyzed the influence of temperature and relative humidity on the fungal community in the sampling sites using Pearson correlation analysis. The results revealed that the fungal diversity based on Shannon index was positively related to temperature only at the 3CO (y = 0.060x-0.423, *r* = 0.659, *P < 0.05), while no significant correlation was found in all other sites (Table 5). The relative humidity showed positive relationship with fungal diversity in the 3CP, 3CO, 5CO, CDO, FDO, LIB, and PS, while was negatively related to diversity of fungi recorded in the 5CP, CDP, and FDP, although the correlation was not significant.

**TABLE 5:** Pearson correlation analysis between Shannon index and environmental factors from different sampling sites.

Factor	3CP	3CO	5CP	5CO	CDP	CDO	FDP	FDO	LIB	PS
Temperature	−0.158	0.659*	0.266	0.362	0.133	−0.082	−0.009	0.244	−0.016	0.178
Relative humidity	0.317	0.387	−0.086	0.3	−0.062	0.572	−0.223	0.449	0.464	0.225

*Correlation significant at 0.05 level (2-tailed).

*3CP, Canteen 3 Peak; 3CO, Canteen 3 Off-peak; 5CP, Canteen 5 Peak; 5CO, Canteen 5 Off-peak; CDP, Chinese student Dorm Peak; CDO, Chinese student Dorm Off-peak; FDP, Foreign student Dorm Peak; FDO, Foreign student Dorm Off-peak; LIB, Library; PS, Peiyang Square.*

**FIGURE 12:**
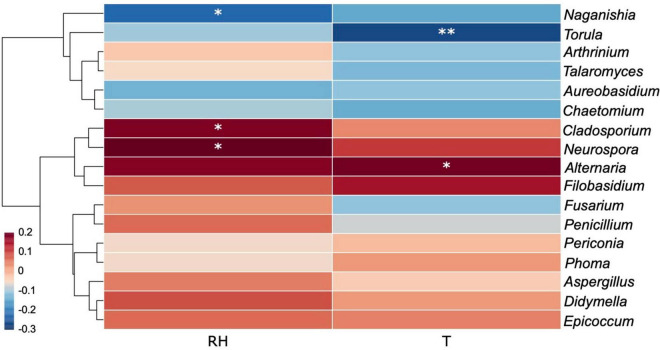
Correlation heat map of the dominant fungal genera (with abundance > 5) and environmental factors. Different colors infer to Spearman’s rank correlation coefficients (*r*). RH and T refer to “relative humidity” and “temperature.” *p* is indicated as: *p < 0.05, ^**^p < 0.01.

The effects of recorded environmental factors on specific fungal genera were evaluated by a correlation heat map (Figure 12). Generally, a large proportion of dominant fungal genera were positively correlated with temperature and relative humidity as revealed by the massive area of red color in the heat map, which represented the correlation coefficients (r) over 0, with several genera showing significant positive correlations, including *Alternaria* (r_T_ = 0.1936, *p*_T_ = 0.0341), *Cladosporium* (r_RH_ = 0.1800, *p*_RH_ = 0.0491), and *Neurospora* (r_RH_ = 0.2193, *p*_RH_ = 0.0161). On the contrary, *Naganishia* (r_RH_ = –0.2303, *p*_RH_ = 0.0114) and *Torula* (r_T_ = –0.2691, *p*_T_ = 0.0030) were found to be significantly negatively correlated with relative humidity and temperature, respectively.

## Discussion

### The Influence of Environmental Factors on Airborne Fungal Distribution and Concentration

This is the first study on culturable airborne fungal diversity in indoor and outdoor environments at Tianjin University campus, Tianjin, China. The dynamism of the fungal diversity and concentration in the six sampling sites was correlated with seasonal variations and environmental conditions (temperature and relative humidity). The overall fungal concentration ranged from 0 to 420 CFU/m^3^ across all the sampling sites. The Ministry of Health (MOH), Ministry of Environmental Protection (MEP), and the General Administration of Quality Supervision, Inspection and Quarantine (AQSIQ) of China, have established a threshold value of 2,500 CFU/m^3^ for total airborne microorganism in Chinese residential and office buildings in the Indoor Air Quality Standard issued in 2002 (GB/T 18883, 2002). None of our indoor environments exceeded the above-mentioned recommended concentration standards, and they were also within the limits recommended by the American Industrial Hygiene Association (AIHA) in 2001, which placed the threshold for fungal spore concentration in indoor environments of residential building below 500 CFU/m^3^ (Wonder Makers Environmental, 2001). Among the investigated sites, LIB showed the highest annual mean fungal concentration, with 101.67 CFU/m^3^, followed by 5CO (76.67 CFU/m^3^), PS (70.00 CFU/m^3^), 3CP (60.00 CFU/m^3^), and 5CP (56.67 CFU/m^3^) while the lowest annual mean fungal concentration was recorded in 3CO, which was 27.50 CFU/m^3^. In a study previously carried out in Chang’ an University (China), the mean fungal concentrations were higher than those registered in Tianjin University campus, being 699 ± 57 CFU/m^3^ in the canteen and 479 ± 66 CFU/m^3^ in the dormitories (Li et al., 2015). Similarly, Lou et al. (2012) reported higher fungal concentration in 4 areas in Zhejiang Gongshang University campus, Hangzhou (China) including the dining, living, teaching, and office areas. The fungal concentrations in the two canteens (3C, 5C) and two dormitories (CD, FD) analyzed in our work did not show significant difference at peak and off-peak periods. This observation is not in agreement with previous studies in which the concentration of airborne fungi was found to increase in the presence of more intense human traffic (Shintani et al., 2004; Chen et al., 2010). However, our results indicated that the annual mean fungal concentration outdoor (PS) was closely similar to that in the indoor environment at peak period (3CP), whereas it was higher than the indoor annual mean concentration during the off-peak period (3CO). Since the canteen 3 (3C) is located at close distance from the analyzed outdoor area of Peiyang Square (PS) (Figure 1), the similar level of fungal concentration in these two environments during the peak of human activity in 3C could be related to the maximum influx of people, which may represent a vehicle for the diffusion of outdoor fungal particles into the indoor environment of the canteen. This hypothesis is in agreement with the study by Chaivisit et al. (2018) where it was found that the concentration of fungi in indoor environments was supported by the presence of fungal spores outdoor.

A seasonal effect on fungal concentration in both indoor and outdoor environments was found in this study. The concentrations of fungal colonies generally alternate with the seasonal variations in each site, with the highest fungal concentrations recorded in autumn, specifically in September, and the least recorded in winter, for both indoor and outdoor sampling sites. Similar observations were reported in different indoor environments at Chang’ an university (China), where higher levels of airborne fungal concentration were found in autumn (Li et al., 2015). In another study, Li et al. (2010) reported the highest concentration of fungal colonies in Shenzhen University campus (China) in September. We observed that the environmental factors had different influences on the fungal concentration in sampling locations and periods (peak and off-peak). The fungal concentration was positively correlated with temperature, at most of the investigated sites except 3CP, CDO, and FDP, and with relative humidity at all sites and sampling periods, with the sole exception of FDP. Rajasekar and Balasubramanian (2011) observed that the indoor fungal concentration in food courts at National university of Singapore (NUS) campus was positively correlated to relative humidity, whereas outdoor fungal concentration was both positively correlated to relative humidity and negatively correlated to temperature. In another study, Li et al. (2015) reported that relative humidity was positively correlated with airborne fungal concentration in canteen and dormitories (*p* < 0.05) of Chang’ an University, China.

### Fungal Diversity and Potential Effects on Air Quality and Human Health

Among the total 641 fungal strains recorded in this study, 84 strains (35 species and 17 genera) were isolated from the outdoor environment of Peiyang Square (PS), which showed a higher diversity of airborne fungi as compared to the outdoor environments previously investigated by Nageen et al. (2021), in the same city district (Nankai), outside Tianjin University. In fact, in the latter study, the total number of isolated fungal strains was 51, belonging to 20 species and 11 genera, and 25 (12 species, 6 genera), for a site characterized by intense car traffic (Nankai P) and a site located in a green area (Nankai G), respectively (Nageen et al., 2021). Compared to the latter two sites, the outdoor environment at Tianjin university campus (PS) is characterized by hybrid environmental conditions, being constituted by a green area with car traffic, which may increase the diversity of fungi. The most dominant fungal genera observed in our study were *Cladosporium* (29.49%), *Alternaria* (25.9%), *Epicoccum* (6.24%), and *Aspergillus* (4.68%). The *Cladosporium* and *Alternaria* genera were also the most dominant genera previously observed in Tianjin at city level, where they accounted for 18.4 and 22% of the total fungal genera, respectively (Nageen et al., 2021). These taxa are not a peculiarity of Tianjin airborne fungal community since they have been reported as common airborne fungi in different environments across the world (Pei-Chih et al., 2000; Fang et al., 2005; Liu et al., 2014; Lee et al., 2015; Wu et al., 2019). Some other dominant fungal genera from our study, such as *Penicillium* and *Fusarium* have been reported earlier, in different countries. For instance, Hargreaves et al. (2003) reported that the most frequently isolated fungal genera from 14 residential sub-urban houses in Brisbane, Australia were *Cladosporium*, *Curvularia*, *Alternaria*, *Fusarium*, and *Penicillium*. Abdel Hameed et al. (2009) reported *Aspergillus*, *Penicillium*, *Alternaria*, and *Cladosporium* as the most dominant airborne fungal genera in the industrial town of Helwan in Egypt. Many fungal species from *Cladosporium*, *Alternaria*, and *Aspergillus* genera have been reported among the most common allergenic microorganisms. For example, elevated concentrations of *Cladosporium* were found to be commonly associated with respiratory symptoms in Topeka, Kansas, United States (Su et al., 1992). The most frequently occurring fungal species in our study were *A. alternata*, *C. cladosporioides*, *Epicoccum nigrum*, *C. tenuissimum*, *C. anthropophilum*, and *A. tenuissima*. Among them, *A. alternata*, *C. cladosporioides*, *A. tenuissima* have been previously reported as the dominant airborne fungi from outdoor environments in Tianjin city, while *E. nigrum*, which was the third most abundant fungus in our study, constituted only 1.43% of the total fungi isolated in Tianjin city by Nageen et al. (2021). In the latter study, *Naganishia albida* was also included in the list of the dominant fungal species recorded in the analyzed environments (Nageen et al., 2021), whereas this taxon, which has been reported as a potential pathogenic species (Aghaei Gharehbolagh et al., 2017), constituted only 0.94% of the total fungi isolated in Tianjin University (Supplementary Table 6). Most of the fungi isolated in our study are common fungal components of bioaerosol and have been previously isolated from different cities in China. For instance, in the study of Fang et al. (2019), the most prevalent fungal species in Hangzhou were *P. chrysogenum*, *C. cladosporioides* and *A. alternata*. The *Alternaria* species, which were the most dominant fungi in our study, have been associated with common allergies and increase in the respiration immunoglobulin E (IgE) diseases, specifically inducing an asthma exacerbation (Downs et al., 2001). In particular, *A. alternata* has been associated with 16 different allergens according to previous studies (Kustrzeba-Wójcicka et al., 2014; Gabriel et al., 2016). *Cladosporium* species, usually occurring as saprobic fungi, have also been well reported as opportunistic pathogenic organisms associated with many human and animal infections (Larsen, 1981; Mercier et al., 2013; Sandoval-Denis et al., 2015), and have been identified in various clinical samples from hospital patients having different symptoms (Sellart-Altisent et al., 2007). *Cladosporium cladosporioides* was identified as the causative agent of Phaeohyphomycotic dermatitis in a giant panda (Ma et al., 2013). *Aspergillus* species are common mycotoxin producing organisms, and they have been recently reported as the most common fungi increasing specific Immunoglobulin E (sIgE) titer. Besides, *Aspergillus* fungi have been correlated with increased allergic rhinitis risk and allergic rhinitis-related symptoms (Sio et al., 2021). Although the concentration of fungal species in Tianjin University air environments was far below the recommended limits (GB/T 18883, 2002), the presence of the above-mentioned fungi with potential hazardous effect on human health deserves attention from a pathological point of view and should be monitored in future studies. Appropriate management practices may contribute to enhance the air quality in the studied indoor environments by preventing microbial contamination from outdoor. For instance, the indoor presence of *Alternaria* species, which are mostly outdoor fungi commonly colonizing plant tissues (Rotem, 1994), could be controlled by reducing the natural ventilation, since the multicellular large-sized conidia of this fungal genus are likely to be trapped by air filters in the mechanical ventilation and air-conditioning system (Mui et al., 2010). Moreover, the penetration in indoor environments of fungi that are present outdoor could be partially removed by limiting the number of people that frequent indoor areas, such as canteens, at a certain time. For other fungal taxa that are primarily associated to indoor environments, such as *Aspergillus* fungi, their dominant presence could be prevented by avoiding the establishment of environmental conditions that sustain indoor fungal growth, including increased humidity, temperature and CO_2_, which can be avoided with adequate ventilation (Burge, 2000; Chao et al., 2002).

### Human Activity, Abiotic Factors, and Seasonal Variation Affect the Airborne Fungal Diversity and Community Dynamics

The abundance and community structure of airborne fungi observed at the campus of Tianjin University during this study varied with location and season. The fungal community structure was influenced by the seasonal variation and human traffic (peak and off-peak). Interestingly, the fungal diversity (based on Shannon index) at the two canteens analyzed in this study was higher during the peak period than in the off-peak period. On the contrary, the fungal community at two dormitories were more diverse during off-peak period than the peak period. In order to explain these contradictory results, it is important to note that the two canteens were opened to all the staff and students, while the access to the two dormitories was restricted to a limited number of authorized students. The presence/continual influx of different visitors at the canteens, may represent a vehicle for the successful invasion of fungi carried from different environments, thus contributing to the higher fungal diversity during the peak period. High human traffic has been reported to influence the interior microclimate of an indoor environment and the resuspension of microorganisms present on the floor (Hoyos et al., 1998; Chen et al., 2010). For the dormitories, we may hypothesize that the air exchange could be the factor supporting the higher diversity of airborne fungi during the off-peak period, when we observed that the windows were left open to allow ventilation. Further studies, based on the measurement of additional environmental parameters, such as wind speed, would be required to test this hypothesis.

Temperature and humidity were also found to affect the diversity of airborne fungi in the different investigated environments. Higher fungal diversity was observed during summer for most of the analyzed locations, which is in agreement with a study carried out in Beijing, where it was shown that air temperature and humidity in summer and autumn were more appropriate for fungal propagation as compared to winter season (Fang et al., 2005). In addition, our results also showed that the fungal diversity was positively correlated to the temperature at the 3CO (*P < 0.05). This observation is consistent with previous studies that described temperature and humidity playing important roles in the airborne fungal distribution and diversity (Hoyos et al., 1998; Fang et al., 2005; Lu et al., 2022). In summary, the analyses performed in our work revealed a remarkable fluctuation in the fungal diversity of every sampling site at different seasons. The environmental conditions, such as temperature and humidity, are an important factor that can affect the airborne fungi of both indoor and outdoor environments. Besides, a crucial role in affecting the concentration of airborne fungi in indoor environment can be attributed to human traffic. The seasonal changes can also significantly influence the distribution and diversity of the airborne fungi in both indoor and outdoor environments.

## Conclusion

In this study, we provided the first detailed report on the diversity, concentration, and community structure of airborne fungi in Tianjin University campus over a period of 1 year. We correlated the dynamics of the investigated fungal communities with abiotic environmental factors (temperature and relative humidity) and human traffic. Our results showed that the human traffic was a key factor shaping the airborne fungal diversity in the studied sites, while temperature and humidity were mostly correlated with the fungal concentration.

Overall, Tianjin University campus air environments showed a rich fungal diversity, while the total concentration of airborne fungi was never above the tolerance limit throughout the study. *Cladosporium* and *Alternaria* were the dominant genera, while *A. alternata, C. cladosporioides, C. tenuissimum*, *C. anthropophilum*, *A. tenuissima*, *A. compacta*, and *C. oxysporum* were the most abundant species among the analyzed culturable airborne fungi. Although, these fungi have commonly been reported from a variety of environments, their presence in the air deserves attention from a pathological point of view due to their potential health hazards. The ventilation system (natural vs. mechanical) could be a crucial factor determining the fungal diversity and concentration in the analyzed indoor areas, which could be protected from microbial contamination generated by outdoor sources, with the use of air filters and the accurate maintenance and fine-tuned operating conditions of mechanical ventilation and air-conditioning system. A limited presence of occupants could also enhance the indoor air quality by limiting the intrusion of outdoor microbial contaminants. The results of this study provide valuable information on the environmental factors shaping the indoor and outdoor fungal communities in a densely populated university area, which can be used in air quality and pollution control management, and for prevention of airborne diseases in environments characterized by high human activity.

## Data Availability Statement

The datasets presented in this study can be found in online repositories. The names of the repository/repositories and accession number(s) can be found below: https://www.ncbi.nlm.nih.gov/genbank/, OM236671–OM237311.

## Author Contributions

LP conceived the study. CY and XW collected the samples. LP designed and supervised the experiments. CY performed the laboratory experiments and analysis. CY, XW, and LP analyzed the results. CY prepared the original draft under the guidance and critical review of LP while XW, and also contributed to write and review the manuscript. All authors have read and agreed to the published version of the manuscript.

## Conflict of Interest

The authors declare that the research was conducted in the absence of any commercial or financial relationships that could be construed as a potential conflict of interest.

## Publisher’s Note

All claims expressed in this article are solely those of the authors and do not necessarily represent those of their affiliated organizations, or those of the publisher, the editors and the reviewers. Any product that may be evaluated in this article, or claim that may be made by its manufacturer, is not guaranteed or endorsed by the publisher.

## Footnotes

1
http://www.ncbi.nlm.nih.gov/BLAST/index.html

2
https://github.com/marbl/Krona/wiki/KronaTools
