# Supplementary material for: Environmental Factors Shaping the Diversity and Spatial-Temporal Distribution of Indoor and Outdoor Culturable Airborne Fungal Communities in Tianjin University Campus, Tianjin, China
Source: Front Microbiol. 2022 Jun 14;13:928921. doi: 10.3389/fmicb.2022.928921 (PMC9237511; doi:10.3389/fmicb.2022.928921)
Supplement: Supplementary file 1 [file Data_Sheet_1.docx]

**Supplementary information**

**Environmental factors shaping the diversity and spatial-temporal distribution of indoor and outdoor culturable airborne fungal communities in Tianjin University campus, Tianjin, China**

Chaonan Yuan^1^, Xiao Wang^1^ and Lorenzo Pecoraro^1^*

^1^School of Pharmaceutical Science and Technology, Tianjin University, 92 Weijin Road, Nankai District, 300072 Tianjin, China; chaonan@tju.edu.cn (C.Y.), wang_xiao1996@163.com (X.W.); lorenzo.pecoraro@tju.edu.cn (L.P.).

*Correspondence: Lorenzo Pecoraro (telephone: +86 18520824550, e-mail: lorenzo.pecoraro@tju.edu.cn) School of Pharmaceutical Science and Technology, Tianjin University, 92 Weijin Road, Nankai District, Tianjin 300072, China.

**Table S1.** Concentration of fungal colonies from each location in each month.

|  | | Colony concentration (CFU/m^3^) | | | | | | | | | |
| --- | --- | --- | --- | --- | --- | --- | --- | --- | --- | --- | --- |
|  | 3CP | | 3CO | 5CP | 5CO | CDP | CDO | FDP | FDO | LIB | PS |
| Jun | 30 | | 30 | 50 | 60 | 10 | 60 | 30 | 60 | 80 | 220 |
| Jul | 80 | | 70 | 200 | 50 | 40 | 40 | 10 | 40 | 130 | 60 |
| Aug | 20 | | 50 | 30 | 20 | 90 | 50 | 0 | 50 | 80 | 0 |
| Sep | 270 | | 0 | 150 | 420 | 40 | 70 | 130 | 80 | 340 | 360 |
| Oct | 20 | | 40 | 10 | 0 | 40 | 50 | 20 | 10 | 70 | 20 |
| Nov | 20 | | 20 | 30 | 20 | 10 | 0 | 0 | 10 | 50 | 50 |
| Dec | 40 | | 0 | 40 | 20 | 20 | 80 | 0 | 20 | 110 | 10 |
| Jan | 40 | | 20 | 0 | 180 | 60 | 20 | 120 | 10 | 90 | 0 |
| Feb | 80 | | 0 | 30 | 10 | 40 | 20 | 30 | 0 | 0 | 30 |
| Mar | 50 | | 10 | 60 | 50 | 10 | 20 | 0 | 20 | 130 | 20 |
| Apr | 50 | | 40 | 40 | 30 | 20 | 50 | 10 | 10 | 70 | 50 |
| May | 20 | | 50 | 40 | 60 | 40 | 30 | 60 | 70 | 70 | 20 |

**Table S2.** Temperature (^o^C) recorded during each sampling.

| Location | Temperature/Month (^o^C) | | | | | | | | | | | |
| --- | --- | --- | --- | --- | --- | --- | --- | --- | --- | --- | --- | --- |
|  | Jun | Jul | Aug | Sep | Oct | Nov | Dec | Jan | Feb | Mar | Apr | May |
| 3CP | 25.8 | 30.6 | 28 | 21 | 20.3 | 19.1 | 7.7 | 16.5 | 13.2 | 15.8 | 15.6 | 27.9 |
| 3CO | 28.6 | 31.4 | 23.8 | 20.2 | 21.5 | 14.3 | 5.0 | 17.8 | 12.1 | 21.9 | 16.1 | 28.8 |
| 5CP | 27.6 | 29.7 | 21.8 | 20.8 | 21.4 | 12.2 | 9.8 | 15.5 | 7.3 | 21.5 | 15.6 | 26.8 |
| 5CO | 27.2 | 29 | 22.4 | 20.3 | 21.4 | 11.5 | 7.7 | 17.7 | 7.0 | 21.5 | 15.8 | 26.8 |
| CDP | 26.5 | 26.3 | 26.7 | 21.2 | 21 | 19.3 | 15.4 | 17.4 | 24.1 | 20.2 | 18.2 | 24.8 |
| CDO | 26.8 | 27.4 | 27.8 | 19.8 | 21.4 | 20.3 | 9.5 | 17.7 | 10.5 | 20.3 | 16 | 27.2 |
| FDP | 26.3 | 23.7 | 24.2 | 19.1 | 22.4 | 21.2 | 21.3 | 20.9 | 5.1 | 21 | 20.4 | 24 |
| FDO | 26.7 | 25.7 | 23.7 | 19.2 | 22.8 | 23.6 | 24.8 | 28.6 | 14.7 | 23.3 | 15.3 | 25.9 |
| LIB | 26.8 | 26.2 | 22.7 | 19.5 | 21.3 | 13.6 | 19.7 | 20.2 | 5.7 | 19.8 | 15.7 | 26.8 |
| PS | 30.4 | 35.8 | 24.7 | 19.6 | 19.5 | 6.6 | 4.7 | 7.3 | 11.0 | 18.8 | 15.4 | 27.4 |

3CP = Canteen 3 Peak, 3CO = Canteen 3 Off-peak, 5CP = Canteen 5 Peak, 5CO = Canteen 5 Off-peak, CDP = Chinese students Dorm Peak, CDO = Chinese students Dorm Off-peak, FDP = Foreign students Dorm Peak, FDO = Foreign students Dorm Off-peak, LIB = Library, PS = Peiyang Square.

**Table S3.** Relative Humidity (%) recorded during each sampling.

| Location | Relative Humidity/Month (%) | | | | | | | | | | | |
| --- | --- | --- | --- | --- | --- | --- | --- | --- | --- | --- | --- | --- |
|  | Jun | Jul | Aug | Sep | Oct | Nov | Dec | Jan | Feb | Mar | Apr | May |
| 3CP | 57.5 | 60.1 | 62.5 | 52.2 | 52.6 | 20.3 | 21.5 | 32.7 | 26.4 | 44.3 | 54.8 | 28.8 |
| 3CO | 54.9 | 55.3 | 73.9 | 48.9 | 41.5 | 22.8 | 7.1 | 58.5 | 27.6 | 47.1 | 58.2 | 30.6 |
| 5CP | 52.3 | 58.3 | 61.6 | 54.5 | 50.2 | 30.1 | 20.8 | 58.5 | 33.0 | 44.1 | 57.0 | 31.5 |
| 5CO | 52.6 | 59.2 | 61.8 | 51.8 | 45.8 | 30.5 | 16.5 | 58.5 | 22.5 | 43.6 | 60.2 | 29.3 |
| CDP | 70.3 | 87.1 | 82.3 | 70.2 | 45.5 | 37.7 | 11.7 | 33.4 | 29.5 | 73.2 | 83.5 | 52.2 |
| CDO | 64.6 | 70.0 | 72.7 | 61.4 | 43.4 | 23.9 | 37.3 | 29.7 | 30.2 | 50.7 | 64.1 | 30.2 |
| FDP | 71.8 | 84.5 | 77.7 | 61.4 | 40.5 | 31.1 | 9.2 | 29.7 | 30.0 | 71.6 | 77.5 | 39.0 |
| FDO | 65.1 | 75.3 | 78.6 | 56.8 | 49.3 | 13.9 | 7.7 | 14.8 | 29.5 | 42.1 | 55.1 | 25.7 |
| LIB | 62.9 | 63.4 | 65.2 | 51.2 | 42.7 | 29.7 | 10.5 | 23.6 | 29.6 | 57.7 | 51.5 | 29.7 |
| PS | 53.4 | 46.4 | 70.1 | 46.5 | 36.0 | 27.7 | 15.4 | 44.8 | 30.1 | 56.6 | 54.7 | 21.8 |

3CP = Canteen 3 Peak, 3CO = Canteen 3 Off-peak, 5CP = Canteen 5 Peak, 5CO = Canteen 5 Off-peak, CDP = Chinese students Dorm Peak, CDO = Chinese students Dorm Off-peak, FDP = Foreign students Dorm Peak, FDO = Foreign students Dorm Off-peak, LIB = Library, PS = Peiyang Square.

**Table S4.** Airborne fungal diversity molecularly detected in indoor and outdoor environments of Tianjin University, from DNA extracted from isolated strains.

| **Sample**  **No.** | **Sampling**  **Month** | **Site**  **code** | **GenBank**  **code** | **Best BLAST match(es)** | **Accession**  **code** | **Overlap**  **length** | **% match** |
| --- | --- | --- | --- | --- | --- | --- | --- |
| 1 | Jun | LIB | OM236671 | *Cladosporium tenuissimum* | [MK370679.1](https://www.ncbi.nlm.nih.gov/nucleotide/MK370679.1?report=genbank&log$=nucltop&blast_rank=1&RID=7V35VZEF016) | 937 | 99.61% |
| 2 | Jun | LIB | OM236672 | *Cladosporium oxysporum* | [MF135506.1](https://www.ncbi.nlm.nih.gov/nucleotide/MF135506.1?report=genbank&log$=nucltop&blast_rank=1&RID=7V3J5BNM01R) | 942 | 99.81% |
| 3 | Jun | LIB | OM236673 | *Alternaria tenuissima* | [MW008914.1](https://www.ncbi.nlm.nih.gov/nucleotide/MW008914.1?report=genbank&log$=nucltop&blast_rank=1&RID=7V3PWSYN013) | 977 | 99.81% |
| 4 | Jun | LIB | OM236674 | *Cladosporium tenuissimum* | [MT072081.1](https://www.ncbi.nlm.nih.gov/nucleotide/MT072081.1?report=genbank&log$=nucltop&blast_rank=1&RID=7V3WEC4201R) | 941 | 99.05% |
| 5 | Jun | LIB | OM236675 | *Aspergillus oryzae* | [MT306006.1](https://www.ncbi.nlm.nih.gov/nucleotide/MT306006.1?report=genbank&log$=nucltop&blast_rank=1&RID=7V41KWX5016) | 1009 | 99.46% |
| 6 | Jun | LIB | OM236676 | Fungal sp. | [KX098100.1](https://www.ncbi.nlm.nih.gov/nucleotide/KX098100.1?report=genbank&log$=nucltop&blast_rank=1&RID=7V45X6VU016) | 961 | 99.43% |
|  |  |  |  | *Aspergillus versicolor* | [MN788648.1](https://www.ncbi.nlm.nih.gov/nucleotide/MN788648.1?report=genbank&log$=nucltop&blast_rank=3&RID=7V45X6VU016) | 955 | 99.25% |
| 7 | Jun | LIB | OM236677 | *Cladosporium* sp. | [KM979743.1](https://www.ncbi.nlm.nih.gov/nucleotide/KM979743.1?report=genbank&log$=nucltop&blast_rank=1&RID=7V4C655U01R) | 928 | 99.22% |
|  |  |  |  | *Cladosporium uredinicola* | [KX082931.1](https://www.ncbi.nlm.nih.gov/nucleotide/KX082931.1?report=genbank&log$=nucltop&blast_rank=2&RID=7V4C655U01R) | 928 | 99.22% |
| 8 | Jun | LIB | OM236678 | *Cladosporium* sp. | [KM979743.1](https://www.ncbi.nlm.nih.gov/nucleotide/KM979743.1?report=genbank&log$=nucltop&blast_rank=1&RID=7V4HAJM201R) | 928 | 99.22% |
|  |  |  |  | *Cladosporium uredinicola* | [KX082931.1](https://www.ncbi.nlm.nih.gov/nucleotide/KX082931.1?report=genbank&log$=nucltop&blast_rank=2&RID=7V4HAJM201R) | 928 | 99.22% |
| 9 | Jun | LIB | OM236679 | *Cladosporium tenuissimum* | [MK370679.1](https://www.ncbi.nlm.nih.gov/nucleotide/MK370679.1?report=genbank&log$=nucltop&blast_rank=1&RID=7V4N7NG0013) | 942 | 99.81% |
| 10 | Jun | LIB | OM236680 | *Cladosporium tenuissimum* | [MT072081.1](https://www.ncbi.nlm.nih.gov/nucleotide/MT072081.1?report=genbank&log$=nucltop&blast_rank=1&RID=7V4SUA0501R) | 933 | 99.23% |
| 11 | Jun | LIB | OM236681 | Fungal sp. | [MH252208.1](https://www.ncbi.nlm.nih.gov/nucleotide/MH252208.1?report=genbank&log$=nucltop&blast_rank=1&RID=7V4ZCJ4H013) | 926 | 99.22% |
|  |  |  |  | *Cladosporium oxysporum* | [KY400086.1](https://www.ncbi.nlm.nih.gov/nucleotide/KY400086.1?report=genbank&log$=nucltop&blast_rank=2&RID=7V4ZCJ4H013) | 926 | 99.22% |
| 12 | Jun | LIB | OM236682 | *Mucor circinelloides* | [MN626446.1](https://www.ncbi.nlm.nih.gov/nucleotide/MN626446.1?report=genbank&log$=nucltop&blast_rank=1&RID=7V54JCVS016) | 1094 | 99.50% |
| 13 | Jun | LIB | OM236683 | *Alternaria alternata* | [MT090004.1](https://www.ncbi.nlm.nih.gov/nucleotide/MT090004.1?report=genbank&log$=nucltop&blast_rank=1&RID=7V57AZ6901R) | 985 | 99.45% |
| 14 | Jun | LIB | OM236684 | *Aspergillus versicolor* | [KM613141.1](https://www.ncbi.nlm.nih.gov/nucleotide/KM613141.1?report=genbank&log$=nucltop&blast_rank=1&RID=7V5BZA7P013) | 979 | 99.26% |
| 15 | Jun | LIB | OM236685 | *Dothideomycetes* sp. | [KF907245.1](https://www.ncbi.nlm.nih.gov/nucleotide/KF907245.1?report=genbank&log$=nucltop&blast_rank=1&RID=7V5FCSFZ013) | 928 | 99.61% |
|  |  |  |  | *Cladosporium cladosporioides* | [MT573533.1](https://www.ncbi.nlm.nih.gov/nucleotide/MT573533.1?report=genbank&log$=nucltop&blast_rank=2&RID=7V5FCSFZ013) | 926 | 99.22% |
| 16 | Jun | LIB | OM236686 | *Cladosporium* sp. | [MT729897.1](https://www.ncbi.nlm.nih.gov/nucleotide/MT729897.1?report=genbank&log$=nucltop&blast_rank=1&RID=7V5K7PD0013) | 939 | 99.61% |
|  |  |  |  | *Cladosporium cladosporioides* | [MT729833.1](https://www.ncbi.nlm.nih.gov/nucleotide/MT729833.1?report=genbank&log$=nucltop&blast_rank=2&RID=7V5K7PD0013) | 939 | 99.80% |
| 17 | Jun | LIB | OM236687 | *Cladosporium cladosporioides* | [MF061760.1](https://www.ncbi.nlm.nih.gov/nucleotide/MF061760.1?report=genbank&log$=nucltop&blast_rank=1&RID=7V5R1UZJ016) | 924 | 99.41% |
| 18 | Jun | LIB | OM236688 | *Cladosporium oxysporum* | [MT079170.1](https://www.ncbi.nlm.nih.gov/nucleotide/MT079170.1?report=genbank&log$=nucltop&blast_rank=1&RID=7V5TFWXP01R) | 928 | 99.80% |
| 19 | Jun | LIB | OM236689 | *Cladosporium* sp. | [KM979743.1](https://www.ncbi.nlm.nih.gov/nucleotide/KM979743.1?report=genbank&log$=nucltop&blast_rank=1&RID=7V5W4MSD013) | 929 | 99.42% |
|  |  |  |  | *Cladosporium cladosporioides* | [MT878604.1](https://www.ncbi.nlm.nih.gov/nucleotide/MT878604.1?report=genbank&log$=nucltop&blast_rank=2&RID=7V5W4MSD013) | 928 | 99.41% |
| 20 | Jun | LIB | OM236690 | *Alternaria compacta* | [MW008933.1](https://www.ncbi.nlm.nih.gov/nucleotide/MW008933.1?report=genbank&log$=nucltop&blast_rank=1&RID=7V5Z4BYC013) | 976 | 100.00% |
| 21 | Jun | LIB | OM236691 | *Cladosporium cladosporioides* | [KU645993.1](https://www.ncbi.nlm.nih.gov/nucleotide/KU645993.1?report=genbank&log$=nucltop&blast_rank=1&RID=7V62P3JX013) | 643 | 88.09% |
| 22 | Jun | LIB | OM236692 | *Cladosporium oxysporum* | [KY400086.1](https://www.ncbi.nlm.nih.gov/nucleotide/KY400086.1?report=genbank&log$=nucltop&blast_rank=1&RID=7V65M8PK016) | 935 | 99.61% |
| 23 | Jun | CDP | OM236693 | *Alternaria alternata* | [MW008926.1](https://www.ncbi.nlm.nih.gov/nucleotide/MW008926.1?report=genbank&log$=nucltop&blast_rank=1&RID=7V682AJF01R) | 985 | 100.00% |
| 24 | Jun | CDO | OM236694 | *Alternaria alternata* | [MT090004.1](https://www.ncbi.nlm.nih.gov/nucleotide/MT090004.1?report=genbank&log$=nucltop&blast_rank=1&RID=7V6ATWH001R) | 985 | 99.81% |
| 25 | Jun | CDO | OM236695 | *Chaetomium globosum* | [MT510030.1](https://www.ncbi.nlm.nih.gov/nucleotide/MT510030.1?report=genbank&log$=nucltop&blast_rank=1&RID=7V6DGPUJ013) | 981 | 99.26% |
| 26 | Jun | CDO | OM236696 | Uncultured endophytic | [EF505560.1](https://www.ncbi.nlm.nih.gov/nucleotide/EF505560.1?report=genbank&log$=nucltop&blast_rank=1&RID=7V6G31G401R) | 989 | 99.45% |
|  |  |  |  | [*Leptosphaeria* sp.](#alnHdr_53125341) | AJ608969.1 | 965 | 98.03% |
| 27 | Jun | CDO | OM236697 | *Sarocladium strictum* | [GU219468.1](https://www.ncbi.nlm.nih.gov/nucleotide/GU219468.1?report=genbank&log$=nucltop&blast_rank=1&RID=7V6JWPDZ016) | 573 | 85.56% |
| 28 | Jun | CDO | OM236698 | *Cladosporium* sp. | [JX675049.1](https://www.ncbi.nlm.nih.gov/nucleotide/JX675049.1?report=genbank&log$=nucltop&blast_rank=1&RID=7V6P4A2H016) | 941 | 97.98% |
| 29 | Jun | CDO | OM236699 | Fungal sp. | [MT112977.1](https://www.ncbi.nlm.nih.gov/nucleotide/MT112977.1?report=genbank&log$=nucltop&blast_rank=1&RID=7V6SFRKY013) | 922 | 99.22% |
|  |  |  |  | *Cladosporium cucumerinum* | [MH464423.1](https://www.ncbi.nlm.nih.gov/nucleotide/MH464423.1?report=genbank&log$=nucltop&blast_rank=2&RID=7V6SFRKY013) | 920 | 98.47% |
| 30 | Jun | FDP | OM236700 | *Cladosporium cladosporioides* | [KU743893.1](https://www.ncbi.nlm.nih.gov/nucleotide/KU743893.1?report=genbank&log$=nucltop&blast_rank=1&RID=7V6VB85U013) | 917 | 99.02% |
| 31 | Jun | FDP | OM236701 | *Aspergillus oryzae* | [MT306006.1](https://www.ncbi.nlm.nih.gov/nucleotide/MT306006.1?report=genbank&log$=nucltop&blast_rank=1&RID=7V6YA56K016) | 963 | 98.71% |
| 32 | Jun | FDP | OM236702 | Uncultured endophytic | [EF505560.1](https://www.ncbi.nlm.nih.gov/nucleotide/EF505560.1?report=genbank&log$=nucltop&blast_rank=1&RID=7V718FHY016) | 992 | 99.63% |
|  | Jun |  |  | *Leptosphaeria* sp. | [MK367477.1](https://www.ncbi.nlm.nih.gov/nucleotide/MK367477.1?report=genbank&log$=nucltop&blast_rank=2&RID=7V718FHY016) | 961 | 98.89% |
| 33 | Jun | FDO | OM236703 | *Cladosporium cladosporioides* | [MT878604.1](https://www.ncbi.nlm.nih.gov/nucleotide/MT878604.1?report=genbank&log$=nucltop&blast_rank=1&RID=827R8E35016) | 942 | 99.23% |
| 34 | Jun | FDO | OM236704 | *Cladosporium cladosporioides* | [MT878604.1](https://www.ncbi.nlm.nih.gov/nucleotide/MT878604.1?report=genbank&log$=nucltop&blast_rank=1&RID=827W64R3016) | 933 | 99.23% |
| 35 | Jun | FDO | OM236705 | *Cladosporium tenuissimum* | [MK370679.1](https://www.ncbi.nlm.nih.gov/nucleotide/MK370679.1?report=genbank&log$=nucltop&blast_rank=1&RID=82840UFJ016) | 937 | 98.86% |
| 36 | Jun | FDO | OM236706 | *Sarocladium strictum* | [MW453217.1](https://www.ncbi.nlm.nih.gov/nucleotide/MW453217.1?report=genbank&log$=nucltop&blast_rank=1&RID=828CYAAU013) | 989 | 98.74% |
| 37 | Jun | FDO | OM236707 | Fungal sp. | [MW603421.1](https://www.ncbi.nlm.nih.gov/nucleotide/MW603421.1?report=genbank&log$=nucltop&blast_rank=1&RID=828P8WHD013) | 933 | 99.61% |
|  |  |  |  | *Cladosporium tenuissimum* | [KP689183.1](https://www.ncbi.nlm.nih.gov/nucleotide/KP689183.1?report=genbank&log$=nucltop&blast_rank=3&RID=828P8WHD013) | 931 | 99.61% |
| 38 | Jun | FDO | OM236708 | *Sarocladium strictum* | [MW453217.1](https://www.ncbi.nlm.nih.gov/nucleotide/MW453217.1?report=genbank&log$=nucltop&blast_rank=1&RID=828Y5FAS01R) | 989 | 98.74% |
| 39 | Jun | 3CO | OM236709 | *Alternaria alternata* | [MH460447.1](https://www.ncbi.nlm.nih.gov/nucleotide/MH460447.1?report=genbank&log$=nucltop&blast_rank=1&RID=82975CG2013) | 1047 | 99.62% |
| 40 | Jun | 3CO | OM236710 | *Dothiorella* sp. | [KX219605.1](https://www.ncbi.nlm.nih.gov/nucleotide/KX219605.1?report=genbank&log$=nucltop&blast_rank=1&RID=829JN6W0013) | 917 | 99.41% |
|  |  |  |  | *Nothophoma spiraeae* | [MN737834.1](https://www.ncbi.nlm.nih.gov/nucleotide/MN737834.1?report=genbank&log$=nucltop&blast_rank=2&RID=829JN6W0013) | 911 | 99.21% |
| 41 | Jun | 3CO | OM236711 | *Alternaria* sp. | [KF558883.1](https://www.ncbi.nlm.nih.gov/nucleotide/KF558883.1?report=genbank&log$=nucltop&blast_rank=1&RID=829WSP8W01R) | 972 | 99.62% |
|  |  |  |  | *Alternaria alternata* | [MW600446.1](https://www.ncbi.nlm.nih.gov/nucleotide/MW600446.1?report=genbank&log$=nucltop&blast_rank=2&RID=829WSP8W01R) | 972 | 99.62% |
| 42 | Jun | 3CP | OM236712 | *Aspergillus* sp. | [MN856344.1](https://www.ncbi.nlm.nih.gov/nucleotide/MN856344.1?report=genbank&log$=nucltop&blast_rank=1&RID=82A94R54016) | 989 | 99.09% |
|  |  |  |  | *Aspergillus westerdijkiae* | [MN856428.1](https://www.ncbi.nlm.nih.gov/nucleotide/MN856428.1?report=genbank&log$=nucltop&blast_rank=2&RID=82A94R54016) | 987 | 98.92% |
| 43 | Jun | 3CP | OM236713 | *Aspergillus protuberus* | [LC307169.1](https://www.ncbi.nlm.nih.gov/nucleotide/LC307169.1?report=genbank&log$=nucltop&blast_rank=1&RID=82AJ1CUV013) | 959 | 99.43% |
| 44 | Jun | 3CP | OM236714 | *Pleosporales* sp. | [MK564739.1](https://www.ncbi.nlm.nih.gov/nucleotide/MK564739.1?report=genbank&log$=nucltop&blast_rank=1&RID=82AZRPAE016) | 970 | 99.62% |
|  | Jun |  |  | *Paraphoma radicina* | [KY810511.1](https://www.ncbi.nlm.nih.gov/nucleotide/KY810511.1?report=genbank&log$=nucltop&blast_rank=2&RID=82AZRPAE016) | 968 | 99.62% |
| 45 | Jun | 5CO | OM236715 | *Cladosporium cladosporioides* | [MW582415.1](https://www.ncbi.nlm.nih.gov/nucleotide/MW582415.1?report=genbank&log$=nucltop&blast_rank=1&RID=82B8Y4GZ013) | 941 | 99.81% |
| 46 | Jun | 5CO | OM236716 | *Alternaria alternata* | [MF422130.1](https://www.ncbi.nlm.nih.gov/nucleotide/MF422130.1?report=genbank&log$=nucltop&blast_rank=1&RID=82BJ7KJE013) | 1112 | 99.09% |
| 47 | Jun | 5CO | OM236717 | *Alternaria* sp. | [MN096578.1](https://www.ncbi.nlm.nih.gov/nucleotide/MN096578.1?report=genbank&log$=nucltop&blast_rank=1&RID=82C5AGTE016) | 981 | 99.81% |
|  |  |  |  | *Alternaria tenuissima* | [KY400094.1](https://www.ncbi.nlm.nih.gov/nucleotide/KY400094.1?report=genbank&log$=nucltop&blast_rank=2&RID=82C5AGTE016) | 981 | 99.81% |
| 48 | Jun | 5CO | OM236718 | *Alternaria alternata* | [MN856409.1](https://www.ncbi.nlm.nih.gov/nucleotide/MN856409.1?report=genbank&log$=nucltop&blast_rank=1&RID=82CC9H6V01R) | 976 | 99.63% |
| 49 | Jun | 5CO | OM236719 | *Cladosporium cladosporioides* | [MW582415.1](https://www.ncbi.nlm.nih.gov/nucleotide/MW582415.1?report=genbank&log$=nucltop&blast_rank=1&RID=82CT8JXS016) | 924 | 100.00% |
| 50 | Jun | 5CO | OM236720 | *Cladosporium cladosporioides* | [MW582415.1](https://www.ncbi.nlm.nih.gov/nucleotide/MW582415.1?report=genbank&log$=nucltop&blast_rank=1&RID=82G74Y9S01R) | 924 | 100.00% |
| 51 | Jun | 5CP | OM236721 | *Alternaria alternata* | [MH460447.1](https://www.ncbi.nlm.nih.gov/nucleotide/MH460447.1?report=genbank&log$=nucltop&blast_rank=1&RID=82GFGVK6013) | 1064 | 99.09% |
| 52 | Jun | 5CP | OM236722 | *Cladosporium* sp. | [MF380508.1](https://www.ncbi.nlm.nih.gov/nucleotide/MF380508.1?report=genbank&log$=nucltop&blast_rank=1&RID=82GVP4Y6013) | 931 | 99.42% |
| 53 | Jun | 5CP | OM236723 | *Alternaria* sp. | [MN856394.1](https://www.ncbi.nlm.nih.gov/nucleotide/MN856394.1?report=genbank&log$=nucltop&blast_rank=1&RID=82H0YF97013) | 972 | 99.62% |
|  |  |  |  | *Alternaria alternata* | [MK518403.1](https://www.ncbi.nlm.nih.gov/nucleotide/MK518403.1?report=genbank&log$=nucltop&blast_rank=2&RID=82H0YF97013) | 972 | 99.08% |
| 54 | Jun | 5CP | OM236724 | *Alternaria alternata* | [MF422130.1](https://www.ncbi.nlm.nih.gov/nucleotide/MF422130.1?report=genbank&log$=nucltop&blast_rank=1&RID=82H9G23T013) | 1108 | 99.27% |
| 55 | Jun | 5CP | OM236725 | *Cladosporium* sp. | [KT989416.1](https://www.ncbi.nlm.nih.gov/nucleotide/KT989416.1?report=genbank&log$=nucltop&blast_rank=1&RID=82HK29A901R) | 944 | 99.61% |
|  |  |  |  | *Cladosporium cladosporioides* | [JX868638.1](https://www.ncbi.nlm.nih.gov/nucleotide/JX868638.1?report=genbank&log$=nucltop&blast_rank=2&RID=82HK29A901R) | 944 | 99.81% |
| 56 | Jun | PS | OM236726 | *Alternaria* sp. | [MN096578.1](https://www.ncbi.nlm.nih.gov/nucleotide/MN096578.1?report=genbank&log$=nucltop&blast_rank=1&RID=82HXR59P016) | 983 | 99.81% |
|  |  |  |  | *Alternaria alternata* | [MK966433.1](https://www.ncbi.nlm.nih.gov/nucleotide/MK966433.1?report=genbank&log$=nucltop&blast_rank=2&RID=82HXR59P016) | 981 | 99.81% |
| 57 | Jun | PS | OM236727 | *Cladosporium* sp. | [MK111616.1](https://www.ncbi.nlm.nih.gov/nucleotide/MK111616.1?report=genbank&log$=nucltop&blast_rank=1&RID=82XZGPUH013) | 937 | 100.00% |
| 58 | Jun | PS | OM236728 | *Alternaria alternata* | [MH938076.1](https://www.ncbi.nlm.nih.gov/nucleotide/MH938076.1?report=genbank&log$=nucltop&blast_rank=1&RID=82YAWW8Y01R) | 950 | 99.62% |
| 59 | Jun | PS | OM236729 | *Cladosporium cladosporioides* | [MW582379.1](https://www.ncbi.nlm.nih.gov/nucleotide/MW582379.1?report=genbank&log$=nucltop&blast_rank=1&RID=82YRKA4V016) | 948 | 99.62% |
| 60 | Jun | PS | OM236730 | *Epicoccum* sp. | [GU973785.1](https://www.ncbi.nlm.nih.gov/nucleotide/GU973785.1?report=genbank&log$=nucltop&blast_rank=1&RID=82Z347SZ013) | 900 | 100.00% |
| 61 | Jun | PS | OM236731 | *Cladosporium cladosporioides* | [MW582379.1](https://www.ncbi.nlm.nih.gov/nucleotide/MW582379.1?report=genbank&log$=nucltop&blast_rank=1&RID=82ZCJZ6H016) | 939 | 99.42% |
| 62 | Jun | PS | OM236732 | *Alternaria tenuissima* | [MK285656.1](https://www.ncbi.nlm.nih.gov/nucleotide/MK285656.1?report=genbank&log$=nucltop&blast_rank=1&RID=82ZMRM3901R) | 965 | 99.81% |
| 63 | Jun | PS | OM236733 | *Alternaria tenuissima* | [FJ812353.1](https://www.ncbi.nlm.nih.gov/nucleotide/FJ812353.1?report=genbank&log$=nucltop&blast_rank=1&RID=82ZZKNKV016) | 955 | 99.43% |
| 1 | Jul | LIB | OM236734 | [*Cladosporium anthropophilum*](https://blast.ncbi.nlm.nih.gov/Blast.cgi) | [MK111497.1](https://www.ncbi.nlm.nih.gov/nucleotide/MK111497.1?report=genbank&log$=nucltop&blast_rank=1&RID=BW352EGN013) | 937 | 99.61% |
| 2 | Jul | LIB | OM236735 | [Fungal sp.](https://blast.ncbi.nlm.nih.gov/Blast.cgi) | [MW603421.1](https://www.ncbi.nlm.nih.gov/nucleotide/MW603421.1?report=genbank&log$=nucltop&blast_rank=1&RID=BW39XRW3016) | 935 | 99.23% |
|  |  |  |  | [*Cladosporium anthropophilum*](https://blast.ncbi.nlm.nih.gov/Blast.cgi) | [MK111497.1](https://www.ncbi.nlm.nih.gov/nucleotide/MK111497.1?report=genbank&log$=nucltop&blast_rank=2&RID=BW39XRW3016) | 933 | 100.00% |
| 3 | Jul | LIB | OM236736 | [*Alternaria* sp.](https://blast.ncbi.nlm.nih.gov/Blast.cgi) | [MW581348.1](https://www.ncbi.nlm.nih.gov/nucleotide/MW581348.1?report=genbank&log$=nucltop&blast_rank=1&RID=BW3JD56D013) | 981 | 99.81% |
|  |  |  |  | [*Alternaria alternata*](https://blast.ncbi.nlm.nih.gov/Blast.cgi) | [MW009024.1](https://www.ncbi.nlm.nih.gov/nucleotide/MW009024.1?report=genbank&log$=nucltop&blast_rank=2&RID=BW3JD56D013) | 981 | 100.00% |
| 4 | Jul | LIB | OM236737 | [*Alternaria* sp.](https://blast.ncbi.nlm.nih.gov/Blast.cgi) | [MW581348.1](https://www.ncbi.nlm.nih.gov/nucleotide/MW581348.1?report=genbank&log$=nucltop&blast_rank=1&RID=BW3P3BK3016) | 985 | 99.63% |
|  |  |  |  | [*Alternaria angustiovoidea*](https://blast.ncbi.nlm.nih.gov/Blast.cgi) | [MW008876.1](https://www.ncbi.nlm.nih.gov/nucleotide/MW008876.1?report=genbank&log$=nucltop&blast_rank=3&RID=BW3P3BK3016) | 985 | 99.81% |
| 5 | Jul | 5CO | OM236738 | [*Alternaria alternata*](https://blast.ncbi.nlm.nih.gov/Blast.cgi) | [MW008892.1](https://www.ncbi.nlm.nih.gov/nucleotide/MW008892.1?report=genbank&log$=nucltop&blast_rank=1&RID=BW3WBK0J013) | 983 | 100.00% |
| 6 | Jul | 5CO | OM236739 | [*Cladosporium tenuissimum*](https://blast.ncbi.nlm.nih.gov/Blast.cgi) | [JN624886.1](https://www.ncbi.nlm.nih.gov/nucleotide/JN624886.1?report=genbank&log$=nucltop&blast_rank=1&RID=BW406TKP013) | 931 | 99.23% |
| 7 | Jul | 5CO | OM236740 | [*Alternaria* sp.](https://blast.ncbi.nlm.nih.gov/Blast.cgi) | [MW009028.1](https://www.ncbi.nlm.nih.gov/nucleotide/MW009028.1?report=genbank&log$=nucltop&blast_rank=1&RID=BW44XEJD016) | 989 | 99.45% |
|  |  |  |  | [*Alternaria tamaricis*](https://blast.ncbi.nlm.nih.gov/Blast.cgi) | [MW009009.1](https://www.ncbi.nlm.nih.gov/nucleotide/MW009009.1?report=genbank&log$=nucltop&blast_rank=2&RID=BW44XEJD016) | 985 | 99.63% |
| 8 | Jul | 5CO | OM236741 | [*Cladosporium tenuissimum*](https://blast.ncbi.nlm.nih.gov/Blast.cgi) | [MH270558.1](https://www.ncbi.nlm.nih.gov/nucleotide/MH270558.1?report=genbank&log$=nucltop&blast_rank=1&RID=BW49UHAH016) | 929 | 99.61% |
| 9 | Jul | FDO | OM236742 | [*Didymella macrostoma*](https://blast.ncbi.nlm.nih.gov/Blast.cgi) | [MN944409.1](https://www.ncbi.nlm.nih.gov/nucleotide/MN944409.1?report=genbank&log$=nucltop&blast_rank=1&RID=BW4ZCU6X016) | 924 | 99.22% |
| 10 | Jul | FDO | OM236743 | [*Alternaria angustiovoidea*](https://blast.ncbi.nlm.nih.gov/Blast.cgi) | [MW009022.1](https://www.ncbi.nlm.nih.gov/nucleotide/MW009022.1?report=genbank&log$=nucltop&blast_rank=1&RID=BW54SZS4013) | 983 | 100.00% |
| 11 | Jul | FDO | OM236744 | [*Alternaria alternata*](https://blast.ncbi.nlm.nih.gov/Blast.cgi) | [MW008875.1](https://www.ncbi.nlm.nih.gov/nucleotide/MW008875.1?report=genbank&log$=nucltop&blast_rank=1&RID=BW597A3D013) | 998 | 99.63% |
| 12 | Jul | FDO | OM236745 | [*Aspergillus niger*](https://blast.ncbi.nlm.nih.gov/Blast.cgi) | [MT620753.1](https://www.ncbi.nlm.nih.gov/nucleotide/MT620753.1?report=genbank&log$=nucltop&blast_rank=1&RID=BW5EU672013) | 859 | 99.79% |
| 13 | Jul | CDO | OM236746 | [*Cladosporium cladosporioides*](https://blast.ncbi.nlm.nih.gov/Blast.cgi) | [MK761055.1](https://www.ncbi.nlm.nih.gov/nucleotide/MK761055.1?report=genbank&log$=nucltop&blast_rank=1&RID=BW5K1GP701R) | 937 | 99.80% |
| 14 | Jul | CDO | OM236747 | [*Alternaria* sp.](https://blast.ncbi.nlm.nih.gov/Blast.cgi) | [MW009026.1](https://www.ncbi.nlm.nih.gov/nucleotide/MW009026.1?report=genbank&log$=nucltop&blast_rank=1&RID=BW6233JW013) | 985 | 99.81% |
|  |  |  |  | [*Alternaria tamaricis*](https://blast.ncbi.nlm.nih.gov/Blast.cgi) | [KY400101.1](https://www.ncbi.nlm.nih.gov/nucleotide/KY400101.1?report=genbank&log$=nucltop&blast_rank=3&RID=BW6233JW013) | 983 | 98.91% |
| 15 | Jul | CDP | OM236748 | [*Alternaria alternata*](https://blast.ncbi.nlm.nih.gov/Blast.cgi) | [MW008970.1](https://www.ncbi.nlm.nih.gov/nucleotide/MW008970.1?report=genbank&log$=nucltop&blast_rank=1&RID=BW65V60G01R) | 987 | 99.45% |
| 16 | Jul | CDP | OM236749 | [*Penicillium mallochii*](https://blast.ncbi.nlm.nih.gov/Blast.cgi) | [MN944416.1](https://www.ncbi.nlm.nih.gov/nucleotide/MN944416.1?report=genbank&log$=nucltop&blast_rank=1&RID=BW69FJTU016) | 990 | 99.45% |
| 17 | Jul | PS | OM236750 | [*Alternaria* sp.](https://blast.ncbi.nlm.nih.gov/Blast.cgi) | [MW009028.1](https://www.ncbi.nlm.nih.gov/nucleotide/MW009028.1?report=genbank&log$=nucltop&blast_rank=1&RID=BW6CS9HS016) | 989 | 99.45% |
|  |  |  |  | [*Alternaria compacta*](https://blast.ncbi.nlm.nih.gov/Blast.cgi) | [MW009019.1](https://www.ncbi.nlm.nih.gov/nucleotide/MW009019.1?report=genbank&log$=nucltop&blast_rank=3&RID=BW6CS9HS016) | 985 | 99.45% |
| 18 | Jul | PS | OM236751 | [*Alternaria angustiovoidea*](https://blast.ncbi.nlm.nih.gov/Blast.cgi) | [MW009022.1](https://www.ncbi.nlm.nih.gov/nucleotide/MW009022.1?report=genbank&log$=nucltop&blast_rank=1&RID=BW6G2HUK01R) | 994 | 99.82% |
| 19 | Jul | PS | OM236752 | [*Cladosporium* sp.](https://blast.ncbi.nlm.nih.gov/Blast.cgi) | [MG572460.1](https://www.ncbi.nlm.nih.gov/nucleotide/MG572460.1?report=genbank&log$=nucltop&blast_rank=1&RID=BW6KPPZW013) | 939 | 99.80% |
|  |  |  |  | [*Cladosporium asperulatum*](https://blast.ncbi.nlm.nih.gov/Blast.cgi) | [KX982236.1](https://www.ncbi.nlm.nih.gov/nucleotide/KX982236.1?report=genbank&log$=nucltop&blast_rank=3&RID=BW6KPPZW013) | 935 | 99.80% |
| 20 | Jul | PS | OM236753 | [*Alternaria alternata*](https://blast.ncbi.nlm.nih.gov/Blast.cgi) | [MW008875.1](https://www.ncbi.nlm.nih.gov/nucleotide/MW008875.1?report=genbank&log$=nucltop&blast_rank=1&RID=BW6RDJR2016) | 996 | 99.63% |
| 21 | Jul | PS | OM236754 | [*Alternaria alternata*](https://blast.ncbi.nlm.nih.gov/Blast.cgi) | [MW008875.1](https://www.ncbi.nlm.nih.gov/nucleotide/MW008875.1?report=genbank&log$=nucltop&blast_rank=1&RID=BW6UXFJN016) | 998 | 99.63% |
| 22 | Jul | PS | OM236755 | [*Alternaria alternata*](https://blast.ncbi.nlm.nih.gov/Blast.cgi) | [MW008875.](https://www.ncbi.nlm.nih.gov/nucleotide/MW008875.1?report=genbank&log$=nucltop&blast_rank=1&RID=BW6YNHRM016) | 998 | 99.63% |
| 23 | Jul | PS | OM236756 | [*Alternaria tamaricis*](https://blast.ncbi.nlm.nih.gov/Blast.cgi) | [KY400101.1](https://www.ncbi.nlm.nih.gov/nucleotide/KY400101.1?report=genbank&log$=nucltop&blast_rank=1&RID=BW72BTV8013) | 985 | 99.81% |
| 24 | Jul | PS | OM236757 | [*Alternaria* sp.](https://blast.ncbi.nlm.nih.gov/Blast.cgi) | [MW009028.1](https://www.ncbi.nlm.nih.gov/nucleotide/MW009028.1?report=genbank&log$=nucltop&blast_rank=1&RID=BW75NVRN016) | 983 | 100.00% |
|  |  |  |  | [*Alternaria solani*](https://blast.ncbi.nlm.nih.gov/Blast.cgi) | [MW009039.1](https://www.ncbi.nlm.nih.gov/nucleotide/MW009039.1?report=genbank&log$=nucltop&blast_rank=2&RID=BW75NVRN016) | 981 | 99.81% |
| 25 | Jul | PS | OM236758 | [*Cladosporium* sp.](https://blast.ncbi.nlm.nih.gov/Blast.cgi) | [MK640598.1](https://www.ncbi.nlm.nih.gov/nucleotide/MK640598.1?report=genbank&log$=nucltop&blast_rank=1&RID=BW79162U013) | 845 | 99.57% |
|  |  |  |  | [*Cladosporium tenuissimum*](https://blast.ncbi.nlm.nih.gov/Blast.cgi) | [MN429197.](https://www.ncbi.nlm.nih.gov/nucleotide/MN429197.1?report=genbank&log$=nucltop&blast_rank=2&RID=BW79162U013) | 845 | 99.57% |
| 26 | Jul | PS | OM236759 | [*Alternaria tamaricis*](https://blast.ncbi.nlm.nih.gov/Blast.cgi) | [MW008886.1](https://www.ncbi.nlm.nih.gov/nucleotide/MW008886.1?report=genbank&log$=nucltop&blast_rank=1&RID=BW7CSCY4013) | 994 | 99.45% |
| 27 | Jul | PS | OM236760 | [*Alternaria tenuissima*](https://blast.ncbi.nlm.nih.gov/Blast.cgi) | [MW008898.1](https://www.ncbi.nlm.nih.gov/nucleotide/MW008898.1?report=genbank&log$=nucltop&blast_rank=1&RID=BW7G0XR5013) | 983 | 99.63% |
| 28 | Jul | 3CP | OM236761 | [*Allophoma labilis*](https://blast.ncbi.nlm.nih.gov/Blast.cgi) | [KT013227.1](https://www.ncbi.nlm.nih.gov/nucleotide/KT013227.1?report=genbank&log$=nucltop&blast_rank=1&RID=BW7KKZR3016) | 1043 | 98.14% |
| 29 | Jul | 3CP | OM236762 | [Fungal endophyte](https://blast.ncbi.nlm.nih.gov/Blast.cgi) | [KP335555.1](https://www.ncbi.nlm.nih.gov/nucleotide/KP335555.1?report=genbank&log$=nucltop&blast_rank=1&RID=BW7RXZ8Y013) | 937 | 97.64% |
|  |  |  |  | [*Aureobasidium iranianum*](https://blast.ncbi.nlm.nih.gov/Blast.cgi) | [KY781746.1](https://www.ncbi.nlm.nih.gov/nucleotide/KY781746.1?report=genbank&log$=nucltop&blast_rank=4&RID=BW7RXZ8Y013) | 922 | 97.10% |
| 30 | Jul | 3CP | OM236763 | [Fungal endophyte](https://blast.ncbi.nlm.nih.gov/Blast.cgi) | [KP335555.1](https://www.ncbi.nlm.nih.gov/nucleotide/KP335555.1?report=genbank&log$=nucltop&blast_rank=1&RID=BW7WB549013) | 957 | 98.19% |
|  |  |  |  | [*Aureobasidium iranianum*](https://blast.ncbi.nlm.nih.gov/Blast.cgi) | [KM093738.1](https://www.ncbi.nlm.nih.gov/nucleotide/KM093738.1?report=genbank&log$=nucltop&blast_rank=4&RID=BW7WB549013) | 944 | 97.64% |
| 31 | Jul | 3CP | OM236764 | *Alternaria tamaricis* | [MW008886.1](https://www.ncbi.nlm.nih.gov/nucleotide/MW008886.1?report=genbank&log$=nucltop&blast_rank=1&RID=EUF28M14016) | 977 | 98.55% |
| 32 | Jul | 3CP | OM236765 | [Fungal endophyte](https://blast.ncbi.nlm.nih.gov/Blast.cgi) | [KP335555.1](https://www.ncbi.nlm.nih.gov/nucleotide/KP335555.1?report=genbank&log$=nucltop&blast_rank=1&RID=BW8223P8013) | 900 | 96.87% |
|  |  |  |  | [*Aureobasidium iranianum*](https://blast.ncbi.nlm.nih.gov/Blast.cgi) | [KY781746.1](https://www.ncbi.nlm.nih.gov/nucleotide/KY781746.1?report=genbank&log$=nucltop&blast_rank=4&RID=BW8223P8013) | 885 | 96.32% |
| 33 | Jul | 3CP | OM236766 | [*Alternaria* sp.](https://blast.ncbi.nlm.nih.gov/Blast.cgi) | [MN519439.1](https://www.ncbi.nlm.nih.gov/nucleotide/MN519439.1?report=genbank&log$=nucltop&blast_rank=1&RID=BW89BZVJ013) | 981 | 99.45% |
|  |  |  |  | [*Alternaria angustiovoidea*](https://blast.ncbi.nlm.nih.gov/Blast.cgi) | [MK910070.1](https://www.ncbi.nlm.nih.gov/nucleotide/MK910070.1?report=genbank&log$=nucltop&blast_rank=2&RID=BW89BZVJ013) | 981 | 99.45% |
| 34 | Jul | 3CP | OM236767 | [*Alternaria solani*](https://blast.ncbi.nlm.nih.gov/Blast.cgi) | [MW009039.1](https://www.ncbi.nlm.nih.gov/nucleotide/MW009039.1?report=genbank&log$=nucltop&blast_rank=1&RID=BW8D3B7U013) | 989 | 100.00% |
| 35 | Jul | 3CO | OM236768 | [*Alternaria* sp.](https://blast.ncbi.nlm.nih.gov/Blast.cgi) | [MW009028.1](https://www.ncbi.nlm.nih.gov/nucleotide/MW009028.1?report=genbank&log$=nucltop&blast_rank=1&RID=BW8GM37U013) | 994 | 99.63% |
|  |  |  |  | [*Alternaria tamaricis*](https://blast.ncbi.nlm.nih.gov/Blast.cgi) | [KY400101.1](https://www.ncbi.nlm.nih.gov/nucleotide/KY400101.1?report=genbank&log$=nucltop&blast_rank=2&RID=BW8GM37U013) | 992 | 99.45% |
| 36 | Jul | 3CO | OM236769 | [*Alternaria solani*](https://blast.ncbi.nlm.nih.gov/Blast.cgi) | [MW009039.1](https://www.ncbi.nlm.nih.gov/nucleotide/MW009039.1?report=genbank&log$=nucltop&blast_rank=1&RID=BW8M40R9016) | 987 | 99.81% |
| 37 | Jul | 3CO | OM236770 | [*Peyronellaea prosopidis*](https://blast.ncbi.nlm.nih.gov/Blast.cgi) | [MG323881.1](https://www.ncbi.nlm.nih.gov/nucleotide/MG323881.1?report=genbank&log$=nucltop&blast_rank=1&RID=BW8STV54013) | 918 | 99.80% |
| 38 | Jul | 3CO | OM236771 | [*Alternaria compacta*](https://blast.ncbi.nlm.nih.gov/Blast.cgi) | [MW008924.1](https://www.ncbi.nlm.nih.gov/nucleotide/MW008924.1?report=genbank&log$=nucltop&blast_rank=1&RID=BW8W7N09016) | 987 | 99.63% |
| 39 | Jul | 3CO | OM236772 | *Botrytis cinerea* | MT708074.1 | 1128 | 0.9865 |
| 40 | Jul | 3CO | OM236773 | [*Alternaria alternata*](https://blast.ncbi.nlm.nih.gov/Blast.cgi) | [MT635274.1](https://www.ncbi.nlm.nih.gov/nucleotide/MT635274.1?report=genbank&log$=nucltop&blast_rank=1&RID=BW908B3F013) | 885 | 99.39% |
| 41 | Jul | 3CO | OM236774 | *Alternaria* sp. | MK037454.1 | 974 | 99.44% |
| 42 | Jul | CDO | OM236775 | [*Alternaria alternata*](https://blast.ncbi.nlm.nih.gov/Blast.cgi) | [MW008911.1](https://www.ncbi.nlm.nih.gov/nucleotide/MW008911.1?report=genbank&log$=nucltop&blast_rank=1&RID=BW94VVR8013) | 990 | 100.00% |
| 43 | Jul | LIB | OM236776 | [*Alternaria* sp.](https://blast.ncbi.nlm.nih.gov/Blast.cgi) | [MW008935.1](https://www.ncbi.nlm.nih.gov/nucleotide/MW008935.1?report=genbank&log$=nucltop&blast_rank=1&RID=BW987TM5013) | 983 | 99.63% |
|  |  |  |  | [*Alternaria compacta*](https://blast.ncbi.nlm.nih.gov/Blast.cgi) | [MW008928.1](https://www.ncbi.nlm.nih.gov/nucleotide/MW008928.1?report=genbank&log$=nucltop&blast_rank=3&RID=BW987TM5013) | 979 | 99.81% |
| 44 | Jul | LIB | OM236777 | [*Penicillium indicum*](https://blast.ncbi.nlm.nih.gov/Blast.cgi) | [NR_121311.1](https://www.ncbi.nlm.nih.gov/nucleotide/NR_121311.1?report=genbank&log$=nucltop&blast_rank=1&RID=BW9BD65V016) | 994 | 99.10% |
| 45 | Jul | 5CO | OM236778 | [*Alternaria alternata*](https://blast.ncbi.nlm.nih.gov/Blast.cgi) | [MW008961.1](https://www.ncbi.nlm.nih.gov/nucleotide/MW008961.1?report=genbank&log$=nucltop&blast_rank=1&RID=BW9EHZKK013) | 983 | 99.63% |
| 46 | Jul | 3CP | OM236779 | [*Cytospora* sp.](https://blast.ncbi.nlm.nih.gov/Blast.cgi) | KF944452.1 | 1042 | 99.65% |
|  |  |  |  | [*Valsa nivea*](https://blast.ncbi.nlm.nih.gov/Blast.cgi) | KF293778.1 | 1040 | 99.65% |
| 47 | Jul | PS | OM236780 | [*Cladosporium herbarum*](https://blast.ncbi.nlm.nih.gov/Blast.cgi) | [MF288739.1](https://www.ncbi.nlm.nih.gov/nucleotide/MF288739.1?report=genbank&log$=nucltop&blast_rank=1&RID=BW9HY0FK013) | 893 | 98.24% |
| 48 | Jul | PS | OM236781 | [Fungal sp.](https://blast.ncbi.nlm.nih.gov/Blast.cgi) | [MT112989.1](https://www.ncbi.nlm.nih.gov/nucleotide/MT112989.1?report=genbank&log$=nucltop&blast_rank=1&RID=BW9RA1ZE01R) | 942 | 99.61% |
|  |  |  |  | [*Cladosporium ramotenellum*](https://blast.ncbi.nlm.nih.gov/Blast.cgi) | [MK387913.1](https://www.ncbi.nlm.nih.gov/nucleotide/MK387913.1?report=genbank&log$=nucltop&blast_rank=3&RID=BW9RA1ZE01R) | 939 | 99.42% |
| 49 | Jul | FDP | OM236782 | [*Neurospora crassa*](https://blast.ncbi.nlm.nih.gov/Blast.cgi) | [MW689660.1](https://www.ncbi.nlm.nih.gov/nucleotide/MW689660.1?report=genbank&log$=nucltop&blast_rank=1&RID=BW9USBB0013) | 1011 | 99.64% |
| 50 | Jul | CDP | OM236783 | [*Alternaria* sp.](https://blast.ncbi.nlm.nih.gov/Blast.cgi) | [MK649973.1](https://www.ncbi.nlm.nih.gov/nucleotide/MK649973.1?report=genbank&log$=nucltop&blast_rank=1&RID=BW9Y6U0F013) | 990 | 99.81% |
|  |  |  |  | [*Alternaria alternata*](https://blast.ncbi.nlm.nih.gov/Blast.cgi) | [KT366738.1](https://www.ncbi.nlm.nih.gov/nucleotide/KT366738.1?report=genbank&log$=nucltop&blast_rank=2&RID=BW9Y6U0F013) | 987 | 99.63% |
| 51 | Jul | CDP | OM236784 | [*Aspergillus niger*](https://blast.ncbi.nlm.nih.gov/Blast.cgi) | [MT597434.1](https://www.ncbi.nlm.nih.gov/nucleotide/MT597434.1?report=genbank&log$=nucltop&blast_rank=1&RID=BWA1BVGY013) | 1037 | 99.30% |
| 52 | Jul | CDO | OM236785 | [Uncultured *Taphrina*](https://blast.ncbi.nlm.nih.gov/Blast.cgi) | [MF555586.1](https://www.ncbi.nlm.nih.gov/nucleotide/MF555586.1?report=genbank&log$=nucltop&blast_rank=1&RID=BWA546Z0013) | 438 | 99.59% |
|  |  |  |  | [*Taphrina betulina*](https://blast.ncbi.nlm.nih.gov/Blast.cgi) | [MN540705.1](https://www.ncbi.nlm.nih.gov/nucleotide/MN540705.1?report=genbank&log$=nucltop&blast_rank=4&RID=BWA546Z0013) | 422 | 84.74% |
| 53 | Jul | 5CP | OM236786 | [*Cladosporium* sp.](https://blast.ncbi.nlm.nih.gov/Blast.cgi) | [KY827345.1](https://www.ncbi.nlm.nih.gov/nucleotide/KY827345.1?report=genbank&log$=nucltop&blast_rank=1&RID=BWACBPJT013) | 832 | 99.13% |
|  |  |  |  | [*Cladosporium tenuissimum*](https://blast.ncbi.nlm.nih.gov/Blast.cgi) | [MT508793.1](https://www.ncbi.nlm.nih.gov/nucleotide/MT508793.1?report=genbank&log$=nucltop&blast_rank=2&RID=BWACBPJT013) | 826 | 98.92% |
| 54 | Jul | 5CP | OM236787 | [*Periconia* sp.](https://blast.ncbi.nlm.nih.gov/Blast.cgi) | MW391746.1 | 924 | 99.03% |
| 55 | Jul | 5CP | OM236788 | [*Alternaria alternata*](https://blast.ncbi.nlm.nih.gov/Blast.cgi) | [MW009041.1](https://www.ncbi.nlm.nih.gov/nucleotide/MW009041.1?report=genbank&log$=nucltop&blast_rank=1&RID=BWAN2GM2013) | 983 | 99.81% |
| 56 | Jul | 5CP | OM236789 | [*Alternaria alternata*](https://blast.ncbi.nlm.nih.gov/Blast.cgi) | [MN826219.1](https://www.ncbi.nlm.nih.gov/nucleotide/MN826219.1?report=genbank&log$=nucltop&blast_rank=1&RID=BWAVE69A016) | 898 | 100.00% |
| 57 | Jul | 5CP | OM236790 | [Fungal sp.](https://blast.ncbi.nlm.nih.gov/Blast.cgi) | [MW603421.1](https://www.ncbi.nlm.nih.gov/nucleotide/MW603421.1?report=genbank&log$=nucltop&blast_rank=1&RID=BWAZB93Y013) | 953 | 100.00% |
|  |  |  |  | [*Cladosporium tenuissimum*](https://blast.ncbi.nlm.nih.gov/Blast.cgi) | [KP689183.1](https://www.ncbi.nlm.nih.gov/nucleotide/KP689183.1?report=genbank&log$=nucltop&blast_rank=2&RID=BWAZB93Y013) | 946 | 99.81% |
| 58 | Jul | 5CP | OM236791 | [*Penicillium oxalicum*](https://blast.ncbi.nlm.nih.gov/Blast.cgi) | [KY400080.1](https://www.ncbi.nlm.nih.gov/nucleotide/KY400080.1?report=genbank&log$=nucltop&blast_rank=1&RID=BWB2RUJ7013) | 1009 | 99.11% |
| 59 | Jul | 5CP | OM236792 | [*Cladosporium tenuissimum*](https://blast.ncbi.nlm.nih.gov/Blast.cgi) | [MN429197.1](https://www.ncbi.nlm.nih.gov/nucleotide/MN429197.1?report=genbank&log$=nucltop&blast_rank=1&RID=BWB5ZBRW01R) | 946 | 99.61% |
| 60 | Jul | 5CP | OM236793 | [*Alternaria compacta*](https://blast.ncbi.nlm.nih.gov/Blast.cgi) | [MW008928.1](https://www.ncbi.nlm.nih.gov/nucleotide/MW008928.1?report=genbank&log$=nucltop&blast_rank=1&RID=BWB9B89701R) | 979 | 100.00% |
| 61 | Jul | 5CP | OM236794 | [*Phaeosphaeriaceae* sp.](https://blast.ncbi.nlm.nih.gov/Blast.cgi) | [KU991891.1](https://www.ncbi.nlm.nih.gov/nucleotide/KU991891.1?report=genbank&log$=nucltop&blast_rank=1&RID=BWBCW4KN013) | 972 | 98.72% |
|  |  |  |  | [*Phoma betae*](https://blast.ncbi.nlm.nih.gov/Blast.cgi) | [MK460862.1](https://www.ncbi.nlm.nih.gov/nucleotide/MK460862.1?report=genbank&log$=nucltop&blast_rank=2&RID=BWBCW4KN013) | 970 | 98.72% |
| 62 | Jul | 5CP | OM236795 | [*Cladosporium* sp.](https://blast.ncbi.nlm.nih.gov/Blast.cgi) | [MT729897.1](https://www.ncbi.nlm.nih.gov/nucleotide/MT729897.1?report=genbank&log$=nucltop&blast_rank=1&RID=BWBGPSV5013) | 929 | 99.61% |
|  |  |  |  | [*Cladosporium cladosporioides*](https://blast.ncbi.nlm.nih.gov/Blast.cgi) | [MT729833.1](https://www.ncbi.nlm.nih.gov/nucleotide/MT729833.1?report=genbank&log$=nucltop&blast_rank=2&RID=BWBGPSV5013) | 929 | 99.61% |
| 63 | Jul | 5CP | OM236796 | [*Aspergillus niger*](https://blast.ncbi.nlm.nih.gov/Blast.cgi) | [MG647867.1](https://www.ncbi.nlm.nih.gov/nucleotide/MG647867.1?report=genbank&log$=nucltop&blast_rank=1&RID=EUD8YC65016) | 1016 | 98.94% |
| 64 | Jul | 5CP | OM236797 | [*Cladosporium oxysporum*](https://blast.ncbi.nlm.nih.gov/Blast.cgi) | [MF040201.1](https://www.ncbi.nlm.nih.gov/nucleotide/MF040201.1?report=genbank&log$=nucltop&blast_rank=1&RID=EGHB853B013) | 931 | 98.85% |
| 65 | Jul | 5CP | OM236798 | [*Cladosporium oxysporum*](https://blast.ncbi.nlm.nih.gov/Blast.cgi) | [MF040201.1](https://www.ncbi.nlm.nih.gov/nucleotide/MF040201.1?report=genbank&log$=nucltop&blast_rank=1&RID=BWBKUGJM01R) | 931 | 99.61% |
| 66 | Jul | 5CP | OM236799 | [*Stagonospora* sp.](https://blast.ncbi.nlm.nih.gov/Blast.cgi) | [MW866691.1](https://www.ncbi.nlm.nih.gov/nucleotide/MW866691.1?report=genbank&log$=nucltop&blast_rank=1&RID=BWBR9DC901R) | 937 | 97.46% |
| 67 | Jul | 5CP | OM236800 | [*Aspergillus ochraceus*](https://blast.ncbi.nlm.nih.gov/Blast.cgi) | [MT582750.1](https://www.ncbi.nlm.nih.gov/nucleotide/MT582750.1?report=genbank&log$=nucltop&blast_rank=1&RID=BWBUVJGT016) | 905 | 99.40% |
| 68 | Jul | 5CP | OM236801 | [*Aspergillus flavus*](https://blast.ncbi.nlm.nih.gov/Blast.cgi) | [MW805395.1](https://www.ncbi.nlm.nih.gov/nucleotide/MW805395.1?report=genbank&log$=nucltop&blast_rank=1&RID=EGHWWAC6013) | 976 | 98.21% |
| 69 | Jul | 5CP | OM236802 | [*Cladosporium cladosporioides*](https://blast.ncbi.nlm.nih.gov/Blast.cgi) | [KX426943.1](https://www.ncbi.nlm.nih.gov/nucleotide/KX426943.1?report=genbank&log$=nucltop&blast_rank=1&RID=BWBZ0ZM9016) | 929 | 99.41% |
| 70 | Jul | 5CP | OM236803 | [*Cladosporium perangustum*](https://blast.ncbi.nlm.nih.gov/Blast.cgi) | [KY400099.1](https://www.ncbi.nlm.nih.gov/nucleotide/KY400099.1?report=genbank&log$=nucltop&blast_rank=1&RID=BWC3VWHB01R) | 928 | 99.80% |
| 71 | Jul | 5CP | OM236804 | [*Phaeosphaeria fuckelii*](https://blast.ncbi.nlm.nih.gov/Blast.cgi) | [MZ396933.1](https://www.ncbi.nlm.nih.gov/nucleotide/MZ396933.1?report=genbank&log$=nucltop&blast_rank=1&RID=EK2MN86F013) | 950 | 99.62% |
| 72 | Jul | 5CP | OM236805 | Fungal sp. | [MW603421.1](https://www.ncbi.nlm.nih.gov/nucleotide/MW603421.1?report=genbank&log$=nucltop&blast_rank=1&RID=BWCAAWVC013) | 931 | 99.61% |
|  |  |  |  | [*Cladosporium anthropophilum*](https://blast.ncbi.nlm.nih.gov/Blast.cgi) | [MK111497.1](https://www.ncbi.nlm.nih.gov/nucleotide/MK111497.1?report=genbank&log$=nucltop&blast_rank=3&RID=BWCAAWVC013) | 928 | 99.41% |
| 1 | Aug | PS | OM236806 | [*Epicoccum* sp.](https://blast.ncbi.nlm.nih.gov/Blast.cgi) | KP721574.1 | 1140 | 98.84% |
|  |  |  |  | [*Epicoccum nigrum*](https://blast.ncbi.nlm.nih.gov/Blast.cgi) | HM055593.1 | 920 | 98.84% |
| 2 | Aug | PS | OM236807 | [*Cladosporium anthropophilum*](https://blast.ncbi.nlm.nih.gov/Blast.cgi) | [MT508803.1](https://www.ncbi.nlm.nih.gov/nucleotide/MT508803.1?report=genbank&log$=nucltop&blast_rank=1&RID=YNVT9KED013) | 909 | 100.00% |
| 3 | Aug | PS | OM236808 | [*Alternaria alternata*](https://blast.ncbi.nlm.nih.gov/Blast.cgi) | [MN249500.1](https://www.ncbi.nlm.nih.gov/nucleotide/MN249500.1?report=genbank&log$=nucltop&blast_rank=1&RID=YNVZVCD2013) | 939 | 100.00% |
| 4 | Aug | PS | OM236809 | [*Cladosporium oxysporum*](https://blast.ncbi.nlm.nih.gov/Blast.cgi) | [MH511102.1](https://www.ncbi.nlm.nih.gov/nucleotide/MH511102.1?report=genbank&log$=nucltop&blast_rank=1&RID=YNW5D1BD016) | 713 | 100.00% |
| 5 | Aug | FDO | OM236810 | [*Cladosporium* sp.](https://blast.ncbi.nlm.nih.gov/Blast.cgi) | [KP720581.1](https://www.ncbi.nlm.nih.gov/nucleotide/KP720581.1?report=genbank&log$=nucltop&blast_rank=1&RID=YNWBMZP2016) | 652 | 100.00% |
|  |  |  |  | [*Cladosporium tenuissimum*](https://blast.ncbi.nlm.nih.gov/Blast.cgi) | [MT508793.1](https://www.ncbi.nlm.nih.gov/nucleotide/MT508793.1?report=genbank&log$=nucltop&blast_rank=2&RID=YNWBMZP2016) | 649 | 100.00% |
| 6 | Aug | FDO | OM236811 | *Alternaria* sp. | [MT557238.1](https://www.ncbi.nlm.nih.gov/nucleotide/MT557238.1?report=genbank&log$=nucltop&blast_rank=1&RID=YNWGM2K3013) | 887 | 100.00% |
|  |  |  |  | [*Alternaria alternata*](https://blast.ncbi.nlm.nih.gov/Blast.cgi) | [MK659949.1](https://www.ncbi.nlm.nih.gov/nucleotide/MK659949.1?report=genbank&log$=nucltop&blast_rank=3&RID=YNWGM2K3013) | 887 | 100.00% |
| 7 | Aug | 5CP | OM236812 | [*Penicillium charlesii*](https://blast.ncbi.nlm.nih.gov/Blast.cgi) | MT309660.1 | 963 | 98.72% |
| 8 | Aug | 5CO | OM236813 | [*Didymella macrostoma*](https://blast.ncbi.nlm.nih.gov/Blast.cgi) | [MN944409.1](https://www.ncbi.nlm.nih.gov/nucleotide/MN944409.1?report=genbank&log$=nucltop&blast_rank=1&RID=EGJ35YJ0016) | 845 | 99.15% |
| 9 | Aug | 3CP | OM236814 | [*Curvularia hawaiiensis*](https://blast.ncbi.nlm.nih.gov/Blast.cgi) | [MT065820.1](https://www.ncbi.nlm.nih.gov/nucleotide/MT065820.1?report=genbank&log$=nucltop&blast_rank=1&RID=YNWT4NN1013) | 813 | 100.00% |
| 10 | Aug | 3CP | OM236815 | [*Didymella macrostoma*](https://blast.ncbi.nlm.nih.gov/Blast.cgi) | [MN944409.1](https://www.ncbi.nlm.nih.gov/nucleotide/MN944409.1?report=genbank&log$=nucltop&blast_rank=1&RID=YNWYNRZ9013) | 870 | 100.00% |
| 11 | Aug | CDP | OM236816 | [*Cladosporium halotolerans*](https://blast.ncbi.nlm.nih.gov/Blast.cgi) | [MK762599.1](https://www.ncbi.nlm.nih.gov/nucleotide/MK762599.1?report=genbank&log$=nucltop&blast_rank=1&RID=EG6805CY013) | 953 | 99.62% |
| 12 | Aug | CDP | OM236817 | [*Filobasidium magnum*](https://blast.ncbi.nlm.nih.gov/Blast.cgi) | MK226274.1 | 1070 | 99.32% |
| 13 | Aug | CDP | OM236818 | [Uncultured *Filobasidium*](https://blast.ncbi.nlm.nih.gov/Blast.cgi) | [MK407305.1](https://www.ncbi.nlm.nih.gov/nucleotide/MK407305.1?report=genbank&log$=nucltop&blast_rank=1&RID=EG6A04HZ013) | 510 | 86.93% |
|  |  |  |  | [*Filobasidium magnum*](https://blast.ncbi.nlm.nih.gov/Blast.cgi) | [MN848545.1](https://www.ncbi.nlm.nih.gov/nucleotide/MN848545.1?report=genbank&log$=nucltop&blast_rank=3&RID=EG6A04HZ013) | 505 | 86.71% |
| 14 | Aug | CDP | OM236819 | *Hanseniaspora opuntiae* | KF728809.1 | 1099 | 94.09% |
| 15 | Aug | 3CO | OM236820 | *Filobasidium magnum* | MN244430.1 | 989 | 99.09% |
| 16 | Aug | 3CO | OM236821 | [*Epicoccum nigrum*](https://blast.ncbi.nlm.nih.gov/Blast.cgi) | MN088703.1 | 918 | 99.22% |
| 17 | Aug | 3CO | OM236822 | *Alternaria* sp. | KP027305.1 | 2265 | 89.77% |
|  |  |  |  | *Alternaria alternata* | JQ080319.1 | 2416 | 90.08% |
| 18 | Aug | CDO | OM236823 | *Alternaria* sp. | KP027305.1 | 1695 | 90.94% |
|  |  |  |  | *Alternaria alternata* | MF422132.1 | 968 | 99.26% |
| 19 | Aug | CDO | OM236824 | *Aspergillus niger* | [MT645496.1](https://www.ncbi.nlm.nih.gov/nucleotide/MT645496.1?report=genbank&log$=nucltop&blast_rank=1&RID=EGJW7KPC016) | 579 | 97.35% |
| 20 | Aug | PS | OM236825 | *Cladosporium cladosporioides* | KF134535.1 | 911 | 98.83% |
| 21 | Aug | PS | OM236826 | *Cladosporium* sp. | KY827345.1 | 815 | 98.49% |
|  |  |  |  | *Cladosporium tenuissimum* | MT508793.1 | 809 | 98.27% |
| 22 | Aug | PS | OM236827 | Fungal sp. | KY775765.1 | 719 | 97.61% |
|  |  |  |  | *Zygosporium oscheoides* | MH861194.1 | 702 | 97.79% |
| 23 | Aug | PS | OM236828 | Fungal sp. | KY775765.1 | 719 | 97.61% |
|  |  |  |  | *Zygosporium oscheoides* | MH861194.1 | 702 | 97.79% |
| 24 | Aug | FDO | OM236829 | [*Phanerochaete sordida*](https://blast.ncbi.nlm.nih.gov/Blast.cgi) | MK269175.1 | 1081 | 100.00% |
| 25 | Aug | FDO | OM236830 | *Fomitopsis ostreiformis* | KY449363.1 | 981 | 99.08% |
| 26 | Aug | FDO | OM236831 | *Coriolopsis trogii* | KY234237.1 | 1640 | 99.34% |
| 27 | Aug | 3CO | OM236832 | *Neurospora tetrasperma* | MN511320.1 | 917 | 99.80% |
| 28 | Aug | CDO | OM236833 | [*Neurospora tetrasperma*](https://blast.ncbi.nlm.nih.gov/Blast.cgi) | MT990555.1 | 972 | 99.81% |
| 29 | Aug | CDP | OM236834 | *Phanerochaete chrysosporium* | KP998195.1 | 2898 | 93.48% |
| 30 | Aug | 5CO | OM236835 | *Coelastrella* sp. | MH176127.1 | 1134 | 97.73% |
| 31 | Aug | 5CP | OM236836 | [*Wallemia mellicola*](https://blast.ncbi.nlm.nih.gov/Blast.cgi) | [KX911856.1](https://www.ncbi.nlm.nih.gov/nucleotide/KX911856.1?report=genbank&log$=nucltop&blast_rank=1&RID=EUFT1342016) | 1362 | 99.08% |
| 32 | Aug | 5CP | OM236837 | [*Wallemia mellicola*](https://blast.ncbi.nlm.nih.gov/Blast.cgi) | [KX911856.1](https://www.ncbi.nlm.nih.gov/nucleotide/KX911856.1?report=genbank&log$=nucltop&blast_rank=1&RID=EUG2BY7K013) | 1373 | 99.34% |
| 33 | Aug | 3CO | OM236838 | [*Xylariaceae* sp.](https://blast.ncbi.nlm.nih.gov/Blast.cgi) | [MT557099.1](https://www.ncbi.nlm.nih.gov/nucleotide/MT557099.1?report=genbank&log$=nucltop&blast_rank=1&RID=EGK27HFF016) | 765 | 99.29% |
|  |  |  |  | *Neurospora tetrasperma* | [MH854922.1](https://www.ncbi.nlm.nih.gov/nucleotide/MH854922.1?report=genbank&log$=nucltop&blast_rank=2&RID=EGK27HFF016) | 765 | 99.29% |
| 34 | Aug | CDP | OM236839 | *Phanerochaete chrysosporium* | KP998195.1 | 2898 | 93.48% |
| 35 | Aug | CDP | OM236840 | [*Coprinopsis atramentaria*](https://blast.ncbi.nlm.nih.gov/Blast.cgi) | MZ151377.1 | 1205 | 99.55% |
| 36 | Aug | CDP | OM236841 | *Gloeophyllum trabeum* | KJ995949.1 | 979 | 97.56% |
| 37 | Aug | CDP | OM236842 | *Gloeophyllum trabeum* | JX501307.1 | 1461 | 99.32% |
| 38 | Aug | CDO | OM236843 | [*Neurospora tetrasperma*](https://blast.ncbi.nlm.nih.gov/Blast.cgi) | [MN511320.1](https://www.ncbi.nlm.nih.gov/nucleotide/MN511320.1?report=genbank&log$=nucltop&blast_rank=1&RID=EUG8PVTM013) | 1062 | 99.32% |
| 39 | Aug | CDO | OM236844 | [*Neurospora tetrasperma*](https://blast.ncbi.nlm.nih.gov/Blast.cgi) | [MN511320.1](https://www.ncbi.nlm.nih.gov/nucleotide/MN511320.1?report=genbank&log$=nucltop&blast_rank=1&RID=EUGBK30Y016) | 1051 | 98.98% |
| 1 | Sep | FDP | OM236845 | *Cladosporium funga* | AF413093.1 | 220 | 95.00% |
|  |  |  |  | *Cladosporium cladosporioides* | KP101204.1 | 219 | 95.00% |
| 2 | Sep | FDP | OM236846 | *Cladosporium anthropophilum* | MT508803.1 | 723 | 100.00% |
| 3 | Sep | FDP | OM236847 | [*Aspergillus* sp.](https://blast.ncbi.nlm.nih.gov/Blast.cgi) | MN634437.1 | 1029 | 99.82% |
| 4 | Sep | FDP | OM236848 | *Alternaria compacta* | [KU323573.1](https://www.ncbi.nlm.nih.gov/nucleotide/KU323573.1?report=genbank&log$=nucltop&blast_rank=1&RID=EGKWSF8R016) | 3001 | 99.16% |
| 5 | Sep | FDP | OM236849 | *Alternaria* sp. | MT557073.1 | 848 | 100.00% |
|  |  |  |  | *Alternaria tenuissima* | MK748314.1 | 848 | 100.00% |
| 6 | Sep | FDP | OM236850 | *Cladosporium ramotenellum* | MT529231.1 | 508 | 99.29% |
| 7 | Sep | FDP | OM236851 | [*Cladosporium cladosporioides*](https://blast.ncbi.nlm.nih.gov/Blast.cgi) | MH270583.1 | 928 | 99.04% |
| 8 | Sep | FDP | OM236852 | [*Alternaria* sp.](https://blast.ncbi.nlm.nih.gov/Blast.cgi) | MH270583.1 | 985 | 99.63% |
|  |  |  |  | [*Alternaria compacta*](https://blast.ncbi.nlm.nih.gov/Blast.cgi) | KU323573.1 | 979 | 99.81% |
| 9 | Sep | FDP | OM236853 | *Cladosporium anthropophilum* | MT508803.1 | 723 | 100.00% |
| 10 | Sep | FDP | OM236854 | [*Cladosporium cladosporioides*](https://blast.ncbi.nlm.nih.gov/Blast.cgi) | [MH270583.1](https://www.ncbi.nlm.nih.gov/nucleotide/MH270583.1?report=genbank&log$=nucltop&blast_rank=1&RID=EJ8HX8KZ016) | 894 | 97.88% |
| 11 | Sep | CDP | OM236855 | *Cladosporium cladosporioides* | MG840676.1 | 327 | 99.45% |
| 12 | Sep | CDP | OM236856 | *Cladosporium* sp. | MT645909.1 | 435 | 100.00% |
|  |  |  |  | *Cladosporium halotolerans* | MT635287.1 | 435 | 100.00% |
| 13 | Sep | CDP | OM236857 | *Cladosporium halotolerans* | [MT635287.1](https://www.ncbi.nlm.nih.gov/nucleotide/MT635287.1?report=genbank&log$=nucltop&blast_rank=1&RID=EUGMYB71013) | 953 | 98.70% |
| 14 | Sep | CDP | OM236858 | [*Aspergillus niger*](https://blast.ncbi.nlm.nih.gov/Blast.cgi) | MT645496.1 | 612 | 99.12% |
| 15 | Sep | CDP | OM236859 | *Cladosporium cladosporioides* | MH270592.1 | 713 | 99.49% |
| 16 | Sep | CDP | OM236860 | *Cladosporium anthropophilum* | MT508803.1 | 723 | 100.00% |
| 17 | Sep | CDO | OM236861 | [*Cladosporium* sp.](https://blast.ncbi.nlm.nih.gov/Blast.cgi) | [KF159973.1](https://www.ncbi.nlm.nih.gov/nucleotide/KF159973.1?report=genbank&log$=nucltop&blast_rank=1&RID=F7PCSYC4016) | 929 | 99.23% |
|  |  |  |  | [*Cladosporium cladosporioides*](https://blast.ncbi.nlm.nih.gov/Blast.cgi) | [MT729833.1](https://www.ncbi.nlm.nih.gov/nucleotide/MT729833.1?report=genbank&log$=nucltop&blast_rank=3&RID=E054F449013) | 928 | 99.42% |
| 18 | Sep | CDO | OM236862 | [*Aspergillus flavus*](https://blast.ncbi.nlm.nih.gov/Blast.cgi) | [AJ874128.1](https://www.ncbi.nlm.nih.gov/nucleotide/AJ874128.1?report=genbank&log$=nucltop&blast_rank=1&RID=EUGV28JB01R) | 957 | 96.20% |
| 19 | Sep | CDO | OM236863 | *Cladosporium ramotenellum* | MT529231.1 | 508 | 99.29% |
| 20 | Sep | CDO | OM236864 | *Cercospora* sp. | MT626618.1 | 453 | 100.00% |
| 21 | Sep | CDO | OM236865 | *Fusarium incarnatum* | [MG808373.1](https://www.ncbi.nlm.nih.gov/nucleotide/MG808373.1?report=genbank&log$=nucltop&blast_rank=1&RID=EUJ8G45901R) | 931 | 99.22% |
| 22 | Sep | CDO | OM236866 | *Cladosporium ramotenellum* | MT529231.1 | 508 | 99.29% |
| 23 | Sep | 5CP | OM236867 | [*Fusarium incarnatum*](https://blast.ncbi.nlm.nih.gov/Blast.cgi) | [MZ267023.1](https://www.ncbi.nlm.nih.gov/nucleotide/MZ267023.1?report=genbank&log$=nucltop&blast_rank=1&RID=E058GU3B013) | 926 | 100.00% |
| 24 | Sep | 5CP | OM236868 | [*Fusarium incarnatum*](https://blast.ncbi.nlm.nih.gov/Blast.cgi) | [MZ267023.1](https://www.ncbi.nlm.nih.gov/nucleotide/MZ267023.1?report=genbank&log$=nucltop&blast_rank=1&RID=EJA6J417013) | 893 | 98.80% |
| 25 | Sep | 5CP | OM236869 | [*Alternaria tenuissima*](https://blast.ncbi.nlm.nih.gov/Blast.cgi) | [MW008898.1](https://www.ncbi.nlm.nih.gov/nucleotide/MW008898.1?report=genbank&log$=nucltop&blast_rank=1&RID=E05CPUYK01R) | 992 | 100.00% |
| 26 | Sep | 5CP | OM236870 | *Neurospora tetrasperma* | [MN511320.1](https://www.ncbi.nlm.nih.gov/nucleotide/MN511320.1?report=genbank&log$=nucltop&blast_rank=1&RID=EUH7KEXR013) | 1051 | 98.98% |
| 27 | Sep | 5CP | OM236871 | *Alternaria alternata* | MN615420.1 | 813 | 100.00% |
| 28 | Sep | 5CP | OM236872 | *Cladosporium anthropophilum* | MT508803.1 | 723 | 100.00% |
| 29 | Sep | 5CP | OM236873 | [*Epicoccum* sp.](https://blast.ncbi.nlm.nih.gov/Blast.cgi) | [KP721574.1](https://www.ncbi.nlm.nih.gov/nucleotide/KP721574.1?report=genbank&log$=nucltop&blast_rank=1&RID=E05EZ5FB013) | 1168 | 99.05% |
|  |  |  |  | [*Epicoccum nigrum*](https://blast.ncbi.nlm.nih.gov/Blast.cgi) | [HM055593.1](https://www.ncbi.nlm.nih.gov/nucleotide/HM055593.1?report=genbank&log$=nucltop&blast_rank=2&RID=E05EZ5FB013) | 928 | 99.04% |
| 30 | Sep | 5CP | OM236874 | [*Cladosporium cladosporioides*](https://blast.ncbi.nlm.nih.gov/Blast.cgi) | [MG820070.1](https://www.ncbi.nlm.nih.gov/nucleotide/MG820070.1?report=genbank&log$=nucltop&blast_rank=1&RID=E05HXDYZ016) | 935 | 99.42% |
| 31 | Sep | 5CP | OM236875 | *Cercospora* sp. | MT626618.1 | 652 | 99.17% |
| 32 | Sep | 5CP | OM236876 | *Alternaria* sp. | MT557073.1 | 924 | 99.80% |
|  |  |  |  | *Alternaria tenuissima* | MK748314.1 | 924 | 99.80% |
| 33 | Sep | 5CP | OM236877 | [*Cladosporium cladosporioides*](https://blast.ncbi.nlm.nih.gov/Blast.cgi) | [KR709050.1](https://www.ncbi.nlm.nih.gov/nucleotide/KR709050.1?report=genbank&log$=nucltop&blast_rank=1&RID=E05M7Z6V013) | 933 | 99.23% |
| 34 | Sep | 5CP | OM236878 | [*Cladosporium* sp.](https://blast.ncbi.nlm.nih.gov/Blast.cgi) | [MN634593.1](https://www.ncbi.nlm.nih.gov/nucleotide/MN634593.1?report=genbank&log$=nucltop&blast_rank=1&RID=E05PCA74016) | 928 | 99.22% |
|  |  |  |  | [*Cladosporium cladosporioides*](https://blast.ncbi.nlm.nih.gov/Blast.cgi) | [MH270583.1](https://www.ncbi.nlm.nih.gov/nucleotide/MH270583.1?report=genbank&log$=nucltop&blast_rank=2&RID=E05PCA74016) | 928 | 99.04% |
| 35 | Sep | 5CP | OM236879 | [*Cladosporium cladosporioides*](https://blast.ncbi.nlm.nih.gov/Blast.cgi) | [MF688676.1](https://www.ncbi.nlm.nih.gov/nucleotide/MF688676.1?report=genbank&log$=nucltop&blast_rank=1&RID=E05TAEV8013) | 717 | 98.52% |
| 36 | Sep | 5CP | OM236880 | [*Cladosporium cladosporioides*](https://blast.ncbi.nlm.nih.gov/Blast.cgi) | [KR709050.1](https://www.ncbi.nlm.nih.gov/nucleotide/KR709050.1?report=genbank&log$=nucltop&blast_rank=1&RID=E05VMPU4016) | 935 | 99.23% |
| 37 | Sep | 5CP | OM236881 | *Alternaria alternata* | MN615420.1 | 833 | 100.00% |
| 38 | Sep | 3CP | OM236882 | [*Cladosporium* sp.](https://blast.ncbi.nlm.nih.gov/Blast.cgi) | [MG746380.1](https://www.ncbi.nlm.nih.gov/nucleotide/MG746380.1?report=genbank&log$=nucltop&blast_rank=1&RID=E05Y5TFU013) | 929 | 99.61% |
|  |  |  |  | [*Cladosporium cladosporioides*](https://blast.ncbi.nlm.nih.gov/Blast.cgi) | [MT367262.1](https://www.ncbi.nlm.nih.gov/nucleotide/MT367262.1?report=genbank&log$=nucltop&blast_rank=2&RID=E05Y5TFU013) | 928 | 99.80% |
| 39 | Sep | 3CP | OM236883 | *Alternaria alternata* | MW361306.1 | 880 | 99.79% |
| 40 | Sep | 3CP | OM236884 | *Alternaria alternata* | MW361306.1 | 913 | 100.00% |
| 41 | Sep | 3CP | OM236885 | *Cladosporium cladosporioides* | KY412173.1 | 555 | 98.12% |
| 42 | Sep | 3CP | OM236886 | [*Cladosporium* sp.](https://blast.ncbi.nlm.nih.gov/Blast.cgi) | [KT826661.1](https://www.ncbi.nlm.nih.gov/nucleotide/KT826661.1?report=genbank&log$=nucltop&blast_rank=1&RID=E0615UWX013) | 946 | 99.24% |
|  |  |  |  | [*Cladosporium asperulatum*](https://blast.ncbi.nlm.nih.gov/Blast.cgi) | [MG647842.1](https://www.ncbi.nlm.nih.gov/nucleotide/MG647842.1?report=genbank&log$=nucltop&blast_rank=2&RID=E0615UWX013) | 944 | 99.42% |
| 43 | Sep | 3CP | OM236887 | *Epicoccum nigrum* | MN089646.1 | 719 | 100.00% |
| 44 | Sep | 3CP | OM236888 | [*Cladosporium oxysporum*](https://blast.ncbi.nlm.nih.gov/Blast.cgi) | [KY400086.1](https://www.ncbi.nlm.nih.gov/nucleotide/KY400086.1?report=genbank&log$=nucltop&blast_rank=1&RID=E0647GHT013) | 915 | 98.46% |
| 45 | Sep | 3CP | OM236889 | *Neurospora tetrasperma* | MN511320.1 | 878 | 100.00% |
| 46 | Sep | 3CP | OM236890 | [*Epicoccum nigrum*](https://blast.ncbi.nlm.nih.gov/Blast.cgi) | [MN089646.1](https://www.ncbi.nlm.nih.gov/nucleotide/MN089646.1?report=genbank&log$=nucltop&blast_rank=1&RID=E06652Z9013) | 771 | 100.00% |
| 47 | Sep | 3CP | OM236891 | *Epicoccum nigrum* | MN089646.1 | 771 | 100.00% |
| 48 | Sep | 3CP | OM236892 | [*Alternaria* sp.](https://blast.ncbi.nlm.nih.gov/Blast.cgi) | [MW009042.1](https://www.ncbi.nlm.nih.gov/nucleotide/MW009042.1?report=genbank&log$=nucltop&blast_rank=1&RID=E0689RJS013) | 989 | 99.81% |
|  |  |  |  | [*Alternaria angustiovoidea*](https://blast.ncbi.nlm.nih.gov/Blast.cgi) | [MW008876.1](https://www.ncbi.nlm.nih.gov/nucleotide/MW008876.1?report=genbank&log$=nucltop&blast_rank=2&RID=E0689RJS013) | 983 | 99.81% |
| 49 | Sep | 3CP | OM236893 | [*Cladosporium cladosporioides*](https://blast.ncbi.nlm.nih.gov/Blast.cgi) | [MH345929.1](https://www.ncbi.nlm.nih.gov/nucleotide/MH345929.1?report=genbank&log$=nucltop&blast_rank=1&RID=E06B2617013) | 935 | 99.42% |
| 50 | Sep | 3CP | OM236894 | [*Epicoccum* sp.](https://blast.ncbi.nlm.nih.gov/Blast.cgi) | [KP721574.1](https://www.ncbi.nlm.nih.gov/nucleotide/KP721574.1?report=genbank&log$=nucltop&blast_rank=1&RID=E06D3MW9016) | 1163 | 98.67% |
|  |  |  |  | [*Epicoccum nigrum*](https://blast.ncbi.nlm.nih.gov/Blast.cgi) | [GU395519.1](https://www.ncbi.nlm.nih.gov/nucleotide/GU395519.1?report=genbank&log$=nucltop&blast_rank=2&RID=E06D3MW9016) | 924 | 99.22% |
| 51 | Sep | 3CP | OM236895 | [*Cladosporium anthropophilum*](https://blast.ncbi.nlm.nih.gov/Blast.cgi) | [MT508803.1](https://www.ncbi.nlm.nih.gov/nucleotide/MT508803.1?report=genbank&log$=nucltop&blast_rank=1&RID=E06FNMAZ013) | 647 | 99.72% |
| 52 | Sep | 3CP | OM236896 | [*Epicoccum nigrum*](https://blast.ncbi.nlm.nih.gov/Blast.cgi) | [MN089646.1](https://www.ncbi.nlm.nih.gov/nucleotide/MN089646.1?report=genbank&log$=nucltop&blast_rank=1&RID=E06HEHYX013) | 638 | 99.71% |
| 53 | Sep | 3CP | OM236897 | [*Cladosporium cladosporioides*](https://blast.ncbi.nlm.nih.gov/Blast.cgi) | [MW556429.1](https://www.ncbi.nlm.nih.gov/nucleotide/MW556429.1?report=genbank&log$=nucltop&blast_rank=1&RID=E06K6EA5013) | 944 | 99.81% |
| 54 | Sep | 3CP | OM236898 | [*Cladosporium anthropophilum*](https://blast.ncbi.nlm.nih.gov/Blast.cgi) | [MT508803.1](https://www.ncbi.nlm.nih.gov/nucleotide/MT508803.1?report=genbank&log$=nucltop&blast_rank=1&RID=E06N15YS013) | 852 | 100.00% |
| 55 | Sep | FDO | OM236899 | [*Alternaria* sp.](https://blast.ncbi.nlm.nih.gov/Blast.cgi) | [MW009028.1](https://www.ncbi.nlm.nih.gov/nucleotide/MW009028.1?report=genbank&log$=nucltop&blast_rank=1&RID=E06T3807016) | 977 | 100.00% |
|  |  |  |  | [*Alternaria tamaricis*](https://blast.ncbi.nlm.nih.gov/Blast.cgi) | [MW009027.1](https://www.ncbi.nlm.nih.gov/nucleotide/MW009027.1?report=genbank&log$=nucltop&blast_rank=2&RID=E06T3807016) | 974 | 100.00% |
| 56 | Sep | FDO | OM236900 | [*Epicoccum nigrum*](https://blast.ncbi.nlm.nih.gov/Blast.cgi) | [MN089646.1](https://www.ncbi.nlm.nih.gov/nucleotide/MN089646.1?report=genbank&log$=nucltop&blast_rank=1&RID=E06WKH34013) | 867 | 99.79% |
| 57 | Sep | FDO | OM236901 | [Uncultured fungus](https://blast.ncbi.nlm.nih.gov/Blast.cgi) | [KX515960.1](https://www.ncbi.nlm.nih.gov/nucleotide/KX515960.1?report=genbank&log$=nucltop&blast_rank=1&RID=E06YP3KF013) | 963 | 98.89% |
|  |  |  |  | [Uncultured *Ochrocladosporium*](https://blast.ncbi.nlm.nih.gov/Blast.cgi) | [JX317417.1](https://www.ncbi.nlm.nih.gov/nucleotide/JX317417.1?report=genbank&log$=nucltop&blast_rank=3&RID=E06YP3KF013) | 942 | 98.16% |
| 58 | Sep | FDO | OM236902 | [Fungal sp.](https://blast.ncbi.nlm.nih.gov/Blast.cgi) | [MW603421.1](https://www.ncbi.nlm.nih.gov/nucleotide/MW603421.1?report=genbank&log$=nucltop&blast_rank=1&RID=E07S2979016) | 946 | 99.43% |
|  |  |  |  | [*Cladosporium cladosporioides*](https://blast.ncbi.nlm.nih.gov/Blast.cgi) | [MH345929.1](https://www.ncbi.nlm.nih.gov/nucleotide/MH345929.1?report=genbank&log$=nucltop&blast_rank=4&RID=E07S2979016) | 937 | 99.42% |
| 59 | Sep | FDO | OM236903 | [*Cladosporium ramotenellum*](https://blast.ncbi.nlm.nih.gov/Blast.cgi) | [MT529231.1](https://www.ncbi.nlm.nih.gov/nucleotide/MT529231.1?report=genbank&log$=nucltop&blast_rank=1&RID=E07XTK8B013) | 676 | 100.00% |
| 60 | Sep | FDO | OM236904 | *Aspergillus flavus* | [AJ874128.1](https://www.ncbi.nlm.nih.gov/nucleotide/AJ874128.1?report=genbank&log$=nucltop&blast_rank=1&RID=EUHE7E3M01R) | 992 | 96.86% |
| 61 | Sep | FDO | OM236905 | [*Cladosporium tenuissimum*](https://blast.ncbi.nlm.nih.gov/Blast.cgi) | [MT508793.1](https://www.ncbi.nlm.nih.gov/nucleotide/MT508793.1?report=genbank&log$=nucltop&blast_rank=1&RID=E08YWKUP013) | 793 | 100.00% |
| 62 | Sep | FDO | OM236906 | [*Cladosporium cladosporioides*](https://blast.ncbi.nlm.nih.gov/Blast.cgi) | [KX639814.1](https://www.ncbi.nlm.nih.gov/nucleotide/KX639814.1?report=genbank&log$=nucltop&blast_rank=1&RID=E0B062XC013) | 924 | 99.61% |
| 63 | Sep | 5CO | OM236907 | [*Cladosporium delicatulum*](https://blast.ncbi.nlm.nih.gov/Blast.cgi) | [MN644691.1](https://www.ncbi.nlm.nih.gov/nucleotide/MN644691.1?report=genbank&log$=nucltop&blast_rank=1&RID=E0BM8WBK013) | 863 | 99.79% |
| 64 | Sep | 5CO | OM236908 | [*Cladosporium oxysporum*](https://blast.ncbi.nlm.nih.gov/Blast.cgi) | [MT569986.1](https://www.ncbi.nlm.nih.gov/nucleotide/MT569986.1?report=genbank&log$=nucltop&blast_rank=1&RID=E0CN6Y1U013) | 606 | 99.40% |
| 65 | Sep | 5CO | OM236909 | [*Cladosporium tenuissimum*](https://blast.ncbi.nlm.nih.gov/Blast.cgi) | [MT508793.1](https://www.ncbi.nlm.nih.gov/nucleotide/MT508793.1?report=genbank&log$=nucltop&blast_rank=1&RID=E0CZSBB501R) | 726 | 100.00% |
| 66 | Sep | 5CO | OM236910 | [*Epicoccum nigrum*](https://blast.ncbi.nlm.nih.gov/Blast.cgi) | [KF986447.1](https://www.ncbi.nlm.nih.gov/nucleotide/KF986447.1?report=genbank&log$=nucltop&blast_rank=1&RID=E0D2K5Z601R) | 869 | 99.79% |
| 67 | Sep | 5CO | OM236911 | [*Stemphylium solani*](https://blast.ncbi.nlm.nih.gov/Blast.cgi) | [LC592373.1](https://www.ncbi.nlm.nih.gov/nucleotide/LC592373.1?report=genbank&log$=nucltop&blast_rank=1&RID=E0D7BBR3016) | 981 | 99.26% |
| 68 | Sep | 5CO | OM236912 | [*Alternaria tenuissima*](https://blast.ncbi.nlm.nih.gov/Blast.cgi) | [MZ160958.1](https://www.ncbi.nlm.nih.gov/nucleotide/MZ160958.1?report=genbank&log$=nucltop&blast_rank=1&RID=E0D96YCU016) | 981 | 99.45% |
| 69 | Sep | 5CO | OM236913 | [*Fusarium incarnatum*](https://blast.ncbi.nlm.nih.gov/Blast.cgi) | [MT447512.1](https://www.ncbi.nlm.nih.gov/nucleotide/MT447512.1?report=genbank&log$=nucltop&blast_rank=1&RID=EG23AY5Z013) | 929 | 99.42% |
| 70 | Sep | 5CO | OM236914 | [*Fusarium oxysporum*](https://blast.ncbi.nlm.nih.gov/Blast.cgi) | [MG136705.1](https://www.ncbi.nlm.nih.gov/nucleotide/MG136705.1?report=genbank&log$=nucltop&blast_rank=1&RID=E0DCFB9Y016) | 926 | 99.80% |
| 71 | Sep | 5CO | OM236915 | [*Alternaria solani*](https://blast.ncbi.nlm.nih.gov/Blast.cgi) | [MW009039.1](https://www.ncbi.nlm.nih.gov/nucleotide/MW009039.1?report=genbank&log$=nucltop&blast_rank=1&RID=E0DESWF9013) | 985 | 99.63% |
| 72 | Sep | 5CO | OM236916 | [*Epicoccum nigrum*](https://blast.ncbi.nlm.nih.gov/Blast.cgi) | [KF986447.1](https://www.ncbi.nlm.nih.gov/nucleotide/KF986447.1?report=genbank&log$=nucltop&blast_rank=1&RID=E0DH0DTW013) | 928 | 99.61% |
| 73 | Sep | 5CO | OM236917 | [*Dothideomycetes* sp.](https://blast.ncbi.nlm.nih.gov/Blast.cgi) | [KX909022.1](https://www.ncbi.nlm.nih.gov/nucleotide/KX909022.1?report=genbank&log$=nucltop&blast_rank=1&RID=E0DJUH2B016) | 843 | 97.57% |
|  |  |  |  | [*Epicoccum nigrum*](https://blast.ncbi.nlm.nih.gov/Blast.cgi) | [KM877476.1](https://www.ncbi.nlm.nih.gov/nucleotide/KM877476.1?report=genbank&log$=nucltop&blast_rank=3&RID=E0DJUH2B016) | 841 | 97.56% |
| 74 | Sep | 5CO | OM236918 | [*Pleosporales* sp.](https://blast.ncbi.nlm.nih.gov/Blast.cgi) | [MN486543.1](https://www.ncbi.nlm.nih.gov/nucleotide/MN486543.1?report=genbank&log$=nucltop&blast_rank=1&RID=E0DPJ3BG013) | 974 | 99.63% |
|  |  |  |  | [*Periconia byssoides*](https://blast.ncbi.nlm.nih.gov/Blast.cgi) | [MW081391.1](https://www.ncbi.nlm.nih.gov/nucleotide/MW081391.1?report=genbank&log$=nucltop&blast_rank=2&RID=E0DPJ3BG013) | 974 | 99.63% |
| 75 | Sep | 5CO | OM236919 | [*Alternaria angustiovoidea*](https://blast.ncbi.nlm.nih.gov/Blast.cgi) | [MW008997.1](https://www.ncbi.nlm.nih.gov/nucleotide/MW008997.1?report=genbank&log$=nucltop&blast_rank=1&RID=E0DTK4X9013) | 985 | 99.81% |
| 76 | Sep | 5CO | OM236920 | [Fungal sp.](https://blast.ncbi.nlm.nih.gov/Blast.cgi) | [MW603421.1](https://www.ncbi.nlm.nih.gov/nucleotide/MW603421.1?report=genbank&log$=nucltop&blast_rank=1&RID=EBD2TF7W01R) | 939 | 99.42% |
|  |  |  |  | [*Cladosporium tenuissimum*](https://blast.ncbi.nlm.nih.gov/Blast.cgi) | [KP689183.1](https://www.ncbi.nlm.nih.gov/nucleotide/KP689183.1?report=genbank&log$=nucltop&blast_rank=3&RID=EBD2TF7W01R) | 935 | 99.23% |
| 77 | Sep | 5CO | OM236921 | [*Cladosporium* sp.](https://blast.ncbi.nlm.nih.gov/Blast.cgi) | [MG572460.1](https://www.ncbi.nlm.nih.gov/nucleotide/MG572460.1?report=genbank&log$=nucltop&blast_rank=1&RID=EBD786G4016) | 867 | 99.79% |
|  |  |  |  | [*Cladosporium cladosporioides*](https://blast.ncbi.nlm.nih.gov/Blast.cgi) | [MW113408.1](https://www.ncbi.nlm.nih.gov/nucleotide/MW113408.1?report=genbank&log$=nucltop&blast_rank=2&RID=EBD786G4016) | 867 | 99.79% |
| 78 | Sep | 5CO | OM236922 | [Fungal sp.](https://blast.ncbi.nlm.nih.gov/Blast.cgi) | [MW603384.1](https://www.ncbi.nlm.nih.gov/nucleotide/MW603384.1?report=genbank&log$=nucltop&blast_rank=1&RID=EBDA295S016) | 942 | 100.00% |
|  |  |  |  | [*Cladosporium cladosporioides*](https://blast.ncbi.nlm.nih.gov/Blast.cgi) | [KY114882.1](https://www.ncbi.nlm.nih.gov/nucleotide/KY114882.1?report=genbank&log$=nucltop&blast_rank=2&RID=EBDA295S016) | 941 | 99.81% |
| 79 | Sep | 5CO | OM236923 | [*Cladosporium* sp.](https://blast.ncbi.nlm.nih.gov/Blast.cgi) | [MK111574.1](https://www.ncbi.nlm.nih.gov/nucleotide/MK111574.1?report=genbank&log$=nucltop&blast_rank=1&RID=EBDDK9VK013) | 939 | 99.61% |
|  |  |  |  | [*Cladosporium tenuissimum*](https://blast.ncbi.nlm.nih.gov/Blast.cgi) | [HM776419.1](https://www.ncbi.nlm.nih.gov/nucleotide/HM776419.1?report=genbank&log$=nucltop&blast_rank=2&RID=EBDDK9VK013) | 939 | 99.42% |
| 80 | Sep | 5CO | OM236924 | [*Cladosporium oxysporum*](https://blast.ncbi.nlm.nih.gov/Blast.cgi) | [MF135506.1](https://www.ncbi.nlm.nih.gov/nucleotide/MF135506.1?report=genbank&log$=nucltop&blast_rank=1&RID=EBDG74GT016) | 939 | 99.80% |
| 81 | Sep | 3CP | OM236925 | [*Alternaria porri*](https://blast.ncbi.nlm.nih.gov/Blast.cgi) | [MW008991.1](https://www.ncbi.nlm.nih.gov/nucleotide/MW008991.1?report=genbank&log$=nucltop&blast_rank=1&RID=EBDHZPKP016) | 983 | 99.63% |
|  |  |  |  | [*Alternaria alternata*](https://blast.ncbi.nlm.nih.gov/Blast.cgi) | [MN872482.1](https://www.ncbi.nlm.nih.gov/nucleotide/MN872482.1?report=genbank&log$=nucltop&blast_rank=2&RID=EBDHZPKP016) | 981 | 99.45% |
| 82 | Sep | 3CP | OM236926 | [*Alternaria solani*](https://blast.ncbi.nlm.nih.gov/Blast.cgi) | [MW009039.1](https://www.ncbi.nlm.nih.gov/nucleotide/MW009039.1?report=genbank&log$=nucltop&blast_rank=1&RID=EBDMZ3CP016) | 983 | 99.81% |
| 83 | Sep | 3CP | OM236927 | *Cladosporium cladosporioides* | [MT367212.1](https://www.ncbi.nlm.nih.gov/nucleotide/MT367212.1?report=genbank&log$=nucltop&blast_rank=1&RID=EUHMESYP01R) | 922 | 98.66% |
| 84 | Sep | 3CP | OM236928 | [*Cladosporium cladosporioides*](https://blast.ncbi.nlm.nih.gov/Blast.cgi) | [MT367212.1](https://www.ncbi.nlm.nih.gov/nucleotide/MT367212.1?report=genbank&log$=nucltop&blast_rank=1&RID=EBDR42HN013) | 939 | 99.61% |
| 85 | Sep | 3CP | OM236929 | [*Epicoccum nigrum*](https://blast.ncbi.nlm.nih.gov/Blast.cgi) | [KM877476.1](https://www.ncbi.nlm.nih.gov/nucleotide/KM877476.1?report=genbank&log$=nucltop&blast_rank=1&RID=EBDSYBAB016) | 933 | 100.00% |
| 86 | Sep | 3CP | OM236930 | [*Epicoccum nigrum*](https://blast.ncbi.nlm.nih.gov/Blast.cgi) | [KM877476.1](https://www.ncbi.nlm.nih.gov/nucleotide/KM877476.1?report=genbank&log$=nucltop&blast_rank=1&RID=EBDUS4SP013) | 933 | 100.00% |
| 87 | Sep | 3CP | OM236931 | [*Alternaria compacta*](https://blast.ncbi.nlm.nih.gov/Blast.cgi) | [MW008918.1](https://www.ncbi.nlm.nih.gov/nucleotide/MW008918.1?report=genbank&log$=nucltop&blast_rank=1&RID=EBDX4VK0016) | 983 | 99.81% |
| 88 | Sep | 3CP | OM236932 | [*Fusarium oxysporum*](https://blast.ncbi.nlm.nih.gov/Blast.cgi) | [MG136705.1](https://www.ncbi.nlm.nih.gov/nucleotide/MG136705.1?report=genbank&log$=nucltop&blast_rank=1&RID=EBDZHECW013) | 926 | 99.61% |
| 89 | Sep | 3CP | OM236933 | [*Cladosporium anthropophilum*](https://blast.ncbi.nlm.nih.gov/Blast.cgi) | [MT508803.1](https://www.ncbi.nlm.nih.gov/nucleotide/MT508803.1?report=genbank&log$=nucltop&blast_rank=1&RID=EBE1JSSB016) | 736 | 98.79% |
| 90 | Sep | LIB | OM236934 | [*Alternaria alternata*](https://blast.ncbi.nlm.nih.gov/Blast.cgi) | [MT093259.1](https://www.ncbi.nlm.nih.gov/nucleotide/MT093259.1?report=genbank&log$=nucltop&blast_rank=1&RID=EBE3APZ0013) | 976 | 99.63% |
| 91 | Sep | LIB | OM236935 | [*Alternaria* sp.](https://blast.ncbi.nlm.nih.gov/Blast.cgi) | [MK649974.1](https://www.ncbi.nlm.nih.gov/nucleotide/MK649974.1?report=genbank&log$=nucltop&blast_rank=1&RID=EBE57G5T016) | 989 | 99.81% |
| 92 | Sep | LIB | OM236936 | [*Epicoccum nigrum*](https://blast.ncbi.nlm.nih.gov/Blast.cgi) | [MN089646.1](https://www.ncbi.nlm.nih.gov/nucleotide/MN089646.1?report=genbank&log$=nucltop&blast_rank=1&RID=EBE7E306016) | 931 | 100.00% |
| 93 | Sep | LIB | OM236937 | [*Alternaria compacta*](https://blast.ncbi.nlm.nih.gov/Blast.cgi) | [MW008918.1](https://www.ncbi.nlm.nih.gov/nucleotide/MW008918.1?report=genbank&log$=nucltop&blast_rank=1&RID=EBE9UFG9013) | 983 | 100.00% |
| 94 | Sep | LIB | OM236938 | [*Alternaria* sp.](https://blast.ncbi.nlm.nih.gov/Blast.cgi) | [MW009042.1](https://www.ncbi.nlm.nih.gov/nucleotide/MW009042.1?report=genbank&log$=nucltop&blast_rank=1&RID=EBEBRTE7013) | 976 | 99.44% |
| 95 | Sep | LIB | OM236939 | [*Alternaria tenuissima*](https://blast.ncbi.nlm.nih.gov/Blast.cgi) | [MW009005.1](https://www.ncbi.nlm.nih.gov/nucleotide/MW009005.1?report=genbank&log$=nucltop&blast_rank=1&RID=EBEDXU06013) | 989 | 100.00% |
| 96 | Sep | LIB | OM236940 | [*Alternaria alternata*](https://blast.ncbi.nlm.nih.gov/Blast.cgi) | [MW008926.1](https://www.ncbi.nlm.nih.gov/nucleotide/MW008926.1?report=genbank&log$=nucltop&blast_rank=1&RID=EBEFWKJD013) | 976 | 100.00% |
| 97 | Sep | LIB | OM236941 | [*Alternaria alternata*](https://blast.ncbi.nlm.nih.gov/Blast.cgi) | [MW361306.1](https://www.ncbi.nlm.nih.gov/nucleotide/MW361306.1?report=genbank&log$=nucltop&blast_rank=1&RID=YP12MS65013) | 889 | 100.00% |
| 98 | Sep | LIB | OM236942 | [*Alternaria alternata*](https://blast.ncbi.nlm.nih.gov/Blast.cgi) | [MW361306.1](https://www.ncbi.nlm.nih.gov/nucleotide/MW361306.1?report=genbank&log$=nucltop&blast_rank=1&RID=YP9HMD58016) | 819 | 100.00% |
| 99 | Sep | LIB | OM236943 | [*Epicoccum nigrum*](https://blast.ncbi.nlm.nih.gov/Blast.cgi) | [MT557531.1](https://www.ncbi.nlm.nih.gov/nucleotide/MT557531.1?report=genbank&log$=nucltop&blast_rank=1&RID=YP9PH9J1016) | 769 | 100.00% |
| 100 | Sep | LIB | OM236944 | *Alternaria* sp. | [MT557238.1](https://www.ncbi.nlm.nih.gov/nucleotide/MT557238.1?report=genbank&log$=nucltop&blast_rank=1&RID=YP9WAT1Y013) | 898 | 100.00% |
|  |  |  |  | *Alternaria alternata* | [MT498268.1](https://www.ncbi.nlm.nih.gov/nucleotide/MT498268.1?report=genbank&log$=nucltop&blast_rank=3&RID=YP9WAT1Y013) | 898 | 100.00% |
| 101 | Sep | LIB | OM236945 | *Alternaria tenuissima* | [MK534905.1](https://www.ncbi.nlm.nih.gov/nucleotide/MK534905.1?report=genbank&log$=nucltop&blast_rank=1&RID=YPA1NKKU016) | 894 | 100.00% |
| 102 | Sep | LIB | OM236946 | *Alternaria alternata* | [MN249500.1](https://www.ncbi.nlm.nih.gov/nucleotide/MN249500.1?report=genbank&log$=nucltop&blast_rank=1&RID=YPA6GCDV013) | 915 | 100.00% |
| 103 | Sep | LIB | OM236947 | *Epicoccum nigrum* | [MN089646.1](https://www.ncbi.nlm.nih.gov/nucleotide/MN089646.1?report=genbank&log$=nucltop&blast_rank=1&RID=YPAD23ZF013) | 771 | 100.00% |
| 104 | Sep | LIB | OM236948 | *Alternaria alternata* | [MN249500.1](https://www.ncbi.nlm.nih.gov/nucleotide/MN249500.1?report=genbank&log$=nucltop&blast_rank=1&RID=YPAJDTEB013) | 939 | 100.00% |
| 105 | Sep | LIB | OM236949 | *Alternaria alternata* | [MN249500.1](https://www.ncbi.nlm.nih.gov/nucleotide/MN249500.1?report=genbank&log$=nucltop&blast_rank=1&RID=YPARJE35013) | 939 | 100.00% |
| 106 | Sep | LIB | OM236950 | [*Cladosporium oxysporum*](https://blast.ncbi.nlm.nih.gov/Blast.cgi) | [MF135506.1](https://www.ncbi.nlm.nih.gov/nucleotide/MF135506.1?report=genbank&log$=nucltop&blast_rank=1&RID=EBEJ1P97016) | 937 | 99.61% |
| 107 | Sep | LIB | OM236951 | *Bipolaris* sp. | [MT366783.1](https://www.ncbi.nlm.nih.gov/nucleotide/MT366783.1?report=genbank&log$=nucltop&blast_rank=1&RID=YPAWN8YA016) | 763 | 100.00% |
|  |  |  |  | [*Cochliobolus cymbopogonis*](https://blast.ncbi.nlm.nih.gov/Blast.cgi) | [JQ783057.1](https://www.ncbi.nlm.nih.gov/nucleotide/JQ783057.1?report=genbank&log$=nucltop&blast_rank=2&RID=YPAWN8YA016) | 750 | 99.52% |
| 108 | Sep | LIB | OM236952 | *Alternaria alternata* | [MW361306.1](https://www.ncbi.nlm.nih.gov/nucleotide/MW361306.1?report=genbank&log$=nucltop&blast_rank=1&RID=YPB1X24A016) | 891 | 100.00% |
| 109 | Sep | LIB | OM236953 | *Cladosporium oxysporum* | [MH511102.1](https://www.ncbi.nlm.nih.gov/nucleotide/MH511102.1?report=genbank&log$=nucltop&blast_rank=1&RID=YPB7EFAV013) | 662 | 100.00% |
| 110 | Sep | LIB | OM236954 | *Alternaria alternata* | [MN249500.1](https://www.ncbi.nlm.nih.gov/nucleotide/MN249500.1?report=genbank&log$=nucltop&blast_rank=1&RID=YPBBWA3A013) | 929 | 100.00% |
| 111 | Sep | LIB | OM236955 | *Cladosporium oxysporum* | [MH511102.1](https://www.ncbi.nlm.nih.gov/nucleotide/MH511102.1?report=genbank&log$=nucltop&blast_rank=1&RID=YPBH1M0X013) | 743 | 100.00% |
| 112 | Sep | LIB | OM236956 | *Cladosporium cladosporioides* | [MT598826.1](https://www.ncbi.nlm.nih.gov/nucleotide/MT598826.1?report=genbank&log$=nucltop&blast_rank=1&RID=YPBP836E016) | 808 | 99.77% |
| 113 | Sep | LIB | OM236957 | *Epicoccum nigrum* | [MN089646.1](https://www.ncbi.nlm.nih.gov/nucleotide/MN089646.1?report=genbank&log$=nucltop&blast_rank=1&RID=YPBVA7XR013) | 695 | 100.00% |
| 114 | Sep | LIB | OM236958 | *Vishniacozyma* sp. | [MT022574.1](https://www.ncbi.nlm.nih.gov/nucleotide/MT022574.1?report=genbank&log$=nucltop&blast_rank=1&RID=YPC99PFG016) | 808 | 100.00% |
|  |  |  |  | *Vishniacozyma taibaiensis* | [MN244420.1](https://www.ncbi.nlm.nih.gov/nucleotide/MN244420.1?report=genbank&log$=nucltop&blast_rank=3&RID=YPC99PFG016) | 808 | 100.00% |
| 115 | Sep | LIB | OM236959 | *Cladosporium* sp. | [KY096665.1](https://www.ncbi.nlm.nih.gov/nucleotide/KY096665.1?report=genbank&log$=nucltop&blast_rank=1&RID=YPCNKV09013) | 673 | 99.20% |
|  |  |  |  | [*Cladosporium ramotenellum*](https://blast.ncbi.nlm.nih.gov/Blast.cgi) | [MT529231.1](https://www.ncbi.nlm.nih.gov/nucleotide/MT529231.1?report=genbank&log$=nucltop&blast_rank=2&RID=YPCNKV09013) | 667 | 98.93% |
| 116 | Sep | LIB | OM236960 | *Alternaria alternata* | [MN548785.1](https://www.ncbi.nlm.nih.gov/nucleotide/MN548785.1?report=genbank&log$=nucltop&blast_rank=1&RID=YSWBEC0K016) | 898 | 99.80% |
| 117 | Sep | LIB | OM236961 | [*Cladosporium cladosporioides*](https://blast.ncbi.nlm.nih.gov/Blast.cgi) | [MT878604.1](https://www.ncbi.nlm.nih.gov/nucleotide/MT878604.1?report=genbank&log$=nucltop&blast_rank=1&RID=EBEMB1W7013) | 939 | 99.42% |
| 118 | Sep | LIB | OM236962 | *Alternaria alternata* | [MN249500.1](https://www.ncbi.nlm.nih.gov/nucleotide/MN249500.1?report=genbank&log$=nucltop&blast_rank=1&RID=YSWN9HBT013) | 891 | 100.00% |
| 119 | Sep | LIB | OM236963 | *Cladosporium anthropophilum* | [MT508803.1](https://www.ncbi.nlm.nih.gov/nucleotide/MT508803.1?report=genbank&log$=nucltop&blast_rank=1&RID=YSWUVSYY013) | 662 | 100.00% |
| 120 | Sep | LIB | OM236964 | [*Alternaria tenuissima*](https://blast.ncbi.nlm.nih.gov/Blast.cgi) | [MW008969.1](https://www.ncbi.nlm.nih.gov/nucleotide/MW008969.1?report=genbank&log$=nucltop&blast_rank=1&RID=EBEPFWRX013) | 979 | 99.81% |
| 121 | Sep | 5CO | OM236965 | *Fusarium incarnatum* | [MG808373.1](https://www.ncbi.nlm.nih.gov/nucleotide/MG808373.1?report=genbank&log$=nucltop&blast_rank=1&RID=EUHZP05T01R) | 920 | 98.29% |
| 122 | Sep | 5CO | OM236966 | [*Fusarium incarnatum*](https://blast.ncbi.nlm.nih.gov/Blast.cgi) | [MG808373.1](https://www.ncbi.nlm.nih.gov/nucleotide/MG808373.1?report=genbank&log$=nucltop&blast_rank=1&RID=EBESRSGG016) | 924 | 99.80% |
| 123 | Sep | 5CO | OM236967 | *Cladosporium anthropophilum* | [MT508803.1](https://www.ncbi.nlm.nih.gov/nucleotide/MT508803.1?report=genbank&log$=nucltop&blast_rank=1&RID=YSX0XKT3013) | 739 | 100.00% |
| 124 | Sep | 5CO | OM236968 | [*Cladosporium* sp.](https://blast.ncbi.nlm.nih.gov/Blast.cgi) | [MK640598.1](https://www.ncbi.nlm.nih.gov/nucleotide/MK640598.1?report=genbank&log$=nucltop&blast_rank=1&RID=EBEUM8GV016) | 743 | 99.03% |
|  |  |  |  | [*Cladosporium tenuissimum*](https://blast.ncbi.nlm.nih.gov/Blast.cgi) | [MN429197.1](https://www.ncbi.nlm.nih.gov/nucleotide/MN429197.1?report=genbank&log$=nucltop&blast_rank=2&RID=EBEUM8GV016) | 743 | 99.03% |
| 125 | Sep | 5CO | OM236969 | *Alternaria alternata* | [MW361306.1](https://www.ncbi.nlm.nih.gov/nucleotide/MW361306.1?report=genbank&log$=nucltop&blast_rank=1&RID=YSX8UVET016) | 822 | 100.00% |
| 126 | Sep | 5CO | OM236970 | *Epicoccum nigrum* | [MN089646.1](https://www.ncbi.nlm.nih.gov/nucleotide/MN089646.1?report=genbank&log$=nucltop&blast_rank=1&RID=YSXFXV21013) | 878 | 100.00% |
| 127 | Sep | 5CO | OM236971 | *Alternaria alternata* | [MN249500.1](https://www.ncbi.nlm.nih.gov/nucleotide/MN249500.1?report=genbank&log$=nucltop&blast_rank=1&RID=YSXT77JJ016) | 950 | 100.00% |
| 128 | Sep | 5CO | OM236972 | *Epicoccum nigrum* | [MN089646.1](https://www.ncbi.nlm.nih.gov/nucleotide/MN089646.1?report=genbank&log$=nucltop&blast_rank=1&RID=YSY18S0C016) | 865 | 100.00% |
| 129 | Sep | 5CO | OM236973 | *Alternaria alternata* | [MW361306.1](https://www.ncbi.nlm.nih.gov/nucleotide/MW361306.1?report=genbank&log$=nucltop&blast_rank=1&RID=YSY8J39P013) | 809 | 100.00% |
| 130 | Sep | 5CO | OM236974 | *Epicoccum nigrum* | [MN089646.1](https://www.ncbi.nlm.nih.gov/nucleotide/MN089646.1?report=genbank&log$=nucltop&blast_rank=1&RID=YSYM5GTW016) | 787 | 100.00% |
| 131 | Sep | 5CO | OM236975 | *Alternaria* sp. | [MT557073.1](https://www.ncbi.nlm.nih.gov/nucleotide/MT557073.1?report=genbank&log$=nucltop&blast_rank=1&RID=YSYTHWMA013) | 941 | 100.00% |
|  |  |  |  | *Alternaria tenuissima* | [MK748314.1](https://www.ncbi.nlm.nih.gov/nucleotide/MK748314.1?report=genbank&log$=nucltop&blast_rank=2&RID=YSYTHWMA013) | 941 | 100.00% |
| 132 | Sep | 5CO | OM236976 | [*Phoma multirostrata*](https://blast.ncbi.nlm.nih.gov/Blast.cgi) | [KR709058.1](https://www.ncbi.nlm.nih.gov/nucleotide/KR709058.1?report=genbank&log$=nucltop&blast_rank=1&RID=EBEXM5JP016) | 944 | 99.81% |
| 133 | Sep | 5CO | OM236977 | [*Epicoccum* sp.](https://blast.ncbi.nlm.nih.gov/Blast.cgi) | [JF817322.1](https://www.ncbi.nlm.nih.gov/nucleotide/JF817322.1?report=genbank&log$=nucltop&blast_rank=1&RID=Z0XAZSJ5013) | 771 | 100.00% |
|  |  |  |  | *Epicoccum nigrum* | [MN089646.1](https://www.ncbi.nlm.nih.gov/nucleotide/MN089646.1?report=genbank&log$=nucltop&blast_rank=2&RID=Z0XAZSJ5013) | 765 | 99.76% |
| 134 | Sep | 5CO | OM236978 | *Epicoccum nigrum* | [KJ589556.1](https://www.ncbi.nlm.nih.gov/nucleotide/KJ589556.1?report=genbank&log$=nucltop&blast_rank=1&RID=Z0XGYY47013) | 1223 | 99.10% |
| 135 | Sep | 5CO | OM236979 | *Alternaria alternata* | [MN249500.1](https://www.ncbi.nlm.nih.gov/nucleotide/MN249500.1?report=genbank&log$=nucltop&blast_rank=1&RID=Z0XPGKTC013) | 893 | 100.00% |
| 136 | Sep | 5CO | OM236980 | [*Epicoccum nigrum*](https://blast.ncbi.nlm.nih.gov/Blast.cgi) | [KM877476.1](https://www.ncbi.nlm.nih.gov/nucleotide/KM877476.1?report=genbank&log$=nucltop&blast_rank=1&RID=EBEZF84M013) | 929 | 99.61% |
| 137 | Sep | 5CO | OM236981 | [*Epicoccum* sp.](https://blast.ncbi.nlm.nih.gov/Blast.cgi) | [HQ914874.1](https://www.ncbi.nlm.nih.gov/nucleotide/HQ914874.1?report=genbank&log$=nucltop&blast_rank=1&RID=EBF1WZTS013) | 939 | 99.61% |
|  |  |  |  | [*Epicoccum nigrum*](https://blast.ncbi.nlm.nih.gov/Blast.cgi) | [MN089646.1](https://www.ncbi.nlm.nih.gov/nucleotide/MN089646.1?report=genbank&log$=nucltop&blast_rank=2&RID=EBF1WZTS013) | 933 | 99.42% |
| 138 | Sep | 5CO | OM236982 | *Penicillium oxalicum* | [KP418787.1](https://www.ncbi.nlm.nih.gov/nucleotide/KP418787.1?report=genbank&log$=nucltop&blast_rank=1&RID=Z0XWA3HE016) | 915 | 100.00% |
| 139 | Sep | 5CO | OM236983 | [*Alternaria alternata*](https://blast.ncbi.nlm.nih.gov/Blast.cgi) | [MZ361914.1](https://www.ncbi.nlm.nih.gov/nucleotide/MZ361914.1?report=genbank&log$=nucltop&blast_rank=1&RID=EBF4HK4F016) | 822 | 99.56% |
| 140 | Sep | 5CO | OM236984 | [*Cladosporium* sp.](https://blast.ncbi.nlm.nih.gov/Blast.cgi) | [MT729897.1](https://www.ncbi.nlm.nih.gov/nucleotide/MT729897.1?report=genbank&log$=nucltop&blast_rank=1&RID=EBF6A8TN013) | 941 | 99.80% |
|  |  |  |  | [*Cladosporium cladosporioides*](https://blast.ncbi.nlm.nih.gov/Blast.cgi) | [MT729833.1](https://www.ncbi.nlm.nih.gov/nucleotide/MT729833.1?report=genbank&log$=nucltop&blast_rank=3&RID=EBF6A8TN013) | 939 | 99.80% |
| 141 | Sep | 5CO | OM236985 | *Penicillium* sp. | [MH622766.1](https://www.ncbi.nlm.nih.gov/nucleotide/MH622766.1?report=genbank&log$=nucltop&blast_rank=1&RID=Z0Y1NJU2016) | 1121 | 99.65% |
|  |  |  |  | [*Penicillium oxalicum*](https://blast.ncbi.nlm.nih.gov/Blast.cgi) | [KY962009.1](https://www.ncbi.nlm.nih.gov/nucleotide/KY962009.1?report=genbank&log$=nucltop&blast_rank=2&RID=Z0Y1NJU2016) | 1048 | 99.48% |
| 142 | Sep | PS | OM236986 | [*Cladosporium* sp.](https://blast.ncbi.nlm.nih.gov/Blast.cgi) | [MT729897.1](https://www.ncbi.nlm.nih.gov/nucleotide/MT729897.1?report=genbank&log$=nucltop&blast_rank=1&RID=EBFASU58016) | 941 | 99.80% |
|  |  |  |  | [*Cladosporium cladosporioides*](https://blast.ncbi.nlm.nih.gov/Blast.cgi) | [MF061760.1](https://www.ncbi.nlm.nih.gov/nucleotide/MF061760.1?report=genbank&log$=nucltop&blast_rank=2&RID=EBFASU58016) | 939 | 99.80% |
| 143 | Sep | PS | OM236987 | [*Cladosporium* sp.](https://blast.ncbi.nlm.nih.gov/Blast.cgi) | [JF819132.1](https://www.ncbi.nlm.nih.gov/nucleotide/JF819132.1?report=genbank&log$=nucltop&blast_rank=1&RID=EBFDB0ES013) | 939 | 99.61% |
|  |  |  |  | [*Cladosporium cladosporioides*](https://blast.ncbi.nlm.nih.gov/Blast.cgi) | [MG820070.1](https://www.ncbi.nlm.nih.gov/nucleotide/MG820070.1?report=genbank&log$=nucltop&blast_rank=3&RID=EBFDB0ES013) | 929 | 99.22% |
| 144 | Sep | PS | OM236988 | *Epicoccum nigrum* | [KF986447.1](https://www.ncbi.nlm.nih.gov/nucleotide/KF986447.1?report=genbank&log$=nucltop&blast_rank=1&RID=Z0Y7P81R013) | 791 | 100.00% |
| 145 | Sep | PS | OM236989 | *Cladosporium anthropophilum* | [MT508803.1](https://www.ncbi.nlm.nih.gov/nucleotide/MT508803.1?report=genbank&log$=nucltop&blast_rank=1&RID=Z0YFWX1J013) | 798 | 100.00% |
| 146 | Sep | PS | OM236990 | *Alternaria alternata* | [MN249500.1](https://www.ncbi.nlm.nih.gov/nucleotide/MN249500.1?report=genbank&log$=nucltop&blast_rank=1&RID=Z0YX8ZSW016) | 893 | 100.00% |
| 147 | Sep | PS | OM236991 | [*Cladosporium cladosporioides*](https://blast.ncbi.nlm.nih.gov/Blast.cgi) | [KR709050.1](https://www.ncbi.nlm.nih.gov/nucleotide/KR709050.1?report=genbank&log$=nucltop&blast_rank=1&RID=EBFH34SD016) | 942 | 99.42% |
| 148 | Sep | PS | OM236992 | [*Epicoccum nigrum*](https://blast.ncbi.nlm.nih.gov/Blast.cgi) | [MN089646.1](https://www.ncbi.nlm.nih.gov/nucleotide/MN089646.1?report=genbank&log$=nucltop&blast_rank=1&RID=EBFKEN6P013) | 931 | 99.42% |
| 150 | Sep | PS | OM236993 | *Epicoccum nigrum* | [MN089646.1](https://www.ncbi.nlm.nih.gov/nucleotide/MN089646.1?report=genbank&log$=nucltop&blast_rank=1&RID=Z0Z2CCT7013) | 771 | 100.00% |
| 151 | Sep | PS | OM236994 | [*Cladosporium cladosporioides*](https://blast.ncbi.nlm.nih.gov/Blast.cgi) | [MW113324.1](https://www.ncbi.nlm.nih.gov/nucleotide/MW113324.1?report=genbank&log$=nucltop&blast_rank=1&RID=EBFNY0ES016) | 946 | 99.81% |
| 152 | Sep | PS | OM236995 | [*Dothideomycetes* sp.](https://blast.ncbi.nlm.nih.gov/Blast.cgi) | [KX909022.1](https://www.ncbi.nlm.nih.gov/nucleotide/KX909022.1?report=genbank&log$=nucltop&blast_rank=1&RID=EBFS4097013) | 922 | 99.80% |
|  |  |  |  | [*Epicoccum nigrum*](https://blast.ncbi.nlm.nih.gov/Blast.cgi) | [MN089646.1](https://www.ncbi.nlm.nih.gov/nucleotide/MN089646.1?report=genbank&log$=nucltop&blast_rank=2&RID=EBFS4097013) | 918 | 99.80% |
| 153 | Sep | PS | OM236996 | *Alternaria alternata* | [MN249500.1](https://www.ncbi.nlm.nih.gov/nucleotide/MN249500.1?report=genbank&log$=nucltop&blast_rank=1&RID=Z0Z81DHR016) | 887 | 100.00% |
| 154 | Sep | PS | OM236997 | *Alternaria alternata* | [MN249500.1](https://www.ncbi.nlm.nih.gov/nucleotide/MN249500.1?report=genbank&log$=nucltop&blast_rank=1&RID=Z0ZFF5NU013) | 893 | 100.00% |
| 155 | Sep | PS | OM236998 | *Cladosporium anthropophilum* | [MT508803.1](https://www.ncbi.nlm.nih.gov/nucleotide/MT508803.1?report=genbank&log$=nucltop&blast_rank=1&RID=Z0ZR6HRE013) | 883 | 100.00% |
| 156 | Sep | PS | OM236999 | *Alternaria alternata* | [MN249500.1](https://www.ncbi.nlm.nih.gov/nucleotide/MN249500.1?report=genbank&log$=nucltop&blast_rank=1&RID=Z2VSUEEM013) | 887 | 100.00% |
| 157 | Sep | PS | OM237001 | [*Cladosporium oxysporum*](https://blast.ncbi.nlm.nih.gov/Blast.cgi) | [MF040201.1](https://www.ncbi.nlm.nih.gov/nucleotide/MF040201.1?report=genbank&log$=nucltop&blast_rank=1&RID=EK3EFMS8013) | 941 | 99.80% |
| 158 | Sep | PS | OM237002 | *Alternaria alternata* | [MN249500.1](https://www.ncbi.nlm.nih.gov/nucleotide/MN249500.1?report=genbank&log$=nucltop&blast_rank=1&RID=Z2VYXZY9016) | 891 | 100.00% |
| 159 | Sep | PS | OM237003 | [*Alternaria alternata*](https://blast.ncbi.nlm.nih.gov/Blast.cgi) | [MZ350148.1](https://www.ncbi.nlm.nih.gov/nucleotide/MZ350148.1?report=genbank&log$=nucltop&blast_rank=1&RID=EBFVBDC5016) | 983 | 100.00% |
| 160 | Sep | PS | OM237004 | *Epicoccum nigrum* | [KF986447.1](https://www.ncbi.nlm.nih.gov/nucleotide/KF986447.1?report=genbank&log$=nucltop&blast_rank=1&RID=Z2W509YC013) | 791 | 99.77% |
| 161 | Sep | PS | OM237005 | *Alternaria alternata* | [MW361306.1](https://www.ncbi.nlm.nih.gov/nucleotide/MW361306.1?report=genbank&log$=nucltop&blast_rank=1&RID=Z2WBMVMF013) | 891 | 100.00% |
| 163 | Sep | 5CO | OM237006 | [*Alternaria alternata*](https://blast.ncbi.nlm.nih.gov/Blast.cgi) | [MW723786.1](https://www.ncbi.nlm.nih.gov/nucleotide/MW723786.1?report=genbank&log$=nucltop&blast_rank=1&RID=EBFXG4HS016) | 778 | 95.16% |
| 164 | Sep | 5CO | OM237007 | [*Epicoccum* sp.](https://blast.ncbi.nlm.nih.gov/Blast.cgi) | [KT588463.1](https://www.ncbi.nlm.nih.gov/nucleotide/KT588463.1?report=genbank&log$=nucltop&blast_rank=1&RID=EBFZ7YEG016) | 918 | 98.84% |
| 165 | Sep | 5CO | OM237008 | [*Sarocladium* sp.](https://blast.ncbi.nlm.nih.gov/Blast.cgi) | [MK793703.1](https://www.ncbi.nlm.nih.gov/nucleotide/MK793703.1?report=genbank&log$=nucltop&blast_rank=1&RID=EBG1V8NA013) | 845 | 100.00% |
|  |  |  |  | [*Sarocladium strictum*](https://blast.ncbi.nlm.nih.gov/Blast.cgi) | [MF818320.1](https://www.ncbi.nlm.nih.gov/nucleotide/MF818320.1?report=genbank&log$=nucltop&blast_rank=2&RID=EBG1V8NA013) | 845 | 100.00% |
| 166 | Sep | PS | OM237009 | *Alternaria alternata* | [MN249500.1](https://www.ncbi.nlm.nih.gov/nucleotide/MN249500.1?report=genbank&log$=nucltop&blast_rank=1&RID=Z2WHCN3F016) | 845 | 100.00% |
| 167 | Sep | PS | OM237010 | [Fungal sp.](https://blast.ncbi.nlm.nih.gov/Blast.cgi) | [MW603421.1](https://www.ncbi.nlm.nih.gov/nucleotide/MW603421.1?report=genbank&log$=nucltop&blast_rank=1&RID=EBG4PJTC013) | 948 | 100.00% |
|  |  |  |  | [*Cladosporium* sp.](https://blast.ncbi.nlm.nih.gov/Blast.cgi) | [KT310929.1](https://www.ncbi.nlm.nih.gov/nucleotide/KT310929.1?report=genbank&log$=nucltop&blast_rank=2&RID=EBG4PJTC013) | 944 | 99.81% |
| 168 | Sep | PS | OM237011 | [*Curvularia lunata*](https://blast.ncbi.nlm.nih.gov/Blast.cgi) | [MN598894.1](https://www.ncbi.nlm.nih.gov/nucleotide/MN598894.1?report=genbank&log$=nucltop&blast_rank=1&RID=EBG82GUX016) | 1026 | 99.47% |
| 169 | Sep | PS | OM237012 | [*Pleosporaceae* sp.](https://blast.ncbi.nlm.nih.gov/Blast.cgi) | [MN486540.1](https://www.ncbi.nlm.nih.gov/nucleotide/MN486540.1?report=genbank&log$=nucltop&blast_rank=1&RID=EK3HCY8B013) | 1072 | 99.49% |
|  |  |  |  | [*Curvularia intermedia*](https://blast.ncbi.nlm.nih.gov/Blast.cgi) | [KU856621.1](https://www.ncbi.nlm.nih.gov/nucleotide/KU856621.1?report=genbank&log$=nucltop&blast_rank=2&RID=EK3HCY8B013) | 1059 | 99.66% |
| 170 | Sep | PS | OM237013 | *Alternaria alternata* | [MN249500.1](https://www.ncbi.nlm.nih.gov/nucleotide/MN249500.1?report=genbank&log$=nucltop&blast_rank=1&RID=Z2WPBUKE013) | 896 | 100.00% |
| 171 | Sep | PS | OM237014 | *Alternaria alternata* | [MN249500.1](https://www.ncbi.nlm.nih.gov/nucleotide/MN249500.1?report=genbank&log$=nucltop&blast_rank=1&RID=Z2WVEB9R016) | 893 | 100.00% |
| 172 | Sep | PS | OM237015 | [*Alternaria tenuissima*](https://blast.ncbi.nlm.nih.gov/Blast.cgi) | [MW009002.1](https://www.ncbi.nlm.nih.gov/nucleotide/MW009002.1?report=genbank&log$=nucltop&blast_rank=1&RID=EBGAPX47016) | 987 | 99.63% |
| 173 | Sep | PS | OM237016 | *Alternaria alternata* | [MN249500.1](https://www.ncbi.nlm.nih.gov/nucleotide/MN249500.1?report=genbank&log$=nucltop&blast_rank=1&RID=Z2X2JPDK013) | 918 | 100.00% |
| 174 | Sep | FDP | OM237017 | [*Cladosporium cladosporioides*](https://blast.ncbi.nlm.nih.gov/Blast.cgi) | [MK127535.1](https://www.ncbi.nlm.nih.gov/nucleotide/MK127535.1?report=genbank&log$=nucltop&blast_rank=1&RID=EBGCTZRB016) | 937 | 99.61% |
| 175 | Sep | FDP | OM237018 | [*Talaromyces funiculosus*](https://blast.ncbi.nlm.nih.gov/Blast.cgi) | [MG873486.1](https://www.ncbi.nlm.nih.gov/nucleotide/MG873486.1?report=genbank&log$=nucltop&blast_rank=1&RID=Z2X8P9F9013) | 878 | 100.00% |
| 176 | Sep | FDP | OM237019 | [*Alternaria alternata*](https://blast.ncbi.nlm.nih.gov/Blast.cgi) | [KY694479.1](https://www.ncbi.nlm.nih.gov/nucleotide/KY694479.1?report=genbank&log$=nucltop&blast_rank=1&RID=EBGF3S10016) | 645 | 88.76% |
| 177 | Sep | 3CP | OM237020 | [*Nothophoma quercina*](https://blast.ncbi.nlm.nih.gov/Blast.cgi) | [MH789714.1](https://www.ncbi.nlm.nih.gov/nucleotide/MH789714.1?report=genbank&log$=nucltop&blast_rank=1&RID=EBGH1MPP016) | 928 | 100.00% |
| 178 | Sep | LIB | OM237021 | *Alternaria alternata* | [MN249500.1](https://www.ncbi.nlm.nih.gov/nucleotide/MN249500.1?report=genbank&log$=nucltop&blast_rank=1&RID=Z2XDXKJ6013) | 876 | 100.00% |
| 179 | Sep | LIB | OM237022 | *Alternaria alternata* | [MN249500.1](https://www.ncbi.nlm.nih.gov/nucleotide/MN249500.1?report=genbank&log$=nucltop&blast_rank=1&RID=Z2XK2T9W013) | 894 | 100.00% |
| 180 | Sep | LIB | OM237023 | [*Cladosporium cladosporioides*](https://blast.ncbi.nlm.nih.gov/Blast.cgi) | [KY114882.1](https://www.ncbi.nlm.nih.gov/nucleotide/KY114882.1?report=genbank&log$=nucltop&blast_rank=1&RID=EBGKART8016) | 931 | 99.61% |
| 181 | Sep | LIB | OM237024 | [Uncultured fungus](https://blast.ncbi.nlm.nih.gov/Blast.cgi) | [KX515416.1](https://www.ncbi.nlm.nih.gov/nucleotide/KX515416.1?report=genbank&log$=nucltop&blast_rank=1&RID=EK3NJWAJ013) | 1016 | 99.82% |
|  |  |  |  | [*Dothidea insculpta*](https://blast.ncbi.nlm.nih.gov/Blast.cgi) | [LC591852.1](https://www.ncbi.nlm.nih.gov/nucleotide/LC591852.1?report=genbank&log$=nucltop&blast_rank=4&RID=EK3NJWAJ013) | 965 | 99.62% |
| 182 | Sep | LIB | OM237025 | [*Alternaria tenuissima*](https://blast.ncbi.nlm.nih.gov/Blast.cgi) | [MW009004.1](https://www.ncbi.nlm.nih.gov/nucleotide/MW009004.1?report=genbank&log$=nucltop&blast_rank=1&RID=EBGNDJYW016) | 987 | 99.81% |
| 183 | Sep | PS | OM237026 | [Fungal sp.](https://blast.ncbi.nlm.nih.gov/Blast.cgi) | [MW603421.1](https://www.ncbi.nlm.nih.gov/nucleotide/MW603421.1?report=genbank&log$=nucltop&blast_rank=1&RID=EBGRECBS016) | 939 | 100.00% |
|  |  |  |  | [*Cladosporium* sp.](https://blast.ncbi.nlm.nih.gov/Blast.cgi) | [KT310929.1](https://www.ncbi.nlm.nih.gov/nucleotide/KT310929.1?report=genbank&log$=nucltop&blast_rank=3&RID=EBGRECBS016) | 933 | 99.80% |
| 184 | Sep | PS | OM237027 | *Epicoccum nigrum* | [KM877476.1](https://www.ncbi.nlm.nih.gov/nucleotide/KM877476.1?report=genbank&log$=nucltop&blast_rank=1&RID=EZSFGJS4013) | 913 | 98.09% |
| 185 | Sep | PS | OM237028 | [*Exserohilum rostratum*](https://blast.ncbi.nlm.nih.gov/Blast.cgi) | [MT322135.1](https://www.ncbi.nlm.nih.gov/nucleotide/MT322135.1?report=genbank&log$=nucltop&blast_rank=1&RID=EBGVE5UA013) | 1035 | 99.30% |
| 186 | Sep | PS | OM237029 | [*Alternaria* sp.](https://blast.ncbi.nlm.nih.gov/Blast.cgi) | [MW581345.1](https://www.ncbi.nlm.nih.gov/nucleotide/MW581345.1?report=genbank&log$=nucltop&blast_rank=1&RID=EBGXPTH2013) | 979 | 99.44% |
| 187 | Sep | PS | OM237030 | [*Epicoccum* sp.](https://blast.ncbi.nlm.nih.gov/Blast.cgi) | [FJ788133.1](https://www.ncbi.nlm.nih.gov/nucleotide/FJ788133.1?report=genbank&log$=nucltop&blast_rank=1&RID=EBGZS8ZS016) | 1136 | 98.86% |
|  |  |  |  | [*Epicoccum nigrum*](https://blast.ncbi.nlm.nih.gov/Blast.cgi) | [KM877476.1](https://www.ncbi.nlm.nih.gov/nucleotide/KM877476.1?report=genbank&log$=nucltop&blast_rank=3&RID=EBGZS8ZS016) | 933 | 99.42% |
| 188 | Sep | PS | OM237031 | [*Alternaria* sp.](https://blast.ncbi.nlm.nih.gov/Blast.cgi) | [MW008994.1](https://www.ncbi.nlm.nih.gov/nucleotide/MW008994.1?report=genbank&log$=nucltop&blast_rank=1&RID=EBH327V5016) | 979 | 99.81% |
|  |  |  |  | [*Alternaria tenuissima*](https://blast.ncbi.nlm.nih.gov/Blast.cgi) | [KF709214.1](https://www.ncbi.nlm.nih.gov/nucleotide/KF709214.1?report=genbank&log$=nucltop&blast_rank=2&RID=EBH327V5016) | 976 | 99.44% |
| 1 | Oct | PS | OM237032 | [*Didymella glomerata*](https://blast.ncbi.nlm.nih.gov/Blast.cgi) | [KY801690.1](https://www.ncbi.nlm.nih.gov/nucleotide/KY801690.1?report=genbank&log$=nucltop&blast_rank=1&RID=BY30CNT5013) | 917 | 99.80% |
| 2 | Oct | PS | OM237033 | *Alternaria alternata* | [MW723716.1](https://www.ncbi.nlm.nih.gov/nucleotide/MW723716.1?report=genbank&log$=nucltop&blast_rank=1&RID=EUVXHY6001R) | 966 | 98.54% |
| 3 | Oct | PS | OM237034 | [Uncultured *Cladosporium*](https://blast.ncbi.nlm.nih.gov/Blast.cgi) | [JQ990199.1](https://www.ncbi.nlm.nih.gov/nucleotide/JQ990199.1?report=genbank&log$=nucltop&blast_rank=1&RID=BY348H1R016) | 920 | 99.03% |
|  |  |  |  | [*Cladosporium uredinicola*](https://blast.ncbi.nlm.nih.gov/Blast.cgi) | [KX082931.1](https://www.ncbi.nlm.nih.gov/nucleotide/KX082931.1?report=genbank&log$=nucltop&blast_rank=2&RID=BY348H1R016) | 918 | 99.03% |
| 4 | Oct | PS | OM237035 | [*Aspergillus nidulans*](https://blast.ncbi.nlm.nih.gov/Blast.cgi) | [MN901610.1](https://www.ncbi.nlm.nih.gov/nucleotide/MN901610.1?report=genbank&log$=nucltop&blast_rank=1&RID=ECTUTEPE016) | 985 | 100.00% |
| 5 | Oct | PS | OM237036 | [*Cladosporium cladosporioides*](https://blast.ncbi.nlm.nih.gov/Blast.cgi) | [MT367262.1](https://www.ncbi.nlm.nih.gov/nucleotide/MT367262.1?report=genbank&log$=nucltop&blast_rank=1&RID=BY38F314016) | 898 | 99.00% |
| 6 | Oct | PS | OM237037 | [Fungal sp.](https://blast.ncbi.nlm.nih.gov/Blast.cgi) | [MW603421.1](https://www.ncbi.nlm.nih.gov/nucleotide/MW603421.1?report=genbank&log$=nucltop&blast_rank=1&RID=BY3DF9X101R) | 941 | 99.61% |
|  |  |  |  | [*Cladosporium tenuissimum*](https://blast.ncbi.nlm.nih.gov/Blast.cgi) | [JN624904.1](https://www.ncbi.nlm.nih.gov/nucleotide/JN624904.1?report=genbank&log$=nucltop&blast_rank=4&RID=BY3DF9X101R) | 935 | 99.80% |
| 7 | Oct | 3CP | OM237038 | [*Aspergillus ochraceus*](https://blast.ncbi.nlm.nih.gov/Blast.cgi) | [KU743890.1](https://www.ncbi.nlm.nih.gov/nucleotide/KU743890.1?report=genbank&log$=nucltop&blast_rank=1&RID=BY3MVGA2013) | 1007 | 99.46% |
| 8 | Oct | 3CP | OM237039 | [Fungal sp.](https://blast.ncbi.nlm.nih.gov/Blast.cgi) | [KX098100.1](https://www.ncbi.nlm.nih.gov/nucleotide/KX098100.1?report=genbank&log$=nucltop&blast_rank=1&RID=BY3SNK6H013) | 977 | 99.63% |
|  |  |  |  | [*Aspergillus creber*](https://blast.ncbi.nlm.nih.gov/Blast.cgi) | [KT310996.1](https://www.ncbi.nlm.nih.gov/nucleotide/KT310996.1?report=genbank&log$=nucltop&blast_rank=2&RID=BY3SNK6H013) | 977 | 99.63% |
| 9 | Oct | 5CP | OM237040 | *Alternaria tenuissima* | [MK683710.1](https://www.ncbi.nlm.nih.gov/nucleotide/MK683710.1?report=genbank&log$=nucltop&blast_rank=1&RID=EUW7KRVF013) | 941 | 97.64% |
| 10 | Oct | LIB | OM237041 | *Arthrinium arundinis* | [LT719147.1](https://www.ncbi.nlm.nih.gov/nucleotide/LT719147.1?report=genbank&log$=nucltop&blast_rank=1&RID=EUWJ9DK5013) | 3192 | 99.33% |
| 11 | Oct | LIB | OM237042 | [*Filobasidium magnum*](https://blast.ncbi.nlm.nih.gov/Blast.cgi) | [MN848545.1](https://www.ncbi.nlm.nih.gov/nucleotide/MN848545.1?report=genbank&log$=nucltop&blast_rank=1&RID=BY3VY5G9016) | 843 | 98.53% |
| 12 | Oct | 3CO | OM237043 | [*Alternaria* sp.](https://blast.ncbi.nlm.nih.gov/Blast.cgi) | [MK649967.1](https://www.ncbi.nlm.nih.gov/nucleotide/MK649967.1?report=genbank&log$=nucltop&blast_rank=1&RID=BY425RAJ013) | 966 | 99.26% |
|  |  |  |  | [*Alternaria consortialis*](https://blast.ncbi.nlm.nih.gov/Blast.cgi) | [KM977759.1](https://www.ncbi.nlm.nih.gov/nucleotide/KM977759.1?report=genbank&log$=nucltop&blast_rank=3&RID=BY425RAJ013) | 965 | 99.07% |
| 13 | Oct | 3CO | OM237044 | [Uncultured fungus](https://blast.ncbi.nlm.nih.gov/Blast.cgi) | [MF971888.1](https://www.ncbi.nlm.nih.gov/nucleotide/MF971888.1?report=genbank&log$=nucltop&blast_rank=1&RID=BY45XMKB016) | 833 | 95.27% |
|  |  |  |  | [*Cymostachys thailandica*](https://blast.ncbi.nlm.nih.gov/Blast.cgi) | [NR_161139.1](https://www.ncbi.nlm.nih.gov/nucleotide/NR_161139.1?report=genbank&log$=nucltop&blast_rank=3&RID=BY45XMKB016) | 815 | 94.72% |
| 14 | Oct | 3CO | OM237045 | *Alternaria alternata* | [MW723716.1](https://www.ncbi.nlm.nih.gov/nucleotide/MW723716.1?report=genbank&log$=nucltop&blast_rank=1&RID=EUW1YS3J013) | 950 | 97.83% |
| 15 | Oct | 3CO | OM237046 | [*Epicoccum* sp.](https://blast.ncbi.nlm.nih.gov/Blast.cgi) | [KC329632.1](https://www.ncbi.nlm.nih.gov/nucleotide/KC329632.1?report=genbank&log$=nucltop&blast_rank=1&RID=EG26V3AV013) | 928 | 99.80% |
|  |  |  |  | [*Epicoccum nigrum*](https://blast.ncbi.nlm.nih.gov/Blast.cgi) | [JN088232.1](https://www.ncbi.nlm.nih.gov/nucleotide/JN088232.1?report=genbank&log$=nucltop&blast_rank=2&RID=EG26V3AV013) | 928 | 99.80% |
| 16 | Oct | CDO | OM237047 | [*Arthrinium* sp.](https://blast.ncbi.nlm.nih.gov/Blast.cgi) | [KP900332.1](https://www.ncbi.nlm.nih.gov/nucleotide/KP900332.1?report=genbank&log$=nucltop&blast_rank=1&RID=BY4AFBTK013) | 966 | 98.90% |
|  |  |  |  | [*Arthrinium arundinis*](https://blast.ncbi.nlm.nih.gov/Blast.cgi) | [LT719147.1](https://www.ncbi.nlm.nih.gov/nucleotide/LT719147.1?report=genbank&log$=nucltop&blast_rank=3&RID=BY4AFBTK013) | 1823 | 98.54% |
| 17 | Oct | CDO | OM237048 | [*Cladosporium anthropophilum*](https://blast.ncbi.nlm.nih.gov/Blast.cgi) | [MT508803.1](https://www.ncbi.nlm.nih.gov/nucleotide/MT508803.1?report=genbank&log$=nucltop&blast_rank=1&RID=EJB0UVSH013) | 745 | 98.13% |
| 18 | Oct | CDO | OM237049 | [*Cladosporium macrocarpum*](https://blast.ncbi.nlm.nih.gov/Blast.cgi) | [KX815295.1](https://www.ncbi.nlm.nih.gov/nucleotide/KX815295.1?report=genbank&log$=nucltop&blast_rank=1&RID=BY4EHP4Z013) | 942 | 99.81% |
| 19 | Oct | CDO | OM237050 | [*Cladosporium oryzae*](https://blast.ncbi.nlm.nih.gov/Blast.cgi) | [KY400092.1](https://www.ncbi.nlm.nih.gov/nucleotide/KY400092.1?report=genbank&log$=nucltop&blast_rank=1&RID=BY4JZ6GT013) | 946 | 99.43% |
| 20 | Oct | CDO | OM237051 | [Uncultured fungus](https://blast.ncbi.nlm.nih.gov/Blast.cgi) | [KX515083.1](https://www.ncbi.nlm.nih.gov/nucleotide/KX515083.1?report=genbank&log$=nucltop&blast_rank=1&RID=EG2BVVME013) | 1053 | 98.98% |
|  |  |  |  | [*Naganishia albida*](https://blast.ncbi.nlm.nih.gov/Blast.cgi) | [KY238163.1](https://www.ncbi.nlm.nih.gov/nucleotide/KY238163.1?report=genbank&log$=nucltop&blast_rank=3&RID=EG2BVVME013) | 1051 | 99.14% |
| 21 | Oct | CDP | OM237052 | [*Cladosporium anthropophilum*](https://blast.ncbi.nlm.nih.gov/Blast.cgi) | [MT508803.1](https://www.ncbi.nlm.nih.gov/nucleotide/MT508803.1?report=genbank&log$=nucltop&blast_rank=1&RID=BY4PPNDV016) | 778 | 99.53% |
| 22 | Oct | CDP | OM237053 | [*Alternaria alternata*](https://blast.ncbi.nlm.nih.gov/Blast.cgi) | [MN249500.1](https://www.ncbi.nlm.nih.gov/nucleotide/MN249500.1?report=genbank&log$=nucltop&blast_rank=1&RID=EJCN20WF016) | 874 | 98.01% |
| 23 | Oct | CDP | OM237054 | [*Epicoccum* sp.](https://blast.ncbi.nlm.nih.gov/Blast.cgi) | [KC329632.1](https://www.ncbi.nlm.nih.gov/nucleotide/KC329632.1?report=genbank&log$=nucltop&blast_rank=1&RID=EJDW0VXG016) | 883 | 98.22% |
|  |  |  |  | [*Epicoccum nigrum*](https://blast.ncbi.nlm.nih.gov/Blast.cgi) | [JN088232.1](https://www.ncbi.nlm.nih.gov/nucleotide/JN088232.1?report=genbank&log$=nucltop&blast_rank=2&RID=EJDW0VXG016) | 883 | 98.22% |
| 24 | Oct | CDP | OM237055 | [Fungal sp.](https://blast.ncbi.nlm.nih.gov/Blast.cgi) | [KX098033.1](https://www.ncbi.nlm.nih.gov/nucleotide/KX098033.1?report=genbank&log$=nucltop&blast_rank=1&RID=BY4US2VN013) | 976 | 99.81% |
|  |  |  |  | [*Aspergillus sydowii*](https://blast.ncbi.nlm.nih.gov/Blast.cgi) | [MT582755.1](https://www.ncbi.nlm.nih.gov/nucleotide/MT582755.1?report=genbank&log$=nucltop&blast_rank=2&RID=BY4US2VN013) | 974 | 99.81% |
| 25 | Oct | PS | OM237056 | [*Talaromyces pinophilus*](https://blast.ncbi.nlm.nih.gov/Blast.cgi) | [MF686812.1](https://www.ncbi.nlm.nih.gov/nucleotide/MF686812.1?report=genbank&log$=nucltop&blast_rank=1&RID=BY4YM4JW016) | 1377 | 99.10% |
| 27 | Oct | FDO | OM237057 | [*Cercospora beticola*](https://blast.ncbi.nlm.nih.gov/Blast.cgi) | [MW581288.1](https://www.ncbi.nlm.nih.gov/nucleotide/MW581288.1?report=genbank&log$=nucltop&blast_rank=1&RID=EG2GJWCX016) | 420 | 85.93% |
| 28 | Oct | FDP | OM237058 | [*Epicoccum nigrum*](https://blast.ncbi.nlm.nih.gov/Blast.cgi) | [GU395519.1](https://www.ncbi.nlm.nih.gov/nucleotide/GU395519.1?report=genbank&log$=nucltop&blast_rank=1&RID=EG2W11F4016) | 928 | 99.61% |
| 29 | Oct | FDP | OM237059 | [*Talaromyces funiculosus*](https://blast.ncbi.nlm.nih.gov/Blast.cgi) | [MW958047.1](https://www.ncbi.nlm.nih.gov/nucleotide/MW958047.1?report=genbank&log$=nucltop&blast_rank=1&RID=BY52P7S6013) | 1000 | 99.82% |
| 1 | Nov | 3CP | OM237060 | [*Naganishia uzbekistanensis*](https://blast.ncbi.nlm.nih.gov/Blast.cgi) | [MG720264.1](https://www.ncbi.nlm.nih.gov/nucleotide/MG720264.1?report=genbank&log$=nucltop&blast_rank=1&RID=0FUXWSHV013) | 905 | 99.80% |
| 2 | Nov | 3CP | OM237061 | [*Naganishia uzbekistanensis*](https://blast.ncbi.nlm.nih.gov/Blast.cgi) | [MG720264.1](https://www.ncbi.nlm.nih.gov/nucleotide/MG720264.1?report=genbank&log$=nucltop&blast_rank=1&RID=0FV3TXU9013) | 904 | 99.80% |
| 3 | Nov | 5CP | OM237062 | [*Cladosporium halotolerans*](https://blast.ncbi.nlm.nih.gov/Blast.cgi) | [MN859971.1](https://www.ncbi.nlm.nih.gov/nucleotide/MN859971.1?report=genbank&log$=nucltop&blast_rank=1&RID=ECTZU27E016) | 939 | 99.23% |
| 4 | Nov | 5CP | OM237063 | [*Periconia pseudodigitata*](https://blast.ncbi.nlm.nih.gov/Blast.cgi) | [LC014589.1](https://www.ncbi.nlm.nih.gov/nucleotide/LC014589.1?report=genbank&log$=nucltop&blast_rank=1&RID=ECU202ZZ016) | 900 | 99.60% |
| 5 | Nov | 5CP | OM237064 | [*Penicillium oxalicum*](https://blast.ncbi.nlm.nih.gov/Blast.cgi) | [MN121543.1](https://www.ncbi.nlm.nih.gov/nucleotide/MN121543.1?report=genbank&log$=nucltop&blast_rank=1&RID=ECU4K3J7016) | 1007 | 100.00% |
| 6 | Nov | 5CO | OM237065 | [*Purpureocillium lilacinum*](https://blast.ncbi.nlm.nih.gov/Blast.cgi) | [MT530278.1](https://www.ncbi.nlm.nih.gov/nucleotide/MT530278.1?report=genbank&log$=nucltop&blast_rank=1&RID=0FVA28XM016) | 715 | 100.00% |
| 7 | Nov | 5CO | OM237066 | [*Stemphylium lycopersici*](https://blast.ncbi.nlm.nih.gov/Blast.cgi) | [KY290559.1](https://www.ncbi.nlm.nih.gov/nucleotide/KY290559.1?report=genbank&log$=nucltop&blast_rank=1&RID=ECU6HGDP013) | 970 | 99.62% |
| 8 | Nov | PS | OM237067 | [*Stemphylium* sp.](https://blast.ncbi.nlm.nih.gov/Blast.cgi) | [MT556676.1](https://www.ncbi.nlm.nih.gov/nucleotide/MT556676.1?report=genbank&log$=nucltop&blast_rank=1&RID=0FVFGXRX016) | 798 | 100.00% |
|  |  |  |  | [*Stemphylium vesicarium*](https://blast.ncbi.nlm.nih.gov/Blast.cgi) | [MT520583.1](https://www.ncbi.nlm.nih.gov/nucleotide/MT520583.1?report=genbank&log$=nucltop&blast_rank=2&RID=0FVFGXRX016) | 798 | 100.00% |
| 9 | Nov | PS | OM237068 | [Uncultured fungus](https://blast.ncbi.nlm.nih.gov/Blast.cgi) | [KX515758.1](https://www.ncbi.nlm.nih.gov/nucleotide/KX515758.1?report=genbank&log$=nucltop&blast_rank=1&RID=ECU8EP5H013) | 931 | 99.23% |
|  |  |  |  | [*Torula caligans*](https://blast.ncbi.nlm.nih.gov/Blast.cgi) | [JX156379.1](https://www.ncbi.nlm.nih.gov/nucleotide/JX156379.1?report=genbank&log$=nucltop&blast_rank=2&RID=ECU8EP5H013) | 931 | 99.42% |
| 10 | Nov | PS | OM237069 | [*Talaromyces radicus*](https://blast.ncbi.nlm.nih.gov/Blast.cgi) | [MK646059.1](https://www.ncbi.nlm.nih.gov/nucleotide/MK646059.1?report=genbank&log$=nucltop&blast_rank=1&RID=ECUGGTJW016) | 1035 | 99.47% |
| 11 | Nov | PS | OM237070 | [*Aspergillus sydowii*](https://blast.ncbi.nlm.nih.gov/Blast.cgi) | [JQ647895.1](https://www.ncbi.nlm.nih.gov/nucleotide/JQ647895.1?report=genbank&log$=nucltop&blast_rank=1&RID=ECUJ87ET013) | 955 | 99.62% |
| 12 | Nov | PS | OM237071 | [*Aspergillus tubingensis*](https://blast.ncbi.nlm.nih.gov/Blast.cgi) | [MT443912.1](https://www.ncbi.nlm.nih.gov/nucleotide/MT443912.1?report=genbank&log$=nucltop&blast_rank=1&RID=BY5BX5UR016) | 928 | 99.22% |
| 13 | Nov | CDP | OM237072 | [*Microsphaeropsis* sp.](https://blast.ncbi.nlm.nih.gov/Blast.cgi) | [MN153956.1](https://www.ncbi.nlm.nih.gov/nucleotide/MN153956.1?report=genbank&log$=nucltop&blast_rank=1&RID=BY5G48V1013) | 752 | 99.28% |
| 14 | Nov | LIB | OM237073 | [*Penicillium concavorugulosum*](https://blast.ncbi.nlm.nih.gov/Blast.cgi) | [MK841454.1](https://www.ncbi.nlm.nih.gov/nucleotide/MK841454.1?report=genbank&log$=nucltop&blast_rank=1&RID=BY5M2BHW013) | 878 | 100.00% |
| 15 | Nov | LIB | OM237074 | [*Cladosporium oryzae*](https://blast.ncbi.nlm.nih.gov/Blast.cgi) | [KY400092.1](https://www.ncbi.nlm.nih.gov/nucleotide/KY400092.1?report=genbank&log$=nucltop&blast_rank=1&RID=ECUM2GVC016) | 929 | 99.61% |
| 16 | Nov | 3CO | OM237075 | [*Aspergillus* sp.](https://blast.ncbi.nlm.nih.gov/Blast.cgi) | [MT497446.1](https://www.ncbi.nlm.nih.gov/nucleotide/MT497446.1?report=genbank&log$=nucltop&blast_rank=1&RID=BY5RMC20013) | 869 | 99.79% |
|  |  |  |  | [*Aspergillus flavus*](https://blast.ncbi.nlm.nih.gov/Blast.cgi) | [MN856403.1](https://www.ncbi.nlm.nih.gov/nucleotide/MN856403.1?report=genbank&log$=nucltop&blast_rank=4&RID=BY5RMC20013) | 869 | 99.79% |
| 17 | Nov | FDO | OM237076 | [*Aspergillus flavus*](https://blast.ncbi.nlm.nih.gov/Blast.cgi) | [MN856403.1](https://www.ncbi.nlm.nih.gov/nucleotide/MN856403.1?report=genbank&log$=nucltop&blast_rank=1&RID=EUWZGNZZ013) | 1075 | 99.00% |
| 18 | Nov | LIB | OM237077 | [*Penicillium concavorugulosum*](https://blast.ncbi.nlm.nih.gov/Blast.cgi) | [MK841454.1](https://www.ncbi.nlm.nih.gov/nucleotide/MK841454.1?report=genbank&log$=nucltop&blast_rank=1&RID=ECUP5VS601R) | 870 | 99.58% |
| 19 | Nov | 3CO | OM237078 | [*Granulobasidium vellereum*](https://blast.ncbi.nlm.nih.gov/Blast.cgi) | [KJ668538.1](https://www.ncbi.nlm.nih.gov/nucleotide/KJ668538.1?report=genbank&log$=nucltop&blast_rank=1&RID=BY5WFJGC013) | 1007 | 99.11% |
| 20 | Nov | LIB | OM237079 | [*Talaromyces radicus*](https://blast.ncbi.nlm.nih.gov/Blast.cgi) | [JN851019.1](https://www.ncbi.nlm.nih.gov/nucleotide/JN851019.1?report=genbank&log$=nucltop&blast_rank=1&RID=ECUSD4NS01R) | 994 | 99.10% |
| 21 | Nov | LIB | OM237080 | [*Aspergillus tubingensis*](https://blast.ncbi.nlm.nih.gov/Blast.cgi) | [MT443912.1](https://www.ncbi.nlm.nih.gov/nucleotide/MT443912.1?report=genbank&log$=nucltop&blast_rank=1&RID=BY6061T4016) | 922 | 99.60% |
| 1 | Dec | PS | OM237081 | [*Aspergillus flavus*](https://blast.ncbi.nlm.nih.gov/Blast.cgi) | [MW113529.1](https://www.ncbi.nlm.nih.gov/nucleotide/MW113529.1?report=genbank&log$=nucltop&blast_rank=1&RID=ECUV16W301R) | 990 | 99.09% |
| 2 | Dec | PS | OM237082 | [*Cladosporium anthropophilum*](https://blast.ncbi.nlm.nih.gov/Blast.cgi) | [MT508803.1](https://www.ncbi.nlm.nih.gov/nucleotide/MT508803.1?report=genbank&log$=nucltop&blast_rank=1&RID=831J3P2S016) | 710 | 100.00% |
| 3 | Dec | PS | OM237083 | [*Alternaria* sp.](https://blast.ncbi.nlm.nih.gov/Blast.cgi) | [KJ541475.1](https://www.ncbi.nlm.nih.gov/nucleotide/KJ541475.1?report=genbank&log$=nucltop&blast_rank=1&RID=831R6TF7016) | 893 | 99.19% |
|  |  |  |  | [*Alternaria alternata*](https://blast.ncbi.nlm.nih.gov/Blast.cgi) | [MN249500.1](https://www.ncbi.nlm.nih.gov/nucleotide/MN249500.1?report=genbank&log$=nucltop&blast_rank=2&RID=831R6TF7016) | 891 | 99.19% |
| 4 | Dec | PS | OM237084 | [Uncultured *Phoma*](https://blast.ncbi.nlm.nih.gov/Blast.cgi) | [MG462850.1](https://www.ncbi.nlm.nih.gov/nucleotide/MG462850.1?report=genbank&log$=nucltop&blast_rank=1&RID=8320NYY301R) | 785 | 100.00% |
|  |  |  |  | [*Didymella* sp.](https://blast.ncbi.nlm.nih.gov/Blast.cgi) | [MN912325.1](https://www.ncbi.nlm.nih.gov/nucleotide/MN912325.1?report=genbank&log$=nucltop&blast_rank=2&RID=8320NYY301R) | 785 | 100.00% |
| 5 | Dec | PS | OM237085 | [*Cladosporium tenuissimum*](https://blast.ncbi.nlm.nih.gov/Blast.cgi) | [KP689183.1](https://www.ncbi.nlm.nih.gov/nucleotide/KP689183.1?report=genbank&log$=nucltop&blast_rank=1&RID=ECUX8VAX01R) | 924 | 99.80% |
| 6 | Dec | PS | OM237086 | [*Alternaria alternata*](https://blast.ncbi.nlm.nih.gov/Blast.cgi) | [MW008926.1](https://www.ncbi.nlm.nih.gov/nucleotide/MW008926.1?report=genbank&log$=nucltop&blast_rank=1&RID=8326G5NM01R) | 977 | 100.00% |
| 7 | Dec | PS | OM237087 | [*Alternaria alternata*](https://blast.ncbi.nlm.nih.gov/Blast.cgi) | [MW008926.1](https://www.ncbi.nlm.nih.gov/nucleotide/MW008926.1?report=genbank&log$=nucltop&blast_rank=1&RID=832A6TTE013) | 970 | 99.81% |
| 8 | Dec | PS | OM237088 | [*Vishniacozyma carnescens*](https://blast.ncbi.nlm.nih.gov/Blast.cgi) | [MK592823.1](https://www.ncbi.nlm.nih.gov/nucleotide/MK592823.1?report=genbank&log$=nucltop&blast_rank=1&RID=832E5A1P016) | 824 | 98.50% |
| 9 | Dec | PS | OM237089 | [*Cladosporium cladosporioides*](https://blast.ncbi.nlm.nih.gov/Blast.cgi) | [MW113412.1](https://www.ncbi.nlm.nih.gov/nucleotide/MW113412.1?report=genbank&log$=nucltop&blast_rank=1&RID=ECUZBJ9C01R) | 933 | 99.23% |
| 10 | Dec | PS | OM237090 | [*Fusarium delphinoides*](https://blast.ncbi.nlm.nih.gov/Blast.cgi) | [MH141288.1](https://www.ncbi.nlm.nih.gov/nucleotide/MH141288.1?report=genbank&log$=nucltop&blast_rank=1&RID=ECV1BCMY01R) | 935 | 99.61% |
| 11 | Dec | 5CO | OM237091 | [*Aureobasidium* sp.](https://blast.ncbi.nlm.nih.gov/Blast.cgi) | [KY621468.1](https://www.ncbi.nlm.nih.gov/nucleotide/KY621468.1?report=genbank&log$=nucltop&blast_rank=1&RID=EJE6HMJA013) | 756 | 96.14% |
| 12 | Dec | 5CP | OM237092 | [*Aureobasidium* sp.](https://blast.ncbi.nlm.nih.gov/Blast.cgi) | [KY621468.1](https://www.ncbi.nlm.nih.gov/nucleotide/KY621468.1?report=genbank&log$=nucltop&blast_rank=1&RID=832KUHEG016) | 800 | 97.85% |
| 13 | Dec | 5CP | OM237093 | [*Cladosporium* sp.](https://blast.ncbi.nlm.nih.gov/Blast.cgi) | [MW764146.1](https://www.ncbi.nlm.nih.gov/nucleotide/MW764146.1?report=genbank&log$=nucltop&blast_rank=1&RID=BY6CU5HB016) | 857 | 99.79% |
|  |  |  |  | [*Cladosporium anthropophilum*](https://blast.ncbi.nlm.nih.gov/Blast.cgi) | [MT508803.1](https://www.ncbi.nlm.nih.gov/nucleotide/MT508803.1?report=genbank&log$=nucltop&blast_rank=2&RID=BY6CU5HB016) | 852 | 100.00% |
| 14 | Dec | 5CP | OM237094 | [*Cladosporium* sp.](https://blast.ncbi.nlm.nih.gov/Blast.cgi) | [MW764146.1](https://www.ncbi.nlm.nih.gov/nucleotide/MW764146.1?report=genbank&log$=nucltop&blast_rank=1&RID=BY6GH2ZV013) | 863 | 100.00% |
|  |  |  |  | [*Cladosporium cladosporioides*](https://blast.ncbi.nlm.nih.gov/Blast.cgi) | [MT598826.1](https://www.ncbi.nlm.nih.gov/nucleotide/MT598826.1?report=genbank&log$=nucltop&blast_rank=3&RID=BY6GH2ZV013) | 852 | 100.00% |
| 15 | Dec | 5CP | OM237095 | [*Cladosporium* sp.](https://blast.ncbi.nlm.nih.gov/Blast.cgi) | [MW764146.1](https://www.ncbi.nlm.nih.gov/nucleotide/MW764146.1?report=genbank&log$=nucltop&blast_rank=1&RID=BY6MKJWJ013) | 857 | 99.79% |
|  |  |  |  | [*Cladosporium asperulatum*](https://blast.ncbi.nlm.nih.gov/Blast.cgi) | [MN202774.1](https://www.ncbi.nlm.nih.gov/nucleotide/MN202774.1?report=genbank&log$=nucltop&blast_rank=2&RID=BY6MKJWJ013) | 856 | 100.00% |
| 16 | Dec | 3CP | OM237096 | [*Cryptococcus* sp.](https://blast.ncbi.nlm.nih.gov/Blast.cgi) | [MH880193.1](https://www.ncbi.nlm.nih.gov/nucleotide/MH880193.1?report=genbank&log$=nucltop&blast_rank=1&RID=EG4PK3Z8016) | 172 | 98.00% |
| 17 | Dec | 3CP | OM237097 | [*Cladosporium* sp.](https://blast.ncbi.nlm.nih.gov/Blast.cgi) | [MW764146.1](https://www.ncbi.nlm.nih.gov/nucleotide/MW764146.1?report=genbank&log$=nucltop&blast_rank=1&RID=BY8R918S016) | 857 | 99.79% |
|  |  |  |  | [*Cladosporium asperulatum*](https://blast.ncbi.nlm.nih.gov/Blast.cgi) | [MN202774.1](https://www.ncbi.nlm.nih.gov/nucleotide/MN202774.1?report=genbank&log$=nucltop&blast_rank=2&RID=BY8R918S016) | 854 | 100.00% |
| 18 | Dec | 3CP | OM237098 | [*Alternaria alternata*](https://blast.ncbi.nlm.nih.gov/Blast.cgi) | [MW723786.1](https://www.ncbi.nlm.nih.gov/nucleotide/MW723786.1?report=genbank&log$=nucltop&blast_rank=1&RID=BY8UNHGE016) | 826 | 95.57% |
| 19 | Dec | 3CP | OM237099 | [Fungal sp.](https://blast.ncbi.nlm.nih.gov/Blast.cgi) | [MT112989.1](https://www.ncbi.nlm.nih.gov/nucleotide/MT112989.1?report=genbank&log$=nucltop&blast_rank=1&RID=BY8YWN39016) | 885 | 98.04% |
|  |  |  |  | [*Cladosporium cucumerinum*](https://blast.ncbi.nlm.nih.gov/Blast.cgi) | [KR912311.1](https://www.ncbi.nlm.nih.gov/nucleotide/KR912311.1?report=genbank&log$=nucltop&blast_rank=5&RID=BY8YWN39016) | 885 | 98.04% |
| 20 | Dec | CDP | OM237100 | [*Aureobasidium pullulans*](https://blast.ncbi.nlm.nih.gov/Blast.cgi) | [MT363099.1](https://www.ncbi.nlm.nih.gov/nucleotide/MT363099.1?report=genbank&log$=nucltop&blast_rank=1&RID=BY93P46B016) | 821 | 97.69% |
| 21 | Dec | CDO | OM237101 | [*Alternaria porri*](https://blast.ncbi.nlm.nih.gov/Blast.cgi) | [MN872486.1](https://www.ncbi.nlm.nih.gov/nucleotide/MN872486.1?report=genbank&log$=nucltop&blast_rank=1&RID=BY96XM8X016) | 935 | 99.42% |
| 22 | Dec | CDO | OM237102 | [*Valsaria insitiva*](https://blast.ncbi.nlm.nih.gov/Blast.cgi) | [NR_138403.1](https://www.ncbi.nlm.nih.gov/nucleotide/NR_138403.1?report=genbank&log$=nucltop&blast_rank=1&RID=BY9AM47J016) | 983 | 99.27% |
| 23 | Dec | CDO | OM237103 | [Fungal sp.](https://blast.ncbi.nlm.nih.gov/Blast.cgi) | [MT113000.1](https://www.ncbi.nlm.nih.gov/nucleotide/MT113000.1?report=genbank&log$=nucltop&blast_rank=1&RID=BY9E7YN4016) | 889 | 99.00% |
|  |  |  |  | [*Microsphaeropsis* sp.](https://blast.ncbi.nlm.nih.gov/Blast.cgi) | [MN153956.1](https://www.ncbi.nlm.nih.gov/nucleotide/MN153956.1?report=genbank&log$=nucltop&blast_rank=2&RID=BY9E7YN4016) | 889 | 98.99% |
| 24 | Dec | CDO | OM237104 | [*Alternaria alternata*](https://blast.ncbi.nlm.nih.gov/Blast.cgi) | [MT635274.1](https://www.ncbi.nlm.nih.gov/nucleotide/MT635274.1?report=genbank&log$=nucltop&blast_rank=1&RID=BY9HCN6W016) | 898 | 100.00% |
| 25 | Dec | CDO | OM237105 | [*Alternaria* sp.](https://blast.ncbi.nlm.nih.gov/Blast.cgi) | [MW009011.1](https://www.ncbi.nlm.nih.gov/nucleotide/MW009011.1?report=genbank&log$=nucltop&blast_rank=1&RID=BY9MVHR1013) | 996 | 99.82% |
|  |  |  |  | [*Alternaria alternata*](https://blast.ncbi.nlm.nih.gov/Blast.cgi) | [MW008999.1](https://www.ncbi.nlm.nih.gov/nucleotide/MW008999.1?report=genbank&log$=nucltop&blast_rank=4&RID=BY9MVHR1013) | 992 | 99.82% |
| 26 | Dec | FDO | OM237106 | [*Cladosporium* sp.](https://blast.ncbi.nlm.nih.gov/Blast.cgi) | [MW764146.1](https://www.ncbi.nlm.nih.gov/nucleotide/MW764146.1?report=genbank&log$=nucltop&blast_rank=1&RID=BY9TTMK7016) | 835 | 98.53% |
|  |  |  |  | [*Cladosporium asperulatum*](https://blast.ncbi.nlm.nih.gov/Blast.cgi) | [MN202774.1](https://www.ncbi.nlm.nih.gov/nucleotide/MN202774.1?report=genbank&log$=nucltop&blast_rank=2&RID=BY9TTMK7016) | 833 | 99.14% |
| 27 | Dec | 5CO | OM237107 | [*Naganishia antarctica*](https://blast.ncbi.nlm.nih.gov/Blast.cgi) | [MW710183.1](https://www.ncbi.nlm.nih.gov/nucleotide/MW710183.1?report=genbank&log$=nucltop&blast_rank=1&RID=BY9Y8NS4013) | 481 | 85.00% |
| 28 | Dec | CDO | OM237108 | [*Geomyces* sp.](https://blast.ncbi.nlm.nih.gov/Blast.cgi) | [MT367257.1](https://www.ncbi.nlm.nih.gov/nucleotide/MT367257.1?report=genbank&log$=nucltop&blast_rank=1&RID=BYA1T3H4016) | 957 | 99.25% |
| 29 | Dec | CDO | OM237109 | [*Naganishia albida*](https://blast.ncbi.nlm.nih.gov/Blast.cgi) | [KY238164.1](https://www.ncbi.nlm.nih.gov/nucleotide/KY238164.1?report=genbank&log$=nucltop&blast_rank=1&RID=BYA5657Y013) | 955 | 99.06% |
| 30 | Dec | CDO | OM237110 | *Cladosporium anthropophilum* | [MT508803.1](https://www.ncbi.nlm.nih.gov/nucleotide/MT508803.1?report=genbank&log$=nucltop&blast_rank=1&RID=EUXTSG8501R) | 896 | 98.43% |
| 31 | Dec | FDO | OM237111 | [*Cladosporium* sp.](https://blast.ncbi.nlm.nih.gov/Blast.cgi) | [MW764146.1](https://www.ncbi.nlm.nih.gov/nucleotide/MW764146.1?report=genbank&log$=nucltop&blast_rank=1&RID=BYA89Z6G013) | 852 | 99.16% |
|  |  |  |  | [*Cladosporium anthropophilum*](https://blast.ncbi.nlm.nih.gov/Blast.cgi) | [MT508803.1](https://www.ncbi.nlm.nih.gov/nucleotide/MT508803.1?report=genbank&log$=nucltop&blast_rank=2&RID=BYA89Z6G013) | 848 | 99.78% |
| 32 | Dec | PS | OM237112 | *Naganishia albida* | [KY238143.1](https://www.ncbi.nlm.nih.gov/nucleotide/KY238143.1?report=genbank&log$=nucltop&blast_rank=1&RID=EUY4CA1J016) | 1066 | 98.99% |
| 33 | Dec | LIB | OM237113 | [*Penicillium* sp.](https://blast.ncbi.nlm.nih.gov/Blast.cgi) | [KY827369.1](https://www.ncbi.nlm.nih.gov/nucleotide/KY827369.1?report=genbank&log$=nucltop&blast_rank=1&RID=ECV4923101R) | 1000 | 99.82% |
|  |  |  |  | [*Aspergillus japonicus*](https://blast.ncbi.nlm.nih.gov/Blast.cgi) | [MF073328.1](https://www.ncbi.nlm.nih.gov/nucleotide/MF073328.1?report=genbank&log$=nucltop&blast_rank=3&RID=ECV4923101R) | 998 | 99.82% |
| 34 | Dec | CDP | OM237114 | [*Chaetomium globosum*](https://blast.ncbi.nlm.nih.gov/Blast.cgi) | [MG885818.1](https://www.ncbi.nlm.nih.gov/nucleotide/MG885818.1?report=genbank&log$=nucltop&blast_rank=1&RID=ECV7JWTS013) | 976 | 99.44% |
| 1 | Jan | 5CO | OM237115 | *Cladosporium cladosporioides* | [MW556429.1](https://www.ncbi.nlm.nih.gov/nucleotide/MW556429.1?report=genbank&log$=nucltop&blast_rank=1&RID=7CZ0DNGM016) | 950 | 100.00% |
| 2 | Jan | 5CO | OM237116 | *Didymella* sp. | [MG198901.1](https://www.ncbi.nlm.nih.gov/nucleotide/MG198901.1?report=genbank&log$=nucltop&blast_rank=1&RID=EUYE9GX6013) | 2514 | 98.84% |
| 3 | Jan | 5CO | OM237117 | *Penicillium* sp. | [GQ856686.1](https://www.ncbi.nlm.nih.gov/nucleotide/GQ856686.1?report=genbank&log$=nucltop&blast_rank=1&RID=7CZAHYZ6016) | 1018 | 99.64% |
| 4 | Jan | 5CO | OM237118 | Fungal sp. | [MW603421.1](https://www.ncbi.nlm.nih.gov/nucleotide/MW603421.1?report=genbank&log$=nucltop&blast_rank=1&RID=7D4BT0ZH013) | 952 | 99.81% |
|  |  |  |  | *Cladosporium tenuissimum* | [KP689183.1](https://www.ncbi.nlm.nih.gov/nucleotide/KP689183.1?report=genbank&log$=nucltop&blast_rank=2&RID=7D4PTHTX016) | 944 | 99.61% |
| 5 | Jan | 5CO | OM237119 | Fungal sp. | [MW603421.1](https://www.ncbi.nlm.nih.gov/nucleotide/MW603421.1?report=genbank&log$=nucltop&blast_rank=1&RID=7D07YJEP016) | 950 | 99.81% |
|  |  |  |  | *Cladosporium cladosporioides* | [MK127535.1](https://www.ncbi.nlm.nih.gov/nucleotide/MK127535.1?report=genbank&log$=nucltop&blast_rank=2&RID=7D07YJEP016) | 944 | 99.61% |
| 6 | Jan | 5CO | OM237120 | *Didymella* sp. | [MG198901.1](https://www.ncbi.nlm.nih.gov/nucleotide/MG198901.1?report=genbank&log$=nucltop&blast_rank=1&RID=7D0ENYT5016) | 1426 | 98.68% |
| 7 | Jan | 5CO | OM237121 | *Cladosporium tenuissimum* | [MH389061.1](https://www.ncbi.nlm.nih.gov/nucleotide/MH389061.1?report=genbank&log$=nucltop&blast_rank=1&RID=7D0KWJ8Y013) | 942 | 99.81% |
| 8 | Jan | 5CO | OM237122 | *Cladosporium halotolerans* | [MH931821.1](https://www.ncbi.nlm.nih.gov/nucleotide/MH931821.1?report=genbank&log$=nucltop&blast_rank=1&RID=7D0WEK72013) | 952 | 99.43% |
| 9 | Jan | 5CO | OM237123 | *Cladosporium cladosporioides* | [MW453186.1](https://www.ncbi.nlm.nih.gov/nucleotide/MW453186.1?report=genbank&log$=nucltop&blast_rank=1&RID=7D17040B016) | 948 | 99.62% |
| 10 | Jan | 3CP | OM237124 | *Alternaria compacta* | [MW008928.1](https://www.ncbi.nlm.nih.gov/nucleotide/MW008928.1?report=genbank&log$=nucltop&blast_rank=1&RID=7D4XWP5U01R) | 981 | 99.81% |
| 12 | Jan | 3CO | OM237125 | *Cryptococcus rajasthanensis* | [AM262325.1](https://www.ncbi.nlm.nih.gov/nucleotide/AM262325.1?report=genbank&log$=nucltop&blast_rank=1&RID=7D26R14M013) | 732 | 97.88% |
| 13 | Jan | 3CO | OM237126 | Fungal sp. | [MW603396.1](https://www.ncbi.nlm.nih.gov/nucleotide/MW603396.1?report=genbank&log$=nucltop&blast_rank=1&RID=7D2MX6JF013) | 946 | 99.61% |
|  |  |  |  | *Cladosporium cladosporioides* | [MH341182.1](https://www.ncbi.nlm.nih.gov/nucleotide/MH341182.1?report=genbank&log$=nucltop&blast_rank=2&RID=7D2MX6JF013) | 942 | 99.61% |
| 14 | Jan | PS | OM237127 | *Phaeosphaeria* sp. | [KT264716.1](https://www.ncbi.nlm.nih.gov/nucleotide/KT264716.1?report=genbank&log$=nucltop&blast_rank=1&RID=7D301182016) | 944 | 97.64% |
| 15 | Jan | PS | OM237128 | *Alternaria tenuissima* | [MW008898.1](https://www.ncbi.nlm.nih.gov/nucleotide/MW008898.1?report=genbank&log$=nucltop&blast_rank=1&RID=7D35WE72016) | 992 | 100.00% |
| 16 | Jan | PS | OM237129 | *Arthrinium marii* | [MK850396.1](https://www.ncbi.nlm.nih.gov/nucleotide/MK850396.1?report=genbank&log$=nucltop&blast_rank=1&RID=7D56HX34016) | 1066 | 99.32% |
| 17 | Jan | PS | OM237130 | *Cladosporium cladosporioides* | [KU743893.1](https://www.ncbi.nlm.nih.gov/nucleotide/KU743893.1?report=genbank&log$=nucltop&blast_rank=1&RID=7D614FZM013) | 948 | 99.81% |
| 18 | Jan | PS | OM237131 | Fungal sp. | [KU728268.1](https://www.ncbi.nlm.nih.gov/nucleotide/KU728268.1?report=genbank&log$=nucltop&blast_rank=1&RID=7D6J62DV016) | 974 | 99.26% |
|  |  |  |  | *Chaetomium globosum* | [MN809366.1](https://www.ncbi.nlm.nih.gov/nucleotide/MN809366.1?report=genbank&log$=nucltop&blast_rank=2&RID=7D6J62DV016) | 966 | 99.26% |
| 19 | Jan | FDO | OM237132 | *Alternaria alternata* | [MN733022.1](https://www.ncbi.nlm.nih.gov/nucleotide/MN733022.1?report=genbank&log$=nucltop&blast_rank=1&RID=7DUXKCEK013) | 979 | 99.45% |
| 20 | Jan | CDO | OM237133 | *Aureobasidium pullulans* | [HQ267771.1](https://www.ncbi.nlm.nih.gov/nucleotide/HQ267771.1?report=genbank&log$=nucltop&blast_rank=1&RID=7DVE5ZGW013) | 998 | 99.46% |
| 21 | Jan | CDP | OM237134 | *Cladosporium oryzae* | [KY400092.1](https://www.ncbi.nlm.nih.gov/nucleotide/KY400092.1?report=genbank&log$=nucltop&blast_rank=1&RID=7DVNGF2A016) | 948 | 99.24% |
| 22 | Jan | CDP | OM237135 | Fungal sp. | [MW603421.1](https://www.ncbi.nlm.nih.gov/nucleotide/MW603421.1?report=genbank&log$=nucltop&blast_rank=1&RID=7DVYX6VT016) | 948 | 99.81% |
|  |  |  |  | *Cladosporium cladosporioides* | [KY114882.1](https://www.ncbi.nlm.nih.gov/nucleotide/KY114882.1?report=genbank&log$=nucltop&blast_rank=3&RID=7DVYX6VT016) | 946 | 99.61% |
| 23 | Jan | CDP | OM237136 | [*Cladosporium tenuissimum*](https://blast.ncbi.nlm.nih.gov/Blast.cgi) | [HM776419.1](https://www.ncbi.nlm.nih.gov/nucleotide/HM776419.1?report=genbank&log$=nucltop&blast_rank=1&RID=7DW8WUX9016) | 944 | 99.61% |
| 24 | Jan | FDP | OM237137 | *Cladosporium tenuissimum* | [MN121556.1](https://www.ncbi.nlm.nih.gov/nucleotide/MN121556.1?report=genbank&log$=nucltop&blast_rank=1&RID=7DWK3R2K01R) | 941 | 99.42% |
| 25 | Jan | 5CO | OM237138 | Fungal sp. | [MW603396.1](https://www.ncbi.nlm.nih.gov/nucleotide/MW603396.1?report=genbank&log$=nucltop&blast_rank=1&RID=7DWW5W96013) | 944 | 99.42% |
|  |  |  |  | *Cladosporium cladosporioides* | [MW453186.1](https://www.ncbi.nlm.nih.gov/nucleotide/MW453186.1?report=genbank&log$=nucltop&blast_rank=2&RID=7DWW5W96013) | 944 | 99.42% |
| 26 | Jan | 5CO | OM237139 | *Alternaria compacta* | [MW008928.1](https://www.ncbi.nlm.nih.gov/nucleotide/MW008928.1?report=genbank&log$=nucltop&blast_rank=1&RID=7DXBVVWW01R) | 990 | 99.81% |
| 27 | Jan | FDP | OM237140 | *Alternaria* sp. | [MW009028.1](https://www.ncbi.nlm.nih.gov/nucleotide/MW009028.1?report=genbank&log$=nucltop&blast_rank=1&RID=7EPGZW2D016) | 990 | 100.00% |
|  |  |  |  | *Alternaria alternata* | [MW009024.1](https://www.ncbi.nlm.nih.gov/nucleotide/MW009024.1?report=genbank&log$=nucltop&blast_rank=3&RID=7EPGZW2D016) | 985 | 99.81% |
| 28 | Jan | FDP | OM237141 | [*Penicillium concavorugulosum*](https://blast.ncbi.nlm.nih.gov/Blast.cgi) | [MK841454.1](https://www.ncbi.nlm.nih.gov/nucleotide/MK841454.1?report=genbank&log$=nucltop&blast_rank=1&RID=EUYR58MB013) | 1020 | 98.95% |
| 29 | Jan | FDP | OM237142 | *Penicillium concavorugulosum* | [MK841454.1](https://www.ncbi.nlm.nih.gov/nucleotide/MK841454.1?report=genbank&log$=nucltop&blast_rank=1&RID=7ER2KU31016) | 946 | 97.99% |
| 30 | Jan | FDP | OM237143 | *Leptosphaeria* sp. | [MH550512.1](https://www.ncbi.nlm.nih.gov/nucleotide/MH550512.1?report=genbank&log$=nucltop&blast_rank=1&RID=7ESASWXC016) | 970 | 99.81% |
|  |  |  |  | *Leptosphaeria sclerotioides* | [MK764997.1](https://www.ncbi.nlm.nih.gov/nucleotide/MK764997.1?report=genbank&log$=nucltop&blast_rank=3&RID=7ESASWXC016) | 968 | 99.07% |
| 31 | Jan | FDP | OM237144 | *Talaromyces stollii* | [MN515359.1](https://www.ncbi.nlm.nih.gov/nucleotide/MN515359.1?report=genbank&log$=nucltop&blast_rank=1&RID=7ESHHBJM01R) | 996 | 99.63% |
| 32 | Jan | FDP | OM237145 | Fungal sp. | [KU977335.1](https://www.ncbi.nlm.nih.gov/nucleotide/KU977335.1?report=genbank&log$=nucltop&blast_rank=1&RID=7ET5T8X6016) | 1026 | 99.82% |
|  |  |  |  | [*Aspergillus niger*](https://blast.ncbi.nlm.nih.gov/Blast.cgi) | [MG647867.1](https://www.ncbi.nlm.nih.gov/nucleotide/MG647867.1?report=genbank&log$=nucltop&blast_rank=2&RID=7ET5T8X6016) | 1024 | 99.82% |
| 33 | Jan | FDP | OM237146 | *Alternaria alternata* | [MW009043.1](https://www.ncbi.nlm.nih.gov/nucleotide/MW009043.1?report=genbank&log$=nucltop&blast_rank=1&RID=7ETK6TMJ01R) | 885 | 96.80% |
|  |  |  |  | *Alternaria alternata* | [KY694475.1](https://www.ncbi.nlm.nih.gov/nucleotide/KY694475.1?report=genbank&log$=nucltop&blast_rank=3&RID=7ETK6TMJ01R) | 883 | 96.15% |
| 34 | Jan | FDP | OM237147 | *Chaetomium globosum* | [MN526595.1](https://www.ncbi.nlm.nih.gov/nucleotide/MN526595.1?report=genbank&log$=nucltop&blast_rank=1&RID=7ETU313Y013) | 983 | 99.27% |
| 35 | Jan | FDP | OM237148 | [Uncultured fungus](https://blast.ncbi.nlm.nih.gov/Blast.cgi) | [HG328163.1](https://www.ncbi.nlm.nih.gov/nucleotide/HG328163.1?report=genbank&log$=nucltop&blast_rank=1&RID=EK4F4KVD013) | 1033 | 99.47% |
|  |  |  |  | [*Cephalotrichum nanum*](https://blast.ncbi.nlm.nih.gov/Blast.cgi) | [LC177640.1](https://www.ncbi.nlm.nih.gov/nucleotide/LC177640.1?report=genbank&log$=nucltop&blast_rank=2&RID=EK4F4KVD013) | 1027 | 99.30% |
| 37 | Jan | PS | OM237149 | *Pleosporales* sp. | [KR909181.1](https://www.ncbi.nlm.nih.gov/nucleotide/KR909181.1?report=genbank&log$=nucltop&blast_rank=1&RID=7EU03K5C016) | 966 | 99.44% |
|  |  |  |  | *Teichospora kingiae* | [NR_154656.1](https://www.ncbi.nlm.nih.gov/nucleotide/NR_154656.1?report=genbank&log$=nucltop&blast_rank=2&RID=7EU03K5C016) | 872 | 97.11% |
| 38 | Jan | PS | OM237150 | Uncultured fungus | [KX515758.1](https://www.ncbi.nlm.nih.gov/nucleotide/KX515758.1?report=genbank&log$=nucltop&blast_rank=1&RID=7EU7RHRC01R) | 953 | 99.43% |
|  |  |  |  | *Torula mackenziei* | [MW723058.1](https://www.ncbi.nlm.nih.gov/nucleotide/MW723058.1?report=genbank&log$=nucltop&blast_rank=3&RID=7EU7RHRC01R) | 931 | 99.61% |
| 39 | Jan | PS | OM237151 | *Lachancea lanzarotensis* | [KY076618.1](https://www.ncbi.nlm.nih.gov/nucleotide/KY076618.1?report=genbank&log$=nucltop&blast_rank=1&RID=7EUEEFEV016) | 1024 | 95.64% |
| 40 | Jan | CDO | OM237152 | *Periconia* sp. | [MG813167.1](https://www.ncbi.nlm.nih.gov/nucleotide/MG813167.1?report=genbank&log$=nucltop&blast_rank=1&RID=7EUKV2XG016) | 917 | 99.80% |
|  |  |  |  | *Periconia neobrittanica* | [NR_166344.1](https://www.ncbi.nlm.nih.gov/nucleotide/NR_166344.1?report=genbank&log$=nucltop&blast_rank=2&RID=7EUKV2XG016) | 909 | 99.60% |
| 41 | Jan | CDP | OM237153 | [Uncultured fungus](https://blast.ncbi.nlm.nih.gov/Blast.cgi) | [KC884377.1](https://www.ncbi.nlm.nih.gov/nucleotide/KC884377.1?report=genbank&log$=nucltop&blast_rank=1&RID=BYAMU7XA016) | 865 | 99.58% |
|  |  |  |  | [*Alternaria alternata*](https://blast.ncbi.nlm.nih.gov/Blast.cgi) | [MW723786.1](https://www.ncbi.nlm.nih.gov/nucleotide/MW723786.1?report=genbank&log$=nucltop&blast_rank=2&RID=BYAMU7XA016) | 865 | 96.41% |
| 42 | Jan | 5CO | OM237154 | *Arthrinium marii* | [MG649253.1](https://www.ncbi.nlm.nih.gov/nucleotide/MG649253.1?report=genbank&log$=nucltop&blast_rank=1&RID=7EUVFNHK01R) | 1072 | 99.49% |
| 43 | Jan | 5CO | OM237155 | *Cladosporium* sp. | [KY022751.1](https://www.ncbi.nlm.nih.gov/nucleotide/KY022751.1?report=genbank&log$=nucltop&blast_rank=1&RID=7EVE845401R) | 941 | 99.42% |
|  |  |  |  | *Cladosporium halotolerans* | [MH931821.1](https://www.ncbi.nlm.nih.gov/nucleotide/MH931821.1?report=genbank&log$=nucltop&blast_rank=2&RID=7EVE845401R) | 939 | 99.23% |
| 44 | Jan | 5CO | OM237156 | *Cladosporium cladosporioides* | [MT786364.1](https://www.ncbi.nlm.nih.gov/nucleotide/MT786364.1?report=genbank&log$=nucltop&blast_rank=1&RID=7EVM1UHG01R) | 946 | 99.61% |
| 45 | Jan | 5CO | OM237157 | Uncultured fungus | [KX515758.1](https://www.ncbi.nlm.nih.gov/nucleotide/KX515758.1?report=genbank&log$=nucltop&blast_rank=1&RID=7EVYH4TF01R) | 957 | 99.43% |
|  |  |  |  | *Torula caligans* | [JX156379.1](https://www.ncbi.nlm.nih.gov/nucleotide/JX156379.1?report=genbank&log$=nucltop&blast_rank=3&RID=7EVYH4TF01R) | 935 | 98.86% |
| 46 | Jan | 3CP | OM237158 | *Arthrinium saccharicola* | [KF144920.1](https://www.ncbi.nlm.nih.gov/nucleotide/KF144920.1?report=genbank&log$=nucltop&blast_rank=1&RID=7EW4Y1NR01R) | 1018 | 99.29% |
| 47 | Jan | 3CP | OM237159 | *Arthrinium saccharicola* | [KF144920.1](https://www.ncbi.nlm.nih.gov/nucleotide/KF144920.1?report=genbank&log$=nucltop&blast_rank=1&RID=7FHWMMNH013) | 1035 | 99.82% |
| 48 | Jan | FDP | OM237160 | *Talaromyces funiculosus* | [MK952427.1](https://www.ncbi.nlm.nih.gov/nucleotide/MK952427.1?report=genbank&log$=nucltop&blast_rank=1&RID=7FJ5BBTY013) | 992 | 99.63% |
| 49 | Jan | FDP | OM237161 | *Talaromyces verruculosus* | [MW081280.1](https://www.ncbi.nlm.nih.gov/nucleotide/MW081280.1?report=genbank&log$=nucltop&blast_rank=1&RID=7FJJUBB2013) | 1000 | 99.63% |
| 50 | Jan | PS | OM237162 | [*Alternaria* sp.](https://blast.ncbi.nlm.nih.gov/Blast.cgi) | [MN634443.1](https://www.ncbi.nlm.nih.gov/nucleotide/MN634443.1?report=genbank&log$=nucltop&blast_rank=1&RID=BYASJXNT013) | 891 | 100.00% |
|  |  |  |  | [*Alternaria alternata*](https://blast.ncbi.nlm.nih.gov/Blast.cgi) | [MN589682.1](https://www.ncbi.nlm.nih.gov/nucleotide/MN589682.1?report=genbank&log$=nucltop&blast_rank=3&RID=BYASJXNT013) | 891 | 100.00% |
| 51 | Jan | 5CO | OM237163 | [*Cladosporium asperulatum*](https://blast.ncbi.nlm.nih.gov/Blast.cgi) | [MN220523.1](https://www.ncbi.nlm.nih.gov/nucleotide/MN220523.1?report=genbank&log$=nucltop&blast_rank=1&RID=BYAVYW76016) | 839 | 96.46% |
| 52 | Jan | 5CO | OM237164 | [*Cladosporium cladosporioides*](https://blast.ncbi.nlm.nih.gov/Blast.cgi) | [MH345929.1](https://www.ncbi.nlm.nih.gov/nucleotide/MH345929.1?report=genbank&log$=nucltop&blast_rank=1&RID=BYAZKF0Y016) | 926 | 99.41% |
| 53 | Jan | 5CO | OM237165 | [*Talaromyces* sp.](https://blast.ncbi.nlm.nih.gov/Blast.cgi) | [MG745316.1](https://www.ncbi.nlm.nih.gov/nucleotide/MG745316.1?report=genbank&log$=nucltop&blast_rank=1&RID=BYB331R4016) | 929 | 98.67% |
|  |  |  |  | [*Talaromyces verruculosus*](https://blast.ncbi.nlm.nih.gov/Blast.cgi) | [MN077562.1](https://www.ncbi.nlm.nih.gov/nucleotide/MN077562.1?report=genbank&log$=nucltop&blast_rank=2&RID=BYB331R4016) | 926 | 98.12% |
| 54 | Jan | CDP | OM237166 | [*Hansfordia* sp.](https://blast.ncbi.nlm.nih.gov/Blast.cgi) | [KF986412.1](https://www.ncbi.nlm.nih.gov/nucleotide/KF986412.1?report=genbank&log$=nucltop&blast_rank=1&RID=BYB6G3NJ016) | 830 | 98.11% |
|  |  |  |  | [*Dicyma olivacea*](https://blast.ncbi.nlm.nih.gov/Blast.cgi) | [KF893287.1](https://www.ncbi.nlm.nih.gov/nucleotide/KF893287.1?report=genbank&log$=nucltop&blast_rank=3&RID=BYB6G3NJ016) | 822 | 98.29% |
| 55 | Jan | CDP | OM237167 | *Aureobasidium pullulans* | [HQ267771.1](https://www.ncbi.nlm.nih.gov/nucleotide/HQ267771.1?report=genbank&log$=nucltop&blast_rank=1&RID=EUZ8A9BZ013) | 990 | 98.92% |
| 1 | Feb | FDP | OM237168 | [*Alternaria compacta*](https://blast.ncbi.nlm.nih.gov/Blast.cgi) | [MW009019.1](https://www.ncbi.nlm.nih.gov/nucleotide/MW009019.1?report=genbank&log$=nucltop&blast_rank=1&RID=BYBTABFW013) | 983 | 99.27% |
| 2 | Feb | FDP | OM237169 | [*Alternaria alternata*](https://blast.ncbi.nlm.nih.gov/Blast.cgi) | [MN826219.1](https://www.ncbi.nlm.nih.gov/nucleotide/MN826219.1?report=genbank&log$=nucltop&blast_rank=1&RID=BYBX459D013) | 998 | 100.00% |
| 3 | Feb | FDP | OM237170 | [*Alternaria* sp.](https://blast.ncbi.nlm.nih.gov/Blast.cgi) | [MK649973.1](https://www.ncbi.nlm.nih.gov/nucleotide/MK649973.1?report=genbank&log$=nucltop&blast_rank=1&RID=BYC1JZCH013) | 917 | 98.11% |
|  |  |  |  | [*Alternaria alternata*](https://blast.ncbi.nlm.nih.gov/Blast.cgi) | [MN826219.1](https://www.ncbi.nlm.nih.gov/nucleotide/MN826219.1?report=genbank&log$=nucltop&blast_rank=2&RID=BYC1JZCH013) | 917 | 98.11% |
| 4 | Feb | 5CP | OM237171 | [*Cladosporium cladosporioides*](https://blast.ncbi.nlm.nih.gov/Blast.cgi) | [KX639814.1](https://www.ncbi.nlm.nih.gov/nucleotide/KX639814.1?report=genbank&log$=nucltop&blast_rank=1&RID=BYC4KMMF016) | 928 | 99.41% |
| 5 | Feb | 5CP | OM237172 | [Uncultured *Dothiorella*](https://blast.ncbi.nlm.nih.gov/Blast.cgi) | [MG462863.1](https://www.ncbi.nlm.nih.gov/nucleotide/MG462863.1?report=genbank&log$=nucltop&blast_rank=1&RID=BYCCPZNF01R) | 920 | 99.41% |
|  |  |  |  | [*Nothophoma quercina*](https://blast.ncbi.nlm.nih.gov/Blast.cgi) | [KX099648.1](https://www.ncbi.nlm.nih.gov/nucleotide/KX099648.1?report=genbank&log$=nucltop&blast_rank=3&RID=BYCCPZNF01R) | 918 | 99.22% |
| 6 | Feb | 5CP | OM237173 | [*Penicillium brevicompactum*](https://blast.ncbi.nlm.nih.gov/Blast.cgi) | [KX426968.1](https://www.ncbi.nlm.nih.gov/nucleotide/KX426968.1?report=genbank&log$=nucltop&blast_rank=1&RID=BYCKURA2013) | 859 | 98.56% |
| 7 | Feb | 5CO | OM237174 | [*Dothideomycetes* sp.](https://blast.ncbi.nlm.nih.gov/Blast.cgi) | [KX641980.1](https://www.ncbi.nlm.nih.gov/nucleotide/KX641980.1?report=genbank&log$=nucltop&blast_rank=1&RID=BYCR5YC2016) | 933 | 99.23% |
|  |  |  |  | [*Cladosporium cladosporioides*](https://blast.ncbi.nlm.nih.gov/Blast.cgi) | [KF876823.1](https://www.ncbi.nlm.nih.gov/nucleotide/KF876823.1?report=genbank&log$=nucltop&blast_rank=3&RID=BYCR5YC2016) | 933 | 99.61% |
| 8 | Feb | LIB | OM237175 | [*Cladosporium cladosporioides*](https://blast.ncbi.nlm.nih.gov/Blast.cgi) | [MT043875.1](https://www.ncbi.nlm.nih.gov/nucleotide/MT043875.1?report=genbank&log$=nucltop&blast_rank=1&RID=BYCUD1Y0016) | 909 | 99.40% |
| 9 | Feb | LIB | OM237176 | [*Papiliotrema aurea*](https://blast.ncbi.nlm.nih.gov/Blast.cgi) | [MK592827.1](https://www.ncbi.nlm.nih.gov/nucleotide/MK592827.1?report=genbank&log$=nucltop&blast_rank=1&RID=BYD3V097013) | 891 | 99.00% |
| 10 | Feb | LIB | OM237177 | [*Alternaria* sp.](https://blast.ncbi.nlm.nih.gov/Blast.cgi) | [KJ541475.1](https://www.ncbi.nlm.nih.gov/nucleotide/KJ541475.1?report=genbank&log$=nucltop&blast_rank=1&RID=BYD7CWRW013) | 904 | 99.60% |
|  |  |  |  | [*Alternaria alternata*](https://blast.ncbi.nlm.nih.gov/Blast.cgi) | [MN249500.1](https://www.ncbi.nlm.nih.gov/nucleotide/MN249500.1?report=genbank&log$=nucltop&blast_rank=2&RID=BYD7CWRW013) | 902 | 99.60% |
| 11 | Feb | CDO | OM237178 | [*Alternaria alternata*](https://blast.ncbi.nlm.nih.gov/Blast.cgi) | [KF380821.1](https://www.ncbi.nlm.nih.gov/nucleotide/KF380821.1?report=genbank&log$=nucltop&blast_rank=1&RID=BYDAH309013) | 985 | 99.63% |
| 12 | Feb | CDP | OM237179 | [*Alternaria alternata*](https://blast.ncbi.nlm.nih.gov/Blast.cgi) | [MN826219.1](https://www.ncbi.nlm.nih.gov/nucleotide/MN826219.1?report=genbank&log$=nucltop&blast_rank=1&RID=BYDDPD18013) | 987 | 99.63% |
| 13 | Feb | CDP | OM237180 | [*Epicoccum nigrum*](https://blast.ncbi.nlm.nih.gov/Blast.cgi) | [MN089646.1](https://www.ncbi.nlm.nih.gov/nucleotide/MN089646.1?report=genbank&log$=nucltop&blast_rank=1&RID=BYDGXWH3016) | 917 | 99.41% |
| 14 | Feb | 3CP | OM237181 | [*Alternaria alternata*](https://blast.ncbi.nlm.nih.gov/Blast.cgi) | [MN826219.1](https://www.ncbi.nlm.nih.gov/nucleotide/MN826219.1?report=genbank&log$=nucltop&blast_rank=1&RID=BYDTDNPM013) | 989 | 99.81% |
| 15 | Feb | 3CP | OM237182 | [*Alternaria alternata*](https://blast.ncbi.nlm.nih.gov/Blast.cgi) | [MN989195.1](https://www.ncbi.nlm.nih.gov/nucleotide/MN989195.1?report=genbank&log$=nucltop&blast_rank=1&RID=BYDWRN4H016) | 983 | 100.00% |
| 16 | Feb | 3CP | OM237183 | [*Alternaria* sp.](https://blast.ncbi.nlm.nih.gov/Blast.cgi) | [MG211082.1](https://www.ncbi.nlm.nih.gov/nucleotide/MG211082.1?report=genbank&log$=nucltop&blast_rank=1&RID=BYE4C5EE016) | 989 | 99.81% |
|  |  |  |  | [*Alternaria alternata*](https://blast.ncbi.nlm.nih.gov/Blast.cgi) | [MN989195.1](https://www.ncbi.nlm.nih.gov/nucleotide/MN989195.1?report=genbank&log$=nucltop&blast_rank=3&RID=BYE4C5EE016) | 983 | 100.00% |
| 17 | Feb | 3CP | OM237184 | [*Chaetomium globosum*](https://blast.ncbi.nlm.nih.gov/Blast.cgi) | [MN173145.1](https://www.ncbi.nlm.nih.gov/nucleotide/MN173145.1?report=genbank&log$=nucltop&blast_rank=1&RID=BYE7F229016) | 885 | 98.99% |
| 18 | Feb | 3CP | OM237185 | [*Phoma fungicola*](https://blast.ncbi.nlm.nih.gov/Blast.cgi) | [KF293780.1](https://www.ncbi.nlm.nih.gov/nucleotide/KF293780.1?report=genbank&log$=nucltop&blast_rank=1&RID=BYEB1ACX013) | 931 | 99.61% |
| 19 | Feb | 3CP | OM237186 | [*Alternaria alternata*](https://blast.ncbi.nlm.nih.gov/Blast.cgi) | [MN826219.1](https://www.ncbi.nlm.nih.gov/nucleotide/MN826219.1?report=genbank&log$=nucltop&blast_rank=1&RID=BYEE3UT8013) | 990 | 100.00% |
| 20 | Feb | CDO | OM237187 | [*Ascomycete* sp.](https://blast.ncbi.nlm.nih.gov/Blast.cgi) | [EU848215.1](https://www.ncbi.nlm.nih.gov/nucleotide/EU848215.1?report=genbank&log$=nucltop&blast_rank=1&RID=BYEH9W86016) | 880 | 97.67% |
| 21 | Feb | CDP | OM237188 | [*Alternaria* sp.](https://blast.ncbi.nlm.nih.gov/Blast.cgi) | [MW008994.1](https://www.ncbi.nlm.nih.gov/nucleotide/MW008994.1?report=genbank&log$=nucltop&blast_rank=1&RID=BYEN7B7P013) | 979 | 99.63% |
|  |  |  |  | [*Alternaria japonica*](https://blast.ncbi.nlm.nih.gov/Blast.cgi) | [MF462306.1](https://www.ncbi.nlm.nih.gov/nucleotide/MF462306.1?report=genbank&log$=nucltop&blast_rank=4&RID=BYEN7B7P013) | 977 | 100.00% |
| 22 | Feb | CDP | OM237189 | [*Alternaria alternata*](https://blast.ncbi.nlm.nih.gov/Blast.cgi) | [MN826219.1](https://www.ncbi.nlm.nih.gov/nucleotide/MN826219.1?report=genbank&log$=nucltop&blast_rank=1&RID=BYETGD6M013) | 989 | 99.63% |
| 23 | Feb | 3CP | OM237190 | [Uncultured fungus](https://blast.ncbi.nlm.nih.gov/Blast.cgi) | [KX515758.1](https://www.ncbi.nlm.nih.gov/nucleotide/KX515758.1?report=genbank&log$=nucltop&blast_rank=1&RID=BYEX5FKY013) | 931 | 98.86% |
|  |  |  |  | [*Torula caligans*](https://blast.ncbi.nlm.nih.gov/Blast.cgi) | [JX156379.1](https://www.ncbi.nlm.nih.gov/nucleotide/JX156379.1?report=genbank&log$=nucltop&blast_rank=2&RID=BYEX5FKY013) | 929 | 98.85% |
| 24 | Feb | 3CP | OM237191 | [*Aspergillus proliferans*](https://blast.ncbi.nlm.nih.gov/Blast.cgi) | [KX696376.1](https://www.ncbi.nlm.nih.gov/nucleotide/KX696376.1?report=genbank&log$=nucltop&blast_rank=1&RID=BYF09ZF1016) | 900 | 99.20% |
| 1 | Mar | 3CP | OM237192 | [Fungal sp.](https://blast.ncbi.nlm.nih.gov/Blast.cgi) | [MW603305.1](https://www.ncbi.nlm.nih.gov/nucleotide/MW603305.1?report=genbank&log$=nucltop&blast_rank=1&RID=C0YJ1RH3016) | 976 | 99.44% |
|  |  |  |  | [*Talaromyces verruculosus*](https://blast.ncbi.nlm.nih.gov/Blast.cgi) | [MW958046.1](https://www.ncbi.nlm.nih.gov/nucleotide/MW958046.1?report=genbank&log$=nucltop&blast_rank=2&RID=C0YJ1RH3016) | 974 | 99.08% |
| 2 | Mar | 3CP | OM237193 | [*Didymella* sp.](https://blast.ncbi.nlm.nih.gov/Blast.cgi) | [MG198901.1](https://www.ncbi.nlm.nih.gov/nucleotide/MG198901.1?report=genbank&log$=nucltop&blast_rank=1&RID=C0YRR5FW016) | 1413 | 99.22% |
|  |  |  |  | [*Ascochyta medicaginicola*](https://blast.ncbi.nlm.nih.gov/Blast.cgi) | [KX064990.1](https://www.ncbi.nlm.nih.gov/nucleotide/KX064990.1?report=genbank&log$=nucltop&blast_rank=3&RID=C0YRR5FW016) | 922 | 99.80% |
| 3 | Mar | 3CP | OM237194 | [*Cladosporium cladosporioides*](https://blast.ncbi.nlm.nih.gov/Blast.cgi) | [KX639814.1](https://www.ncbi.nlm.nih.gov/nucleotide/KX639814.1?report=genbank&log$=nucltop&blast_rank=1&RID=C0YVWTR7016) | 928 | 99.41% |
| 4 | Mar | 3CP | OM237195 | [*Epicoccum nigrum*](https://blast.ncbi.nlm.nih.gov/Blast.cgi) | [KM877476.1](https://www.ncbi.nlm.nih.gov/nucleotide/KM877476.1?report=genbank&log$=nucltop&blast_rank=1&RID=C10PEYJS013) | 922 | 99.80% |
| 5 | Mar | 3CP | OM237196 | [*Penicillium polonicum*](https://blast.ncbi.nlm.nih.gov/Blast.cgi) | [JQ082508.1](https://www.ncbi.nlm.nih.gov/nucleotide/JQ082508.1?report=genbank&log$=nucltop&blast_rank=1&RID=EUZK1XWD013) | 977 | 98.05% |
| 7 | Mar | PS | OM237197 | [Uncultured *Cladosporium*](https://blast.ncbi.nlm.nih.gov/Blast.cgi) | [LN833532.1](https://www.ncbi.nlm.nih.gov/nucleotide/LN833532.1?report=genbank&log$=nucltop&blast_rank=1&RID=C10UCNFN016) | 939 | 99.42% |
|  |  |  |  | [*Cladosporium cladosporioides*](https://blast.ncbi.nlm.nih.gov/Blast.cgi) | [MT786364.1](https://www.ncbi.nlm.nih.gov/nucleotide/MT786364.1?report=genbank&log$=nucltop&blast_rank=2&RID=C10UCNFN016) | 937 | 99.23% |
| 8 | Mar | PS | OM237198 | [*Didymellaceae* sp.](https://blast.ncbi.nlm.nih.gov/Blast.cgi) | [KR709032.1](https://www.ncbi.nlm.nih.gov/nucleotide/KR709032.1?report=genbank&log$=nucltop&blast_rank=1&RID=C1114TPP01R) | 976 | 99.44% |
| 9 | Mar | PS | OM237199 | [*Alternaria alternata*](https://blast.ncbi.nlm.nih.gov/Blast.cgi) | [KU324792.1](https://www.ncbi.nlm.nih.gov/nucleotide/KU324792.1?report=genbank&log$=nucltop&blast_rank=1&RID=C117KUJ901R) | 987 | 99.81% |
| 10 | Mar | PS | OM237200 | [*Penicillium polonicum*](https://blast.ncbi.nlm.nih.gov/Blast.cgi) | [JQ082508.1](https://www.ncbi.nlm.nih.gov/nucleotide/JQ082508.1?report=genbank&log$=nucltop&blast_rank=1&RID=C11B5NDC016) | 922 | 99.41% |
| 11 | Mar | PS | OM237201 | [*Cladosporium cladosporioides*](https://blast.ncbi.nlm.nih.gov/Blast.cgi) | [MT367212.1](https://www.ncbi.nlm.nih.gov/nucleotide/MT367212.1?report=genbank&log$=nucltop&blast_rank=1&RID=C11EMCJ4016) | 928 | 100.00% |
| 12 | Mar | PS | OM237202 | [*Cladosporium cladosporioides*](https://blast.ncbi.nlm.nih.gov/Blast.cgi) | [MK761055.1](https://www.ncbi.nlm.nih.gov/nucleotide/MK761055.1?report=genbank&log$=nucltop&blast_rank=1&RID=C11P5FEP013) | 928 | 100.00% |
| 13 | Mar | PS | OM237203 | [*Fusarium equiseti*](https://blast.ncbi.nlm.nih.gov/Blast.cgi) | [MW362787.1](https://www.ncbi.nlm.nih.gov/nucleotide/MW362787.1?report=genbank&log$=nucltop&blast_rank=1&RID=ECVJ430Y01R) | 915 | 100.00% |
| 14 | Mar | PS | OM237204 | [*Cladosporium* sp.](https://blast.ncbi.nlm.nih.gov/Blast.cgi) | [MK640598.1](https://www.ncbi.nlm.nih.gov/nucleotide/MK640598.1?report=genbank&log$=nucltop&blast_rank=1&RID=C11U0XRP01R) | 924 | 99.22% |
|  |  |  |  | [*Cladosporium cladosporioides*](https://blast.ncbi.nlm.nih.gov/Blast.cgi) | [MH425309.1](https://www.ncbi.nlm.nih.gov/nucleotide/MH425309.1?report=genbank&log$=nucltop&blast_rank=2&RID=C11U0XRP01R) | 924 | 99.22% |
| 15 | Mar | PS | OM237205 | [*Rhodotorula diobovata*](https://blast.ncbi.nlm.nih.gov/Blast.cgi) | [MH481699.1](https://www.ncbi.nlm.nih.gov/nucleotide/MH481699.1?report=genbank&log$=nucltop&blast_rank=1&RID=C11YF0WX013) | 942 | 99.42% |
| 16 | Mar | PS | OM237206 | [*Alternaria* sp.](https://blast.ncbi.nlm.nih.gov/Blast.cgi) | [MW581345.1](https://www.ncbi.nlm.nih.gov/nucleotide/MW581345.1?report=genbank&log$=nucltop&blast_rank=1&RID=C122951D016) | 990 | 99.27% |
|  |  |  |  | [*Alternaria tenuissima*](https://blast.ncbi.nlm.nih.gov/Blast.cgi) | [MW008969.1](https://www.ncbi.nlm.nih.gov/nucleotide/MW008969.1?report=genbank&log$=nucltop&blast_rank=2&RID=C122951D016) | 990 | 99.63% |
| 17 | Mar | PS | OM237207 | [*Alternaria* sp.](https://blast.ncbi.nlm.nih.gov/Blast.cgi) | [MW581345.1](https://www.ncbi.nlm.nih.gov/nucleotide/MW581345.1?report=genbank&log$=nucltop&blast_rank=1&RID=C12D1ST0016) | 990 | 99.27% |
|  |  |  |  | [*Alternaria tenuissima*](https://blast.ncbi.nlm.nih.gov/Blast.cgi) | [MW008969.1](https://www.ncbi.nlm.nih.gov/nucleotide/MW008969.1?report=genbank&log$=nucltop&blast_rank=2&RID=C12D1ST0016) | 990 | 99.63% |
| 18 | Mar | PS | OM237208 | [*Alternaria tenuissima*](https://blast.ncbi.nlm.nih.gov/Blast.cgi) | [MW009025.1](https://www.ncbi.nlm.nih.gov/nucleotide/MW009025.1?report=genbank&log$=nucltop&blast_rank=1&RID=C125Z9XD013) | 981 | 99.45% |
| 19 | Mar | PS | OM237209 | [Uncultured *Talaromyces*](https://blast.ncbi.nlm.nih.gov/Blast.cgi) | [KT900531.1](https://www.ncbi.nlm.nih.gov/nucleotide/KT900531.1?report=genbank&log$=nucltop&blast_rank=1&RID=C12HT0UA013) | 987 | 99.27% |
|  |  |  |  | [*Talaromyces verruculosus*](https://blast.ncbi.nlm.nih.gov/Blast.cgi) | [MN429191.1](https://www.ncbi.nlm.nih.gov/nucleotide/MN429191.1?report=genbank&log$=nucltop&blast_rank=3&RID=C12HT0UA013) | 981 | 99.63% |
| 20 | Mar | FDO | OM237210 | [*Talaromyces verruculosus*](https://blast.ncbi.nlm.nih.gov/Blast.cgi) | [MW958043.1](https://www.ncbi.nlm.nih.gov/nucleotide/MW958043.1?report=genbank&log$=nucltop&blast_rank=1&RID=C131R6S2016) | 992 | 100.00% |
| 21 | Mar | FDO | OM237211 | [*Talaromyces purpureogenus*](https://blast.ncbi.nlm.nih.gov/Blast.cgi) | [MN121629.1](https://www.ncbi.nlm.nih.gov/nucleotide/MN121629.1?report=genbank&log$=nucltop&blast_rank=1&RID=C139GNCU016) | 996 | 100.00% |
| 23 | Mar | CDP | OM237212 | [Fungal sp.](https://blast.ncbi.nlm.nih.gov/Blast.cgi) | [MG779600.1](https://www.ncbi.nlm.nih.gov/nucleotide/MG779600.1?report=genbank&log$=nucltop&blast_rank=1&RID=C13DZPTW013) | 929 | 100.00% |
|  |  |  |  | [*Didymella macrostoma*](https://blast.ncbi.nlm.nih.gov/Blast.cgi) | [MN944409.1](https://www.ncbi.nlm.nih.gov/nucleotide/MN944409.1?report=genbank&log$=nucltop&blast_rank=2&RID=C13DZPTW013) | 928 | 99.61% |
| 24 | Mar | LIB | OM237213 | [*Talaromyces funiculosus*](https://blast.ncbi.nlm.nih.gov/Blast.cgi) | [MW958047.1](https://www.ncbi.nlm.nih.gov/nucleotide/MW958047.1?report=genbank&log$=nucltop&blast_rank=1&RID=C1A40R06016) | 990 | 99.45% |
| 25 | Mar | LIB | OM237214 | [*Cladosporium cladosporioides*](https://blast.ncbi.nlm.nih.gov/Blast.cgi) | [MW556429.1](https://www.ncbi.nlm.nih.gov/nucleotide/MW556429.1?report=genbank&log$=nucltop&blast_rank=1&RID=C1A6Z9Y0013) | 944 | 99.81% |
| 26 | Mar | CDO | OM237215 | [Fungal sp.](https://blast.ncbi.nlm.nih.gov/Blast.cgi) | [MT112978.1](https://www.ncbi.nlm.nih.gov/nucleotide/MT112978.1?report=genbank&log$=nucltop&blast_rank=1&RID=C1A9556U013) | 939 | 99.80% |
|  |  |  |  | [*Cladosporium allicinum*](https://blast.ncbi.nlm.nih.gov/Blast.cgi) | [MT974153.1](https://www.ncbi.nlm.nih.gov/nucleotide/MT974153.1?report=genbank&log$=nucltop&blast_rank=3&RID=C1A9556U013) | 937 | 99.80% |
| 27 | Mar | CDO | OM237216 | [*Talaromyces funiculosus*](https://blast.ncbi.nlm.nih.gov/Blast.cgi) | [MT072069.1](https://www.ncbi.nlm.nih.gov/nucleotide/MT072069.1?report=genbank&log$=nucltop&blast_rank=1&RID=C1AJN4SM016) | 992 | 99.63% |
| 28 | Mar | 3CO | OM237217 | [*Botrytis cinerea*](https://blast.ncbi.nlm.nih.gov/Blast.cgi) | [MK748141.1](https://www.ncbi.nlm.nih.gov/nucleotide/MK748141.1?report=genbank&log$=nucltop&blast_rank=1&RID=C1ASJDK8016) | 920 | 99.60% |
| 29 | Mar | 5CP | OM237218 | [*Phoma* sp.](https://blast.ncbi.nlm.nih.gov/Blast.cgi) | [JX896660.1](https://www.ncbi.nlm.nih.gov/nucleotide/JX896660.1?report=genbank&log$=nucltop&blast_rank=1&RID=C1AVS52M013) | 926 | 99.80% |
|  |  |  |  | [*Peyronellaea glomerata*](https://blast.ncbi.nlm.nih.gov/Blast.cgi) | [KM014745.1](https://www.ncbi.nlm.nih.gov/nucleotide/KM014745.1?report=genbank&log$=nucltop&blast_rank=2&RID=C1AVS52M013) | 920 | 99.60% |
| 30 | Mar | 5CP | OM237219 | [*Talaromyces funiculosus*](https://blast.ncbi.nlm.nih.gov/Blast.cgi) | [MW958047.1](https://www.ncbi.nlm.nih.gov/nucleotide/MW958047.1?report=genbank&log$=nucltop&blast_rank=1&RID=C1B01ETY013) | 987 | 99.81% |
| 31 | Mar | 5CP | OM237220 | [*Pleosporales* sp.](https://blast.ncbi.nlm.nih.gov/Blast.cgi) | [KX379244.1](https://www.ncbi.nlm.nih.gov/nucleotide/KX379244.1?report=genbank&log$=nucltop&blast_rank=1&RID=C1B6TEJD016) | 944 | 99.81% |
|  |  |  |  | [*Cladosporium tenuissimum*](https://blast.ncbi.nlm.nih.gov/Blast.cgi) | [MN429197.1](https://www.ncbi.nlm.nih.gov/nucleotide/MN429197.1?report=genbank&log$=nucltop&blast_rank=2&RID=C1B6TEJD016) | 941 | 99.81% |
| 32 | Mar | 5CP | OM237221 | [*Talaromyces funiculosus*](https://blast.ncbi.nlm.nih.gov/Blast.cgi) | [MW958047.1](https://www.ncbi.nlm.nih.gov/nucleotide/MW958047.1?report=genbank&log$=nucltop&blast_rank=1&RID=C1BAF6EZ013) | 996 | 98.75% |
| 33 | Mar | 5CP | OM237222 | [*Talaromyces funiculosus*](https://blast.ncbi.nlm.nih.gov/Blast.cgi) | [MG873486.1](https://www.ncbi.nlm.nih.gov/nucleotide/MG873486.1?report=genbank&log$=nucltop&blast_rank=1&RID=C1BEBPXU013) | 996 | 100.00% |
| 34 | Mar | 5CP | OM237223 | [*Aureobasidium pullulans*](https://blast.ncbi.nlm.nih.gov/Blast.cgi) | [MN922096.1](https://www.ncbi.nlm.nih.gov/nucleotide/MN922096.1?report=genbank&log$=nucltop&blast_rank=1&RID=C1BGJWXD013) | 856 | 97.79% |
| 35 | Mar | 5CP | OM237224 | [*Alternaria alternata*](https://blast.ncbi.nlm.nih.gov/Blast.cgi) | [MW008988.1](https://www.ncbi.nlm.nih.gov/nucleotide/MW008988.1?report=genbank&log$=nucltop&blast_rank=1&RID=C1BJC0MK016) | 992 | 99.63% |
| 36 | Mar | 5CP | OM237225 | [*Hypoxylon macrocarpum*](https://blast.ncbi.nlm.nih.gov/Blast.cgi) | [MW567284.1](https://www.ncbi.nlm.nih.gov/nucleotide/MW567284.1?report=genbank&log$=nucltop&blast_rank=1&RID=ECVMP727016) | 1026 | 98.30% |
| 37 | Mar | 5CO | OM237226 | [Fungal sp.](https://blast.ncbi.nlm.nih.gov/Blast.cgi) | [MW603282.1](https://www.ncbi.nlm.nih.gov/nucleotide/MW603282.1?report=genbank&log$=nucltop&blast_rank=1&RID=C1BPERM3016) | 939 | 99.80% |
|  |  |  |  | [*Cladosporium cladosporioides*](https://blast.ncbi.nlm.nih.gov/Blast.cgi) | [MT367213.1](https://www.ncbi.nlm.nih.gov/nucleotide/MT367213.1?report=genbank&log$=nucltop&blast_rank=2&RID=C1BPERM3016) | 937 | 99.80% |
| 38 | Mar | 5CO | OM237227 | [*Talaromyces verruculosus*](https://blast.ncbi.nlm.nih.gov/Blast.cgi) | [MW958040.1](https://www.ncbi.nlm.nih.gov/nucleotide/MW958040.1?report=genbank&log$=nucltop&blast_rank=1&RID=C1BTPKWN013) | 990 | 99.45% |
| 39 | Mar | 5CO | OM237228 | [*Talaromyces rogersiae*](https://blast.ncbi.nlm.nih.gov/Blast.cgi) | [MT072067.1](https://www.ncbi.nlm.nih.gov/nucleotide/MT072067.1?report=genbank&log$=nucltop&blast_rank=1&RID=C1BW0H5Y016) | 1014 | 99.46% |
| 1 | Apr | 5CP | OM237229 | [*Cladosporium* sp.](https://blast.ncbi.nlm.nih.gov/Blast.cgi) | [KT826668.1](https://www.ncbi.nlm.nih.gov/nucleotide/KT826668.1?report=genbank&log$=nucltop&blast_rank=1&RID=ECVR2X1Z013) | 946 | 100.00% |
|  |  |  |  | [*Cladosporium allicinum*](https://blast.ncbi.nlm.nih.gov/Blast.cgi) | [MT974153.1](https://www.ncbi.nlm.nih.gov/nucleotide/MT974153.1?report=genbank&log$=nucltop&blast_rank=2&RID=ECVR2X1Z013) | 944 | 100.00% |
| 2 | Apr | 5CP | OM237230 | [*Alternaria alternata*](https://blast.ncbi.nlm.nih.gov/Blast.cgi) | [MW008970.1](https://www.ncbi.nlm.nih.gov/nucleotide/MW008970.1?report=genbank&log$=nucltop&blast_rank=1&RID=C1BZU3T7016) | 987 | 99.63% |
| 3 | Apr | 5CP | OM237231 | [*Penicillium expansum*](https://blast.ncbi.nlm.nih.gov/Blast.cgi) | [MH879148.1](https://www.ncbi.nlm.nih.gov/nucleotide/MH879148.1?report=genbank&log$=nucltop&blast_rank=1&RID=C1C272KC016) | 994 | 99.63% |
| 4 | Apr | 5CP | OM237232 | [*Cladosporium cladosporioides*](https://blast.ncbi.nlm.nih.gov/Blast.cgi) | [KF876823.1](https://www.ncbi.nlm.nih.gov/nucleotide/KF876823.1?report=genbank&log$=nucltop&blast_rank=1&RID=ECVTRR33016) | 942 | 99.42% |
| 5 | Apr | 5CO | OM237233 | [*Aureobasidium pullulans*](https://blast.ncbi.nlm.nih.gov/Blast.cgi) | [MZ133727.1](https://www.ncbi.nlm.nih.gov/nucleotide/MZ133727.1?report=genbank&log$=nucltop&blast_rank=1&RID=C1C49BES016) | 981 | 99.26% |
| 6 | Apr | 5CO | OM237234 | [*Alternaria* sp.](https://blast.ncbi.nlm.nih.gov/Blast.cgi) | [MW008953.1](https://www.ncbi.nlm.nih.gov/nucleotide/MW008953.1?report=genbank&log$=nucltop&blast_rank=1&RID=C1C7TY3C016) | 981 | 100.00% |
|  |  |  |  | [*Alternaria alternata*](https://blast.ncbi.nlm.nih.gov/Blast.cgi) | [MW008975.1](https://www.ncbi.nlm.nih.gov/nucleotide/MW008975.1?report=genbank&log$=nucltop&blast_rank=2&RID=C1C7TY3C016) | 979 | 99.63% |
| 7 | Apr | 5CO | OM237235 | [*Alternaria* sp.](https://blast.ncbi.nlm.nih.gov/Blast.cgi) | [MW009026.1](https://www.ncbi.nlm.nih.gov/nucleotide/MW009026.1?report=genbank&log$=nucltop&blast_rank=1&RID=C1CASZP4013) | 922 | 98.66% |
|  |  |  |  | [*Alternaria alternata*](https://blast.ncbi.nlm.nih.gov/Blast.cgi) | [KR709045.1](https://www.ncbi.nlm.nih.gov/nucleotide/KR709045.1?report=genbank&log$=nucltop&blast_rank=4&RID=C1CASZP4013) | 920 | 98.65% |
| 8 | Apr | CDO | OM237236 | [*Alternaria solani*](https://blast.ncbi.nlm.nih.gov/Blast.cgi) | [MW009039.1](https://www.ncbi.nlm.nih.gov/nucleotide/MW009039.1?report=genbank&log$=nucltop&blast_rank=1&RID=C1CG56KP016) | 989 | 99.81% |
| 9 | Apr | CDO | OM237237 | [*Alternaria compacta*](https://blast.ncbi.nlm.nih.gov/Blast.cgi) | [MW008978.1](https://www.ncbi.nlm.nih.gov/nucleotide/MW008978.1?report=genbank&log$=nucltop&blast_rank=1&RID=C1CJ57F0016) | 989 | 100.00% |
| 10 | Apr | CDO | OM237238 | [Fungal sp.](https://blast.ncbi.nlm.nih.gov/Blast.cgi) | [MW603421.1](https://www.ncbi.nlm.nih.gov/nucleotide/MW603421.1?report=genbank&log$=nucltop&blast_rank=1&RID=C1CNAVM5016) | 941 | 99.42% |
|  |  |  |  | [*Cladosporium cladosporioides*](https://blast.ncbi.nlm.nih.gov/Blast.cgi) | [MT367213.1](https://www.ncbi.nlm.nih.gov/nucleotide/MT367213.1?report=genbank&log$=nucltop&blast_rank=2&RID=C1CNAVM5016) | 939 | 99.42% |
| 11 | Apr | CDO | OM237239 | [*Cladosporium velox*](https://blast.ncbi.nlm.nih.gov/Blast.cgi) | [MK814794.1](https://www.ncbi.nlm.nih.gov/nucleotide/MK814794.1?report=genbank&log$=nucltop&blast_rank=1&RID=C5KMHNMM016) | 928 | 99.03% |
| 12 | Apr | CDO | OM237240 | [*Alternaria alternata*](https://blast.ncbi.nlm.nih.gov/Blast.cgi) | [MW008894.1](https://www.ncbi.nlm.nih.gov/nucleotide/MW008894.1?report=genbank&log$=nucltop&blast_rank=1&RID=C5KR8CJC016) | 983 | 99.81% |
| 13 | Apr | CDP | OM237241 | [Fungal sp.](https://blast.ncbi.nlm.nih.gov/Blast.cgi) | [MT112989.1](https://www.ncbi.nlm.nih.gov/nucleotide/MT112989.1?report=genbank&log$=nucltop&blast_rank=1&RID=C5KTA2AZ013) | 939 | 99.80% |
|  |  |  |  | [*Cladosporium cucumerinum*](https://blast.ncbi.nlm.nih.gov/Blast.cgi) | [KR912311.1](https://www.ncbi.nlm.nih.gov/nucleotide/KR912311.1?report=genbank&log$=nucltop&blast_rank=8&RID=C5KTA2AZ013) | 933 | 99.61% |
| 14 | Apr | CDP | OM237242 | [*Alternaria* sp.](https://blast.ncbi.nlm.nih.gov/Blast.cgi) | [MW008953.1](https://www.ncbi.nlm.nih.gov/nucleotide/MW008953.1?report=genbank&log$=nucltop&blast_rank=1&RID=C5KWSKWB016) | 983 | 99.63% |
|  |  |  |  | [*Alternaria alternata*](https://blast.ncbi.nlm.nih.gov/Blast.cgi) | [MW008975.1](https://www.ncbi.nlm.nih.gov/nucleotide/MW008975.1?report=genbank&log$=nucltop&blast_rank=4&RID=C5KWSKWB016) | 979 | 99.63% |
| 15 | Apr | LIB | OM237243 | [*Cladosporium herbarum*](https://blast.ncbi.nlm.nih.gov/Blast.cgi) | [KX611004.1](https://www.ncbi.nlm.nih.gov/nucleotide/KX611004.1?report=genbank&log$=nucltop&blast_rank=1&RID=C5M01MK6016) | 935 | 99.04% |
| 16 | Apr | LIB | OM237244 | [*Pithomyces chartarum*](https://blast.ncbi.nlm.nih.gov/Blast.cgi) | [MW898423.1](https://www.ncbi.nlm.nih.gov/nucleotide/MW898423.1?report=genbank&log$=nucltop&blast_rank=1&RID=C5M20X3K016) | 1035 | 99.47% |
| 17 | Apr | LIB | OM237245 | [*Schizophyllum commune*](https://blast.ncbi.nlm.nih.gov/Blast.cgi) | [MH483677.1](https://www.ncbi.nlm.nih.gov/nucleotide/MH483677.1?report=genbank&log$=nucltop&blast_rank=1&RID=C5M40PBA013) | 1088 | 99.34% |
| 18 | Apr | LIB | OM237246 | [Fungal sp.](https://blast.ncbi.nlm.nih.gov/Blast.cgi) | [KP893227.1](https://www.ncbi.nlm.nih.gov/nucleotide/KP893227.1?report=genbank&log$=nucltop&blast_rank=1&RID=C5M5X0F1016) | 976 | 99.26% |
|  |  |  |  | [*Chaetomium globosum*](https://blast.ncbi.nlm.nih.gov/Blast.cgi) | [LT906579.1](https://www.ncbi.nlm.nih.gov/nucleotide/LT906579.1?report=genbank&log$=nucltop&blast_rank=2&RID=C5M5X0F1016) | 974 | 99.26% |
| 19 | Apr | LIB | OM237247 | [*Naganishia albida*](https://blast.ncbi.nlm.nih.gov/Blast.cgi) | [KY238170.1](https://www.ncbi.nlm.nih.gov/nucleotide/KY238170.1?report=genbank&log$=nucltop&blast_rank=1&RID=C5M93Y8K016) | 1083 | 99.83% |
| 20 | Apr | 3CP | OM237248 | *Aspergillus flavus* | [AJ874128.1](https://www.ncbi.nlm.nih.gov/nucleotide/AJ874128.1?report=genbank&log$=nucltop&blast_rank=1&RID=EUZYHXRF013) | 970 | 96.22% |
| 21 | Apr | 3CP | OM237249 | [*Kwoniella* sp.](https://blast.ncbi.nlm.nih.gov/Blast.cgi) | [MT777412.1](https://www.ncbi.nlm.nih.gov/nucleotide/MT777412.1?report=genbank&log$=nucltop&blast_rank=1&RID=C5MB00FW016) | 699 | 98.97% |
|  |  |  |  | [*Kwoniella shandongensis*](https://blast.ncbi.nlm.nih.gov/Blast.cgi) | [MW710242.1](https://www.ncbi.nlm.nih.gov/nucleotide/MW710242.1?report=genbank&log$=nucltop&blast_rank=2&RID=C5MB00FW016) | 675 | 96.57% |
| 22 | Apr | 3CP | OM237250 | [*Naganishia albida*](https://blast.ncbi.nlm.nih.gov/Blast.cgi) | [MW709944.1](https://www.ncbi.nlm.nih.gov/nucleotide/MW709944.1?report=genbank&log$=nucltop&blast_rank=1&RID=C5MF5JY2016) | 1066 | 100.00% |
| 23 | Apr | 3CP | OM237251 | [*Cladosporium cladosporioides*](https://blast.ncbi.nlm.nih.gov/Blast.cgi) | [MT878604.1](https://www.ncbi.nlm.nih.gov/nucleotide/MT878604.1?report=genbank&log$=nucltop&blast_rank=1&RID=C5MH1G9U013) | 928 | 99.41% |
| 24 | Apr | 3CP | OM237252 | [*Cladosporium cladosporioides*](https://blast.ncbi.nlm.nih.gov/Blast.cgi) | [KF709213.1](https://www.ncbi.nlm.nih.gov/nucleotide/KF709213.1?report=genbank&log$=nucltop&blast_rank=1&RID=C5MK44XY016) | 933 | 99.80% |
| 25 | Apr | 3CP | OM237253 | [*Botrytis cinerea*](https://blast.ncbi.nlm.nih.gov/Blast.cgi) | [MT705878.1](https://www.ncbi.nlm.nih.gov/nucleotide/MT705878.1?report=genbank&log$=nucltop&blast_rank=1&RID=C5N5S6VJ016) | 771 | 95.14% |
| 26 | Apr | 3CP | OM237254 | [*Botrytis cinerea*](https://blast.ncbi.nlm.nih.gov/Blast.cgi) | [MT708074.1](https://www.ncbi.nlm.nih.gov/nucleotide/MT708074.1?report=genbank&log$=nucltop&blast_rank=1&RID=ECVWCV3W013) | 1128 | 98.65% |
| 27 | Apr | 3CO | OM237255 | [*Cladosporium tenuissimum*](https://blast.ncbi.nlm.nih.gov/Blast.cgi) | [JN624886.1](https://www.ncbi.nlm.nih.gov/nucleotide/JN624886.1?report=genbank&log$=nucltop&blast_rank=1&RID=C5N7TP59013) | 937 | 99.42% |
| 28 | Apr | 3CO | OM237256 | [*Cladosporium cladosporioides*](https://blast.ncbi.nlm.nih.gov/Blast.cgi) | [KY114882.1](https://www.ncbi.nlm.nih.gov/nucleotide/KY114882.1?report=genbank&log$=nucltop&blast_rank=1&RID=C5N9T0JP013) | 942 | 99.81% |
| 29 | Apr | PS | OM237257 | [*Didymella* sp.](https://blast.ncbi.nlm.nih.gov/Blast.cgi) | [MG198901.1](https://www.ncbi.nlm.nih.gov/nucleotide/MG198901.1?report=genbank&log$=nucltop&blast_rank=1&RID=C5NBN6F6013) | 1422 | 98.67% |
|  |  |  |  | [*Nothophoma spiraeae*](https://blast.ncbi.nlm.nih.gov/Blast.cgi) | [MN737832.1](https://www.ncbi.nlm.nih.gov/nucleotide/MN737832.1?report=genbank&log$=nucltop&blast_rank=2&RID=C5NBN6F6013) | 929 | 99.23% |
| 30 | Apr | PS | OM237258 | [*Aureobasidium pullulans*](https://blast.ncbi.nlm.nih.gov/Blast.cgi) | [MK772062.](https://www.ncbi.nlm.nih.gov/nucleotide/MK772062.1?report=genbank&log$=nucltop&blast_rank=1&RID=C5NGN738013) | 990 | 99.63% |
| 31 | Apr | PS | OM237259 | [*Didymella pinodella*](https://blast.ncbi.nlm.nih.gov/Blast.cgi) | [KX099646.1](https://www.ncbi.nlm.nih.gov/nucleotide/KX099646.1?report=genbank&log$=nucltop&blast_rank=1&RID=C6D8ZMKB013) | 937 | 99.04% |
| 32 | Apr | PS | OM237260 | [*Alternaria compacta*](https://blast.ncbi.nlm.nih.gov/Blast.cgi) | [MW008918.1](https://www.ncbi.nlm.nih.gov/nucleotide/MW008918.1?report=genbank&log$=nucltop&blast_rank=1&RID=C6DAW8R9016) | 989 | 100.00% |
| 33 | Apr | PS | OM237261 | [*Cladosporium herbarum*](https://blast.ncbi.nlm.nih.gov/Blast.cgi) | [KM977762.1](https://www.ncbi.nlm.nih.gov/nucleotide/KM977762.1?report=genbank&log$=nucltop&blast_rank=1&RID=C6M86TAZ013) | 941 | 99.42% |
| 34 | Apr | PS | OM237262 | [*Torula* sp.](https://blast.ncbi.nlm.nih.gov/Blast.cgi) | [KC427082.1](https://www.ncbi.nlm.nih.gov/nucleotide/KC427082.1?report=genbank&log$=nucltop&blast_rank=1&RID=C6MB9RBV013) | 948 | 98.69% |
|  |  |  |  | [*Latorua caligans*](https://blast.ncbi.nlm.nih.gov/Blast.cgi) | [MG655170.1](https://www.ncbi.nlm.nih.gov/nucleotide/MG655170.1?report=genbank&log$=nucltop&blast_rank=4&RID=C6MB9RBV013) | 924 | 98.84% |
| 35 | Apr | PS | OM237263 | [*Filobasidium* sp.](https://blast.ncbi.nlm.nih.gov/Blast.cgi) | [MN299307.1](https://www.ncbi.nlm.nih.gov/nucleotide/MN299307.1?report=genbank&log$=nucltop&blast_rank=1&RID=C6MGEAEU013) | 1002 | 98.93% |
|  |  |  |  | [*Cryptococcus chernovii*](https://blast.ncbi.nlm.nih.gov/Blast.cgi) | [FN400936.1](https://www.ncbi.nlm.nih.gov/nucleotide/FN400936.1?report=genbank&log$=nucltop&blast_rank=2&RID=C6MGEAEU013) | 1002 | 98.93% |
| 36 | Apr | FDP | OM237264 | [*Cladosporium cladosporioides*](https://blast.ncbi.nlm.nih.gov/Blast.cgi) | [MW453186.1](https://www.ncbi.nlm.nih.gov/nucleotide/MW453186.1?report=genbank&log$=nucltop&blast_rank=1&RID=C6MMKR8B016) | 937 | 99.42% |
| 37 | Apr | FDO | OM237265 | [*Phoma medicaginis*](https://blast.ncbi.nlm.nih.gov/Blast.cgi) | [LN827697.1](https://www.ncbi.nlm.nih.gov/nucleotide/LN827697.1?report=genbank&log$=nucltop&blast_rank=1&RID=C6MR674C013) | 1256 | 99.00% |
| 1 | May | 3CO | OM237266 | [Uncultured fungus](https://blast.ncbi.nlm.nih.gov/Blast.cgi) | [JX984780.1](https://www.ncbi.nlm.nih.gov/nucleotide/JX984780.1?report=genbank&log$=nucltop&blast_rank=1&RID=C6PZXTE8016) | 953 | 99.06% |
|  |  |  |  | *Periconia* sp. | MZ374640.1 | 939 | 99.80% |
| 2 | May | 3CO | OM237267 | [*Didymella glomerata*](https://blast.ncbi.nlm.nih.gov/Blast.cgi) | [MK180611.1](https://www.ncbi.nlm.nih.gov/nucleotide/MK180611.1?report=genbank&log$=nucltop&blast_rank=1&RID=C6R4CTFE016) | 922 | 99.80% |
| 3 | May | 3CO | OM237268 | [*Aureobasidium pullulans*](https://blast.ncbi.nlm.nih.gov/Blast.cgi) | [MW710410.1](https://www.ncbi.nlm.nih.gov/nucleotide/MW710410.1?report=genbank&log$=nucltop&blast_rank=1&RID=C6R6MWEY016) | 568 | 91.17% |
| 4 | May | 3CO | OM237269 | [*Didymella* sp.](https://blast.ncbi.nlm.nih.gov/Blast.cgi) | [MK595527.1](https://www.ncbi.nlm.nih.gov/nucleotide/MK595527.1?report=genbank&log$=nucltop&blast_rank=1&RID=C6R97XW2016) | 926 | 99.61% |
| 5 | May | 3CO | OM237270 | [*Cladosporium* sp.](https://blast.ncbi.nlm.nih.gov/Blast.cgi) | [KT826668.1](https://www.ncbi.nlm.nih.gov/nucleotide/KT826668.1?report=genbank&log$=nucltop&blast_rank=1&RID=C6REJ2YE016) | 944 | 99.61% |
| 6 | May | 5CO | OM237271 | [*Phoma* sp.](https://blast.ncbi.nlm.nih.gov/Blast.cgi) | [JX896660.1](https://www.ncbi.nlm.nih.gov/nucleotide/JX896660.1?report=genbank&log$=nucltop&blast_rank=1&RID=C6RN6KJN016) | 922 | 99.60% |
|  |  |  |  | [*Peyronellaea glomerata*](https://blast.ncbi.nlm.nih.gov/Blast.cgi) | [KM014745.1](https://www.ncbi.nlm.nih.gov/nucleotide/KM014745.1?report=genbank&log$=nucltop&blast_rank=2&RID=C6RN6KJN016) | 917 | 99.41% |
| 7 | May | 5CO | OM237272 | [Fungal sp.](https://blast.ncbi.nlm.nih.gov/Blast.cgi) | [MT112978.1](https://www.ncbi.nlm.nih.gov/nucleotide/MT112978.1?report=genbank&log$=nucltop&blast_rank=1&RID=C6RU1E4C01R) | 941 | 99.42% |
|  |  |  |  | [*Cladosporium herbarum*](https://blast.ncbi.nlm.nih.gov/Blast.cgi) | [KU182498.1](https://www.ncbi.nlm.nih.gov/nucleotide/KU182498.1?report=genbank&log$=nucltop&blast_rank=3&RID=C6RU1E4C01R) | 939 | 99.23% |
| 8 | May | 5CO | OM237273 | [*Alternaria alternata*](https://blast.ncbi.nlm.nih.gov/Blast.cgi) | [MW009036.1](https://www.ncbi.nlm.nih.gov/nucleotide/MW009036.1?report=genbank&log$=nucltop&blast_rank=1&RID=C6RZW6T501R) | 990 | 99.81% |
| 9 | May | 5CO | OM237274 | [*Alternaria alternata*](https://blast.ncbi.nlm.nih.gov/Blast.cgi) | [MW008875.1](https://www.ncbi.nlm.nih.gov/nucleotide/MW008875.1?report=genbank&log$=nucltop&blast_rank=1&RID=C6S31XYC016) | 1000 | 99.82% |
| 10 | May | 5CO | OM237275 | [*Candida parapsilosis*](https://blast.ncbi.nlm.nih.gov/Blast.cgi) | [KP674969.1](https://www.ncbi.nlm.nih.gov/nucleotide/KP674969.1?report=genbank&log$=nucltop&blast_rank=1&RID=C6S593NA01R) | 889 | 99.59% |
| 11 | May | 5CO | *OM237276* | [*Stagonospora* sp.](#alnHdr_1238828057) | MF788208.1 | 985 | 99.63% |
| 12 | May | FDP | OM237277 | [*Penicillium olsonii*](https://blast.ncbi.nlm.nih.gov/Blast.cgi) | [MH705351.1](https://www.ncbi.nlm.nih.gov/nucleotide/MH705351.1?report=genbank&log$=nucltop&blast_rank=1&RID=C6SAUK8501R) | 985 | 99.27% |
| 13 | May | FDP | OM237278 | [*Cladosporium asperulatum*](https://blast.ncbi.nlm.nih.gov/Blast.cgi) | [MT786367.1](https://www.ncbi.nlm.nih.gov/nucleotide/MT786367.1?report=genbank&log$=nucltop&blast_rank=1&RID=C6T0MSZW01R) | 946 | 99.81% |
| 14 | May | FDP | OM237279 | [*Didymella pinodella*](https://blast.ncbi.nlm.nih.gov/Blast.cgi) | [KX099646.1](https://www.ncbi.nlm.nih.gov/nucleotide/KX099646.1?report=genbank&log$=nucltop&blast_rank=1&RID=CS45B4DH016) | 937 | 99.61% |
| 15 | May | FDP | OM237280 | [*Cladosporium herbarum*](https://blast.ncbi.nlm.nih.gov/Blast.cgi) | [KX611004.1](https://www.ncbi.nlm.nih.gov/nucleotide/KX611004.1?report=genbank&log$=nucltop&blast_rank=1&RID=C6T2MT4F01R) | 948 | 100.00% |
| 16 | May | FDP | OM237281 | [*Phoma fungicola*](https://blast.ncbi.nlm.nih.gov/Blast.cgi) | [KF293763.1](https://www.ncbi.nlm.nih.gov/nucleotide/KF293763.1?report=genbank&log$=nucltop&blast_rank=1&RID=C6T59GX3013) | 929 | 99.80% |
| 17 | May | FDP | OM237282 | [Fungal sp.](https://blast.ncbi.nlm.nih.gov/Blast.cgi) | [KY404958.1](https://www.ncbi.nlm.nih.gov/nucleotide/KY404958.1?report=genbank&log$=nucltop&blast_rank=1&RID=C6T766U9016) | 1009 | 99.28% |
|  |  |  |  | *Camarosporium* sp. | MG065734.1 | 992 | 98.58% |
| 18 | May | 5CP | OM237283 | [*Dothiorella* sp.](https://blast.ncbi.nlm.nih.gov/Blast.cgi) | [KX219605.1](https://www.ncbi.nlm.nih.gov/nucleotide/KX219605.1?report=genbank&log$=nucltop&blast_rank=1&RID=C6TAPD9301R) | 926 | 100.00% |
| 19 | May | 5CP | OM237284 | [*Alternaria angustiovoidea*](https://blast.ncbi.nlm.nih.gov/Blast.cgi) | [MW008905.1](https://www.ncbi.nlm.nih.gov/nucleotide/MW008905.1?report=genbank&log$=nucltop&blast_rank=1&RID=C6TED5GE016) | 992 | 99.82% |
| 20 | May | 5CP | OM237285 | [*Cladosporium sphaerospermum*](https://blast.ncbi.nlm.nih.gov/Blast.cgi) | [KM278022.1](https://www.ncbi.nlm.nih.gov/nucleotide/KM278022.1?report=genbank&log$=nucltop&blast_rank=1&RID=C6TGAVZ4013) | 941 | 99.42% |
| 21 | May | 5CP | OM237286 | [*Scheleobrachea quadrata*](https://blast.ncbi.nlm.nih.gov/Blast.cgi) | [MH858886.1](https://www.ncbi.nlm.nih.gov/nucleotide/MH858886.1?report=genbank&log$=nucltop&blast_rank=1&RID=C6TJ8XAB01R) | 1031 | 100.00% |
| 22 | May | 3CP | OM237287 | [*Filobasidium magnum*](https://blast.ncbi.nlm.nih.gov/Blast.cgi) | [MH197140.1](https://www.ncbi.nlm.nih.gov/nucleotide/MH197140.1?report=genbank&log$=nucltop&blast_rank=1&RID=C6TMFSP001R) | 1077 | 99.33% |
| 23 | May | 3CP | OM237288 | [*Cryptococcus* sp.](https://blast.ncbi.nlm.nih.gov/Blast.cgi) | [HQ426594.1](https://www.ncbi.nlm.nih.gov/nucleotide/HQ426594.1?report=genbank&log$=nucltop&blast_rank=1&RID=C6TPFGXC01R) | 1077 | 99.33% |
|  |  |  |  | [*Cryptococcus magnus*](https://blast.ncbi.nlm.nih.gov/Blast.cgi) | [EU871517.1](https://www.ncbi.nlm.nih.gov/nucleotide/EU871517.1?report=genbank&log$=nucltop&blast_rank=2&RID=C6TPFGXC01R) | 1077 | 99.33% |
| 24 | May | LIB | OM237289 | [*Curvularia kusanoi*](https://blast.ncbi.nlm.nih.gov/Blast.cgi) | [NR_165187.1](https://www.ncbi.nlm.nih.gov/nucleotide/NR_165187.1?report=genbank&log$=nucltop&blast_rank=1&RID=C6TT2XZX01R) | 994 | 98.25% |
| 25 | May | LIB | OM237290 | [*Cladosporium macrocarpum*](https://blast.ncbi.nlm.nih.gov/Blast.cgi) | [KX815295.1](https://www.ncbi.nlm.nih.gov/nucleotide/KX815295.1?report=genbank&log$=nucltop&blast_rank=1&RID=C6TX3WPD01R) | 948 | 100.00% |
| 26 | May | PS | OM237291 | [Fungal sp.](https://blast.ncbi.nlm.nih.gov/Blast.cgi) | [MW603421.1](https://www.ncbi.nlm.nih.gov/nucleotide/MW603421.1?report=genbank&log$=nucltop&blast_rank=1&RID=C6TYX8JY016) | 935 | 99.80% |
|  |  |  |  | [*Cladosporium cladosporioides*](https://blast.ncbi.nlm.nih.gov/Blast.cgi) | [MW113271.1](https://www.ncbi.nlm.nih.gov/nucleotide/MW113271.1?report=genbank&log$=nucltop&blast_rank=4&RID=C6TYX8JY016) | 931 | 99.80% |
| 27 | May | PS | OM237292 | [*Naganishia* sp.](https://blast.ncbi.nlm.nih.gov/Blast.cgi) | [MK942582.1](https://www.ncbi.nlm.nih.gov/nucleotide/MK942582.1?report=genbank&log$=nucltop&blast_rank=1&RID=C6U2HY6101R) | 1072 | 99.49% |
| 28 | May | PS | OM237293 | [Fungal sp.](https://blast.ncbi.nlm.nih.gov/Blast.cgi) | [MK756042.1](https://www.ncbi.nlm.nih.gov/nucleotide/MK756042.1?report=genbank&log$=nucltop&blast_rank=1&RID=C6U4P77R01R) | 1020 | 99.64% |
|  |  |  |  | [*Purpureocillium lilacinum*](https://blast.ncbi.nlm.nih.gov/Blast.cgi) | [MH483905.1](https://www.ncbi.nlm.nih.gov/nucleotide/MH483905.1?report=genbank&log$=nucltop&blast_rank=2&RID=C6U4P77R01R) | 1020 | 99.64% |
| 29 | May | PS | OM237294 | [*Alternaria alternata*](https://blast.ncbi.nlm.nih.gov/Blast.cgi) | [MW009000.1](https://www.ncbi.nlm.nih.gov/nucleotide/MW009000.1?report=genbank&log$=nucltop&blast_rank=1&RID=C6UASV2401R) | 990 | 99.63% |
| 30 | May | PS | OM237295 | [*Alternaria angustiovoidea*](https://blast.ncbi.nlm.nih.gov/Blast.cgi) | [MW008876.1](https://www.ncbi.nlm.nih.gov/nucleotide/MW008876.1?report=genbank&log$=nucltop&blast_rank=1&RID=C6UCVS8F01R) | 987 | 99.81% |
| 31 | May | PS | OM237296 | [*Alternaria* sp.](https://blast.ncbi.nlm.nih.gov/Blast.cgi) | [KT269408.1](https://www.ncbi.nlm.nih.gov/nucleotide/KT269408.1?report=genbank&log$=nucltop&blast_rank=1&RID=C6UF1DN5016) | 1018 | 99.12% |
| 32 | May | PS | OM237297 | [*Alternaria* sp.](https://blast.ncbi.nlm.nih.gov/Blast.cgi) | [MW009011.1](https://www.ncbi.nlm.nih.gov/nucleotide/MW009011.1?report=genbank&log$=nucltop&blast_rank=1&RID=C6UJ6W2G01R) | 992 | 99.81% |
|  |  |  |  | [*Alternaria alternata*](https://blast.ncbi.nlm.nih.gov/Blast.cgi) | [MW008875.1](https://www.ncbi.nlm.nih.gov/nucleotide/MW008875.1?report=genbank&log$=nucltop&blast_rank=2&RID=C6UJ6W2G01R) | 989 | 99.27% |
| 33 | May | CDP | OM237298 | [*Cladosporium* sp.](https://blast.ncbi.nlm.nih.gov/Blast.cgi) | [KT826668.1](https://www.ncbi.nlm.nih.gov/nucleotide/KT826668.1?report=genbank&log$=nucltop&blast_rank=1&RID=C6UNF1S901R) | 944 | 99.81% |
|  |  |  |  | [*Cladosporium sinuosum*](https://blast.ncbi.nlm.nih.gov/Blast.cgi) | [MN826919.1](https://www.ncbi.nlm.nih.gov/nucleotide/MN826919.1?report=genbank&log$=nucltop&blast_rank=3&RID=C6UNF1S901R) | 942 | 99.81% |
| 34 | May | CDP | OM237299 | [*Alternaria* sp.](https://blast.ncbi.nlm.nih.gov/Blast.cgi) | [MK649968.1](https://www.ncbi.nlm.nih.gov/nucleotide/MK649968.1?report=genbank&log$=nucltop&blast_rank=1&RID=C6USYWHB01R) | 977 | 99.44% |
|  |  |  |  | [*Actinomucor elegans*](https://blast.ncbi.nlm.nih.gov/Blast.cgi) | [MT449941.1](https://www.ncbi.nlm.nih.gov/nucleotide/MT449941.1?report=genbank&log$=nucltop&blast_rank=3&RID=C6USYWHB01R) | 977 | 99.44% |
| 35 | May | CDP | OM237300 | [*Alternaria* sp.](https://blast.ncbi.nlm.nih.gov/Blast.cgi) | [MW009028.1](https://www.ncbi.nlm.nih.gov/nucleotide/MW009028.1?report=genbank&log$=nucltop&blast_rank=1&RID=C6UXY08901R) | 990 | 99.81% |
|  |  |  |  | [*Alternaria tamaricis*](https://blast.ncbi.nlm.nih.gov/Blast.cgi) | [MW009027.1](https://www.ncbi.nlm.nih.gov/nucleotide/MW009027.1?report=genbank&log$=nucltop&blast_rank=2&RID=C6UXY08901R) | 987 | 99.45% |
| 36 | May | FDO | OM237301 | [*Dothiorella gregaria*](https://blast.ncbi.nlm.nih.gov/Blast.cgi) | [MH791151.1](https://www.ncbi.nlm.nih.gov/nucleotide/MH791151.1?report=genbank&log$=nucltop&blast_rank=1&RID=C6V0UZ2G01R) | 933 | 99.61% |
| 37 | May | FDO | OM237302 | [*Phoma* sp.](https://blast.ncbi.nlm.nih.gov/Blast.cgi) | [KM454881.1](https://www.ncbi.nlm.nih.gov/nucleotide/KM454881.1?report=genbank&log$=nucltop&blast_rank=1&RID=C6V33W2X01R) | 929 | 99.61% |
| 38 | May | FDO | OM237303 | [*Naganishia uzbekistanensis*](https://blast.ncbi.nlm.nih.gov/Blast.cgi) | [MG020691.1](https://www.ncbi.nlm.nih.gov/nucleotide/MG020691.1?report=genbank&log$=nucltop&blast_rank=1&RID=C6V8HBS501R) | 1081 | 99.83% |
| 39 | May | FDO | OM237304 | [Uncultured *Phoma*](https://blast.ncbi.nlm.nih.gov/Blast.cgi) | [MG462850.1](https://www.ncbi.nlm.nih.gov/nucleotide/MG462850.1?report=genbank&log$=nucltop&blast_rank=1&RID=C6VCBJKZ016) | 928 | 99.61% |
|  |  |  |  | [*Leptosphaerulina australis*](https://blast.ncbi.nlm.nih.gov/Blast.cgi) | [KF293970.1](https://www.ncbi.nlm.nih.gov/nucleotide/KF293970.1?report=genbank&log$=nucltop&blast_rank=3&RID=C6VCBJKZ016) | 926 | 99.22% |
| 40 | May | FDO | OM237305 | [*Coniothyrium aleuritis*](https://blast.ncbi.nlm.nih.gov/Blast.cgi) | [MK070113.1](https://www.ncbi.nlm.nih.gov/nucleotide/MK070113.1?report=genbank&log$=nucltop&blast_rank=1&RID=C6VGJXK0016) | 902 | 100.00% |
| 41 | May | FDO | OM237306 | [Uncultured fungus](https://blast.ncbi.nlm.nih.gov/Blast.cgi) | [JX984780.1](https://www.ncbi.nlm.nih.gov/nucleotide/JX984780.1?report=genbank&log$=nucltop&blast_rank=1&RID=C6VJMCX501R) | 953 | 99.43% |
|  |  |  |  | [*Periconia macrospinosa*](https://blast.ncbi.nlm.nih.gov/Blast.cgi) | [MG576113.1](https://www.ncbi.nlm.nih.gov/nucleotide/MG576113.1?report=genbank&log$=nucltop&blast_rank=4&RID=C6VJMCX501R) | 917 | 98.46% |
| 42 | May | FDO | OM237307 | [*Naganishia albida*](https://blast.ncbi.nlm.nih.gov/Blast.cgi) | [KY238152.1](https://www.ncbi.nlm.nih.gov/nucleotide/KY238152.1?report=genbank&log$=nucltop&blast_rank=1&RID=C6VST7WU016) | 1075 | 99.66% |
| 43 | May | CDP | OM237308 | [*Coprinopsis kubickae*](https://blast.ncbi.nlm.nih.gov/Blast.cgi) | [MH422562.1](https://www.ncbi.nlm.nih.gov/nucleotide/MH422562.1?report=genbank&log$=nucltop&blast_rank=1&RID=C6VUU9NW013) | 1144 | 99.06% |
| 44 | May | CDO | OM237309 | [Uncultured fungus](https://blast.ncbi.nlm.nih.gov/Blast.cgi) | [FJ820753.1](https://www.ncbi.nlm.nih.gov/nucleotide/FJ820753.1?report=genbank&log$=nucltop&blast_rank=1&RID=EG53UTTF013) | 942 | 99.61% |
|  |  |  |  | [*Cladosporium* sp.](https://blast.ncbi.nlm.nih.gov/Blast.cgi) | [MW713481.1](https://www.ncbi.nlm.nih.gov/nucleotide/MW713481.1?report=genbank&log$=nucltop&blast_rank=4&RID=EG53UTTF013) | 928 | 99.80% |
| 45 | May | CDO | OM237310 | [*Cladosporium* sp.](https://blast.ncbi.nlm.nih.gov/Blast.cgi) | [KX034366.1](https://www.ncbi.nlm.nih.gov/nucleotide/KX034366.1?report=genbank&log$=nucltop&blast_rank=1&RID=C6W04K1U01R) | 941 | 99.42% |
| 46 | May | CDO | OM237311 | [*Cladosporium cladosporioides*](https://blast.ncbi.nlm.nih.gov/Blast.cgi) | [MK761055.1](https://www.ncbi.nlm.nih.gov/nucleotide/MK761055.1?report=genbank&log$=nucltop&blast_rank=2&RID=C6W04K1U01R) | 939 | 99.42% |

BLAST search closest matches of fungal internal transcribed spacer DNA sequences amplified from Tianjin University airborne isolated strains. Strain GenBank accession codes, accession codes for the closest GenBank matches, sequence identity, and overlap of each match are reported. 3CP = Canteen 3 Peak, 3CO = Canteen 3 Off-peak, 5CP = Canteen 5 Peak, 5CO = Canteen 5 Off-peak, CDP = Chinese students Dorm Peak, CDO = Chinese students Dorm Off-peak, FDP = Foreign students Dorm Peak, FDO = Foreign students Dorm Off-peak, LIB = Library, PS = Peiyang Square.

**Table S5.** Airborne fungal genera and number of strains isolated in each month.

| **Fungal Genera** | **Jun** | **Jul** | **Aug** | **Sep** | **Oct** | **Nov** | **Dec** | **Jan** | **Feb** | **Mar** | **Apr** | **May** | **Total** | **Percentage (%)** |
| --- | --- | --- | --- | --- | --- | --- | --- | --- | --- | --- | --- | --- | --- | --- |
| *Cladosporium* | 30 | 18 | 6 | 63 | 7 | 2 | 11 | 17 | 3 | 9 | 12 | 11 | 189 | 29.49 |
| *Alternaria* | 17 | 32 | 4 | 60 | 5 | 0 | 7 | 8 | 12 | 5 | 8 | 8 | 166 | 25.90 |
| *Epicoccum* | 1 | 0 | 2 | 32 | 3 | 0 | 0 | 0 | 1 | 1 | 0 | 0 | 40 | 6.24 |
| *Aspergillus* | 6 | 5 | 1 | 4 | 4 | 5 | 2 | 1 | 1 | 0 | 1 | 0 | 30 | 4.68 |
| *Talaromyces* | 0 | 0 | 0 | 1 | 2 | 2 | 0 | 4 | 0 | 11 | 0 | 0 | 20 | 3.12 |
| *Penicillium* | 0 | 3 | 1 | 2 | 0 | 3 | 0 | 3 | 1 | 2 | 1 | 1 | 17 | 2.65 |
| *Aureobasidium* | 0 | 3 | 0 | 0 | 0 | 0 | 3 | 2 | 0 | 1 | 2 | 1 | 12 | 1.87 |
| *Didymella* | 0 | 1 | 2 | 0 | 1 | 0 | 1 | 2 | 0 | 1 | 1 | 3 | 12 | 1.87 |
| *Naganishia* | 0 | 0 | 0 | 0 | 1 | 2 | 3 | 0 | 0 | 0 | 2 | 3 | 11 | 1.72 |
| *Fusarium* | 0 | 0 | 0 | 8 | 0 | 0 | 1 | 0 | 0 | 1 | 0 | 0 | 10 | 1.56 |
| *Neurospora* | 0 | 1 | 5 | 2 | 0 | 0 | 0 | 0 | 0 | 0 | 0 | 0 | 8 | 1.25 |
| *Arthrinium* | 0 | 0 | 0 | 0 | 2 | 0 | 0 | 4 | 0 | 0 | 0 | 0 | 6 | 0.94 |
| *Chaetomium* | 1 | 0 | 0 | 0 | 0 | 0 | 1 | 2 | 1 | 0 | 1 | 0 | 6 | 0.94 |
| *Periconia* | 0 | 1 | 0 | 1 | 0 | 1 | 0 | 1 | 0 | 0 | 0 | 2 | 6 | 0.94 |
| *Phoma* | 0 | 1 | 0 | 1 | 0 | 0 | 0 | 0 | 1 | 0 | 1 | 2 | 6 | 0.94 |
| *Cryptococcus* | 0 | 0 | 0 | 0 | 0 | 0 | 1 | 2 | 0 | 0 | 1 | 1 | 5 | 0.78 |
| *Filobasidium* | 0 | 0 | 3 | 0 | 1 | 0 | 0 | 0 | 0 | 0 | 0 | 1 | 5 | 0.78 |
| *Torula* | 0 | 0 | 0 | 0 | 0 | 1 | 0 | 2 | 2 | 0 | 0 | 0 | 5 | 0.78 |
| *Botrytis* | 0 | 1 | 0 | 0 | 0 | 0 | 0 | 0 | 0 | 1 | 2 | 0 | 4 | 0.62 |
| *Curvularia* | 0 | 0 | 1 | 2 | 0 | 0 | 0 | 0 | 0 | 0 | 0 | 1 | 4 | 0.62 |
| *Nothophoma* | 1 | 0 | 0 | 1 | 0 | 0 | 0 | 0 | 1 | 0 | 1 | 0 | 4 | 0.62 |
| *Sarocladium* | 3 | 0 | 0 | 1 | 0 | 0 | 0 | 0 | 0 | 0 | 0 | 0 | 4 | 0.62 |
| *Leptosphaeria* | 2 | 0 | 0 | 0 | 0 | 0 | 0 | 1 | 0 | 0 | 0 | 0 | 3 | 0.47 |
| *Cercospora* | 0 | 0 | 0 | 2 | 1 | 0 | 0 | 0 | 0 | 0 | 0 | 0 | 3 | 0.47 |
| *Peyronellaea* | 0 | 1 | 0 | 0 | 0 | 0 | 0 | 0 | 0 | 1 | 0 | 1 | 3 | 0.47 |
| *Phanerochaete* | 0 | 0 | 3 | 0 | 0 | 0 | 0 | 0 | 0 | 0 | 0 | 0 | 3 | 0.47 |
| *Stemphylium* | 0 | 0 | 0 | 1 | 0 | 2 | 0 | 0 | 0 | 0 | 0 | 0 | 3 | 0.47 |
| *Coprinopsis* | 0 | 0 | 1 | 0 | 0 | 0 | 0 | 0 | 0 | 0 | 0 | 1 | 2 | 0.31 |
| *Dothiorella* | 0 | 0 | 0 | 0 | 0 | 0 | 0 | 0 | 0 | 0 | 0 | 2 | 2 | 0.31 |
| *Gloeophyllum* | 0 | 0 | 2 | 0 | 0 | 0 | 0 | 0 | 0 | 0 | 0 | 0 | 2 | 0.31 |
| *Microsphaeropsis* | 0 | 0 | 0 | 0 | 0 | 1 | 1 | 0 | 0 | 0 | 0 | 0 | 2 | 0.31 |
| *Phaeosphaeria* | 0 | 1 | 0 | 0 | 0 | 0 | 0 | 1 | 0 | 0 | 0 | 0 | 2 | 0.31 |
| *Purpureocillium* | 0 | 0 | 0 | 0 |  | 1 | 0 | 0 | 0 | 0 | 0 | 1 | 2 | 0.31 |
| *Vishniacozyma* | 0 | 0 | 0 | 1 | 0 | 0 | 1 | 0 | 0 | 0 | 0 | 0 | 2 | 0.31 |
| *Wallemia* | 0 | 0 | 2 | 0 | 0 | 0 | 0 | 0 | 0 | 0 | 0 | 0 | 2 | 0.31 |
| *Zygosporium* | 0 | 0 | 2 | 0 | 0 | 0 | 0 | 0 | 0 | 0 | 0 | 0 | 2 | 0.31 |
| *Actinomucor* | 0 | 0 | 0 | 0 | 0 | 0 | 0 | 0 | 0 | 0 | 0 | 1 | 1 | 0.16 |
| *Allophoma* | 0 | 1 | 0 | 0 | 0 | 0 | 0 | 0 | 0 | 0 | 0 | 0 | 1 | 0.16 |
| *Ascochyta* | 0 | 0 | 0 | 0 | 0 | 0 | 0 | 0 | 0 | 1 | 0 | 0 | 1 | 0.16 |
| *Camarosporium* | 0 | 0 | 0 | 0 | 0 | 0 | 0 | 0 | 0 | 0 | 0 | 1 | 1 | 0.16 |
| *Candida* | 0 | 0 | 0 | 0 | 0 | 0 | 0 | 0 | 0 | 0 | 0 | 1 | 1 | 0.16 |
| *Cephalotrichum* | 0 | 0 | 0 | 0 | 0 | 0 | 0 | 1 | 0 | 0 | 0 | 0 | 1 | 0.16 |
| *Cochliobolus* | 0 | 0 | 0 | 1 | 0 | 0 | 0 | 0 | 0 | 0 | 0 | 0 | 1 | 0.16 |
| *Coelastrella* | 0 | 0 | 1 | 0 | 0 | 0 | 0 | 0 | 0 | 0 | 0 | 0 | 1 | 0.16 |
| *Coniothyrium* | 0 | 0 | 0 | 0 | 0 | 0 | 0 | 0 | 0 | 0 | 0 | 1 | 1 | 0.16 |
| *Coriolopsis* | 0 | 0 | 1 | 0 | 0 | 0 | 0 | 0 | 0 | 0 | 0 | 0 | 1 | 0.16 |
| *Cymostachys* | 0 | 0 | 0 | 0 | 1 | 0 | 0 | 0 | 0 | 0 | 0 | 0 | 1 | 0.16 |
| *Dicyma* | 0 | 0 | 0 | 0 | 0 | 0 | 0 | 1 | 0 | 0 | 0 | 0 | 1 | 0.16 |
| [*Periconia*](https://blast.ncbi.nlm.nih.gov/Blast.cgi) | 0 | 0 | 0 | 0 | 0 | 0 | 0 | 0 | 0 | 1 | 0 | 0 | 1 | 0.16 |
| *Dothidea* | 0 | 0 | 0 | 1 | 0 | 0 | 0 | 0 | 0 | 0 | 0 | 0 | 1 | 0.16 |
| *Exserohilum* | 0 | 0 | 0 | 1 | 0 | 0 | 0 | 0 | 0 | 0 | 0 | 0 | 1 | 0.16 |
| *Fomitopsis* | 0 | 0 | 1 | 0 | 0 | 0 | 0 | 0 | 0 | 0 | 0 | 0 | 1 | 0.16 |
| *Geomyces* | 0 | 0 | 0 | 0 | 0 | 0 | 1 | 0 | 0 | 0 | 0 | 0 | 1 | 0.16 |
| *Granulobasidium* | 0 | 0 | 0 | 0 | 0 | 1 | 0 | 0 | 0 | 0 | 0 | 0 | 1 | 0.16 |
| *Hanseniaspora* | 0 | 0 | 1 | 0 | 0 | 0 | 0 | 0 | 0 | 0 | 0 | 0 | 1 | 0.16 |
| *Hypoxylon* | 0 | 0 | 0 | 0 | 0 | 0 | 0 | 0 | 0 | 1 | 0 | 0 | 1 | 0.16 |
| *Kwoniella* | 0 | 0 | 0 | 0 | 0 | 0 | 0 | 0 | 0 | 0 | 1 | 0 | 1 | 0.16 |
| *Lachancea* | 0 | 0 | 0 | 0 | 0 | 0 | 0 | 1 | 0 | 0 | 0 | 0 | 1 | 0.16 |
| *Latorua* | 0 | 0 | 0 | 0 | 0 | 0 | 0 | 0 | 0 | 0 | 1 | 0 | 1 | 0.16 |
| *Leptosphaerulina* | 0 | 0 | 0 | 0 | 0 | 0 | 0 | 0 | 0 | 0 | 0 | 1 | 1 | 0.16 |
| *Mucor* | 1 | 0 | 0 | 0 | 0 | 0 | 0 | 0 | 0 | 0 | 0 | 0 | 1 | 0.16 |
| *Ochrocladosporium* | 0 | 0 | 0 | 1 | 0 | 0 | 0 | 0 | 0 | 0 | 0 | 0 | 1 | 0.16 |
| *Papiliotrema* | 0 | 0 | 0 | 0 | 0 | 0 | 0 | 0 | 1 | 0 | 0 | 0 | 1 | 0.16 |
| *Paraphoma* | 1 | 0 | 0 | 0 | 0 | 0 | 0 | 0 | 0 | 0 | 0 | 0 | 1 | 0.16 |
| [*Stagonospora*](https://blast.ncbi.nlm.nih.gov/Blast.cgi) | 0 | 0 | 0 | 0 | 0 | 0 | 0 | 0 | 0 | 0 | 0 | 1 | 1 | 0.16 |
| *Pithomyces* | 0 | 0 | 0 | 0 | 0 | 0 | 0 | 0 | 0 | 0 | 1 | 0 | 1 | 0.16 |
| *Rhodotorula* | 0 | 0 | 0 | 0 | 0 | 0 | 0 | 0 | 0 | 1 | 0 | 0 | 1 | 0.16 |
| *Scheleobrachea* | 0 | 0 | 0 | 0 | 0 | 0 | 0 | 0 | 0 | 0 | 0 | 1 | 1 | 0.16 |
| *Schizophyllum* | 0 | 0 | 0 | 0 | 0 | 0 | 0 | 0 | 0 | 0 | 1 | 0 | 1 | 0.16 |
| *Stagonospora* | 0 | 1 | 0 | 0 | 0 | 0 | 0 | 0 | 0 | 0 | 0 | 0 | 1 | 0.16 |
| *Taphrina* | 0 | 1 | 0 | 0 | 0 | 0 | 0 | 0 | 0 | 0 | 0 | 0 | 1 | 0.16 |
| *Teichospora* | 0 | 0 | 0 | 0 | 0 | 0 | 0 | 1 | 0 | 0 | 0 | 0 | 1 | 0.16 |
| *Valsa* | 0 | 1 | 0 | 0 | 0 | 0 | 0 | 0 | 0 | 0 | 0 | 0 | 1 | 0.16 |
| *Valsaria* | 0 | 0 | 0 | 0 | 0 | 0 | 1 | 0 | 0 | 0 | 0 | 0 | 1 | 0.16 |

**Table S6.** Airborne fungal species and number of strains isolated in each month.

| **Fungal Species** | **Jun** | **Jul** | **Aug** | **Sep** | **Oct** | **Nov** | **Dec** | **Jan** | **Feb** | **Mar** | **Apr** | **May** | **Total** | **Percentage (%)** |
| --- | --- | --- | --- | --- | --- | --- | --- | --- | --- | --- | --- | --- | --- | --- |
| *Alternaria alternata* | 12 | 13 | 4 | 35 | 3 | 0 | 6 | 5 | 10 | 2 | 5 | 4 | 99 | 15.44 |
| *Cladosporium cladosporioides* | 14 | 3 | 1 | 28 | 1 | 0 | 2 | 9 | 3 | 7 | 6 | 2 | 76 | 11.86 |
| *Epicoccum nigrum* | 0 | 0 | 2 | 30 | 3 | 0 | 0 | 0 | 1 | 1 | 0 | 0 | 37 | 5.77 |
| *Cladosporium tenuissimum* | 6 | 6 | 2 | 5 | 1 | 0 | 1 | 4 | 0 | 1 | 1 | 0 | 27 | 4.21 |
| [*Cladosporium anthropophilum*](https://blast.ncbi.nlm.nih.gov/Blast.cgi) | 0 | 3 | 1 | 11 | 2 | 0 | 4 | 0 | 0 | 0 | 0 | 0 | 21 | 3.28 |
| *Alternaria tenuissima* | 4 | 1 | 0 | 11 | 1 | 0 | 0 | 1 | 0 | 3 | 0 | 0 | 21 | 3.28 |
| *Alternaria compacta* | 1 | 4 | 0 | 4 | 0 | 0 | 0 | 2 | 1 | 0 | 2 | 0 | 14 | 2.18 |
| [*Cladosporium oxysporum*](https://blast.ncbi.nlm.nih.gov/Blast.cgi) | 4 | 2 | 1 | 7 | 0 | 0 | 0 | 0 | 0 | 0 | 0 | 0 | 14 | 2.18 |
| *Cladosporium* sp. | 3 | 0 | 0 | 3 | 0 | 0 | 0 | 0 | 0 | 0 | 0 | 3 | 9 | 1.40 |
| [*Alternaria angustiovoidea*](https://blast.ncbi.nlm.nih.gov/Blast.cgi) | 0 | 4 | 0 | 2 | 0 | 0 | 0 | 0 | 0 | 0 | 0 | 2 | 8 | 1.25 |
| *Alternaria tamaricis* | 0 | 6 | 0 | 1 | 0 | 0 | 0 | 0 | 0 | 0 | 0 | 1 | 8 | 1.25 |
| [*Talaromyces funiculosus*](https://blast.ncbi.nlm.nih.gov/Blast.cgi) | 0 | 0 | 0 | 1 | 1 | 0 | 0 | 1 | 0 | 5 | 0 | 0 | 8 | 1.25 |
| [*Aspergillus flavus*](https://blast.ncbi.nlm.nih.gov/Blast.cgi) | 0 | 1 | 0 | 2 | 0 | 2 | 1 | 0 | 0 | 0 | 1 | 0 | 7 | 1.09 |
| [*Aureobasidium pullulans*](https://blast.ncbi.nlm.nih.gov/Blast.cgi) | 0 | 0 | 0 | 0 | 0 | 0 | 1 | 2 | 0 | 1 | 2 | 1 | 7 | 1.09 |
| *Chaetomium globosum* | 1 | 0 | 0 | 0 | 0 | 0 | 1 | 2 | 1 | 1 | 1 | 0 | 7 | 1.09 |
| [*Cladosporium asperulatum*](https://blast.ncbi.nlm.nih.gov/Blast.cgi) | 0 | 1 | 0 | 1 | 0 | 0 | 3 | 1 | 0 | 0 | 0 | 1 | 7 | 1.09 |
| *Neurospora tetrasperma* | 0 | 0 | 5 | 2 | 0 | 0 | 0 | 0 | 0 | 0 | 0 | 0 | 7 | 1.09 |
| *Alternaria solani* | 0 | 3 | 0 | 2 | 0 | 0 | 0 | 0 | 0 | 0 | 1 | 0 | 6 | 0.94 |
| *Alternaria* sp. | 0 | 1 | 0 | 4 | 0 | 0 | 0 | 0 | 0 | 0 | 0 | 1 | 6 | 0.94 |
| [*Aspergillus niger*](https://blast.ncbi.nlm.nih.gov/Blast.cgi) | 0 | 3 | 1 | 1 | 0 | 0 | 0 | 1 | 0 | 0 | 0 | 0 | 6 | 0.94 |
| [*Cladosporium ramotenellum*](https://blast.ncbi.nlm.nih.gov/Blast.cgi) | 0 | 1 | 0 | 5 | 0 | 0 | 0 | 0 | 0 | 0 | 0 | 0 | 6 | 0.94 |
| *Fusarium incarnatum* | 0 | 0 | 0 | 6 | 0 | 0 | 0 | 0 | 0 | 0 | 0 | 0 | 6 | 0.94 |
| [*Naganishia albida*](https://blast.ncbi.nlm.nih.gov/Blast.cgi) | 0 | 0 | 0 | 0 | 1 | 0 | 2 | 0 | 0 | 0 | 2 | 1 | 6 | 0.94 |
| *Talaromyces verruculosus* | 0 | 0 | 0 | 0 | 0 | 0 | 0 | 2 | 0 | 4 | 0 | 0 | 6 | 0.94 |
| *Cladosporium halotolerans* | 0 | 0 | 1 | 1 | 0 | 1 | 0 | 2 | 0 | 0 | 0 | 0 | 5 | 0.78 |
| [*Cladosporium herbarum*](https://blast.ncbi.nlm.nih.gov/Blast.cgi) | 0 | 1 | 0 | 0 | 0 | 0 | 0 | 0 | 0 | 0 | 2 | 2 | 5 | 0.78 |
| *Filobasidium magnum* | 0 | 0 | 3 | 0 | 1 | 0 | 0 | 0 | 0 | 0 | 0 | 1 | 5 | 0.78 |
| *Botrytis cinerea* | 0 | 1 | 0 | 0 | 0 | 0 | 0 | 0 | 0 | 1 | 2 | 0 | 4 | 0.62 |
| [*Didymella macrostoma*](https://blast.ncbi.nlm.nih.gov/Blast.cgi) | 0 | 1 | 2 | 0 | 0 | 0 | 0 | 0 | 0 | 1 | 0 | 0 | 4 | 0.62 |
| [*Didymella* sp.](https://blast.ncbi.nlm.nih.gov/Blast.cgi) | 0 | 0 | 0 | 0 | 0 | 0 | 1 | 2 | 0 | 0 | 0 | 1 | 4 | 0.62 |
| *Penicillium concavorugulosum* | 0 | 0 | 0 | 0 | 0 | 2 | 0 | 2 | 0 | 0 | 0 | 0 | 4 | 0.62 |
| [*Penicillium oxalicum*](https://blast.ncbi.nlm.nih.gov/Blast.cgi) | 0 | 1 | 0 | 2 | 0 | 1 | 0 | 0 | 0 | 0 | 0 | 0 | 4 | 0.62 |
| [*Sarocladium strictum*](https://blast.ncbi.nlm.nih.gov/Blast.cgi) | 3 | 0 | 0 | 1 | 0 | 0 | 0 | 0 | 0 | 0 | 0 | 0 | 4 | 0.62 |
| [*Aureobasidium iranianum*](https://blast.ncbi.nlm.nih.gov/Blast.cgi) | 0 | 3 | 0 | 0 | 0 | 0 | 0 | 0 | 0 | 0 | 0 | 0 | 3 | 0.47 |
| *Cladosporium cucumerinum* | 1 | 0 | 0 | 0 | 0 | 0 | 1 | 0 | 0 | 0 | 1 | 0 | 3 | 0.47 |
| *Cladosporium oryzae* | 0 | 0 | 0 | 0 | 1 | 1 |  | 1 | 0 | 0 | 0 | 0 | 3 | 0.47 |
| [*Cladosporium uredinicola*](https://blast.ncbi.nlm.nih.gov/Blast.cgi) | 2 | 0 | 0 | 0 | 1 | 0 | 0 | 0 | 0 | 0 | 0 | 0 | 3 | 0.47 |
| *Epicoccum* sp. | 1 | 0 | 0 | 2 | 0 | 0 | 0 | 0 | 0 | 0 | 0 | 0 | 3 | 0.47 |
| [*Naganishia uzbekistanensis*](https://blast.ncbi.nlm.nih.gov/Blast.cgi) | 0 | 0 | 0 | 0 | 0 | 2 | 0 | 0 | 0 | 0 | 0 | 1 | 3 | 0.47 |
| *Torula caligans* | 0 | 0 | 0 | 0 | 0 | 1 | 0 | 1 | 1 | 0 | 0 | 0 | 3 | 0.47 |
| *Alternaria porri* | 0 | 0 | 0 | 1 | 0 | 0 | 1 | 0 | 0 | 0 | 0 | 0 | 2 | 0.31 |
| [*Arthrinium arundinis*](https://blast.ncbi.nlm.nih.gov/Blast.cgi) | 0 | 0 | 0 |  | 2 | 0 | 0 | 0 | 0 | 0 | 0 | 0 | 2 | 0.31 |
| *Arthrinium marii* | 0 | 0 | 0 | 0 | 0 | 0 | 0 | 2 | 0 | 0 | 0 | 0 | 2 | 0.31 |
| *Arthrinium saccharicola* | 0 | 0 | 0 | 0 | 0 | 0 | 0 | 2 | 0 | 0 | 0 | 0 | 2 | 0.31 |
| [*Aspergillus ochraceus*](https://blast.ncbi.nlm.nih.gov/Blast.cgi) | 0 | 1 | 0 | 0 | 1 | 0 | 0 | 0 | 0 | 0 | 0 | 0 | 2 | 0.31 |
| *Aspergillus oryzae* | 2 | 0 | 0 | 0 | 0 | 0 | 0 | 0 | 0 | 0 | 0 | 0 | 2 | 0.31 |
| [*Aspergillus sydowii*](https://blast.ncbi.nlm.nih.gov/Blast.cgi) | 0 | 0 | 0 | 0 | 1 | 1 | 0 | 0 | 0 | 0 | 0 | 0 | 2 | 0.31 |
| [*Aspergillus tubingensis*](https://blast.ncbi.nlm.nih.gov/Blast.cgi) | 0 | 0 | 0 | 0 | 0 | 2 | 0 | 0 | 0 | 0 | 0 | 0 | 2 | 0.31 |
| *Aspergillus versicolor* | 2 | 0 | 0 | 0 | 0 | 0 | 0 | 0 | 0 | 0 | 0 | 0 | 2 | 0.31 |
| [*Aureobasidium* sp.](https://blast.ncbi.nlm.nih.gov/Blast.cgi) | 0 | 0 | 0 | 0 | 0 | 0 | 2 | 0 | 0 | 0 | 0 | 0 | 2 | 0.31 |
| *Cercospora* sp. | 0 | 0 | 0 | 2 | 0 | 0 | 0 | 0 | 0 | 0 | 0 | 0 | 2 | 0.31 |
| [*Cladosporium macrocarpum*](https://blast.ncbi.nlm.nih.gov/Blast.cgi) | 0 | 0 | 0 | 0 | 1 | 0 | 0 | 0 | 0 | 0 | 0 | 1 | 2 | 0.31 |
| [*Didymella glomerata*](https://blast.ncbi.nlm.nih.gov/Blast.cgi) | 0 | 0 | 0 | 0 | 1 | 0 | 0 | 0 | 0 | 0 | 0 | 1 | 2 | 0.31 |
| [*Didymella pinodella*](https://blast.ncbi.nlm.nih.gov/Blast.cgi) | 0 | 0 | 0 | 0 | 0 | 0 | 0 | 0 | 0 | 0 | 1 | 1 | 2 | 0.31 |
| *Fusarium oxysporum* | 0 | 0 | 0 | 2 | 0 | 0 | 0 | 0 | 0 | 0 | 0 | 0 | 2 | 0.31 |
| *Gloeophyllum trabeum* | 0 | 0 | 2 | 0 | 0 | 0 | 0 | 0 | 0 | 0 | 0 | 0 | 2 | 0.31 |
| *Leptosphaeria* sp. | 2 | 0 | 0 | 0 | 0 | 0 | 0 | 0 | 0 | 0 | 0 | 0 | 2 | 0.31 |
| [*Microsphaeropsis* sp.](https://blast.ncbi.nlm.nih.gov/Blast.cgi) | 0 | 0 | 0 | 0 | 0 | 1 | 1 | 0 | 0 | 0 | 0 | 0 | 2 | 0.31 |
| *Nothophoma quercina* | 0 | 0 | 0 | 1 | 0 | 0 | 0 | 0 | 1 | 0 | 0 | 0 | 2 | 0.31 |
| *Nothophoma spiraeae* | 1 | 0 | 0 | 0 | 0 | 0 | 0 | 0 | 0 | 0 | 1 | 0 | 2 | 0.31 |
| *Periconia byssoides* | 0 | 0 | 0 | 1 | 0 | 0 | 0 | 0 | 0 | 1 | 0 | 0 | 2 | 0.31 |
| [*Penicillium polonicum*](https://blast.ncbi.nlm.nih.gov/Blast.cgi) | 0 | 0 | 0 | 0 | 0 | 0 | 0 | 0 | 0 | 2 | 0 | 0 | 2 | 0.31 |
| [*Peyronellaea glomerata*](https://blast.ncbi.nlm.nih.gov/Blast.cgi) | 0 | 0 | 0 | 0 | 0 | 0 | 0 | 0 | 0 | 1 | 0 | 1 | 2 | 0.31 |
| *Phanerochaete chrysosporium* | 0 | 0 | 2 | 0 | 0 | 0 | 0 | 0 | 0 | 0 | 0 | 0 | 2 | 0.31 |
| [*Phoma fungicola*](https://blast.ncbi.nlm.nih.gov/Blast.cgi) | 0 | 0 | 0 | 0 | 0 | 0 | 0 | 0 | 1 | 0 | 0 | 1 | 2 | 0.31 |
| [*Purpureocillium lilacinum*](https://blast.ncbi.nlm.nih.gov/Blast.cgi) | 0 | 0 | 0 | 0 | 0 | 1 | 0 | 0 | 0 | 0 | 0 | 1 | 2 | 0.31 |
| *Talaromyces radicus* | 0 | 0 | 0 | 0 | 0 | 2 | 0 | 0 | 0 | 0 | 0 | 0 | 2 | 0.31 |
| *Wallemia mellicola* | 0 | 0 | 2 | 0 | 0 | 0 | 0 | 0 | 0 | 0 | 0 | 0 | 2 | 0.31 |
| *Zygosporium oscheoides* | 0 | 0 | 2 | 0 | 0 | 0 | 0 | 0 | 0 | 0 | 0 | 0 | 2 | 0.31 |
| [*Actinomucor elegans*](https://blast.ncbi.nlm.nih.gov/Blast.cgi) | 0 | 0 | 0 | 0 | 0 | 0 | 0 | 0 | 0 | 0 | 0 | 1 | 1 | 0.16 |
| [*Allophoma labilis*](https://blast.ncbi.nlm.nih.gov/Blast.cgi) | 0 | 1 | 0 | 0 | 0 | 0 | 0 | 0 | 0 | 0 | 0 | 0 | 1 | 0.16 |
| [*Alternaria consortialis*](https://blast.ncbi.nlm.nih.gov/Blast.cgi) | 0 | 0 | 0 | 0 | 1 | 0 | 0 | 0 | 0 | 0 | 0 | 0 | 1 | 0.16 |
| [*Alternaria japonica*](https://blast.ncbi.nlm.nih.gov/Blast.cgi) | 0 | 0 | 0 | 0 | 0 | 0 | 0 | 0 | 1 | 0 | 0 | 0 | 1 | 0.16 |
| [*Ascochyta medicaginicola*](https://blast.ncbi.nlm.nih.gov/Blast.cgi) | 0 | 0 | 0 | 0 | 0 | 0 | 0 | 0 | 0 | 1 | 0 | 0 | 1 | 0.16 |
| [*Aspergillus creber*](https://blast.ncbi.nlm.nih.gov/Blast.cgi) | 0 | 0 | 0 | 0 | 1 | 0 | 0 | 0 | 0 | 0 | 0 | 0 | 1 | 0.16 |
| *Aspergillus japonicus* | 0 | 0 | 0 | 0 | 0 | 0 | 1 | 0 | 0 | 0 | 0 | 0 | 1 | 0.16 |
| *Aspergillus nidulans* | 0 | 0 | 0 | 0 | 1 | 0 | 0 | 0 | 0 | 0 | 0 | 0 | 1 | 0.16 |
| [*Aspergillus proliferans*](https://blast.ncbi.nlm.nih.gov/Blast.cgi) | 0 | 0 | 0 | 0 | 0 | 0 | 0 | 0 | 1 | 0 | 0 | 0 | 1 | 0.16 |
| *Aspergillus protuberus* | 1 | 0 | 0 | 0 | 0 | 0 | 0 | 0 | 0 | 0 | 0 | 0 | 1 | 0.16 |
| *Aspergillus* sp. | 0 | 0 | 0 | 1 | 0 | 0 | 0 | 0 | 0 | 0 | 0 | 0 | 1 | 0.16 |
| *Aspergillus westerdijkiae* | 1 | 0 | 0 | 0 | 0 | 0 | 0 | 0 | 0 | 0 | 0 | 0 | 1 | 0.16 |
| [*Candida parapsilosis*](https://blast.ncbi.nlm.nih.gov/Blast.cgi) | 0 | 0 | 0 | 0 | 0 | 0 | 0 | 0 | 0 | 0 | 0 | 1 | 1 | 0.16 |
| *Cephalotrichum nanum* | 0 | 0 | 0 | 0 | 0 | 0 | 0 | 1 | 0 | 0 | 0 | 0 | 1 | 0.16 |
| *Cercospora beticola* | 0 | 0 | 0 | 0 | 1 | 0 | 0 | 0 | 0 | 0 | 0 | 0 | 1 | 0.16 |
| [*Cladosporium allicinum*](https://blast.ncbi.nlm.nih.gov/Blast.cgi) | 0 | 0 | 0 | 0 | 0 | 0 | 0 | 0 | 0 | 0 | 1 | 0 | 1 | 0.16 |
| *Cladosporium delicatulum* | 0 | 0 | 0 | 1 | 0 | 0 | 0 | 0 | 0 | 0 | 0 | 0 | 1 | 0.16 |
| *Cladosporium funga* | 0 | 0 | 0 | 1 | 0 | 0 | 0 | 0 | 0 | 0 | 0 | 0 | 1 | 0.16 |
| [*Cladosporium perangustum*](https://blast.ncbi.nlm.nih.gov/Blast.cgi) | 0 | 1 | 0 | 0 | 0 | 0 | 0 | 0 | 0 | 0 | 0 | 0 | 1 | 0.16 |
| [*Cladosporium sinuosum*](https://blast.ncbi.nlm.nih.gov/Blast.cgi) | 0 | 0 | 0 | 0 | 0 | 0 | 0 | 0 | 0 | 0 | 0 | 1 | 1 | 0.16 |
| [*Cladosporium sphaerospermum*](https://blast.ncbi.nlm.nih.gov/Blast.cgi) | 0 | 0 | 0 | 0 | 0 | 0 | 0 | 0 | 0 | 0 | 0 | 1 | 1 | 0.16 |
| [*Cladosporium velox*](https://blast.ncbi.nlm.nih.gov/Blast.cgi) | 0 | 0 | 0 | 0 | 0 | 0 | 0 | 0 | 0 | 0 | 1 | 0 | 1 | 0.16 |
| *Cochliobolus cymbopogonis* | 0 | 0 | 0 | 1 | 0 | 0 | 0 | 0 | 0 | 0 | 0 | 0 | 1 | 0.16 |
| *Coelastrella* sp. | 0 | 0 | 1 | 0 | 0 | 0 | 0 | 0 | 0 | 0 | 0 | 0 | 1 | 0.16 |
| [*Coniothyrium aleuritis*](https://blast.ncbi.nlm.nih.gov/Blast.cgi) | 0 | 0 | 0 | 0 | 0 | 0 | 0 | 0 | 0 | 0 | 0 | 1 | 1 | 0.16 |
| *Coprinopsis atramentaria* | 0 | 0 | 1 | 0 | 0 | 0 | 0 | 0 | 0 | 0 | 0 | 0 | 1 | 0.16 |
| [*Coprinopsis kubickae*](https://blast.ncbi.nlm.nih.gov/Blast.cgi) | 0 | 0 | 0 | 0 | 0 | 0 | 0 | 0 | 0 | 0 | 0 | 1 | 1 | 0.16 |
| *Coriolopsis trogii* | 0 | 0 | 1 | 0 | 0 | 0 | 0 | 0 | 0 | 0 | 0 | 0 | 1 | 0.16 |
| [*Cryptococcus chernovii*](https://blast.ncbi.nlm.nih.gov/Blast.cgi) | 0 | 0 | 0 | 0 | 0 | 0 | 0 | 0 | 0 | 0 | 1 | 0 | 1 | 0.16 |
| [*Cryptococcusmagnus*](https://blast.ncbi.nlm.nih.gov/Blast.cgi) | 0 | 0 | 0 | 0 | 0 | 0 | 0 | 0 | 0 | 0 | 0 | 1 | 1 | 0.16 |
| *Cryptococcus rajasthanensis* | 0 | 0 | 0 | 0 | 0 | 0 | 0 | 1 | 0 | 0 | 0 | 0 | 1 | 0.16 |
| *Cryptococcus* sp. | 0 | 0 | 0 | 0 | 0 | 0 | 1 | 0 | 0 | 0 | 0 | 0 | 1 | 0.16 |
| [*Curvularia hawaiiensis*](https://blast.ncbi.nlm.nih.gov/Blast.cgi) | 0 | 0 | 1 | 0 | 0 | 0 | 0 | 0 | 0 | 0 | 0 | 0 | 1 | 0.16 |
| *Curvularia intermedia* | 0 | 0 | 0 | 1 | 0 | 0 | 0 | 0 | 0 | 0 | 0 | 0 | 1 | 0.16 |
| [*Curvularia kusanoi*](https://blast.ncbi.nlm.nih.gov/Blast.cgi) | 0 | 0 | 0 | 0 | 0 | 0 | 0 | 0 | 0 | 0 | 0 | 1 | 1 | 0.16 |
| *Curvularia lunata* | 0 | 0 | 0 | 1 | 0 | 0 | 0 | 0 | 0 | 0 | 0 | 0 | 1 | 0.16 |
| [*Cymostachys thailandica*](https://blast.ncbi.nlm.nih.gov/Blast.cgi) | 0 | 0 | 0 | 0 | 1 | 0 | 0 | 0 | 0 | 0 | 0 | 0 | 1 | 0.16 |
| [*Dicyma olivacea*](https://blast.ncbi.nlm.nih.gov/Blast.cgi) | 0 | 0 | 0 | 0 | 0 | 0 | 0 | 1 | 0 | 0 | 0 | 0 | 1 | 0.16 |
| *Dothidea insculpta* | 0 | 0 | 0 | 1 | 0 | 0 | 0 | 0 | 0 | 0 | 0 | 0 | 1 | 0.16 |
| [*Dothiorella gregaria*](https://blast.ncbi.nlm.nih.gov/Blast.cgi) | 0 | 0 | 0 | 0 | 0 | 0 | 0 | 0 | 0 | 0 | 0 | 1 | 1 | 0.16 |
| [*Dothiorella* sp.](https://blast.ncbi.nlm.nih.gov/Blast.cgi) | 0 | 0 | 0 | 0 | 0 | 0 | 0 | 0 | 0 | 0 | 0 | 1 | 1 | 0.16 |
| *Exserohilum rostratum* | 0 | 0 | 0 | 1 | 0 | 0 | 0 | 0 | 0 | 0 | 0 | 0 | 1 | 0.16 |
| *Fomitopsis ostreiformis* | 0 | 0 | 1 | 0 | 0 | 0 | 0 | 0 | 0 | 0 | 0 | 0 | 1 | 0.16 |
| *Fusarium delphinoides* | 0 | 0 | 0 | 0 | 0 | 0 | 1 | 0 | 0 | 0 | 0 | 0 | 1 | 0.16 |
| *Fusarium equiseti* | 0 | 0 | 0 | 0 | 0 | 0 | 0 | 0 | 0 | 1 | 0 | 0 | 1 | 0.16 |
| [*Geomyces* sp.](https://blast.ncbi.nlm.nih.gov/Blast.cgi) | 0 | 0 | 0 | 0 | 0 | 0 | 1 | 0 | 0 | 0 | 0 | 0 | 1 | 0.16 |
| [*Granulobasidium vellereum*](https://blast.ncbi.nlm.nih.gov/Blast.cgi) | 0 | 0 | 0 | 0 | 0 | 1 | 0 | 0 | 0 | 0 | 0 | 0 | 1 | 0.16 |
| *Hanseniaspora opuntiae* | 0 | 0 | 1 | 0 | 0 | 0 | 0 | 0 | 0 | 0 | 0 | 0 | 1 | 0.16 |
| *Hypoxylon macrocarpum* | 0 | 0 | 0 | 0 | 0 | 0 | 0 | 0 | 0 | 1 | 0 | 0 | 1 | 0.16 |
| [*Kwoniella shandongensis*](https://blast.ncbi.nlm.nih.gov/Blast.cgi) | 0 | 0 | 0 | 0 | 0 | 0 | 0 | 0 | 0 | 0 | 1 | 0 | 1 | 0.16 |
| *Lachancea lanzarotensis* | 0 | 0 | 0 | 0 | 0 | 0 | 0 | 1 | 0 | 0 | 0 | 0 | 1 | 0.16 |
| [*Latorua caligans*](https://blast.ncbi.nlm.nih.gov/Blast.cgi) | 0 | 0 | 0 | 0 | 0 | 0 | 0 | 0 | 0 | 0 | 1 | 0 | 1 | 0.16 |
| *Leptosphaeria sclerotioides* | 0 | 0 | 0 | 0 | 0 | 0 | 0 | 1 | 0 | 0 | 0 | 0 | 1 | 0.16 |
| [*Leptosphaerulina australis*](https://blast.ncbi.nlm.nih.gov/Blast.cgi) | 0 | 0 | 0 | 0 | 0 | 0 | 0 | 0 | 0 | 0 | 0 | 1 | 1 | 0.16 |
| *Mucor circinelloides* | 1 | 0 | 0 | 0 | 0 | 0 | 0 | 0 | 0 | 0 | 0 | 0 | 1 | 0.16 |
| [*Naganishia antarctica*](https://blast.ncbi.nlm.nih.gov/Blast.cgi) | 0 | 0 | 0 | 0 | 0 | 0 | 1 | 0 | 0 | 0 | 0 | 0 | 1 | 0.16 |
| [*Naganishia* sp.](https://blast.ncbi.nlm.nih.gov/Blast.cgi) | 0 | 0 | 0 | 0 | 0 | 0 | 0 | 0 | 0 | 0 | 0 | 1 | 1 | 0.16 |
| [*Neurospora crassa*](https://blast.ncbi.nlm.nih.gov/Blast.cgi) | 0 | 1 | 0 | 0 | 0 | 0 | 0 | 0 | 0 | 0 | 0 | 0 | 1 | 0.16 |
| [*Papiliotrema aurea*](https://blast.ncbi.nlm.nih.gov/Blast.cgi) | 0 | 0 | 0 | 0 | 0 | 0 | 0 | 0 | 1 | 0 | 0 | 0 | 1 | 0.16 |
| *Paraphoma radicina* | 1 | 0 | 0 | 0 | 0 | 0 | 0 | 0 | 0 | 0 | 0 | 0 | 1 | 0.16 |
| [*Penicillium brevicompactum*](https://blast.ncbi.nlm.nih.gov/Blast.cgi) | 0 | 0 | 0 | 0 | 0 | 0 | 0 | 0 | 1 | 0 | 0 | 0 | 1 | 0.16 |
| *Penicillium charlesii* | 0 | 0 | 1 | 0 | 0 | 0 | 0 | 0 | 0 | 0 | 0 | 0 | 1 | 0.16 |
| [*Penicillium expansum*](https://blast.ncbi.nlm.nih.gov/Blast.cgi) | 0 | 0 | 0 | 0 | 0 | 0 | 0 | 0 | 0 | 0 | 1 | 0 | 1 | 0.16 |
| [*Penicillium indicum*](https://blast.ncbi.nlm.nih.gov/Blast.cgi) | 0 | 1 | 0 | 0 | 0 | 0 | 0 | 0 | 0 | 0 | 0 | 0 | 1 | 0.16 |
| [*Penicillium mallochii*](https://blast.ncbi.nlm.nih.gov/Blast.cgi) | 0 | 1 | 0 | 0 | 0 | 0 | 0 | 0 | 0 | 0 | 0 | 0 | 1 | 0.16 |
| [*Penicillium olsonii*](https://blast.ncbi.nlm.nih.gov/Blast.cgi) | 0 | 0 | 0 | 0 | 0 | 0 | 0 | 0 | 0 | 0 | 0 | 1 | 1 | 0.16 |
| *Penicillium* sp. | 0 | 0 | 0 | 0 | 0 | 0 | 0 | 1 | 0 | 0 | 0 | 0 | 1 | 0.16 |
| [*Periconia macrospinosa*](https://blast.ncbi.nlm.nih.gov/Blast.cgi) | 0 | 0 | 0 | 0 | 0 | 0 | 0 | 0 | 0 | 0 | 0 | 1 | 1 | 0.16 |
| *Periconia neobrittanica* | 0 | 0 | 0 | 0 | 0 | 0 | 0 | 1 | 0 | 0 | 0 | 0 | 1 | 0.16 |
| *Periconia pseudodigitata* | 0 | 0 | 0 | 0 | 0 | 1 | 0 | 0 | 0 | 0 | 0 | 0 | 1 | 0.16 |
| *Periconia* sp. | 0 | 0 | 0 | 0 | 0 | 0 | 0 | 0 | 0 | 0 | 0 | 1 | 1 | 0.16 |
| [*Peyronellaea prosopidis*](https://blast.ncbi.nlm.nih.gov/Blast.cgi) | 0 | 1 | 0 | 0 | 0 | 0 | 0 | 0 | 0 | 0 | 0 | 0 | 1 | 0.16 |
| [*Periconia* sp.](https://blast.ncbi.nlm.nih.gov/Blast.cgi) | 0 | 1 | 0 | 0 | 0 | 0 | 0 | 0 | 0 | 0 | 0 | 0 | 1 | 0.16 |
| *Phaeosphaeria fuckelii* | 0 | 1 | 0 | 0 | 0 | 0 | 0 | 0 | 0 | 0 | 0 | 0 | 1 | 0.16 |
| *Phaeosphaeria* sp. | 0 | 0 | 0 | 0 | 0 | 0 | 0 | 1 | 0 | 0 | 0 | 0 | 1 | 0.16 |
| [*Phaeosphaeriaceae* sp.](https://blast.ncbi.nlm.nih.gov/Blast.cgi) | 0 | 0 | 0 | 0 | 0 | 0 | 0 | 0 | 0 | 0 | 0 | 1 | 1 | 0.16 |
| *Phanerochaete sordida* | 0 | 0 | 1 | 0 | 0 | 0 | 0 | 0 | 0 | 0 | 0 | 0 | 1 | 0.16 |
| [*Phoma betae*](https://blast.ncbi.nlm.nih.gov/Blast.cgi) | 0 | 1 | 0 | 0 | 0 | 0 | 0 | 0 | 0 | 0 | 0 | 0 | 1 | 0.16 |
| [*Phoma medicaginis*](https://blast.ncbi.nlm.nih.gov/Blast.cgi) | 0 | 0 | 0 | 0 | 0 | 0 | 0 | 0 | 0 | 0 | 1 | 0 | 1 | 0.16 |
| *Phoma multirostrata* | 0 | 0 | 0 | 1 | 0 | 0 | 0 | 0 | 0 | 0 | 0 | 0 | 1 | 0.16 |
| [*Phoma* sp.](https://blast.ncbi.nlm.nih.gov/Blast.cgi) | 0 | 0 | 0 | 0 | 0 | 0 | 0 | 0 | 0 | 0 | 0 | 1 | 1 | 0.16 |
| [*Pithomyces chartarum*](https://blast.ncbi.nlm.nih.gov/Blast.cgi) | 0 | 0 | 0 | 0 | 0 | 0 | 0 | 0 | 0 | 0 | 1 | 0 | 1 | 0.16 |
| [*Rhodotorula diobovata*](https://blast.ncbi.nlm.nih.gov/Blast.cgi) | 0 | 0 | 0 | 0 | 0 | 0 | 0 | 0 | 0 | 1 | 0 | 0 | 1 | 0.16 |
| [*Scheleobrachea quadrata*](https://blast.ncbi.nlm.nih.gov/Blast.cgi) | 0 | 0 | 0 | 0 | 0 | 0 | 0 | 0 | 0 | 0 | 0 | 1 | 1 | 0.16 |
| [*Schizophyllum commune*](https://blast.ncbi.nlm.nih.gov/Blast.cgi) | 0 | 0 | 0 | 0 | 0 | 0 | 0 | 0 | 0 | 0 | 1 | 0 | 1 | 0.16 |
| [*Stagonospora* sp.](https://blast.ncbi.nlm.nih.gov/Blast.cgi) | 0 | 1 | 0 | 0 | 0 | 0 | 0 | 0 | 0 | 0 | 0 | 0 | 1 | 0.16 |
| *Stemphylium lycopersici* | 0 | 0 | 0 | 0 | 0 | 1 | 0 | 0 | 0 | 0 | 0 | 0 | 1 | 0.16 |
| *Stemphylium solani* | 0 | 0 | 0 | 1 | 0 | 0 | 0 | 0 | 0 | 0 | 0 | 0 | 1 | 0.16 |
| [*Stemphylium vesicarium*](https://blast.ncbi.nlm.nih.gov/Blast.cgi) | 0 | 0 | 0 | 0 | 0 | 1 | 0 | 0 | 0 | 0 | 0 | 0 | 1 | 0.16 |
| [*Talaromyces pinophilus*](https://blast.ncbi.nlm.nih.gov/Blast.cgi) | 0 | 0 | 0 | 0 | 1 | 0 | 0 | 0 | 0 | 0 | 0 | 0 | 1 | 0.16 |
| [*Talaromyces purpureogenus*](https://blast.ncbi.nlm.nih.gov/Blast.cgi) | 0 | 0 | 0 | 0 | 0 | 0 | 0 | 0 | 0 | 1 | 0 | 0 | 1 | 0.16 |
| [*Talaromyces rogersiae*](https://blast.ncbi.nlm.nih.gov/Blast.cgi) | 0 | 0 | 0 | 0 | 0 | 0 | 0 | 0 | 0 | 1 | 0 | 0 | 1 | 0.16 |
| *Talaromyces stollii* | 0 | 0 | 0 | 0 | 0 | 0 | 0 | 1 | 0 | 0 | 0 | 0 | 1 | 0.16 |
| [*Taphrina betulina*](https://blast.ncbi.nlm.nih.gov/Blast.cgi) | 0 | 1 | 0 | 0 | 0 | 0 | 0 | 0 | 0 | 0 | 0 | 0 | 1 | 0.16 |
| *Teichospora kingiae* | 0 | 0 | 0 | 0 | 0 | 0 | 0 | 1 | 0 | 0 | 0 | 0 | 1 | 0.16 |
| [*Torula herbarum*](https://blast.ncbi.nlm.nih.gov/Blast.cgi) | 0 | 0 | 0 | 0 | 0 | 0 | 0 |  | 1 | 0 | 0 | 0 | 1 | 0.16 |
| *Torula mackenziei* | 0 | 0 | 0 | 0 | 0 | 0 | 0 | 1 | 0 | 0 | 0 | 0 | 1 | 0.16 |
| *Uncultured Cryptococcus* | 0 | 0 | 0 | 0 | 0 | 0 | 0 | 1 | 0 | 0 | 0 | 0 | 1 | 0.16 |
| *Uncultured Ochrocladosporium* | 0 | 0 | 0 | 1 | 0 | 0 | 0 | 0 | 0 | 0 | 0 | 0 | 1 | 0.16 |
| *Valsa nivea* | 0 | 1 | 0 | 0 | 0 | 0 | 0 | 0 | 0 | 0 | 0 | 0 | 1 | 0.16 |
| [*Valsaria insitiva*](https://blast.ncbi.nlm.nih.gov/Blast.cgi) | 0 | 0 | 0 | 0 | 0 | 0 | 1 | 0 | 0 | 0 | 0 | 0 | 1 | 0.16 |
| [*Vishniacozyma carnescens*](https://blast.ncbi.nlm.nih.gov/Blast.cgi) | 0 | 0 | 0 | 0 | 0 | 0 | 1 | 0 | 0 | 0 | 0 | 0 | 1 | 0.16 |
| *Vishniacozyma taibaiensis* | 0 | 0 | 0 | 1 | 0 | 0 | 0 | 0 | 0 | 0 | 0 | 0 | 1 | 0.16 |
| *Camarosporium* sp. | 0 | 0 | 0 | 0 | 0 | 0 | 0 | 0 | 0 | 0 | 0 | 1 | 1 | 0.16 |

**Table S7.** Number of fungal colonies isolated for each species, at each location, from June 2020 to May 2021.

|  | **June 2020** | | | | | | | | | | |
| --- | --- | --- | --- | --- | --- | --- | --- | --- | --- | --- | --- |
| **Fungal Species** | **3CP** | **3CO** | **5CP** | **5CO** | **CDP** | **CDO** | **FDP** | **FDO** | **LIB** | **PS** | **Total** |
| *Alternaria alternata* | 0 | 2 | 3 | 2 | 1 | 1 | 0 | 0 | 1 | 2 | **12** |
| *Alternaria compacta* | 0 | 0 | 0 | 0 | 0 | 0 | 0 | 0 | 1 | 0 | **1** |
| *Alternaria tenuissima* | 0 | 0 | 0 | 1 | 0 | 0 | 0 | 0 | 1 | 2 | **4** |
| *Aspergillus oryzae* | 0 | 0 | 0 | 0 | 0 | 0 | 1 | 0 | 1 | 0 | **2** |
| *Aspergillus protuberus* | 1 | 0 | 0 | 0 | 0 | 0 | 0 | 0 | 0 | 0 | **1** |
| *Aspergillus versicolor* | 0 | 0 | 0 | 0 | 0 | 0 | 0 | 0 | 2 | 0 | **2** |
| *Aspergillus westerdijkiae* | 1 | 0 | 0 | 0 | 0 | 0 | 0 | 0 | 0 | 0 | **1** |
| *Chaetomium globosum* | 0 | 0 | 0 | 0 | 0 | 1 | 0 | 0 | 0 | 0 | **1** |
| *Cladosporium cladosporioides* | 0 | 0 | 1 | 3 | 0 | 0 | 1 | 2 | 5 | 2 | **14** |
| *Cladosporium cucumerinum* | 0 | 0 | 0 | 0 | 0 | 1 | 0 | 0 | 0 | 0 | **1** |
| *Cladosporium oxysporum* | 0 | 0 | 0 | 0 | 0 | 0 | 0 | 0 | 4 | 0 | **4** |
| *Cladosporium* sp. | 0 | 0 | 1 | 0 | 0 | 1 | 0 | 0 | 0 | 1 | **3** |
| *Cladosporium tenuissimum* | 0 | 0 | 0 | 0 | 0 | 0 | 0 | 2 | 4 | 0 | **6** |
| *Cladosporium uredinicola* | 0 | 0 | 0 | 0 | 0 | 0 | 0 | 0 | 2 | 0 | **2** |
| *Epicoccum* sp. | 0 | 0 | 0 | 0 | 0 | 0 | 0 | 0 | 0 | 1 | **1** |
| *Leptosphaeria* sp. | 0 | 0 | 0 | 0 | 0 | 1 | 1 | 0 | 0 | 0 | **1** |
| *Mucor circinelloides* | 0 | 0 | 0 | 0 | 0 | 0 | 0 | 0 | 1 | 0 | **1** |
| *Nothophoma spiraeae* | 0 | 1 | 0 | 0 | 0 | 0 | 0 | 0 | 0 | 0 | **1** |
| *Paraphoma radicina* | 1 | 0 | 0 | 0 | 0 | 0 | 0 | 0 | 0 | 0 | **1** |
| *Sarocladium strictum* | 0 | 0 | 0 | 0 | 0 | 1 | 0 | 2 | 0 | 0 | **3** |
| **Total** | **3** | **3** | **5** | **6** | **1** | **6** | **3** | **6** | **22** | **8** | **63** |
|  | **July 2020** | | | | | | | | | | |
| **Fungal Species** | **3CP** | **3CO** | **5CP** | **5CO** | **CDP** | **CDO** | **FDP** | **FDO** | **LIB** | **PS** | **Total** |
| [*Allophoma labilis*](https://blast.ncbi.nlm.nih.gov/Blast.cgi) | 1 | 0 | 0 | 0 | 0 | 0 | 0 | 0 | 0 | 0 | **1** |
| [*Alternaria alternata*](https://blast.ncbi.nlm.nih.gov/Blast.cgi) | 0 | 1 | 2 | 2 | 2 | 1 | 0 | 1 | 1 | 3 | **13** |
| [*Alternaria angustiovoidea*](https://blast.ncbi.nlm.nih.gov/Blast.cgi) | 1 | 0 | 0 | 0 | 0 | 0 | 0 | 1 | 1 | 1 | **4** |
| [*Alternaria compacta*](https://blast.ncbi.nlm.nih.gov/Blast.cgi) | 0 | 1 | 1 | 0 | 0 | 0 | 0 | 0 | 1 | 1 | **4** |
| [*Alternaria solani*](https://blast.ncbi.nlm.nih.gov/Blast.cgi) | 1 | 1 | 0 | 0 | 0 | 0 | 0 | 0 | 0 | 1 | **3** |
| *Alternaria* sp. | 0 | 1 | 0 | 0 | 0 | 0 | 0 | 0 | 0 | 0 | **1** |
| [*Alternaria tamaricis*](https://blast.ncbi.nlm.nih.gov/Blast.cgi) | 1 | 1 | 0 | 1 | 0 | 1 | 0 | 0 | 0 | 2 | **6** |
| [*Alternaria tenuissima*](https://blast.ncbi.nlm.nih.gov/Blast.cgi) | 0 | 0 | 0 | 0 | 0 | 0 | 0 | 0 | 0 | 1 | **1** |
| [*Aspergillus flavus*](https://blast.ncbi.nlm.nih.gov/Blast.cgi) | 0 | 0 | 1 | 0 | 0 | 0 | 0 | 0 | 0 | 0 | **1** |
| [*Aspergillus niger*](https://blast.ncbi.nlm.nih.gov/Blast.cgi) | 0 | 0 | 1 | 0 | 1 | 0 | 0 | 1 | 0 | 0 | **3** |
| [*Aspergillus ochraceus*](https://blast.ncbi.nlm.nih.gov/Blast.cgi) | 0 | 0 | 1 | 0 | 0 | 0 | 0 | 0 | 0 | 0 | **1** |
| [*Aureobasidium iranianum*](https://blast.ncbi.nlm.nih.gov/Blast.cgi) | 3 | 0 | 0 | 0 | 0 | 0 | 0 | 0 | 0 | 0 | **3** |
| *Botrytis cinerea* | 0 | 1 | 0 | 0 | 0 | 0 | 0 | 0 | 0 | 0 | **1** |
| [*Cladosporium anthropophilum*](https://blast.ncbi.nlm.nih.gov/Blast.cgi) | 0 | 0 | 1 | 0 | 0 | 0 | 0 | 0 | 2 | 0 | **3** |
| [*Cladosporium asperulatum*](https://blast.ncbi.nlm.nih.gov/Blast.cgi) | 0 | 0 | 0 | 0 | 0 | 0 | 0 | 0 | 0 | 1 | **1** |
| [*Cladosporium cladosporioides*](https://blast.ncbi.nlm.nih.gov/Blast.cgi) | 0 | 0 | 2 | 0 | 0 | 1 | 0 | 0 | 0 | 0 | **3** |
| [*Cladosporium herbarum*](https://blast.ncbi.nlm.nih.gov/Blast.cgi) | 0 | 0 | 0 | 0 | 0 | 0 | 0 | 0 | 0 | 1 | **1** |
| [*Cladosporium oxysporum*](https://blast.ncbi.nlm.nih.gov/Blast.cgi) | 0 | 0 | 2 | 0 | 0 | 0 | 0 | 0 | 0 | 0 | **2** |
| [*Cladosporium perangustum*](https://blast.ncbi.nlm.nih.gov/Blast.cgi) | 0 | 0 | 1 | 0 | 0 | 0 | 0 | 0 | 0 | 0 | **1** |
| [*Cladosporium ramotenellum*](https://blast.ncbi.nlm.nih.gov/Blast.cgi) | 0 | 0 | 0 | 0 | 0 | 0 | 0 | 0 | 0 | 1 | **1** |
| [*Cladosporium tenuissimum*](https://blast.ncbi.nlm.nih.gov/Blast.cgi) | 0 | 0 | 3 | 2 | 0 | 0 | 0 | 0 | 0 | 1 | **6** |
| [*Didymella macrostoma*](https://blast.ncbi.nlm.nih.gov/Blast.cgi) | 0 | 0 | 0 | 0 | 0 | 0 | 0 | 1 | 0 | 0 | **1** |
| [*Periconia* sp.](https://blast.ncbi.nlm.nih.gov/Blast.cgi) | 0 | 0 | 1 | 0 | 0 | 0 | 0 | 0 | 0 | 0 | **1** |
| [*Neurospora crassa*](https://blast.ncbi.nlm.nih.gov/Blast.cgi) | 0 | 0 | 0 | 0 | 0 | 0 | 1 | 0 | 0 | 0 | **1** |
| [*Penicillium indicum*](https://blast.ncbi.nlm.nih.gov/Blast.cgi) | 0 | 0 | 0 | 0 | 0 | 0 | 0 | 0 | 1 | 0 | **1** |
| [*Penicillium mallochii*](https://blast.ncbi.nlm.nih.gov/Blast.cgi) | 0 | 0 | 0 | 0 | 1 | 0 | 0 | 0 | 0 | 0 | **1** |
| [*Penicillium oxalicum*](https://blast.ncbi.nlm.nih.gov/Blast.cgi) | 0 | 0 | 1 | 0 | 0 | 0 | 0 | 0 | 0 | 0 | **1** |
| [*Peyronellaea prosopidis*](https://blast.ncbi.nlm.nih.gov/Blast.cgi) | 0 | 1 | 0 | 0 | 0 | 0 | 0 | 0 | 0 | 0 | **1** |
| *Phaeosphaeria fuckelii* | 0 | 0 | 1 | 0 | 0 | 0 | 0 | 0 | 0 | 0 | **1** |
| [*Phoma betae*](https://blast.ncbi.nlm.nih.gov/Blast.cgi) | 0 | 0 | 1 | 0 | 0 | 0 | 0 | 0 | 0 | 0 | **1** |
| [*Stagonospora* sp.](https://blast.ncbi.nlm.nih.gov/Blast.cgi) | 0 | 0 | 1 | 0 | 0 | 0 | 0 | 0 | 0 | 0 | **1** |
| [*Taphrina betulina*](https://blast.ncbi.nlm.nih.gov/Blast.cgi) | 0 | 0 | 0 | 0 | 0 | 1 | 0 | 0 | 0 | 0 | **1** |
| *Valsa nivea* | 1 | 0 | 0 | 0 | 0 | 0 | 0 | 0 | 0 | 0 | **1** |
| **Total** | **8** | **7** | **20** | **5** | **4** | **4** | **1** | **4** | **6** | **13** | **72** |
|  | **Aug 2020** | | | | | | | | | | |
| **Fungal Species** | **3CP** | **3CO** | **5CP** | **5CO** | **CDP** | **CDO** | **FDP** | **FDO** | **LIB** | **PS** | **Total** |
| [*Alternaria alternata*](https://blast.ncbi.nlm.nih.gov/Blast.cgi) | 0 | 1 | 0 | 0 | 0 | 1 | 0 | 1 | 0 | 1 | **4** |
| [*Aspergillus niger*](https://blast.ncbi.nlm.nih.gov/Blast.cgi) | 0 | 0 | 0 | 0 | 0 | 1 | 0 | 0 | 0 | 0 | **1** |
| [*Cladosporium anthropophilum*](https://blast.ncbi.nlm.nih.gov/Blast.cgi) | 0 | 0 | 0 | 0 | 0 | 0 | 0 | 0 | 0 | 1 | **1** |
| *Cladosporium cladosporioides* | 0 | 0 | 0 | 0 | 0 | 0 | 0 | 0 | 0 | 1 | **1** |
| *Cladosporium halotolerans* | 0 | 0 | 0 | 0 | 1 | 0 | 0 | 0 | 0 | 0 | **1** |
| [*Cladosporium oxysporum*](https://blast.ncbi.nlm.nih.gov/Blast.cgi) | 0 | 0 | 0 | 0 | 0 | 0 | 0 | 0 | 0 | 1 | **1** |
| [*Cladosporium tenuissimum*](https://blast.ncbi.nlm.nih.gov/Blast.cgi) | 0 | 0 | 0 | 0 | 0 | 0 | 0 | 1 | 0 | 1 | **2** |
| *Coelastrella* sp. | 0 | 0 | 0 | 1 | 0 | 0 | 0 | 0 | 0 | 0 | **1** |
| *Coprinopsis atramentaria* | 0 | 0 | 0 | 0 | 1 | 0 | 0 | 0 | 0 | 0 | **1** |
| *Coriolopsis trogii* | 0 | 0 | 0 | 0 | 0 | 0 | 0 | 1 | 0 | 0 | **1** |
| [*Curvularia hawaiiensis*](https://blast.ncbi.nlm.nih.gov/Blast.cgi) | 1 | 0 | 0 | 0 | 0 | 0 | 0 | 0 | 0 | 0 | **1** |
| [*Didymella macrostoma*](https://blast.ncbi.nlm.nih.gov/Blast.cgi) | 1 | 0 | 0 | 1 | 0 | 0 | 0 | 0 | 0 | 0 | **2** |
| *Epicoccum nigrum* | 0 | 1 | 0 | 0 | 0 | 0 | 0 | 0 | 0 | 1 | **2** |
| *Filobasidium magnum* | 0 | 1 | 0 | 0 | 2 | 0 | 0 | 0 | 0 | 0 | **3** |
| *Fomitopsis ostreiformis* | 0 | 0 | 0 | 0 | 0 | 0 | 0 | 1 | 0 | 0 | **1** |
| *Gloeophyllum trabeum* | 0 | 0 | 0 | 0 | 2 | 0 | 0 | 0 | 0 | 0 | **2** |
| *Hanseniaspora opuntiae* | 0 | 0 | 0 | 0 | 1 | 0 | 0 | 0 | 0 | 0 | **1** |
| *Neurospora tetrasperma* | 0 | 2 | 0 | 0 | 0 | 3 | 0 | 0 | 0 | 0 | **5** |
| *Penicillium charlesii* | 0 | 0 | 1 | 0 | 0 | 0 | 0 | 0 | 0 | 0 | **1** |
| *Phanerochaete chrysosporium* | 0 | 0 | 0 | 0 | 2 | 0 | 0 | 0 | 0 | 0 | **2** |
| *Phanerochaete sordida* | 0 | 0 | 0 | 0 | 0 | 0 | 0 | 1 | 0 | 0 | **1** |
| *Wallemia mellicola* | 0 | 0 | 2 | 0 | 0 | 0 | 0 | 0 | 0 | 0 | **2** |
| *Zygosporium oscheoides* | 0 | 0 | 0 | 0 | 0 | 0 | 0 | 0 | 0 | 2 | **2** |
| **Total** | **2** | **5** | **3** | **2** | **9** | **5** | **0** | **5** | **0** | **8** | **39** |
|  | **Sep 2020** | | | | | | | | | | |
| **Fungal Species** | **3CP** | **3CO** | **5CP** | **5CO** | **CDP** | **CDO** | **FDP** | **FDO** | **LIB** | **PS** | **Total** |
| *Alternaria alternata* | 2 | 0 | 2 | 6 | 0 | 0 | 1 | 0 | 13 | 11 | **35** |
| *Alternaria angustiovoidea* | 1 | 0 | 0 | 1 | 0 | 0 | 0 | 0 | 0 | 0 | **2** |
| *Alternaria compacta* | 1 | 0 | 0 | 0 | 0 | 0 | 2 | 0 | 1 | 0 | **4** |
| *Alternaria porri* | 1 | 0 | 0 | 0 | 0 | 0 | 0 | 0 | 0 | 0 | **1** |
| *Alternaria solani* | 1 | 0 | 0 | 1 | 0 | 0 | 0 | 0 | 0 | 0 | **2** |
| *Alternaria* sp. | 0 | 0 | 0 | 0 | 0 | 0 | 0 | 0 | 3 | 1 | **4** |
| *Alternaria tamaricis* | 0 | 0 | 0 | 0 | 0 | 0 | 0 | 1 | 0 | 0 | **1** |
| *Alternaria tenuissima* | 0 | 0 | 2 | 2 | 0 | 0 | 1 | 0 | 4 | 2 | **11** |
| *Aspergillus flavus* | 0 | 0 | 0 | 0 | 0 | 1 | 0 | 1 | 0 | 0 | **2** |
| *Aspergillus niger* | 0 | 0 | 0 | 0 | 1 | 0 | 0 | 0 | 0 | 0 | **1** |
| *Aspergillus* sp. | 0 | 0 | 0 | 0 | 0 | 0 | 1 | 0 | 0 | 0 | **1** |
| *Cercospora* sp. | 0 | 0 | 1 | 0 | 0 | 1 | 0 | 0 | 0 | 0 | **2** |
| *Cladosporium anthropophilum* | 3 | 0 | 1 | 1 | 0 | 1 | 2 | 0 | 1 | 2 | **11** |
| *Cladosporium asperulatum* | 1 | 0 | 0 | 0 | 0 | 0 | 0 | 0 | 0 | 0 | **1** |
| *Cladosporium cladosporioides* | 6 | 0 | 5 | 3 | 2 | 1 | 3 | 1 | 3 | 4 | **28** |
| *Cladosporium delicatulum* | 0 | 0 | 0 | 1 | 0 | 0 | 0 | 0 | 0 | 0 | **1** |
| *Cladosporium funga* | 0 | 0 | 0 | 0 | 0 | 0 | 1 | 0 | 0 | 0 | **1** |
| *Cladosporium halotolerans* | 0 | 0 | 0 | 0 | 1 | 0 | 0 | 0 | 0 | 0 | **1** |
| [*Cladosporium oxysporum*](https://blast.ncbi.nlm.nih.gov/Blast.cgi) | 1 | 0 | 0 | 2 | 0 | 0 | 0 | 0 | 3 | 1 | **7** |
| *Cladosporium ramotenellum* | 0 | 0 | 0 | 0 | 0 | 2 | 1 | 1 | 1 | 0 | **5** |
| *Cladosporium* sp. | 0 | 0 | 0 | 0 | 0 | 0 | 0 | 1 | 0 | 2 | **3** |
| *Cladosporium tenuissimum* | 0 | 0 | 0 | 4 | 0 | 0 | 0 | 1 | 0 | 0 | **5** |
| *Cochliobolus cymbopogonis* | 0 | 0 | 0 | 0 | 0 | 0 | 0 | 0 | 1 | 0 | **1** |
| *Curvularia intermedia* | 0 | 0 | 0 | 0 | 0 | 0 | 0 | 0 | 0 | 1 | **1** |
| *Curvularia lunata* | 0 | 0 | 0 | 0 | 0 | 0 | 0 | 0 | 0 | 1 | **1** |
| *Dothidea insculpta* | 0 | 0 | 0 | 0 | 0 | 0 | 0 | 0 | 1 | 0 | **1** |
| *Epicoccum nigrum* | 7 | 0 | 1 | 10 | 0 | 0 | 0 | 1 | 4 | 8 | **31** |
| *Epicoccum* sp. | 0 | 0 | 0 | 1 | 0 | 0 | 0 | 0 | 0 | 0 | **1** |
| *Exserohilum rostratum* | 0 | 0 | 0 | 0 | 0 | 0 | 0 | 0 | 0 | 1 | **1** |
| *Fusarium incarnatum* | 0 | 0 | 2 | 3 | 0 | 1 | 0 | 0 | 0 | 0 | **6** |
| *Fusarium oxysporum f.* | 1 | 0 | 0 | 1 | 0 | 0 | 0 | 0 | 0 | 0 | **2** |
| *Neurospora tetrasperma* | 1 | 0 | 1 | 0 | 0 | 0 | 0 | 0 | 0 | 0 | **2** |
| *Nothophoma quercina* | 1 | 0 | 0 | 0 | 0 | 0 | 0 | 0 | 0 | 0 | **1** |
| *Penicillium oxalicum* | 0 | 0 | 0 | 2 | 0 | 0 | 0 | 0 | 0 | 0 | **2** |
| *Periconia byssoides* | 0 | 0 | 0 | 1 | 0 | 0 | 0 | 0 | 0 | 0 | **1** |
| *Phoma multirostrata* | 0 | 0 | 0 | 1 | 0 | 0 | 0 | 0 | 0 | 0 | **1** |
| [*Sarocladium strictum*](https://blast.ncbi.nlm.nih.gov/Blast.cgi) | 0 | 0 | 0 | 1 | 0 | 0 | 0 | 0 | 0 | 0 | **1** |
| *Stemphylium solani* | 0 | 0 | 0 | 1 | 0 | 0 | 0 | 0 | 0 | 0 | **1** |
| [*Talaromyces funiculosus*](https://blast.ncbi.nlm.nih.gov/Blast.cgi) | 0 | 0 | 0 | 0 | 0 | 0 | 1 | 0 | 0 | 0 | **1** |
| *Uncultured Ochrocladosporium* | 0 | 0 | 0 | 0 | 0 | 0 | 0 | 1 | 0 | 0 | **1** |
| *Vishniacozyma taibaiensis* | 0 | 0 | 0 | 0 | 0 | 0 | 0 | 0 | 1 | 0 | **1** |
| **Total** | **27** | **0** | **15** | **42** | **4** | **7** | **13** | **8** | **36** | **34** | **186** |
|  | **Oct 2020** | | | | | | | | | | |
| **Fungal Species** | **3CP** | **3CO** | **5CP** | **5CO** | **CDP** | **CDO** | **FDP** | **FDO** | **LIB** | **PS** | **Total** |
| *Alternaria alternata* | 0 | 1 | 0 | 0 | 1 | 0 | 0 | 0 | 0 | 1 | **3** |
| [*Alternaria consortialis*](https://blast.ncbi.nlm.nih.gov/Blast.cgi) | 0 | 1 | 0 | 0 | 0 | 0 | 0 | 0 | 0 | 0 | **1** |
| *Alternaria tenuissima* | 0 | 0 | 1 | 0 | 0 | 0 | 0 | 0 | 0 | 0 | **1** |
| [*Arthrinium arundinis*](https://blast.ncbi.nlm.nih.gov/Blast.cgi) | 0 | 0 | 0 | 0 | 0 | 1 | 0 | 0 | 1 | 0 | **2** |
| [*Aspergillus creber*](https://blast.ncbi.nlm.nih.gov/Blast.cgi) | 1 | 0 | 0 | 0 | 0 | 0 | 0 | 0 | 0 | 0 | **1** |
| *Aspergillus nidulans* | 0 | 0 | 0 | 0 | 0 | 0 | 0 | 0 | 0 | 1 | **1** |
| [*Aspergillus ochraceus*](https://blast.ncbi.nlm.nih.gov/Blast.cgi) | 1 | 0 | 0 | 0 | 0 | 0 | 0 | 0 | 0 | 0 | **1** |
| [*Aspergillus sydowii*](https://blast.ncbi.nlm.nih.gov/Blast.cgi) | 0 | 0 | 0 | 0 | 1 | 0 | 0 | 0 | 0 | 0 | **1** |
| *Cercospora beticola* | 0 | 0 | 0 | 0 | 0 | 0 | 0 | 1 | 0 | 0 | **1** |
| [*Cladosporium anthropophilum*](https://blast.ncbi.nlm.nih.gov/Blast.cgi) | 0 | 0 | 0 | 0 | 1 | 1 | 0 | 0 | 0 | 0 | **2** |
| [*Cladosporium cladosporioides*](https://blast.ncbi.nlm.nih.gov/Blast.cgi) | 0 | 0 | 0 | 0 | 0 | 0 | 0 | 0 | 0 | 1 | **1** |
| [*Cladosporium macrocarpum*](https://blast.ncbi.nlm.nih.gov/Blast.cgi) | 0 | 0 | 0 | 0 | 0 | 1 | 0 | 0 | 0 | 0 | **1** |
| [*Cladosporium oryzae*](https://blast.ncbi.nlm.nih.gov/Blast.cgi) | 0 | 0 | 0 | 0 | 0 | 1 | 0 | 0 | 0 | 0 | **1** |
| [*Cladosporium tenuissimum*](https://blast.ncbi.nlm.nih.gov/Blast.cgi) | 0 | 0 | 0 | 0 | 0 | 0 | 0 | 0 | 0 | 1 | **1** |
| [*Cladosporium uredinicola*](https://blast.ncbi.nlm.nih.gov/Blast.cgi) | 0 | 0 | 0 | 0 | 0 | 0 | 0 | 0 | 0 | 1 | **1** |
| [*Cymostachys thailandica*](https://blast.ncbi.nlm.nih.gov/Blast.cgi) | 0 | 1 | 0 | 0 | 0 | 0 | 0 | 0 | 0 | 0 | **1** |
| [*Didymella glomerata*](https://blast.ncbi.nlm.nih.gov/Blast.cgi) | 0 | 0 | 0 | 0 | 0 | 0 | 0 | 0 | 0 | 1 | **1** |
| *Epicoccum nigrum* | 0 | 1 | 0 | 0 | 1 | 0 | 1 | 0 | 0 | 0 | **3** |
| [*Filobasidium magnum*](https://blast.ncbi.nlm.nih.gov/Blast.cgi) | 0 | 0 | 0 | 0 | 0 | 0 | 0 | 0 | 1 | 0 | **1** |
| *Naganishia albida* | 0 | 0 | 0 | 0 | 0 | 1 | 0 | 0 | 0 | 0 | **1** |
| [*Talaromyces funiculosus*](https://blast.ncbi.nlm.nih.gov/Blast.cgi) | 0 | 0 | 0 | 0 | 0 | 0 | 1 | 0 | 0 | 0 | **1** |
| [*Talaromyces pinophilus*](https://blast.ncbi.nlm.nih.gov/Blast.cgi) | 0 | 0 | 0 | 0 | 0 | 0 | 0 | 0 | 0 | 1 | **1** |
| **Total** | **2** | **4** | **1** | **0** | **4** | **5** | **2** | **1** | **2** | **7** | **28** |
|  | **Nov 2020** | | | | | | | | | | |
| **Fungal Species** | **3CP** | **3CO** | **5CP** | **5CO** | **CDP** | **CDO** | **FDP** | **FDO** | **LIB** | **PS** | **Total** |
| [*Aspergillus flavus*](https://blast.ncbi.nlm.nih.gov/Blast.cgi) | 0 | 1 | 0 | 0 | 0 | 0 | 0 | 1 | 0 | 0 | **2** |
| *Aspergillus sydowii* | 0 | 0 | 0 | 0 | 0 | 0 | 0 | 0 | 0 | 1 | **1** |
| [*Aspergillus tubingensis*](https://blast.ncbi.nlm.nih.gov/Blast.cgi) | 0 | 0 | 0 | 0 | 0 | 0 | 0 | 0 | 1 | 1 | **2** |
| *Cladosporium halotolerans* | 0 | 0 | 1 | 0 | 0 | 0 | 0 | 0 | 0 | 0 | **1** |
| *Cladosporium oryzae* | 0 | 0 | 0 | 0 | 0 | 0 | 0 | 0 | 1 | 0 | **1** |
| [*Granulobasidium vellereum*](https://blast.ncbi.nlm.nih.gov/Blast.cgi) | 0 | 1 | 0 | 0 | 0 | 0 | 0 | 0 | 0 | 0 | **1** |
| [*Microsphaeropsis* sp.](https://blast.ncbi.nlm.nih.gov/Blast.cgi) | 0 | 0 | 0 | 0 | 1 | 0 | 0 | 0 | 0 | 0 | **1** |
| [*Naganishia uzbekistanensis*](https://blast.ncbi.nlm.nih.gov/Blast.cgi) | 2 | 0 | 0 | 0 | 0 | 0 | 0 | 0 | 0 | 0 | **2** |
| *Penicillium concavorugulosum* | 0 | 0 | 0 | 0 | 0 | 0 | 0 | 0 | 2 | 0 | **2** |
| *Penicillium oxalicum* | 0 | 0 | 1 | 0 | 0 | 0 | 0 | 0 | 0 | 0 | **1** |
| *Periconia pseudodigitata* | 0 | 0 | 1 | 0 | 0 | 0 | 0 | 0 | 0 | 0 | **1** |
| [*Purpureocillium lilacinum*](https://blast.ncbi.nlm.nih.gov/Blast.cgi) | 0 | 0 | 0 | 1 | 0 | 0 | 0 | 0 | 0 | 0 | **1** |
| *Stemphylium lycopersici* | 0 | 0 | 0 | 1 | 0 | 0 | 0 | 0 | 0 | 0 | **1** |
| [*Stemphylium vesicarium*](https://blast.ncbi.nlm.nih.gov/Blast.cgi) | 0 | 0 | 0 | 0 | 0 | 0 | 0 | 0 | 0 | 1 | **1** |
| *Talaromyces radicus* | 0 | 0 | 0 | 0 | 0 | 0 | 0 | 0 | 1 | 1 | **2** |
| *Torula caligans* | 0 | 0 | 0 | 0 | 0 | 0 | 0 | 0 | 0 | 1 | **1** |
| **Total** | **2** | **2** | **3** | **2** | **1** | **0** | **0** | **1** | **5** | **5** | **21** |
|  | **Dec 2020** | | | | | | | | | | |
| **Fungal Species** | **3CP** | **3CO** | **5CP** | **5CO** | **CDP** | **CDO** | **FDP** | **FDO** | **LIB** | **PS** | **Total** |
| [*Alternaria alternata*](https://blast.ncbi.nlm.nih.gov/Blast.cgi) | 1 | 0 | 0 | 0 | 0 | 2 | 0 | 0 | 0 | 3 | **6** |
| [*Alternaria porri*](https://blast.ncbi.nlm.nih.gov/Blast.cgi) | 0 | 0 | 0 | 0 | 0 | 1 | 0 | 0 | 0 | 0 | **1** |
| *Aspergillus flavus* | 0 | 0 | 0 | 0 | 0 | 0 | 0 | 0 | 0 | 1 | **1** |
| *Aspergillus japonicus* | 0 | 0 | 0 | 0 | 0 | 0 | 0 | 0 | 1 | 0 | **1** |
| [*Aureobasidium pullulans*](https://blast.ncbi.nlm.nih.gov/Blast.cgi) | 0 | 0 | 0 | 0 | 1 | 0 | 0 | 0 | 0 | 0 | **1** |
| [*Aureobasidium* sp.](https://blast.ncbi.nlm.nih.gov/Blast.cgi) | 0 | 0 | 1 | 1 | 0 | 0 | 0 | 0 | 0 | 0 | **2** |
| *Chaetomium globosum* | 0 | 0 | 0 | 0 | 1 | 0 | 0 | 0 | 0 | 0 | **1** |
| [*Cladosporium anthropophilum*](https://blast.ncbi.nlm.nih.gov/Blast.cgi) | 0 | 0 | 1 | 0 | 0 | 1 | 0 | 1 | 0 | 1 | **4** |
| [*Cladosporium asperulatum*](https://blast.ncbi.nlm.nih.gov/Blast.cgi) | 1 | 0 | 1 | 0 | 0 | 0 | 0 | 1 | 0 | 0 | **3** |
| [*Cladosporium cladosporioides*](https://blast.ncbi.nlm.nih.gov/Blast.cgi) | 0 | 0 | 1 | 0 | 0 | 0 | 0 | 0 | 0 | 1 | **2** |
| [*Cladosporium cucumerinum*](https://blast.ncbi.nlm.nih.gov/Blast.cgi) | 1 | 0 | 0 | 0 | 0 | 0 | 0 | 0 | 0 | 0 | **1** |
| *Cladosporium tenuissimum* | 0 | 0 | 0 | 0 | 0 | 0 | 0 | 0 | 0 | 1 | **1** |
| *Cryptococcus* sp. | 1 | 0 | 0 | 0 | 0 | 0 | 0 | 0 | 0 | 0 | **1** |
| [*Didymella* sp.](https://blast.ncbi.nlm.nih.gov/Blast.cgi) | 0 | 0 | 0 | 0 | 0 | 0 | 0 | 0 | 0 | 1 | **1** |
| *Fusarium delphinoides* | 0 | 0 | 0 | 0 | 0 | 0 | 0 | 0 | 0 | 1 | **1** |
| [*Geomyces* sp.](https://blast.ncbi.nlm.nih.gov/Blast.cgi) | 0 | 0 | 0 | 0 | 0 | 1 | 0 | 0 | 0 | 0 | **1** |
| [*Microsphaeropsis* sp.](https://blast.ncbi.nlm.nih.gov/Blast.cgi) | 0 | 0 | 0 | 0 | 0 | 1 | 0 | 0 | 0 | 0 | **1** |
| [*Naganishia albida*](https://blast.ncbi.nlm.nih.gov/Blast.cgi) | 0 | 0 | 0 | 0 | 0 | 1 | 0 | 0 | 0 | 1 | **2** |
| [*Naganishia antarctica*](https://blast.ncbi.nlm.nih.gov/Blast.cgi) | 0 | 0 | 0 | 1 | 0 | 0 | 0 | 0 | 0 | 0 | **1** |
| [*Valsaria insitiva*](https://blast.ncbi.nlm.nih.gov/Blast.cgi) | 0 | 0 | 0 | 0 | 0 | 1 | 0 | 0 | 0 | 0 | **1** |
| [*Vishniacozyma carnescens*](https://blast.ncbi.nlm.nih.gov/Blast.cgi) | 0 | 0 | 0 | 0 | 0 | 0 | 0 | 0 | 0 | 1 | **1** |
| **Total** | **4** | **0** | **4** | **2** | **2** | **8** | **0** | **2** | **1** | **11** | **34** |
|  | **Jan 2021** | | | | | | | | | | |
| **Fungal Species** | **3CP** | **3CO** | **5CP** | **5CO** | **CDP** | **CDO** | **FDP** | **FDO** | **LIB** | **PS** | **Total** |
| *Alternaria alternata* | 0 | 0 | 0 | 0 | 1 | 0 | 2 | 1 | 0 | 1 | **5** |
| *Alternaria compacta* | 1 | 0 | 0 | 1 | 0 | 0 | 0 | 0 | 0 | 0 | **2** |
| *Alternaria tenuissima* | 0 | 0 | 0 | 0 | 0 | 0 | 0 | 0 | 0 | 1 | **1** |
| *Arthrinium marii* | 0 | 0 | 0 | 1 | 0 | 0 | 0 | 0 | 0 | 1 | **2** |
| *Arthrinium saccharicola* | 2 | 0 | 0 |  | 0 | 0 | 0 | 0 | 0 | 0 | **2** |
| [*Aspergillus niger*](https://blast.ncbi.nlm.nih.gov/Blast.cgi) | 0 | 0 | 0 | 0 | 0 | 0 | 1 | 0 | 0 | 0 | **1** |
| *Aureobasidium pullulans* | 0 | 0 | 0 | 0 | 1 | 1 | 0 | 0 | 0 | 0 | **2** |
| *Cephalotrichum nanum* | 0 | 0 | 0 | 0 | 0 | 0 | 1 | 0 | 0 | 0 | **1** |
| *Chaetomium globosum* | 0 | 0 | 0 | 0 | 0 | 0 | 1 | 0 | 0 | 1 | **2** |
| [*Cladosporium asperulatum*](https://blast.ncbi.nlm.nih.gov/Blast.cgi) | 0 | 0 | 0 | 1 | 0 | 0 | 0 | 0 | 0 | 0 | **1** |
| *Cladosporium cladosporioides* | 0 | 1 | 0 | 6 | 1 | 0 | 0 | 0 | 0 | 1 | **9** |
| *Cladosporium halotolerans* | 0 | 0 | 0 | 2 | 0 | 0 | 0 | 0 | 0 | 0 | **2** |
| *Cladosporium oryzae* | 0 | 0 | 0 | 0 | 1 | 0 | 0 | 0 | 0 | 0 | **1** |
| *Cladosporium tenuissimum* | 0 | 0 | 0 | 2 | 1 | 0 | 1 | 0 | 0 | 0 | **4** |
| *Cryptococcus rajasthanensis* | 0 | 1 | 0 | 0 | 0 | 0 | 0 | 0 | 0 | 0 | **1** |
| [*Dicyma olivacea*](https://blast.ncbi.nlm.nih.gov/Blast.cgi) | 0 | 0 | 0 | 0 | 1 | 0 | 0 | 0 | 0 | 0 | **1** |
| *Didymella* sp. | 0 | 0 | 0 | 2 | 0 | 0 | 0 | 0 | 0 | 0 | **2** |
| *Lachancea lanzarotensis* | 0 | 0 | 0 | 0 | 0 | 0 | 0 | 0 | 0 | 1 | **1** |
| *Leptosphaeria sclerotioides* | 0 | 0 | 0 | 0 | 0 | 0 | 1 | 0 | 0 | 0 | **1** |
| *Penicillium concavorugulosum* | 0 | 0 | 0 | 0 | 0 | 0 | 2 | 0 | 0 | 0 | **2** |
| *Penicillium* sp. | 0 | 0 | 0 | 1 | 0 | 0 | 0 | 0 | 0 | 0 | **1** |
| *Periconia neobrittanica* | 0 | 0 | 0 | 0 | 0 | 1 | 0 | 0 | 0 | 0 | **1** |
| *Phaeosphaeria* sp. | 0 | 0 | 0 | 0 | 0 | 0 | 0 | 0 | 0 | 1 | **1** |
| *Talaromyces funiculosus* | 0 | 0 | 0 | 0 | 0 | 0 | 1 | 0 | 0 | 0 | **1** |
| *Talaromyces stollii* | 0 | 0 | 0 | 0 | 0 | 0 | 1 | 0 | 0 | 0 | **1** |
| *Talaromyces verruculosus* | 0 | 0 | 0 | 1 | 0 | 0 | 1 | 0 | 0 | 0 | **2** |
| *Teichospora kingiae* | 0 | 0 | 0 | 0 | 0 | 0 | 0 | 0 | 0 | 1 | **1** |
| *Torula caligans* | 0 | 0 | 0 | 1 | 0 | 0 | 0 | 0 | 0 | 0 | **1** |
| *Torula mackenziei* | 0 | 0 | 0 | 0 | 0 | 0 | 0 | 0 | 0 | 1 | **1** |
| *Uncultured Cryptococcus* | 1 | 0 | 0 | 0 | 0 | 0 | 0 | 0 | 0 | 0 | **1** |
| **Total** | **4** | **2** | **0** | **18** | **6** | **2** | **12** | **1** | **0** | **9** | **54** |
|  | **Feb 2021** | | | | | | | | | | |
| **Fungal Species** | **3CP** | **3CO** | **5CP** | **5CO** | **CDP** | **CDO** | **FDP** | **FDO** | **LIB** | **PS** | **Total** |
| [*Alternaria alternata*](https://blast.ncbi.nlm.nih.gov/Blast.cgi) | 4 | 0 | 0 | 0 | 2 | 1 | 2 | 0 | 1 | 0 | **10** |
| [*Alternaria compacta*](https://blast.ncbi.nlm.nih.gov/Blast.cgi) | 0 | 0 | 0 | 0 | 0 | 0 | 1 | 0 | 0 | 0 | **1** |
| [*Alternaria japonica*](https://blast.ncbi.nlm.nih.gov/Blast.cgi) | 0 | 0 | 0 |  | 1 | 0 | 0 | 0 | 0 | 0 | **1** |
| [*Aspergillus proliferans*](https://blast.ncbi.nlm.nih.gov/Blast.cgi) | 1 | 0 | 0 | 0 | 0 | 0 | 0 | 0 | 0 | 0 | **1** |
| [*Chaetomium globosum*](https://blast.ncbi.nlm.nih.gov/Blast.cgi) | 1 | 0 | 0 | 0 | 0 | 0 | 0 | 0 | 0 | 0 | **1** |
| [*Cladosporium cladosporioides*](https://blast.ncbi.nlm.nih.gov/Blast.cgi) | 0 | 0 | 1 | 1 | 0 | 0 | 0 | 0 | 1 | 0 | **3** |
| [*Epicoccum nigrum*](https://blast.ncbi.nlm.nih.gov/Blast.cgi) | 0 | 0 | 0 | 0 | 1 | 0 | 0 | 0 | 0 | 0 | **1** |
| [*Nothophoma quercina*](https://blast.ncbi.nlm.nih.gov/Blast.cgi) | 0 | 0 | 1 | 0 | 0 | 0 | 0 | 0 | 0 | 0 | **1** |
| [*Papiliotrema aurea*](https://blast.ncbi.nlm.nih.gov/Blast.cgi) | 0 | 0 | 0 | 0 | 0 | 0 | 0 | 0 | 1 | 0 | **1** |
| [*Penicillium brevicompactum*](https://blast.ncbi.nlm.nih.gov/Blast.cgi) | 0 | 0 | 1 | 0 | 0 | 0 | 0 | 0 | 0 | 0 | **1** |
| [*Phoma fungicola*](https://blast.ncbi.nlm.nih.gov/Blast.cgi) | 1 | 0 | 0 | 0 | 0 | 0 | 0 | 0 | 0 | 0 | **1** |
| [*Torula caligans*](https://blast.ncbi.nlm.nih.gov/Blast.cgi) | 1 | 0 | 0 | 0 | 0 | 0 | 0 | 0 | 0 | 0 | **1** |
| [*Torula herbarum*](https://blast.ncbi.nlm.nih.gov/Blast.cgi) | 0 | 0 | 0 | 0 |  | 1 | 0 | 0 | 0 | 0 | **1** |
| **Total** | **8** | **0** | **3** | **1** | **4** | **2** | **3** | **0** | **3** | **0** | **24** |
|  | **Mar 2021** | | | | | | | | | | |
| **Fungal Species** | **3CP** | **3CO** | **5CP** | **5CO** | **CDP** | **CDO** | **FDP** | **FDO** | **LIB** | **PS** | **Total** |
| [*Alternaria alternata*](https://blast.ncbi.nlm.nih.gov/Blast.cgi) | 0 | 0 | 0 | 1 | 0 | 0 | 0 | 0 | 0 | 1 | **2** |
| [*Alternaria tenuissima*](https://blast.ncbi.nlm.nih.gov/Blast.cgi) | 0 | 0 | 0 | 0 | 0 | 0 | 0 | 0 | 0 | 3 | **3** |
| [*Ascochyta medicaginicola*](https://blast.ncbi.nlm.nih.gov/Blast.cgi) | 1 | 0 | 0 | 0 | 0 | 0 | 0 | 0 | 0 | 0 | **1** |
| [*Aureobasidium pullulans*](https://blast.ncbi.nlm.nih.gov/Blast.cgi) | 0 | 0 | 1 | 0 | 0 | 0 | 0 | 0 | 0 | 0 | **1** |
| [*Botrytis cinerea*](https://blast.ncbi.nlm.nih.gov/Blast.cgi) | 0 | 1 | 0 | 0 | 0 | 0 | 0 | 0 | 0 | 0 | **1** |
| [*Cladosporium allicinum*](https://blast.ncbi.nlm.nih.gov/Blast.cgi) | 0 | 0 | 0 | 0 | 0 | 1 | 0 | 0 | 0 | 0 | **1** |
| [*Cladosporium cladosporioides*](https://blast.ncbi.nlm.nih.gov/Blast.cgi) | 1 | 0 | 0 | 1 | 0 | 0 | 0 | 0 | 1 | 4 | **7** |
| [*Cladosporium tenuissimum*](https://blast.ncbi.nlm.nih.gov/Blast.cgi) | 0 | 0 | 1 | 0 | 0 | 0 | 0 | 0 | 0 | 0 | **1** |
| [*Didymella macrostoma*](https://blast.ncbi.nlm.nih.gov/Blast.cgi) | 0 | 0 | 0 | 0 | 1 | 0 | 0 | 0 | 0 | 0 | **1** |
| [*Periconia byssoides*](https://blast.ncbi.nlm.nih.gov/Blast.cgi) | 0 | 0 | 0 | 0 | 0 | 0 | 0 | 0 | 0 | 1 | **1** |
| [*Epicoccum nigrum*](https://blast.ncbi.nlm.nih.gov/Blast.cgi) | 1 | 0 | 0 | 0 | 0 | 0 | 0 | 0 | 0 | 0 | **1** |
| *Fusarium equiseti* | 0 | 0 | 0 | 0 | 0 | 0 | 0 | 0 | 0 | 1 | **1** |
| *Hypoxylon macrocarpum* | 0 | 0 | 0 | 1 | 0 | 0 | 0 | 0 | 0 | 0 | **1** |
| [*Penicillium polonicum*](https://blast.ncbi.nlm.nih.gov/Blast.cgi) | 1 | 0 | 0 | 0 | 0 | 0 | 0 | 0 | 0 | 1 | **2** |
| [*Peyronellaea glomerata*](https://blast.ncbi.nlm.nih.gov/Blast.cgi) | 0 | 0 | 1 | 0 | 0 | 0 | 0 | 0 | 0 | 0 | **1** |
| [*Rhodotorula diobovata*](https://blast.ncbi.nlm.nih.gov/Blast.cgi) | 0 | 0 | 0 | 0 | 0 | 0 | 0 | 0 | 0 | 1 | **1** |
| [*Talaromyces funiculosus*](https://blast.ncbi.nlm.nih.gov/Blast.cgi) | 0 | 0 | 3 | 0 | 0 | 1 | 0 | 0 | 1 | 0 | **5** |
| [*Talaromyces purpureogenus*](https://blast.ncbi.nlm.nih.gov/Blast.cgi) | 0 | 0 | 0 | 0 | 0 | 0 | 0 | 1 | 0 | 0 | **1** |
| [*Talaromyces rogersiae*](https://blast.ncbi.nlm.nih.gov/Blast.cgi) | 0 | 0 | 0 | 1 | 0 | 0 | 0 | 0 | 0 | 0 | **1** |
| [*Talaromyces verruculosus*](https://blast.ncbi.nlm.nih.gov/Blast.cgi) | 1 | 0 | 0 | 1 | 0 | 0 | 0 | 1 | 0 | 1 | **4** |
| **Total** | **5** | **1** | **6** | **5** | **1** | **2** | **0** | **2** | **2** | **13** | **37** |
|  | **Apr 2021** | | | | | | | | | | |
| **Fungal Species** | **3CP** | **3CO** | **5CP** | **5CO** | **CDP** | **CDO** | **FDP** | **FDO** | **LIB** | **PS** | **Total** |
| [*Alternaria alternata*](https://blast.ncbi.nlm.nih.gov/Blast.cgi) | 0 | 0 | 1 | 2 | 1 | 1 | 0 | 0 | 0 | 0 | **5** |
| [*Alternaria compacta*](https://blast.ncbi.nlm.nih.gov/Blast.cgi) | 0 | 0 | 0 | 0 | 0 | 1 | 0 | 0 | 0 | 1 | **2** |
| [*Alternaria solani*](https://blast.ncbi.nlm.nih.gov/Blast.cgi) | 0 | 0 | 0 | 0 | 0 | 1 | 0 | 0 | 0 | 0 | **1** |
| *Aspergillus flavus* | 1 | 0 | 0 | 0 | 0 | 0 | 0 | 0 | 0 | 0 | **1** |
| [*Aureobasidium pullulans*](https://blast.ncbi.nlm.nih.gov/Blast.cgi) | 0 | 0 | 0 | 1 | 0 | 0 | 0 | 0 | 0 | 1 | **2** |
| *Botrytis cinerea* | 0 | 2 | 0 | 0 | 0 | 0 | 0 | 0 | 0 | 0 | **2** |
| [*Chaetomium globosum*](https://blast.ncbi.nlm.nih.gov/Blast.cgi) | 0 | 0 | 0 | 0 | 0 | 0 | 0 | 0 | 1 | 0 | **1** |
| *Cladosporium allicinum* | 0 | 0 | 1 | 0 | 0 | 0 | 0 | 0 | 0 | 0 | **1** |
| [*Cladosporium cladosporioides*](https://blast.ncbi.nlm.nih.gov/Blast.cgi) | 2 | 1 | 1 | 0 | 0 | 1 | 1 | 0 | 0 | 0 | **6** |
| [*Cladosporium cucumerinum*](https://blast.ncbi.nlm.nih.gov/Blast.cgi) | 0 | 0 | 0 | 0 | 1 | 0 | 0 | 0 | 0 | 0 | **1** |
| [*Cladosporium herbarum*](https://blast.ncbi.nlm.nih.gov/Blast.cgi) | 0 | 0 | 0 | 0 | 0 | 0 | 0 | 0 | 1 | 1 | **2** |
| [*Cladosporium tenuissimum*](https://blast.ncbi.nlm.nih.gov/Blast.cgi) | 0 | 1 | 0 | 0 | 0 | 0 | 0 | 0 | 0 | 0 | **1** |
| [*Cladosporium velox*](https://blast.ncbi.nlm.nih.gov/Blast.cgi) | 0 | 0 | 0 | 0 | 0 | 1 | 0 | 0 | 0 | 0 | **1** |
| [*Cryptococcus chernovii*](https://blast.ncbi.nlm.nih.gov/Blast.cgi) | 0 | 0 | 0 | 0 | 0 | 0 | 0 | 0 | 0 | 1 | **1** |
| [*Didymella pinodella*](https://blast.ncbi.nlm.nih.gov/Blast.cgi) | 0 | 0 | 0 | 0 | 0 | 0 | 0 | 0 | 0 | 1 | **1** |
| [*Kwoniella shandongensis*](https://blast.ncbi.nlm.nih.gov/Blast.cgi) | 1 | 0 | 0 | 0 | 0 | 0 | 0 | 0 | 0 | 0 | **1** |
| [*Latorua caligans*](https://blast.ncbi.nlm.nih.gov/Blast.cgi) | 0 | 0 | 0 | 0 | 0 | 0 | 0 | 0 | 0 | 1 | **1** |
| [*Naganishia albida*](https://blast.ncbi.nlm.nih.gov/Blast.cgi) | 1 | 0 | 0 | 0 | 0 | 0 | 0 | 0 | 1 | 0 | **2** |
| [*Nothophoma spiraeae*](https://blast.ncbi.nlm.nih.gov/Blast.cgi) | 0 | 0 | 0 | 0 | 0 | 0 | 0 | 0 | 0 | 1 | **1** |
| [*Penicillium expansum*](https://blast.ncbi.nlm.nih.gov/Blast.cgi) | 0 | 0 | 1 | 0 | 0 | 0 | 0 | 0 | 0 | 0 | **1** |
| [*Phoma medicaginis*](https://blast.ncbi.nlm.nih.gov/Blast.cgi) | 0 | 0 | 0 | 0 | 0 | 0 | 0 | 1 | 0 | 0 | **1** |
| [*Pithomyces chartarum*](https://blast.ncbi.nlm.nih.gov/Blast.cgi) | 0 | 0 | 0 | 0 | 0 | 0 | 0 | 0 | 1 | 0 | **1** |
| [*Schizophyllum commune*](https://blast.ncbi.nlm.nih.gov/Blast.cgi) | 0 | 0 | 0 | 0 | 0 | 0 | 0 | 0 | 1 | 0 | **1** |
| **Total** | **5** | **4** | **4** | **3** | **2** | **5** | **1** | **1** | **5** | **7** | **37** |
|  | **May 2021** | | | | | | | | | | |
| **Fungal Species** | **3CP** | **3CO** | **5CP** | **5CO** | **CDP** | **CDO** | **FDP** | **FDO** | **LIB** | **PS** | **Total** |
| [*Actinomucor elegans*](https://blast.ncbi.nlm.nih.gov/Blast.cgi) | 0 | 0 | 0 | 0 | 1 | 0 | 0 | 0 | 0 | 0 | **1** |
| [*Alternaria alternata*](https://blast.ncbi.nlm.nih.gov/Blast.cgi) | 0 | 0 | 0 | 2 | 0 | 0 | 0 | 0 | 0 | 2 | **4** |
| [*Alternaria angustiovoidea*](https://blast.ncbi.nlm.nih.gov/Blast.cgi) | 0 | 0 | 1 | 0 | 0 | 0 | 0 | 0 | 0 | 1 | **2** |
| [*Alternaria* sp.](https://blast.ncbi.nlm.nih.gov/Blast.cgi) | 0 | 0 | 0 | 0 | 0 | 0 | 0 | 0 | 0 | 1 | **1** |
| [*Alternaria tamaricis*](https://blast.ncbi.nlm.nih.gov/Blast.cgi) | 0 | 0 | 0 | 0 | 1 | 0 | 0 | 0 | 0 | 0 | **1** |
| [*Aureobasidium pullulans*](https://blast.ncbi.nlm.nih.gov/Blast.cgi) | 0 | 1 | 0 | 0 | 0 | 0 | 0 | 0 | 0 | 0 | **1** |
| [*Candida parapsilosis*](https://blast.ncbi.nlm.nih.gov/Blast.cgi) | 0 | 0 | 0 | 1 | 0 | 0 | 0 | 0 | 0 | 0 | **1** |
| [*Cladosporium asperulatum*](https://blast.ncbi.nlm.nih.gov/Blast.cgi) | 0 | 0 | 0 | 0 | 0 | 0 | 1 | 0 | 0 | 0 | **1** |
| [*Cladosporium cladosporioides*](https://blast.ncbi.nlm.nih.gov/Blast.cgi) | 0 | 0 | 0 | 0 | 0 | 1 | 0 | 0 | 0 | 1 | **2** |
| [*Cladosporium herbarum*](https://blast.ncbi.nlm.nih.gov/Blast.cgi) | 0 | 0 | 0 | 1 | 0 | 0 | 1 | 0 | 0 | 0 | **2** |
| [*Cladosporium macrocarpum*](https://blast.ncbi.nlm.nih.gov/Blast.cgi) | 0 | 0 | 0 | 0 | 0 | 0 | 0 | 0 | 1 | 0 | **1** |
| [*Cladosporium sinuosum*](https://blast.ncbi.nlm.nih.gov/Blast.cgi) | 0 | 0 | 0 | 0 | 1 | 0 | 0 | 0 | 0 | 0 | **1** |
| [*Cladosporium* sp.](https://blast.ncbi.nlm.nih.gov/Blast.cgi) | 0 | 1 | 0 | 0 | 0 | 2 | 0 | 0 | 0 | 0 | **3** |
| [*Cladosporium sphaerospermum*](https://blast.ncbi.nlm.nih.gov/Blast.cgi) | 0 | 0 | 1 | 0 | 0 | 0 | 0 | 0 | 0 | 0 | **1** |
| [*Coniothyrium aleuritis*](https://blast.ncbi.nlm.nih.gov/Blast.cgi) | 0 | 0 | 0 | 0 | 0 | 0 | 0 | 1 | 0 | 0 | **1** |
| [*Coprinopsis kubickae*](https://blast.ncbi.nlm.nih.gov/Blast.cgi) | 0 | 0 | 0 | 0 | 1 | 0 | 0 | 0 | 0 | 0 | **1** |
| [*Cryptococcus magnus*](https://blast.ncbi.nlm.nih.gov/Blast.cgi) | 1 | 0 | 0 | 0 | 0 | 0 | 0 | 0 | 0 | 0 | **1** |
| [*Curvularia kusanoi*](https://blast.ncbi.nlm.nih.gov/Blast.cgi) | 0 | 0 | 0 | 0 | 0 | 0 | 0 | 0 | 1 | 0 | **1** |
| [*Didymella glomerata*](https://blast.ncbi.nlm.nih.gov/Blast.cgi) | 0 | 1 | 0 | 0 | 0 | 0 | 0 | 0 | 0 | 0 | **1** |
| [*Didymella pinodella*](https://blast.ncbi.nlm.nih.gov/Blast.cgi) | 0 | 0 | 0 | 0 | 0 | 0 | 1 | 0 | 0 | 0 | **1** |
| [*Didymella* sp.](https://blast.ncbi.nlm.nih.gov/Blast.cgi) | 0 | 1 | 0 | 0 | 0 | 0 | 0 | 0 | 0 | 0 | **1** |
| [*Camarosporium* sp.](https://blast.ncbi.nlm.nih.gov/Blast.cgi) | 0 | 0 | 0 | 0 | 0 | 0 | 1 | 0 | 0 | 0 | **1** |
| [*Dothiorella gregaria*](https://blast.ncbi.nlm.nih.gov/Blast.cgi) | 0 | 0 | 0 | 0 | 0 | 0 | 0 | 1 | 0 | 0 | **1** |
| [*Dothiorella* sp.](https://blast.ncbi.nlm.nih.gov/Blast.cgi) | 0 | 0 | 1 | 0 | 0 | 0 | 0 | 0 | 0 | 0 | **1** |
| [*Filobasidium magnum*](https://blast.ncbi.nlm.nih.gov/Blast.cgi) | 1 | 0 | 0 | 0 | 0 | 0 | 0 | 0 | 0 | 0 | **1** |
| [*Leptosphaerulina australis*](https://blast.ncbi.nlm.nih.gov/Blast.cgi) | 0 | 0 | 0 | 0 | 0 | 0 | 0 | 1 | 0 | 0 | **1** |
| [*Naganishia albida*](https://blast.ncbi.nlm.nih.gov/Blast.cgi) | 0 | 0 | 0 | 0 | 0 | 0 | 0 | 1 | 0 | 0 | **1** |
| [*Naganishia* sp.](https://blast.ncbi.nlm.nih.gov/Blast.cgi) | 0 | 0 | 0 | 0 | 0 | 0 | 0 | 0 | 0 | 1 | **1** |
| [*Naganishia uzbekistanensis*](https://blast.ncbi.nlm.nih.gov/Blast.cgi) | 0 | 0 | 0 | 0 | 0 | 0 | 0 | 1 | 0 | 0 | **1** |
| [*Penicillium olsonii*](https://blast.ncbi.nlm.nih.gov/Blast.cgi) | 0 | 0 | 0 | 0 | 0 | 0 | 1 | 0 | 0 | 0 | **1** |
| [*Periconia macrospinosa*](https://blast.ncbi.nlm.nih.gov/Blast.cgi) | 0 | 0 | 0 | 0 | 0 | 0 | 0 | 1 | 0 | 0 | **1** |
| [*Peyronellaea glomerata*](https://blast.ncbi.nlm.nih.gov/Blast.cgi) | 0 | 0 | 0 | 1 | 0 | 0 | 0 | 0 | 0 | 0 | **1** |
| [*Stagonospora*](https://blast.ncbi.nlm.nih.gov/Blast.cgi) | 0 | 0 | 0 | 1 | 0 | 0 | 0 | 0 | 0 | 0 | **1** |
| [*Phoma fungicola*](https://blast.ncbi.nlm.nih.gov/Blast.cgi) | 0 | 0 | 0 | 0 | 0 | 0 | 1 | 0 | 0 | 0 | **1** |
| [*Phoma* sp.](https://blast.ncbi.nlm.nih.gov/Blast.cgi) | 0 | 0 | 0 | 0 | 0 | 0 | 0 | 1 | 0 | 0 | **1** |
| [*Pleosporales* sp.](https://blast.ncbi.nlm.nih.gov/Blast.cgi) | 0 | 1 | 0 | 0 | 0 | 0 | 0 | 0 | 0 | 0 | **1** |
| [*Purpureocillium lilacinum*](https://blast.ncbi.nlm.nih.gov/Blast.cgi) | 0 | 0 | 0 | 0 | 0 | 0 | 0 | 0 | 0 | 1 | **1** |
| [*Scheleobrachea quadrata*](https://blast.ncbi.nlm.nih.gov/Blast.cgi) | 0 | 0 | 1 | 0 | 0 | 0 | 0 | 0 | 0 | 0 | **1** |
| **Total** | **2** | **5** | **4** | **6** | **4** | **3** | **6** | **7** | **2** | **7** | **46** |

**Table S8.** Permutational multivariate analysis showing the effects temperature and relative humidity on fungal community abundance.

| Factor | Df | F | R2 | *p*-value |
| --- | --- | --- | --- | --- |
| Relative humidity | 1 | 1.119726605 | 0.02871894 | 0.324 |
| Temperature | 1 | 0.86940769 | 0.022298717 | 0.591 |
| Residuals | 37 | - | 0.948982343 | - |
| Total | 39 | - | 1 | - |
